# Computational Prediction of Hot Spots and Binding Site of Inhibitor NSC23766 on Rac1 Binding With Tiam1

**DOI:** 10.3389/fchem.2020.625437

**Published:** 2021-02-02

**Authors:** Chunwen Zheng, Xiaodong Wu, Ruijie Zeng, Lirui Lin, Liyan Xu, Enmin Li, Geng Dong

**Affiliations:** ^1^ Shantou University Medical College, Shantou, China; ^2^ Department of Biochemistry and Molecular Biology, Shantou University Medical College, Shantou, China; ^3^ Medical Informatics Research Center, Shantou University Medical College, Shantou, China; ^4^ Key Laboratory of Molecular Biology in High Cancer Incidence Coastal Area of Guangdong Higher Education Institutes, Shantou University Medical College, Shantou, China

**Keywords:** Rac1, Tiam1, NSC23766, molecular docking, MD simulation, binding free energy

## Abstract

Rac1 is a small signaling protein, which belongs to the Rho subfamily of Ras superfamily. It is activated by binding GTP and inactivated by exchanging GDP for GTP. The ability of nucleotide exchange depends on guanine nucleotide exchange factors (GEFs) family proteins. T-lymphoma invasion and metastasis factor 1 (Tiam1) is a member of GEFs. Rac1 participates in multiple signaling pathways and regulates various cellular events by interacting with GEFs. Particularly, it is involved in the development and progression of various kinds of tumors. In this paper, we have studied the detailed interaction between Rac1 and Tiam1. Seven residues on Rac1 are predicted to be important for the interaction with Tiam1, i.e. E31, Y32, D38, N39, Y64, D65 and W56. All these residues are located on the switch 1 and 2 domains which are the interface between Rac1 and Tiam1, except W56. In addition, we analyzed how inhibitor NSC23766 interacts with Rac1. Our docking results show that NSC23766 binds to the same region as Tiam1. Several residues, i.e. F37, D38, N39, W56, Y64, L67, L70 and S71, contribute much to binding free energy. These findings are very useful for the structure-based design of inhibitors toward Rac1.

## Introduction

Rac1, a member of Rho family GTPase, is found to involve in the development and progression of various kinds of tumors (Myant et al., 2013; Wang et al., 2015). Rac1 is reported to play both tumor-promoting role and tumor-suppression role in certain kinds of tumors, which indicates that the function of Rac1 remains controversial and is related to a complex network of tumor regulation (Engers et al., 2001; Malliri et al., 2002; Baugher et al., 2005; Chen et al., 2011; Frances et al., 2015). Similar to the other Rho GTPases, Rac1 could causes chemoresistance in various kinds of cancers. It can be turned on and off as a molecular switch and transformed between inactive state with guanosine diphosphate (GDP) and active state with guanosine triphosphate (GTP) (Um et al., 2014; Cardama et al., 2017). When Rac1 is activated, it launches a broad spectrum of downstream pathways and involves in modulating various processes of cancer progression, including cytoskeletal reorganization, migration, invasion and metastasis (Kamai et al., 2010; Myant et al., 2013; Wang et al., 2015). Recently, we found that Rac1 played an important role of tumor-promoting in the progression of esophageal squamous cell carcinoma (ESCC) and the expression of Rac1 was positively related to cisplatin resistance in ESCC cells (Zeng et al., 2019). Therefore, inhibiting Rac1 is a solution to inhibit progression of tumors.

Tiam1 is one of the guanine nucleotide exchange factors (GEFs) those activate small GTPases transforming from GDP-bound to GTP-bound state, leading to a tumor-promoting effect (Um et al., 2014; Ruihua et al., 2016; Cardama et al., 2017). In 1994, Habets and his coworkers first identified Tiam1 and found that Tiam1 confers an invasive phenotype to murine T-lymphoma cells (Habets et al., 1994). In the same work, they reported that the function of Tiam1 is mainly for the activation of Rac1 by stimulating the exchange of GDP for GTP. During this process, the structure of Tiam1 was suggested to be adjusted for recognition and binding to Rac1 (Habets et al., 1994; Boissier and Huynh-Do, 2014).

In order to investigate the interaction between Rac1 and Tiam1, Worthylake et al. determined the crystal structure of DH and PH domains of Tiam1 in complex with Rac1 (David et al., 2000). The crystal structure of Tiam1-Rac1 complex provides novel insights into developing therapeutic strategy to inhibit Rho GTPase activity and the associated downstream pathways (Akbar et al., 2006). By blocking the interactions between Rho GTPases and the specific Dbl family GEFs, the Rho GTPase activity is inhibited and the progression of cancer cells may be arrested.

Based on the crystal structure of Tiam1- Rac1 complex, the chemical compound NSC23766 was found to targeting to Rac1 by Gao et al. using computational method (Gao et al., 2004). NSC23766 is the first and most widely used Rac1 inhibitor that binding to Rac1-GEF interface (Levay et al., 2013; Dütting et al., 2015). Owing to the discovery of Rac1 activation-specific inhibitor, research on cell biological studies of Rac1 functions and chemotherapeutic targeting at Rac1 dysregulation is largely promoted (Gao et al., 2004). Recently, our study demonstrated that inhibition of Rac1 resulted in a significant effect in reversing chemoresistance in ESCC on molecular, cellular and xenograft mice model levels (Zeng et al., 2019). However, the detailed mechanism is unclear.

Despite the crystal structure of Rac1-Tiam1 complex has be solved, the key residues (hot spots) that affect the interaction between Rac1 and Tiam1 is not fully identified. In particular, the mechanism of the binding of NSC23766 has not been systematical studied. In this work, we have studied the detailed interactions between Rac1 and Tiam1 and between Rac1 and NSC23766, and predicted the hot spots on Rac1 by theoretical methods. This study provides valuable insights for the cancer treatment based on the inhibition of Rac1.

## Methods

### Molecular Docking

To obtain the complex of Rac1 with NSC23766, we performed the molecular docking calculations. The structure of Rac1 was used with PDB ID: 5N6O (Ferrandez et al., 2017). Since different scoring functions are used in different docking programs, docking calculations were carried out with three docking programs to make a comparison, i.e. Autodock4, Autodock Vina and HDCOK (Pearson and Lipman, 1988; Berman et al., 2000; Martí-Renom et al., 2000; Larkin et al., 2007; Morris et al., 2009; Trott and Olson, 2010; Remmert et al., 2011; Sievers et al., 2011). The former two programs are implemented in the AMDock software (Valdés-Tresanco et al., 2020). For Autudock 4 and Autodock Vina, the grid boxes were set to the same position as it is in the study by Gao et al. (2004), whereas automatic searching mode was used for HDOCK. All docking calculations were carried out with default parameters. Finally, the best pose from each docking program was used for further studies.

### Molecular Dynamics Simulations

The calculation of Rac1-Tiam1 complex was based on the 2.8 Å crystal structure (PDB ID: 1FOE) (David et al., 2000) and Rac1-NSC23766 complex was based on the 2.59 Å crystal structure (PDB ID: 5N6O) (Ferrandez et al., 2017). The Rac1-Tiam1 complex and Rac1-NSC23766 were solvated in a truncated octahedral box extending at least 10 Å on all sides of the complex. The protonation states of all the residues were determined by using PROPKA (Bas et al., 2010), a study of the hydrogen-bond pattern around the His residues, the solvent accessibility, and the possible formation of ionic pairs. All Arg, Lys, Asp, and Glu residues were assumed to be charged. As for histidine, in the Rac1-Tiam1 complex, His1149, 1178, 1216 and 1333 of Tiam1 and His104 of Rac1 were assumed be protonated on ND1, and His1214 of Tiam1 was assumed to be protonated on the NE2 atom, besides remaining His residues were modeled as doubly protonated. In the Rac1-NSC23766 complexes, we assumed His104 to be protonated on the ND1 atom and His103 as doubly protonated.

All the simulations were run using the pmemd module in Amber 18 (Case et al. 2018). By using Langevin dynamics (Wu and Brooks, 2003) we kept the temperate at 300 K, and a collision frequency of 2.0 ps^−1^, the pressure was kept constant at 1 atm using a weak-coupling isotropic algorithm with a relaxation time of 1 ps (Berendsen et al., 1984). Particle mesh Ewald summation with a fourth-order B spline interpolation and a tolerance of 10^–5^ was used to handle the long-range electrostatics (Darden et al., 1993). The SHAKE algorithm (Ryckaert et al., 1977) was used to constrain bonds involving hydrogen atoms so that a 2 fs time step could be used. The cutoff for nonbonded interactions was set to 8 Å. The ff14SB force field (Maier et al., 2015) was used for protein and the protein and ligand were solvated in a truncated octahedral box of TIP3P molecules, extending 10 Å from the protein and ligand (Gillan et al., 2016). The AM1-BCC atomic charges (Jakalian et al., 2000; Jakalian et al., 2002) were assigned to each atom of ligand by using the antechamber module (Junmei et al., 2010). the general AMBER force field (GAFF) were used for NSC23776 (Wang et al., 2004).

All simulations were started by a 1000-step minimization, and followed by 20 ps constant-volume equilibration and 20 ps constant-pressure equilibration. The heavy atoms of protein were restrained during the equilibration. Then, 1ns constant-pressure equilibration was performed without any restraints. Finally, the 10-ns production simulation was carried out and coordinates were saved every 2 ps. For the Rac1-Tiam1 system, seven replicates of production calculations were run and three replicates were run for Rac1-NSC23776 system.

Finally we calculated the root mean square deviation (RMSD) and root mean square fluctuation (RMSF) using the *cpptrajy* module in AMBER 18 (Case et al. 2018).

### The Relative Binding Free Energy With Alanine Scanning

Alanine-scanning calculations were performed for Rac1-Tiam1 system. The alanine-mutant trajectory was obtained from the trajectory of MD simulation for wild-type protein. This strategy of Ala scanning is the simplest and most efficient approach that has been shown to give reasonable results (Kuhn and Kollman, 2000; Huo et al., 2001; Moreira et al., 2007). In this work, we run Ala-scanning calculations for the seven parallel trajectories. Frames from 1,000 to 5,000 were select for alanine scanning calculations. Finally, the average and standard deviation of the binding free energies were calculated.

### Absolute Binding Free Energy Calculations With MM/GBSA

Conventional molecular dynamics simulations were conducted to generate a representative ensemble of structures, and MM/GBSA method was then used to calculate absolute binding free energies of NSC23776 binding to Rac1 and Tiam1 binding to Rac1. In this method, the calculation of Δ*G*_bind_ can be expressed as follow:

$$\Delta G_{\text{bind}}\;=\;G_{\text{complex}}\;–\;G_{\text{protein}}\;–\;G_{\text{ligand}}\;$$ (1)

where the binding free energy (Δ*G*_bind_) is computed as the difference between the free energies of the complex (*G*_complex_), the protein (*G*_protein_) and the ligand (*G*_ligand_).

For all systems, snapshots from the last 8-ns MD trajectory were used. To evaluate the contribution of specific residues to binding free energies, a residue-based free energy decomposition method was employed to calculate the complexes interaction spectrum by using the same snapshots as those used in MM/GBSA calculations (Gohlke et al., 2003).

## Result

### Interaction Analysis Based on MD Simulation

**FIGURE 1:**
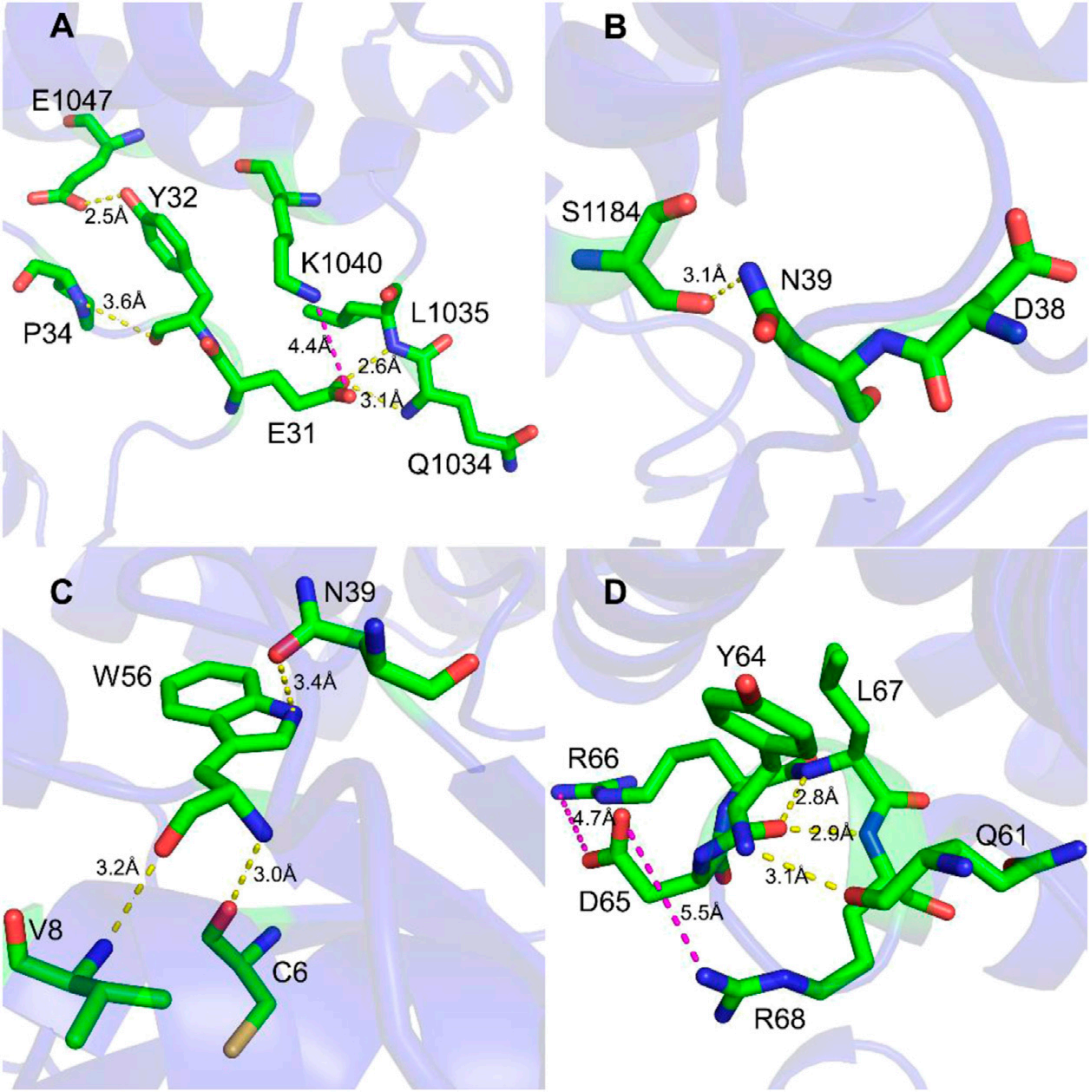
Interaction detail of hot spots in Rac1-Tiam1 structure. The yellow dotted lines represent hydrogen bond, and magenta dotted lines represent salt-bridge interaction. **(A,B)** show interactions formed by residues on switch 1 of Rac1. **(A)** Interaction around residue E31_R_ and Y32_R_. **(B)** Interaction around residue N39_R_. **(C,D)** show the intermolecular interactions of switch 2 of Rac1. **(C)** Interactions around residue W56_R_. **(D)** Interactions around residue Y64_R_ and D65_R_.

To study the interaction between Rac1 and Tiam1, we first performed MD simulation for the Rac1-Tiam1 complex. In the complex, the switch 1 and 2 domains of Rac1 might be important regions in the interaction with Tiam1, because the two domains locate on the interface of the two proteins. First, we focused on the residues on the switch 1 domain. As is shown in Figure 1A, E31_R_ (subscript R means that residue belongs Rac1) forms hydrogen bond with Q1034_T_ and L1035_T_ (subscript T means that residue belongs Tiam1), and salt bridge with K1040_T_. Y32_R_ forms hydrogen bond with E1047_T_ and P34_R_. This local area contains abundant intermolecular interactions, indicating that the residues on Rac1 in this region are important for the binding with Tiam1, and the mutation of these residues may significantly influence the function of Rac1. In addition, the residue N39_R_ forms hydrogen bond with S1184_T_ (Figure 1B). Next, we analyzed the intermolecular interactions in switch 2 domain. Residues of C6, V8, N39 and W56 on Rac1 directly interact with Tiam1. N39_R_ and W56_R_ have been validated by previous studies (Gao et al., 2002; Gao et al., 2004). W56_R_ forms three hydrogen bonds with the other three residues, i.e. N39_R_, V8_R_ and C6_R_. In addition, the intermolecular interactions formed by other residues in switch 2 domain were shown in Figure 1D. Y64_R_ forms hydrogen bonds with Q61_R_, L67_R_ and R68_R_, and D65_R_ forms salt bridges with R66_R_ and R68_R_. Thus, our results show that residues in switch 1 and 2 domains of Rac1 play an important role to binding with Tiam1.

### Prediction of Hot Spots on Rac1

In the study by Yi Zheng et al., the binding interaction assay shown that E31, Y32, D38 and N39 have strong effect on Rac1 binding to TrioN (GEF-H1, another Rac1 binding protein) and binding affinity will be significantly decreased by mutation to alanine (Gao et al., 2002). The mutants Y64A and D65A were also found to decrease the binding affinity of Rac1 interacting with TrioN, experimentally (Gao et al., 2002). As we have shown above, E31, Y32, N39, Y64, D65 and W56 have many interactions with the adjacent residues during the MD simulations. We then performed alanine scanning calculations for those seven residues on Rac1, i.e. E31, Y32, D38, N39, W56, Y64 and D65. The alanine scanning results are collected in Table 1. Our calculations show that all mutants decrease the binding affinity of Rac1. D38A and N39A have stronger effect on Rac1-Tiam1 system than E31A in switch 1 domain and Y64A has a larger effect than D65A in switch 2. Our result have a good agreement with experimental data (Gao et al., 2002). In addition, the W56A mutant have the largest effect in our calculations.

**TABLE 1:** Computational alanine scanning results for Rac1-Tiam1 complex.

Mutant	Domain	ΔΔG^a^ (kcal/mol)
E31A	Switch 1	−4.9 ± 1.6
Y32A	Switch 1	−8.6 ± 0.8
D38A	Switch 1	−6.5 ± 0.5
N39A	Switch 1	−6.7 ± 0.5
Y64A	Switch 2	−6.0 ± 0.5
D65A	Switch 2	−4.3 ± 1.5
W56A	Close to switch 2	−12.6 ± 0.6

^a^ ΔΔG = ΔG (wild type) − ΔG (mutant).

**FIGURE 2:**
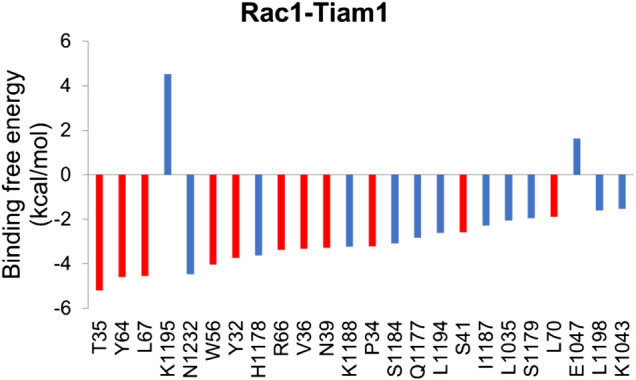
Residue-specific binding free energy for Rac1-Tiam1 complex. The red columns represent residues on Rac1 and blue columns represent residues on Tiam1.

In order to understand the contribution of each residue on Rac1 to the total binding free energy, residue-based free energy decomposition was carried out. In WT Rac1-Tiam1 complex, residues Y32, N39, W56 and Y64 contribute much to the binding free energy, −3.73, −3.28, −4.04, and −4.59 kcal/mol, respectively. The contributions of VDW, electrostatic, polar solvation and nonpolar are shown in Table 2. As we have discussed before that these residues form strong interactions with the residues on Tiam1. In addition, our calculations show that residues T35, L67, R66, V36, P34 and L70 have a large contribution and all of them locate in switch 1 or 2 domains of Rac1(Figure 2). Among these residues, P34, T35 and V36 form hydrogen bond with E1047_T_ on Tiam1, the R66 forms hydrogen bond with N1232_T_ and salt bridge with E1239_T_, the L67 forms hydrogen bond with residues W64 and L70. These interactions might play important roles in Rac1 binding with Tiam1. Therefore, based on previous studies and our calculated results, the switch 1 and 2 domains of Rac1 are important for the binding with Tiam1.

**TABLE 2:** Residue-based free energy decomposition for Rac1-Tiam1 complex (kcal/mol).

Mutant	VDW	Electrostatic	Polar solvation	Nonpolar	Total
Y32	−4.33	−7.22	8.50	−0.67	−3.73
N39	−3.58	−10.28	11.09	−0.51	−3.28
W56	−2.87	−2.53	1.77	−0.40	−4.04
Y64	−5.78	−2.09	4.02	−0.73	−4.59

### Molecular Docking

**FIGURE 3:**
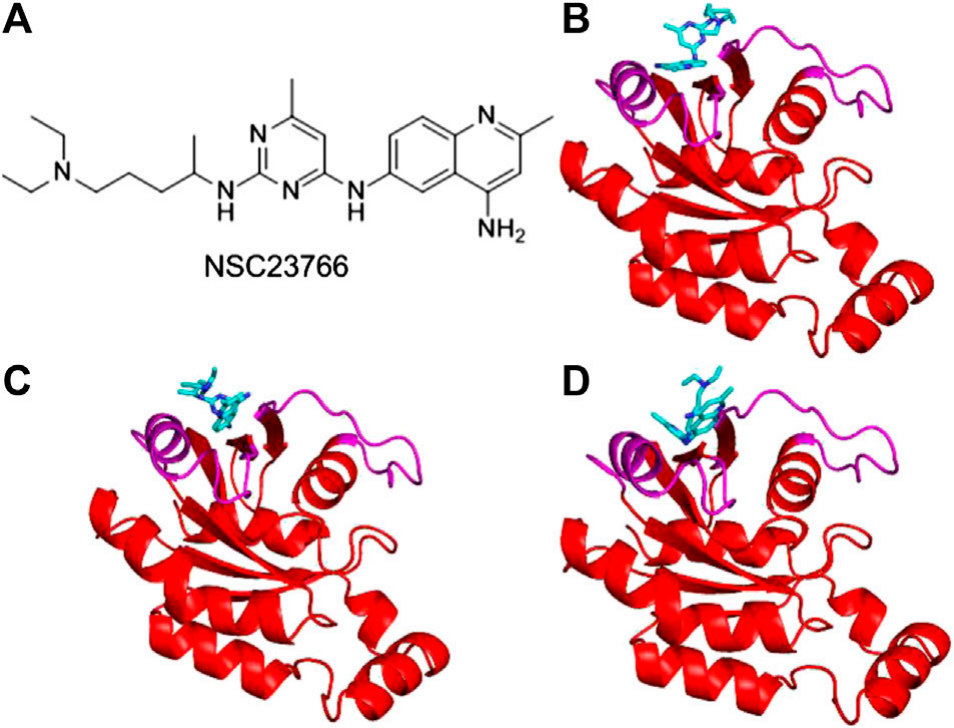
The inhibitor NSC23766 **(A)** and the docking structures of Rac1 with NSC23766 molecule from Autodock **(B)**, Autodock Vina **(C)** and HDock **(D)**. The switch 1 and 2 domains of Rac1 were shown in magenta, the NSC23766 molecule was shown as cyan stick model.

Inhibitor NSC23766 (Figure 3A) was developed by Zheng and coworkers to specifically block the binding of Tiam1 to Rac1(Gao et al., 2004). However, no complex structure of Rac1-NSC23766 was captured so far. Therefore, we performed molecular docking to obtain the complex of Rac1 with NSC23766. In this work, three docking programs were used, i.e. Autodock4, Autodock Vina and HDock. The best pose of each program was selected for the further studies (Figure 3). The regions shown in magenta in Figure 3 are the switch 1 and 2 domains of Rac1. Our docking results show that NSC23766 binds at switch 1 and 2 domains, which are the same regions for Rac1 interacting with Tiam1. For structure from Autodock, NSC23766 forms hydrogen bond with D57, and NSC23766 located at the hydrophobic pocket produced by residue F37, D38, N39, W56, A59, Y64, L67, R68 and S71. For the structure from Vina, NSC23766 forms hydrogen bond with D57, and NSC23766 located at the hydrophobic pocket formed by residue F37, D38, N39, W56, T58, A59, Y64, L67, R68, L70 and S71. For structure from HDock, NSC23766 forms hydrogen bond with D57, and NSC23766 located at the hydrophobic pocket produced by residue F37, D38, N39, W56, A59, Y64, L67, R68, L70 and S71.

### RMSD Analysis

**FIGURE 4:**
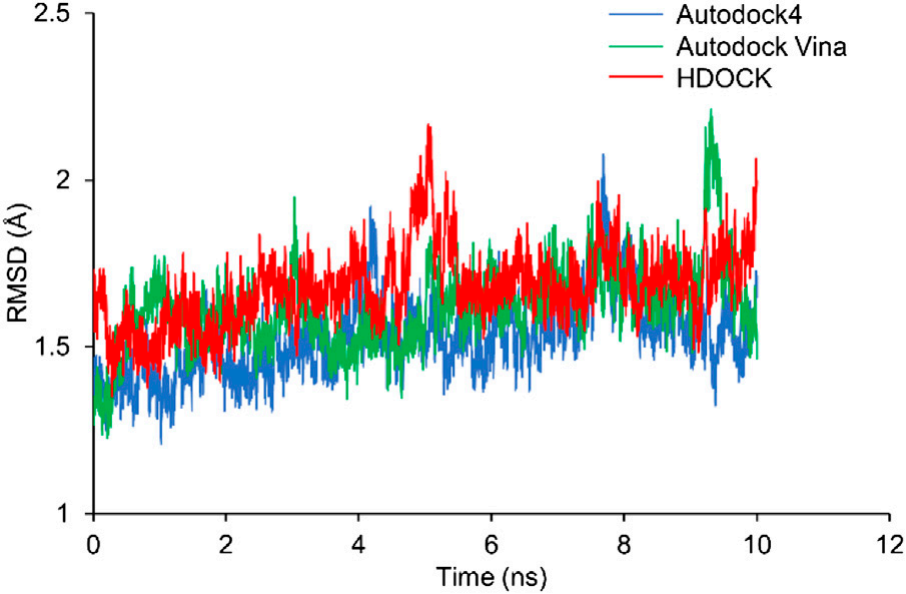
The RMSDs from MD simulations for the three docking structures. The blue, green and red lines are for the structures from Autodock4, Autodock Vina and HDOCK, respectively.

RMSD shows how much the structure differs from the reference structure. The crystal structure of Rac1 (PDB ID: 5N6O) was used as the reference. All the three docking structures were optimized by 10-ns MD simulations, and all simulations were repeated 3 times. RMSD of Rac1 were calculated. Our results show that RMSDs for all the replicates are almost identical (Supplementary Figure S1). Thus, only one of the RMSDs for replicates was selected in Figure 4. It can be seen from Figure 4 that no significant difference is found on Rac1 among the three simulations for three structures, and all of them are around 1.5 Å. Therefore, our results indicate the NSC23766 molecules in the three structures have a similar effect to Rac1.

### RMSF Analysis

**FIGURE 5:**
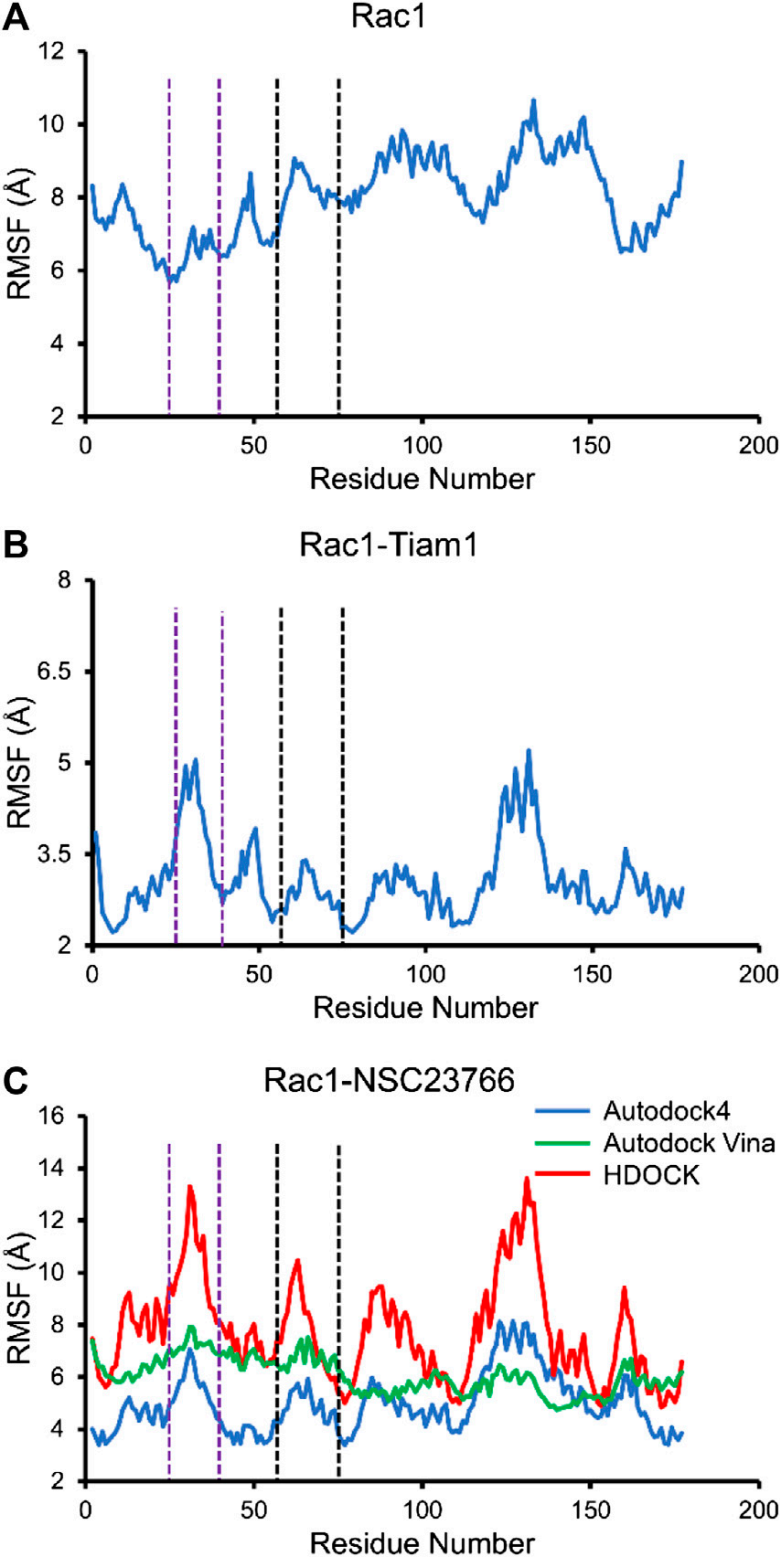
The RMSF for all simulated system. The residues between two purple dotted lines are located at switch 1, and those between two black dotted lines are located at switch 2. **(A)** RMSF of Rac1 in isolated Rac1. **(B)** RMSF of Rac1 in Rac1-Tiam1 system. **(C)** RMSF for three Rac1-NSC23766 complex.

**FIGURE 6:**
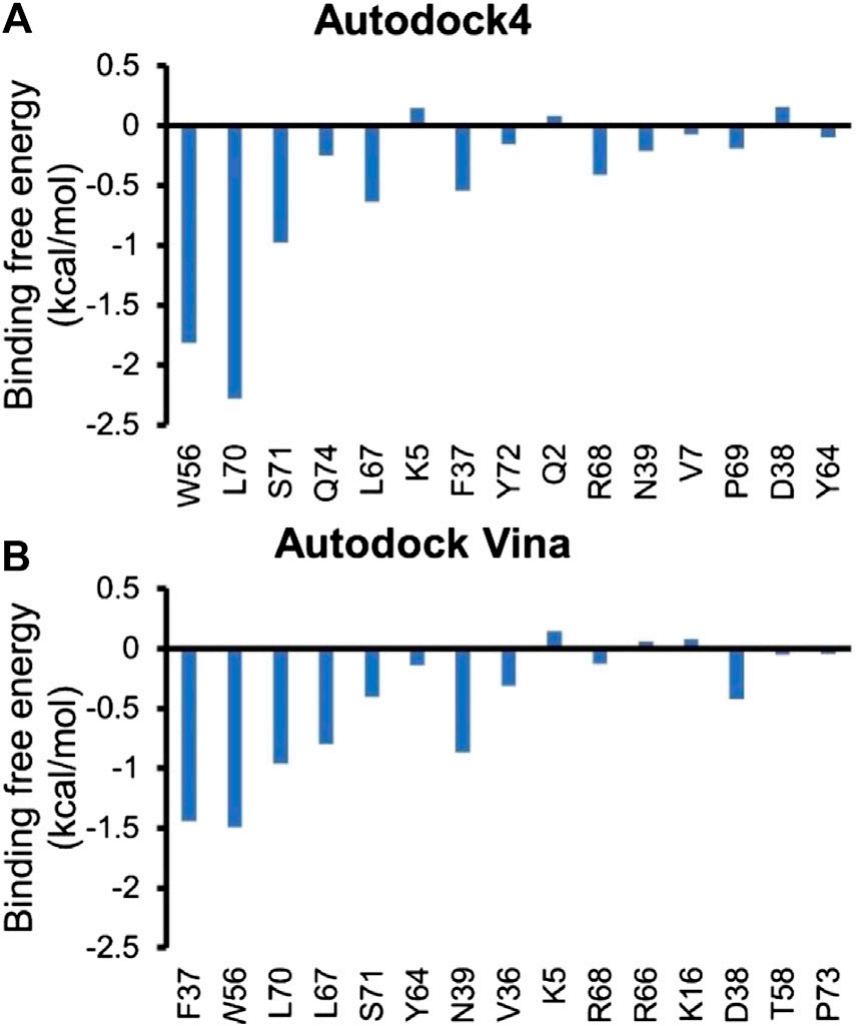
Residue-specific binding free energy for Rac1-NSC23766 complexes.

Next, we calculated the RMSF of Rac1-Tiam1 complex, Rac1-NSC23766 complexes, as well as Rac1 protein. For isolated Rac1, it can be seen from Figure 5A that fluctuations of all residues are above 6 Å and the fluctuations of residues in switch 1 domain (residues 25–39, between purple dotted lines) are lower than those in switch 2 and other regions. When Tiam1 binds to Rac1, (Figure 5B), the fluctuation of the whole system is lower than it in only Rac1 protein. In addition, in Figure 6C, the RMSF of the residues in switch 1 and 2 domains are larger than those residues in other regions, except residues 120–150. In addition, the RMSF of Rac1 from HDOCK is larger than the other two.

### The Prediction of Hot Spots on Rac1 in Rac1-NSC23766 Complex

In this study, three docking programs were used for prediction of structure of Rac1-NSC23766 complex and the best pose from each software was selected. Our docking results show that NSC23766 molecules in the three docking structures reside in the same area with different poses. Next, we calculated the binding free energy for these structures with MM/GBSA, and the results are collected in Table 3. It can be seen that Autodock4 structure gives a lowest binding free energy, which is 0.3 and 2.4 kcal/mol lower than Autodock Vina and HDOCK structures, respectively. The highest binding free energy from HDOCK structure might be caused by the large fluctuation (Figure 5C). Thus, the result from HDOCK suggested to be ignored.

**TABLE 3:** Binding free energy from MM/GBSA for Rac1-Tiam1 complex.

Docking programs	ΔG (kcal/mol)
Autodock4	−16.8
Autodock-Vina	−16.5
HDOCK	−14.4

Next, residue based binding free energy decompositions were calculated. It can be seen from Figure 6 that the residues those have the large contribution to binding locate in switch 1 and 2 domains. Some of the hot spots in Rac1-NSC23766 complex are also important for the interaction between Rac1 and Tiam1, eg W56, L70, etc. Thus, the inhibitor NSC23766 binds to Rac1 and interacts with residues those are important for binding to Tiam1 to blocks the Tiam1 to interact with Rac1.

## Discussion

With the advances in cancer therapeutics, chemoresistance that leads to impaired treatment efficacy or treatment failure, becomes a great challenge of current clinical practice and scientific research. Individualized evaluation and targeted therapy are popularly pursued. Increasing numbers of studies are carried on to identify the key molecules that modulate chemoresistance and to develop the corresponding drugs to reverse the resistance (Gatenby, 2009; Alfarouk et al., 2015). As a member of Rho family GTPases, Rac1 is widely reported to involve in the development of various malignancies and to modulate chemoresistance or enhance chemosensitivity through a wide spectrum of downstream pathways (Shen et al., 2004; Lee et al., 2007; Zeng et al., 2019; Zhou et al., 2019). Since the activation of Rac1 is regulated by GEFs, targeting Rac1-GEF complex is taken advantage to develop cancer therapeutics (Gao et al., 2004; Akbar et al., 2006). By using some small molecules/inhibitors of Rac1, we can prevent the formation of Rac1-GEF complex to inhibit the activation of Rac1.

Previous experimental study has identified some crucial residues that affect the interaction between Rac1 and Tiam1 (Worthylake et al., 2000; Gao et al., 2002). Our work herein confirms the crucial role of these residues based on molecular docking and MD simulations and predicts hot spots on Rac1 and the binding sites of NSC23766 molecule. In this work, three programs, i.e. Autodock, Autodock Vina and HDOCK, were used for docking study and the best pose from each software was selected to discuss the computational prediction of hot spots and binding sites of NSC23766 on Rac1 in Rac1-Tiam1 interaction. Our results show that the structure from Autodock Vina and Autodock4 give low binding free energies. The binding pose predicted by HDOCK gives the highest binding free energy and large RMSF. Hence, the poses from Autodock4 and Autodock Vina are suggested to be the poses for NSC23766 binding with Rac1.

To find hot spots on Rac1, alanine scanning calculations were carried out. Some of these residues were already mentioned as important residues in previous experimental investigations on the interaction between Rac1 and NSC23766. Gao et al., 2002 generated a panel of point mutants at both switch 1 and 2 domains, and found out that D38A and N39A were inactive species for GEF binding activity and GEF responsiveness (Gao et al., 2002). Similarly, Q61L, Y64A and R66A/L67A species lost their binding activity and failed to undergo nucleotide exchange (Gao et al., 2002). Our alanine scanning results (Table 1) agree with the experimental data. We then analyze the detailed interactions and find that all these resides form strong interaction with the residues on Tiam1. In addition, our study predicts that W56 could be a hot spot in both Rac1-Tiam1 and Rac1-NSC23766 complex. Based on the crystal structure, we can find that W56 locates in the region near the switch 2 domain and it interacts with N39, C6 and V8 by forming hydrogen bonds.

To obtain binding free energy contribution by each residue that involves in the interaction, we run residue based binding free energy decomposition, which is illustrated in Figure 6. Our results show that D38, N39, Y64 and L67 contribute much to binding free energy, which is consistent with the previous experimental studies (Gao et al., 2002). In addition, our calculations prediction some more residues that largely contribute the binding free energy, i.e. F37, W56, L70 and S71. Although we mainly focus on switch 1 and 2 domains, the functions and conformational changes of the resting regions of Rac1 are also noteworthy. There could be some allosteric sites far from the binding site. Nevertheless, no experimentally approved regions or residues are reported. Our study highlights these residues in the hope that they can gain attention from the scientific community, since they are likely to be key residues influencing Rac1-GEF and Rac1-NSC23766 interactions.

Accumulating evidence shows that residues in switch 1 and 2 domains of Rac1 play a significant role in Rac1-GEF and Rac1-NSC23766 interactions. Those residues contribute much to the binding free energy. Mutation of those residues could significantly change the structure and activity of Rac1. On the other hand, based on the interactions in this region, we could design new inhibitors to deactivate Rac1.

In conclusion, we have predicted the binding site of NSC23766 on Rac1. Our results demonstrate that the inhibitor resides in the region belongs to switch domains 1 and 2. In addition, some residues on Rac1 are found to be important to interact with Tiam1 and NSC23766. The detailed interactions of those key residues are studied. This would be very important for the development of next-generation drugs which have better effects on blocking Rac1 interacting with GEFs. Thereby, Rac1 activation by Tiam1 can be inhibited. This would be very important for reducing the influences of Rac1 on cellular functions of cancer cells to reverse chemoresistance and improve therapeutic efficacy. With the discovery of novel functions of Rac1 in other diseases, Rac1-GEF inhibitors are prone to provide more promising treatment options. In addition, predictions of hot spots and binding site are fast and cheap methods for guiding site-specific mutations, and these would provide new insights into the detailed mechanism of inhibitor interaction with target protein.

## Data Availability Statement

The raw data supporting the conclusions of this article will be made available by the authors, without undue reservation.

## Author Contributions

All authors listed have made a substantial, direct, and intellectual contribution to the work and approved it for publication.

## Funding

This investigation has been supported by grants from This investigation has been supported by the Special Funds for the Cultivation of Guangdong College Students’ Scientific and Technological Innovation (‘Climbing Program’ Special Funds, pdjh2019a0182), the National Undergraduate Training Program for Innovation and Entrepreneurship (201810560037), ‘Young Physician Scientist Cultivation’ Program of Shantou University Medical College-Li Ka Shing Foundation, 2017–2020 (SMLYPSC-01), and the National Natural Science Foundation of China (21907063).

## Conflict of Interest

The authors declare that the research was conducted in the absence of any commercial or financial relationships that could be construed as a potential conflict of interest.
