# Supplementary material for: Computational Prediction of Hot Spots and Binding Site of Inhibitor NSC23766 on Rac1 Binding With Tiam1
Source: Front Chem. 2021 Feb 2;8:625437. doi: 10.3389/fchem.2020.625437 (PMC7884829; doi:10.3389/fchem.2020.625437)
Supplement: Supplementary file 1 [file table1.docx]

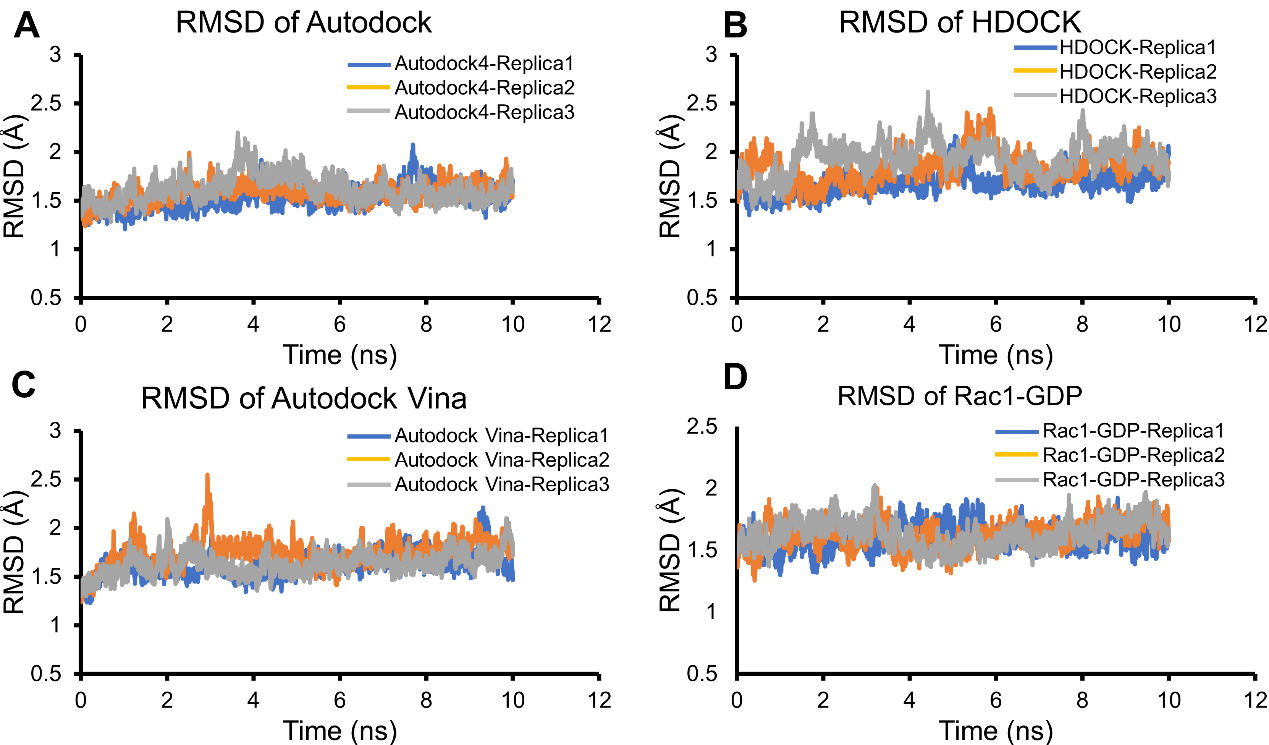


Figure S1. RMSD of different simulated systems. Different colors indicate different replicas.

Rac1-NSC23766 structure from Autodock4

ATOM 1 N GLN A 2 0.578 19.705 12.487 1.00 44.49 N

ATOM 2 CA GLN A 2 -0.825 19.520 12.140 1.00 44.24 C

ATOM 3 C GLN A 2 -1.331 18.147 12.607 1.00 47.85 C

ATOM 4 O GLN A 2 -0.743 17.125 12.243 1.00 48.30 O

ATOM 5 CB GLN A 2 -1.050 19.726 10.627 1.00 45.62 C

ATOM 6 CG GLN A 2 -2.530 19.905 10.242 1.00 69.79 C

ATOM 7 CD GLN A 2 -2.781 20.211 8.776 1.00 97.83 C

ATOM 8 OE1 GLN A 2 -1.863 20.308 7.953 1.00 97.85 O

ATOM 9 NE2 GLN A 2 -4.046 20.386 8.416 1.00 88.23 N

ATOM 10 N ALA A 3 -2.418 18.135 13.426 1.00 42.73 N

ATOM 11 CA ALA A 3 -3.041 16.921 13.975 1.00 40.85 C

ATOM 12 C ALA A 3 -4.173 16.436 13.064 1.00 42.21 C

ATOM 13 O ALA A 3 -5.178 17.138 12.894 1.00 42.92 O

ATOM 14 CB ALA A 3 -3.559 17.173 15.388 1.00 41.27 C

ATOM 15 N ILE A 4 -3.987 15.250 12.455 1.00 34.48 N

ATOM 16 CA ILE A 4 -4.940 14.625 11.535 1.00 32.17 C

ATOM 17 C ILE A 4 -5.801 13.595 12.277 1.00 32.61 C

ATOM 18 O ILE A 4 -5.348 13.017 13.259 1.00 32.48 O

ATOM 19 CB ILE A 4 -4.181 13.991 10.336 1.00 35.14 C

ATOM 20 CG1 ILE A 4 -3.220 14.997 9.673 1.00 35.33 C

ATOM 21 CG2 ILE A 4 -5.136 13.363 9.311 1.00 35.93 C

ATOM 22 CD1 ILE A 4 -2.371 14.399 8.620 1.00 43.68 C

ATOM 23 N LYS A 5 -7.046 13.387 11.815 1.00 26.95 N

ATOM 24 CA LYS A 5 -7.990 12.407 12.359 1.00 25.22 C

ATOM 25 C LYS A 5 -8.423 11.451 11.244 1.00 28.73 C

ATOM 26 O LYS A 5 -9.010 11.866 10.241 1.00 26.42 O

ATOM 27 CB LYS A 5 -9.193 13.082 13.015 1.00 25.75 C

ATOM 28 CG LYS A 5 -10.119 12.094 13.698 1.00 21.04 C

ATOM 29 CD LYS A 5 -11.454 12.699 14.010 1.00 17.37 C

ATOM 30 CE LYS A 5 -12.456 11.661 14.403 1.00 17.54 C

ATOM 31 NZ LYS A 5 -13.690 12.296 14.948 1.00 21.40 N

ATOM 32 N CYS A 6 -8.108 10.165 11.440 1.00 27.17 N

ATOM 33 CA CYS A 6 -8.360 9.088 10.493 1.00 27.18 C

ATOM 34 C CYS A 6 -9.274 8.037 11.084 1.00 28.15 C

ATOM 35 O CYS A 6 -8.880 7.301 11.990 1.00 27.82 O

ATOM 36 CB CYS A 6 -7.037 8.491 10.004 1.00 28.23 C

ATOM 37 SG CYS A 6 -7.200 6.942 9.070 1.00 32.45 S

ATOM 38 N VAL A 7 -10.493 7.956 10.562 1.00 23.30 N

ATOM 39 CA VAL A 7 -11.449 6.951 11.017 1.00 23.01 C

ATOM 40 C VAL A 7 -11.292 5.670 10.177 1.00 25.99 C

ATOM 41 O VAL A 7 -11.189 5.732 8.949 1.00 25.41 O

ATOM 42 CB VAL A 7 -12.914 7.479 11.056 1.00 26.75 C

ATOM 43 CG1 VAL A 7 -13.853 6.458 11.689 1.00 26.39 C

ATOM 44 CG2 VAL A 7 -12.994 8.791 11.823 1.00 26.54 C

ATOM 45 N VAL A 8 -11.251 4.519 10.855 1.00 21.47 N

ATOM 46 CA VAL A 8 -11.146 3.207 10.223 1.00 20.92 C

ATOM 47 C VAL A 8 -12.533 2.538 10.320 1.00 27.79 C

ATOM 48 O VAL A 8 -13.055 2.366 11.426 1.00 28.30 O

ATOM 49 CB VAL A 8 -10.058 2.337 10.885 1.00 23.79 C

ATOM 50 CG1 VAL A 8 -9.807 1.079 10.072 1.00 23.42 C

ATOM 51 CG2 VAL A 8 -8.764 3.116 11.084 1.00 23.60 C

ATOM 52 N VAL A 9 -13.130 2.198 9.165 1.00 24.58 N

ATOM 53 CA VAL A 9 -14.451 1.560 9.048 1.00 24.55 C

ATOM 54 C VAL A 9 -14.353 0.270 8.227 1.00 28.24 C

ATOM 55 O VAL A 9 -13.391 0.102 7.477 1.00 28.46 O

ATOM 56 CB VAL A 9 -15.558 2.517 8.512 1.00 28.43 C

ATOM 57 CG1 VAL A 9 -15.870 3.618 9.520 1.00 28.71 C

ATOM 58 CG2 VAL A 9 -15.198 3.106 7.147 1.00 27.77 C

ATOM 59 N GLY A 10 -15.337 -0.615 8.379 1.00 23.59 N

ATOM 60 CA GLY A 10 -15.372 -1.906 7.700 1.00 23.34 C

ATOM 61 C GLY A 10 -16.065 -2.970 8.518 1.00 28.54 C

ATOM 62 O GLY A 10 -16.413 -2.724 9.678 1.00 29.15 O

ATOM 63 N ASP A 11 -16.300 -4.159 7.919 1.00 25.69 N

ATOM 64 CA ASP A 11 -17.002 -5.279 8.589 1.00 24.89 C

ATOM 65 C ASP A 11 -16.218 -5.798 9.783 1.00 26.12 C

ATOM 66 O ASP A 11 -15.016 -5.609 9.851 1.00 23.37 O

ATOM 67 CB ASP A 11 -17.244 -6.470 7.621 1.00 26.26 C

ATOM 68 CG ASP A 11 -18.279 -6.317 6.507 1.00 33.03 C

ATOM 69 OD1 ASP A 11 -18.784 -5.179 6.301 1.00 32.56 O

ATOM 70 OD2 ASP A 11 -18.573 -7.333 5.829 1.00 33.02 O

ATOM 71 N GLY A 12 -16.911 -6.470 10.701 1.00 24.44 N

ATOM 72 CA GLY A 12 -16.284 -7.158 11.821 1.00 23.83 C

ATOM 73 C GLY A 12 -15.379 -8.252 11.265 1.00 27.31 C

ATOM 74 O GLY A 12 -15.650 -8.799 10.184 1.00 26.80 O

ATOM 75 N ALA A 13 -14.251 -8.505 11.962 1.00 22.12 N

ATOM 76 CA ALA A 13 -13.217 -9.496 11.673 1.00 21.11 C

ATOM 77 C ALA A 13 -12.358 -9.186 10.422 1.00 26.35 C

ATOM 78 O ALA A 13 -11.515 -10.007 10.064 1.00 25.75 O

ATOM 79 CB ALA A 13 -13.810 -10.901 11.604 1.00 21.36 C

ATOM 80 N VAL A 14 -12.498 -7.988 9.802 1.00 24.19 N

ATOM 81 CA VAL A 14 -11.665 -7.613 8.631 1.00 23.61 C

ATOM 82 C VAL A 14 -10.207 -7.383 9.029 1.00 27.91 C

ATOM 83 O VAL A 14 -9.338 -7.352 8.171 1.00 28.47 O

ATOM 84 CB VAL A 14 -12.198 -6.439 7.759 1.00 26.32 C

ATOM 85 CG1 VAL A 14 -13.527 -6.775 7.109 1.00 25.46 C

ATOM 86 CG2 VAL A 14 -12.272 -5.133 8.540 1.00 26.01 C

ATOM 87 N GLY A 15 -9.963 -7.201 10.319 1.00 24.51 N

ATOM 88 CA GLY A 15 -8.623 -7.003 10.845 1.00 24.18 C

ATOM 89 C GLY A 15 -8.262 -5.567 11.141 1.00 26.98 C

ATOM 90 O GLY A 15 -7.068 -5.249 11.184 1.00 26.37 O

ATOM 91 N LYS A 16 -9.292 -4.700 11.346 1.00 22.10 N

ATOM 92 CA LYS A 16 -9.158 -3.267 11.653 1.00 22.43 C

ATOM 93 C LYS A 16 -8.323 -2.986 12.912 1.00 28.18 C

ATOM 94 O LYS A 16 -7.422 -2.151 12.860 1.00 29.82 O

ATOM 95 CB LYS A 16 -10.539 -2.604 11.828 1.00 24.99 C

ATOM 96 CG LYS A 16 -11.284 -2.296 10.533 1.00 29.17 C

ATOM 97 CD LYS A 16 -12.641 -1.584 10.752 1.00 34.50 C

ATOM 98 CE LYS A 16 -13.580 -2.119 11.830 1.00 31.06 C

ATOM 99 NZ LYS A 16 -13.945 -3.549 11.645 1.00 27.95 N

ATOM 100 N THR A 17 -8.635 -3.649 14.037 1.00 23.93 N

ATOM 101 CA THR A 17 -7.923 -3.434 15.297 1.00 24.58 C

ATOM 102 C THR A 17 -6.478 -3.934 15.239 1.00 31.44 C

ATOM 103 O THR A 17 -5.594 -3.302 15.829 1.00 32.16 O

ATOM 104 CB THR A 17 -8.696 -4.031 16.483 1.00 29.50 C

ATOM 105 OG1 THR A 17 -10.100 -3.812 16.317 1.00 26.79 O

ATOM 106 CG2 THR A 17 -8.204 -3.504 17.838 1.00 21.76 C

ATOM 107 N CYS A 18 -6.240 -5.066 14.539 1.00 27.97 N

ATOM 108 CA CYS A 18 -4.898 -5.631 14.360 1.00 27.67 C

ATOM 109 C CYS A 18 -4.049 -4.709 13.502 1.00 29.21 C

ATOM 110 O CYS A 18 -2.856 -4.588 13.742 1.00 30.44 O

ATOM 111 CB CYS A 18 -4.971 -7.026 13.750 1.00 28.42 C

ATOM 112 SG CYS A 18 -5.387 -8.329 14.931 1.00 32.52 S

ATOM 113 N LEU A 19 -4.666 -4.070 12.507 1.00 22.92 N

ATOM 114 CA LEU A 19 -4.032 -3.135 11.586 1.00 22.50 C

ATOM 115 C LEU A 19 -3.485 -1.925 12.386 1.00 28.29 C

ATOM 116 O LEU A 19 -2.300 -1.602 12.289 1.00 28.03 O

ATOM 117 CB LEU A 19 -5.086 -2.719 10.554 1.00 22.11 C

ATOM 118 CG LEU A 19 -4.725 -1.714 9.497 1.00 27.48 C

ATOM 119 CD1 LEU A 19 -3.998 -2.374 8.376 1.00 28.37 C

ATOM 120 CD2 LEU A 19 -5.979 -1.079 8.932 1.00 30.78 C

ATOM 121 N LEU A 20 -4.344 -1.316 13.210 1.00 26.08 N

ATOM 122 CA LEU A 20 -4.022 -0.184 14.079 1.00 26.11 C

ATOM 123 C LEU A 20 -3.001 -0.528 15.168 1.00 29.46 C

ATOM 124 O LEU A 20 -2.127 0.303 15.435 1.00 29.32 O

ATOM 125 CB LEU A 20 -5.303 0.413 14.705 1.00 25.77 C

ATOM 126 CG LEU A 20 -6.288 0.998 13.711 1.00 30.06 C

ATOM 127 CD1 LEU A 20 -7.650 1.098 14.295 1.00 30.72 C

ATOM 128 CD2 LEU A 20 -5.835 2.325 13.216 1.00 32.47 C

ATOM 129 N ILE A 21 -3.097 -1.728 15.799 1.00 25.42 N

ATOM 130 CA ILE A 21 -2.129 -2.114 16.831 1.00 26.03 C

ATOM 131 C ILE A 21 -0.778 -2.405 16.182 1.00 30.67 C

ATOM 132 O ILE A 21 0.210 -1.846 16.634 1.00 31.01 O

ATOM 133 CB ILE A 21 -2.637 -3.227 17.797 1.00 29.74 C

ATOM 134 CG1 ILE A 21 -3.434 -2.601 18.956 1.00 30.30 C

ATOM 135 CG2 ILE A 21 -1.498 -4.078 18.382 1.00 29.92 C

ATOM 136 CD1 ILE A 21 -4.801 -2.447 18.728 1.00 36.19 C

ATOM 137 N SER A 22 -0.744 -3.183 15.078 1.00 26.96 N

ATOM 138 CA SER A 22 0.490 -3.471 14.336 1.00 25.97 C

ATOM 139 C SER A 22 1.229 -2.205 13.971 1.00 28.39 C

ATOM 140 O SER A 22 2.413 -2.104 14.237 1.00 28.68 O

ATOM 141 CB SER A 22 0.202 -4.268 13.068 1.00 29.18 C

ATOM 142 OG SER A 22 -0.279 -5.559 13.393 1.00 41.63 O

ATOM 143 N TYR A 23 0.531 -1.234 13.394 1.00 24.70 N

ATOM 144 CA TYR A 23 1.121 0.019 12.954 1.00 24.64 C

ATOM 145 C TYR A 23 1.744 0.852 14.068 1.00 29.15 C

ATOM 146 O TYR A 23 2.887 1.279 13.931 1.00 29.71 O

ATOM 147 CB TYR A 23 0.102 0.854 12.167 1.00 25.25 C

ATOM 148 CG TYR A 23 0.660 2.199 11.750 1.00 25.31 C

ATOM 149 CD1 TYR A 23 1.692 2.288 10.823 1.00 25.81 C

ATOM 150 CD2 TYR A 23 0.159 3.381 12.288 1.00 26.06 C

ATOM 151 CE1 TYR A 23 2.213 3.518 10.438 1.00 25.69 C

ATOM 152 CE2 TYR A 23 0.690 4.619 11.926 1.00 26.86 C

ATOM 153 CZ TYR A 23 1.722 4.680 11.003 1.00 31.27 C

ATOM 154 OH TYR A 23 2.242 5.885 10.606 1.00 30.44 O

ATOM 155 N THR A 24 0.985 1.101 15.140 1.00 25.56 N

ATOM 156 CA THR A 24 1.389 1.942 16.268 1.00 25.18 C

ATOM 157 C THR A 24 2.380 1.278 17.203 1.00 31.01 C

ATOM 158 O THR A 24 3.267 1.957 17.724 1.00 30.94 O

ATOM 159 CB THR A 24 0.148 2.421 17.047 1.00 25.73 C

ATOM 160 OG1 THR A 24 -0.599 1.282 17.482 1.00 28.62 O

ATOM 161 CG2 THR A 24 -0.740 3.358 16.238 1.00 14.61 C

ATOM 162 N THR A 25 2.211 -0.038 17.432 1.00 29.14 N

ATOM 163 CA THR A 25 2.968 -0.871 18.373 1.00 29.61 C

ATOM 164 C THR A 25 4.183 -1.560 17.717 1.00 36.21 C

ATOM 165 O THR A 25 5.132 -1.914 18.426 1.00 35.88 O

ATOM 166 CB THR A 25 1.950 -1.838 19.038 1.00 35.76 C

ATOM 167 OG1 THR A 25 1.402 -1.224 20.207 1.00 32.34 O

ATOM 168 CG2 THR A 25 2.489 -3.232 19.346 1.00 35.11 C

ATOM 169 N ASN A 26 4.169 -1.711 16.376 1.00 34.41 N

ATOM 170 CA ASN A 26 5.192 -2.409 15.578 1.00 34.83 C

ATOM 171 C ASN A 26 5.116 -3.931 15.777 1.00 38.43 C

ATOM 172 O ASN A 26 6.059 -4.636 15.417 1.00 38.83 O

ATOM 173 CB ASN A 26 6.617 -1.857 15.791 1.00 37.31 C

ATOM 174 CG ASN A 26 6.916 -0.645 14.956 1.00 67.73 C

ATOM 175 OD1 ASN A 26 7.303 -0.754 13.786 1.00 65.29 O

ATOM 176 ND2 ASN A 26 6.717 0.537 15.530 1.00 58.37 N

ATOM 177 N ALA A 27 3.976 -4.441 16.304 1.00 33.93 N

ATOM 178 CA ALA A 27 3.783 -5.871 16.548 1.00 33.76 C

ATOM 179 C ALA A 27 2.376 -6.345 16.265 1.00 36.71 C

ATOM 180 O ALA A 27 1.417 -5.613 16.512 1.00 36.29 O

ATOM 181 CB ALA A 27 4.167 -6.225 17.981 1.00 34.57 C

ATOM 182 N PHE A 28 2.249 -7.590 15.772 1.00 32.60 N

ATOM 183 CA PHE A 28 0.946 -8.197 15.504 1.00 32.11 C

ATOM 184 C PHE A 28 0.346 -8.754 16.818 1.00 37.57 C

ATOM 185 O PHE A 28 0.974 -9.614 17.440 1.00 37.87 O

ATOM 186 CB PHE A 28 1.046 -9.273 14.411 1.00 33.18 C

ATOM 187 CG PHE A 28 -0.300 -9.804 13.978 1.00 34.44 C

ATOM 188 CD1 PHE A 28 -1.181 -9.008 13.252 1.00 36.14 C

ATOM 189 CD2 PHE A 28 -0.699 -11.095 14.316 1.00 36.17 C

ATOM 190 CE1 PHE A 28 -2.424 -9.499 12.856 1.00 36.83 C

ATOM 191 CE2 PHE A 28 -1.951 -11.580 13.927 1.00 38.37 C

ATOM 192 CZ PHE A 28 -2.804 -10.779 13.201 1.00 36.12 C

ATOM 193 N PRO A 29 -0.854 -8.291 17.256 1.00 34.63 N

ATOM 194 CA PRO A 29 -1.413 -8.757 18.543 1.00 35.50 C

ATOM 195 C PRO A 29 -1.809 -10.237 18.662 1.00 43.19 C

ATOM 196 O PRO A 29 -2.305 -10.634 19.714 1.00 41.85 O

ATOM 197 CB PRO A 29 -2.632 -7.852 18.753 1.00 36.80 C

ATOM 198 CG PRO A 29 -2.985 -7.383 17.430 1.00 40.85 C

ATOM 199 CD PRO A 29 -1.712 -7.256 16.657 1.00 36.30 C

ATOM 200 N GLY A 30 -1.578 -11.027 17.616 1.00 44.10 N

ATOM 201 CA GLY A 30 -1.886 -12.452 17.597 1.00 46.09 C

ATOM 202 C GLY A 30 -3.322 -12.797 17.942 1.00 55.43 C

ATOM 203 O GLY A 30 -4.256 -12.302 17.297 1.00 55.23 O

ATOM 204 N GLU A 31 -3.499 -13.645 18.982 1.00 55.41 N

ATOM 205 CA GLU A 31 -4.817 -14.106 19.439 1.00 56.45 C

ATOM 206 C GLU A 31 -5.517 -13.080 20.338 1.00 60.26 C

ATOM 207 O GLU A 31 -6.657 -12.710 20.026 1.00 60.91 O

ATOM 208 CB GLU A 31 -4.774 -15.514 20.083 1.00 58.37 C

ATOM 209 CG GLU A 31 -3.807 -15.682 21.252 1.00 74.12 C

ATOM 210 CD GLU A 31 -4.047 -16.858 22.184 1.00103.99 C

ATOM 211 OE1 GLU A 31 -5.113 -17.509 22.083 1.00100.46 O

ATOM 212 OE2 GLU A 31 -3.171 -17.106 23.044 1.00103.64 O

ATOM 213 N TYR A 32 -4.857 -12.598 21.426 1.00 54.97 N

ATOM 214 CA TYR A 32 -5.517 -11.594 22.262 1.00 53.78 C

ATOM 215 C TYR A 32 -5.366 -10.188 21.694 1.00 49.03 C

ATOM 216 O TYR A 32 -4.346 -9.526 21.893 1.00 47.38 O

ATOM 217 CB TYR A 32 -5.164 -11.637 23.771 1.00 56.98 C

ATOM 218 CG TYR A 32 -6.023 -10.655 24.549 1.00 61.10 C

ATOM 219 CD1 TYR A 32 -7.368 -10.920 24.795 1.00 62.90 C

ATOM 220 CD2 TYR A 32 -5.537 -9.394 24.894 1.00 63.34 C

ATOM 221 CE1 TYR A 32 -8.191 -9.980 25.417 1.00 64.48 C

ATOM 222 CE2 TYR A 32 -6.355 -8.441 25.502 1.00 64.18 C

ATOM 223 CZ TYR A 32 -7.679 -8.741 25.768 1.00 72.55 C

ATOM 224 OH TYR A 32 -8.480 -7.814 26.389 1.00 75.10 O

ATOM 225 N ILE A 33 -6.432 -9.743 21.021 1.00 40.85 N

ATOM 226 CA ILE A 33 -6.607 -8.421 20.424 1.00 38.10 C

ATOM 227 C ILE A 33 -7.422 -7.615 21.447 1.00 38.70 C

ATOM 228 O ILE A 33 -8.402 -8.159 21.960 1.00 39.15 O

ATOM 229 CB ILE A 33 -7.379 -8.561 19.077 1.00 40.38 C

ATOM 230 CG1 ILE A 33 -6.716 -9.592 18.139 1.00 41.11 C

ATOM 231 CG2 ILE A 33 -7.563 -7.224 18.370 1.00 39.62 C

ATOM 232 CD1 ILE A 33 -7.680 -10.614 17.539 1.00 45.52 C

ATOM 233 N PRO A 34 -7.073 -6.343 21.777 1.00 32.45 N

ATOM 234 CA PRO A 34 -7.923 -5.578 22.715 1.00 30.68 C

ATOM 235 C PRO A 34 -9.373 -5.517 22.231 1.00 29.98 C

ATOM 236 O PRO A 34 -9.641 -5.416 21.031 1.00 26.43 O

ATOM 237 CB PRO A 34 -7.282 -4.182 22.742 1.00 32.59 C

ATOM 238 CG PRO A 34 -6.419 -4.117 21.529 1.00 37.45 C

ATOM 239 CD PRO A 34 -5.949 -5.519 21.278 1.00 33.39 C

ATOM 240 N THR A 35 -10.307 -5.625 23.174 1.00 27.16 N

ATOM 241 CA THR A 35 -11.735 -5.654 22.857 1.00 26.36 C

ATOM 242 C THR A 35 -12.388 -4.271 23.026 1.00 28.68 C

ATOM 243 O THR A 35 -13.414 -4.010 22.410 1.00 28.20 O

ATOM 244 CB THR A 35 -12.411 -6.772 23.637 1.00 28.95 C

ATOM 245 OG1 THR A 35 -12.150 -6.562 25.026 1.00 35.74 O

ATOM 246 CG2 THR A 35 -11.901 -8.151 23.221 1.00 17.78 C

ATOM 247 N VAL A 36 -11.760 -3.375 23.795 1.00 25.06 N

ATOM 248 CA VAL A 36 -12.232 -1.996 23.974 1.00 24.60 C

ATOM 249 C VAL A 36 -11.975 -1.234 22.668 1.00 27.70 C

ATOM 250 O VAL A 36 -10.835 -1.230 22.178 1.00 28.23 O

ATOM 251 CB VAL A 36 -11.548 -1.303 25.194 1.00 28.07 C

ATOM 252 CG1 VAL A 36 -11.923 0.180 25.284 1.00 27.61 C

ATOM 253 CG2 VAL A 36 -11.888 -2.027 26.498 1.00 27.75 C

ATOM 254 N PHE A 37 -13.023 -0.620 22.086 1.00 22.24 N

ATOM 255 CA PHE A 37 -12.844 0.148 20.852 1.00 21.90 C

ATOM 256 C PHE A 37 -12.183 1.460 21.232 1.00 28.40 C

ATOM 257 O PHE A 37 -12.756 2.263 21.964 1.00 29.62 O

ATOM 258 CB PHE A 37 -14.155 0.356 20.085 1.00 23.06 C

ATOM 259 CG PHE A 37 -14.915 -0.909 19.760 1.00 23.76 C

ATOM 260 CD1 PHE A 37 -14.669 -1.606 18.576 1.00 25.40 C

ATOM 261 CD2 PHE A 37 -15.901 -1.392 20.623 1.00 23.37 C

ATOM 262 CE1 PHE A 37 -15.387 -2.770 18.270 1.00 24.84 C

ATOM 263 CE2 PHE A 37 -16.622 -2.544 20.308 1.00 25.14 C

ATOM 264 CZ PHE A 37 -16.363 -3.220 19.131 1.00 23.24 C

ATOM 265 N ASP A 38 -10.941 1.624 20.830 1.00 25.05 N

ATOM 266 CA ASP A 38 -10.191 2.781 21.232 1.00 25.67 C

ATOM 267 C ASP A 38 -9.717 3.603 20.051 1.00 29.81 C

ATOM 268 O ASP A 38 -10.070 3.326 18.907 1.00 29.42 O

ATOM 269 CB ASP A 38 -9.002 2.314 22.104 1.00 28.51 C

ATOM 270 CG ASP A 38 -8.596 3.254 23.238 1.00 45.62 C

ATOM 271 OD1 ASP A 38 -9.012 4.441 23.214 1.00 46.49 O

ATOM 272 OD2 ASP A 38 -7.824 2.818 24.119 1.00 54.24 O

ATOM 273 N ASN A 39 -8.966 4.663 20.357 1.00 26.75 N

ATOM 274 CA ASN A 39 -8.305 5.552 19.418 1.00 26.03 C

ATOM 275 C ASN A 39 -6.831 5.464 19.707 1.00 26.87 C

ATOM 276 O ASN A 39 -6.425 5.254 20.842 1.00 24.48 O

ATOM 277 CB ASN A 39 -8.800 6.999 19.526 1.00 29.94 C

ATOM 278 CG ASN A 39 -8.790 7.600 20.915 1.00 57.35 C

ATOM 279 OD1 ASN A 39 -9.794 7.567 21.638 1.00 50.08 O

ATOM 280 ND2 ASN A 39 -7.668 8.208 21.299 1.00 49.62 N

ATOM 281 N TYR A 40 -6.036 5.612 18.682 1.00 24.82 N

ATOM 282 CA TYR A 40 -4.586 5.507 18.758 1.00 24.20 C

ATOM 283 C TYR A 40 -3.947 6.764 18.173 1.00 27.69 C

ATOM 284 O TYR A 40 -4.649 7.622 17.641 1.00 25.92 O

ATOM 285 CB TYR A 40 -4.126 4.220 18.046 1.00 24.74 C

ATOM 286 CG TYR A 40 -4.869 2.992 18.530 1.00 26.90 C

ATOM 287 CD1 TYR A 40 -4.534 2.380 19.737 1.00 28.84 C

ATOM 288 CD2 TYR A 40 -5.968 2.497 17.832 1.00 27.70 C

ATOM 289 CE1 TYR A 40 -5.228 1.264 20.200 1.00 29.79 C

ATOM 290 CE2 TYR A 40 -6.673 1.382 18.287 1.00 28.65 C

ATOM 291 CZ TYR A 40 -6.295 0.764 19.468 1.00 35.12 C

ATOM 292 OH TYR A 40 -6.985 -0.335 19.918 1.00 32.27 O

ATOM 293 N SER A 41 -2.631 6.888 18.313 1.00 25.66 N

ATOM 294 CA SER A 41 -1.900 8.057 17.855 1.00 25.99 C

ATOM 295 C SER A 41 -0.550 7.663 17.305 1.00 29.48 C

ATOM 296 O SER A 41 0.046 6.699 17.775 1.00 28.66 O

ATOM 297 CB SER A 41 -1.715 9.039 19.014 1.00 29.27 C

ATOM 298 OG SER A 41 -1.047 10.224 18.615 1.00 35.80 O

ATOM 299 N ALA A 42 -0.058 8.432 16.338 1.00 27.46 N

ATOM 300 CA ALA A 42 1.277 8.256 15.782 1.00 29.18 C

ATOM 301 C ALA A 42 1.875 9.613 15.359 1.00 37.66 C

ATOM 302 O ALA A 42 1.262 10.338 14.568 1.00 37.59 O

ATOM 303 CB ALA A 42 1.254 7.280 14.615 1.00 29.84 C

ATOM 304 N ASN A 43 3.040 9.972 15.931 1.00 37.14 N

ATOM 305 CA ASN A 43 3.778 11.188 15.569 1.00 38.38 C

ATOM 306 C ASN A 43 4.694 10.793 14.428 1.00 47.90 C

ATOM 307 O ASN A 43 5.680 10.075 14.634 1.00 47.49 O

ATOM 308 CB ASN A 43 4.594 11.723 16.741 1.00 36.60 C

ATOM 309 CG ASN A 43 3.758 12.302 17.840 1.00 58.04 C

ATOM 310 OD1 ASN A 43 2.948 13.210 17.630 1.00 54.58 O

ATOM 311 ND2 ASN A 43 3.995 11.840 19.053 1.00 48.76 N

ATOM 312 N VAL A 44 4.324 11.185 13.212 1.00 49.18 N

ATOM 313 CA VAL A 44 5.079 10.826 12.018 1.00 51.23 C

ATOM 314 C VAL A 44 5.333 12.015 11.119 1.00 59.56 C

ATOM 315 O VAL A 44 4.470 12.875 10.978 1.00 59.24 O

ATOM 316 CB VAL A 44 4.444 9.638 11.245 1.00 55.69 C

ATOM 317 CG1 VAL A 44 4.464 8.368 12.089 1.00 56.00 C

ATOM 318 CG2 VAL A 44 3.023 9.955 10.784 1.00 55.38 C

ATOM 319 N MET A 45 6.523 12.069 10.515 1.00 59.88 N

ATOM 320 CA MET A 45 6.878 13.139 9.588 1.00 61.29 C

ATOM 321 C MET A 45 7.274 12.568 8.238 1.00 68.13 C

ATOM 322 O MET A 45 8.344 11.978 8.107 1.00 68.02 O

ATOM 323 CB MET A 45 7.928 14.105 10.170 1.00 63.87 C

ATOM 324 CG MET A 45 9.304 13.501 10.365 0.00 67.86 C

ATOM 325 SD MET A 45 10.338 14.574 11.386 0.00 72.65 S

ATOM 326 CE MET A 45 9.908 13.980 13.001 0.00 69.33 C

ATOM 327 N VAL A 46 6.363 12.668 7.253 1.00 67.17 N

ATOM 328 CA VAL A 46 6.588 12.178 5.889 1.00 67.70 C

ATOM 329 C VAL A 46 6.893 13.386 4.975 1.00 73.00 C

ATOM 330 O VAL A 46 5.986 14.134 4.594 1.00 73.06 O

ATOM 331 CB VAL A 46 5.481 11.207 5.365 0.00 71.66 C

ATOM 332 CG1 VAL A 46 4.078 11.817 5.432 0.00 71.48 C

ATOM 333 CG2 VAL A 46 5.800 10.689 3.966 0.00 71.49 C

ATOM 334 N ASP A 47 8.206 13.588 4.693 1.00 69.57 N

ATOM 335 CA ASP A 47 8.820 14.672 3.906 1.00 69.18 C

ATOM 336 C ASP A 47 8.263 16.072 4.284 1.00 71.81 C

ATOM 337 O ASP A 47 7.685 16.785 3.453 1.00 71.42 O

ATOM 338 CB ASP A 47 8.796 14.410 2.380 0.00 70.98 C

ATOM 339 CG ASP A 47 7.461 14.018 1.779 0.00 80.64 C

ATOM 340 OD1 ASP A 47 6.644 14.923 1.510 0.00 81.07 O

ATOM 341 OD2 ASP A 47 7.252 12.811 1.539 0.00 86.46 O

ATOM 342 N GLY A 48 8.459 16.431 5.552 1.00 66.66 N

ATOM 343 CA GLY A 48 8.025 17.705 6.104 1.00 65.73 C

ATOM 344 C GLY A 48 8.401 17.864 7.557 1.00 68.36 C

ATOM 345 O GLY A 48 9.527 17.548 7.955 1.00 67.75 O

ATOM 346 N LYS A 49 7.449 18.371 8.352 1.00 63.92 N

ATOM 347 CA LYS A 49 7.578 18.594 9.795 1.00 62.76 C

ATOM 348 C LYS A 49 6.695 17.570 10.558 1.00 63.67 C

ATOM 349 O LYS A 49 5.936 16.854 9.890 1.00 62.95 O

ATOM 350 CB LYS A 49 7.218 20.054 10.137 0.00 65.36 C

ATOM 351 CG LYS A 49 8.377 21.018 9.908 0.00 80.31 C

ATOM 352 CD LYS A 49 8.099 22.404 10.462 0.00 90.85 C

ATOM 353 CE LYS A 49 9.290 23.315 10.295 0.00102.35 C

ATOM 354 NZ LYS A 49 9.033 24.669 10.853 0.00111.76 N

ATOM 355 N PRO A 50 6.796 17.424 11.914 1.00 58.23 N

ATOM 356 CA PRO A 50 5.971 16.404 12.604 1.00 57.01 C

ATOM 357 C PRO A 50 4.458 16.556 12.439 1.00 56.67 C

ATOM 358 O PRO A 50 3.924 17.673 12.403 1.00 56.49 O

ATOM 359 CB PRO A 50 6.392 16.516 14.077 1.00 58.99 C

ATOM 360 CG PRO A 50 7.702 17.223 14.060 1.00 63.99 C

ATOM 361 CD PRO A 50 7.664 18.141 12.875 1.00 59.63 C

ATOM 362 N VAL A 51 3.777 15.415 12.316 1.00 48.75 N

ATOM 363 CA VAL A 51 2.329 15.343 12.160 1.00 46.75 C

ATOM 364 C VAL A 51 1.804 14.286 13.138 1.00 47.14 C

ATOM 365 O VAL A 51 2.350 13.185 13.189 1.00 46.48 O

ATOM 366 CB VAL A 51 1.945 15.026 10.684 1.00 50.53 C

ATOM 367 CG1 VAL A 51 0.535 14.464 10.565 1.00 50.05 C

ATOM 368 CG2 VAL A 51 2.124 16.244 9.783 1.00 50.47 C

ATOM 369 N ASN A 52 0.765 14.625 13.922 1.00 41.56 N

ATOM 370 CA ASN A 52 0.142 13.676 14.837 1.00 40.45 C

ATOM 371 C ASN A 52 -1.114 13.082 14.193 1.00 42.66 C

ATOM 372 O ASN A 52 -2.078 13.796 13.900 1.00 41.81 O

ATOM 373 CB ASN A 52 -0.165 14.292 16.200 1.00 39.87 C

ATOM 374 CG ASN A 52 -0.652 13.262 17.178 1.00 57.82 C

ATOM 375 OD1 ASN A 52 -1.852 13.112 17.397 1.00 51.52 O

ATOM 376 ND2 ASN A 52 0.260 12.451 17.692 1.00 49.50 N

ATOM 377 N LEU A 53 -1.080 11.774 13.957 1.00 38.02 N

ATOM 378 CA LEU A 53 -2.176 11.056 13.331 1.00 37.13 C

ATOM 379 C LEU A 53 -3.036 10.322 14.364 1.00 37.79 C

ATOM 380 O LEU A 53 -2.641 9.278 14.881 1.00 38.40 O

ATOM 381 CB LEU A 53 -1.621 10.098 12.244 1.00 37.47 C

ATOM 382 CG LEU A 53 -2.608 9.242 11.424 1.00 42.60 C

ATOM 383 CD1 LEU A 53 -3.575 10.104 10.603 1.00 43.04 C

ATOM 384 CD2 LEU A 53 -1.860 8.315 10.491 1.00 45.63 C

ATOM 385 N GLY A 54 -4.199 10.888 14.647 1.00 31.04 N

ATOM 386 CA GLY A 54 -5.206 10.295 15.515 1.00 29.60 C

ATOM 387 C GLY A 54 -5.941 9.234 14.720 1.00 31.25 C

ATOM 388 O GLY A 54 -6.505 9.528 13.662 1.00 30.50 O

ATOM 389 N LEU A 55 -5.866 7.980 15.176 1.00 27.11 N

ATOM 390 CA LEU A 55 -6.452 6.820 14.494 1.00 26.24 C

ATOM 391 C LEU A 55 -7.630 6.328 15.269 1.00 30.07 C

ATOM 392 O LEU A 55 -7.501 6.036 16.448 1.00 29.12 O

ATOM 393 CB LEU A 55 -5.401 5.714 14.348 1.00 26.00 C

ATOM 394 CG LEU A 55 -4.180 6.100 13.521 1.00 30.15 C

ATOM 395 CD1 LEU A 55 -2.931 5.446 14.025 1.00 29.85 C

ATOM 396 CD2 LEU A 55 -4.408 5.851 12.079 1.00 32.07 C

ATOM 397 N TRP A 56 -8.793 6.275 14.631 1.00 28.37 N

ATOM 398 CA TRP A 56 -10.020 5.880 15.310 1.00 28.59 C

ATOM 399 C TRP A 56 -10.575 4.562 14.831 1.00 29.62 C

ATOM 400 O TRP A 56 -10.865 4.403 13.652 1.00 29.63 O

ATOM 401 CB TRP A 56 -11.054 7.007 15.217 1.00 28.43 C

ATOM 402 CG TRP A 56 -10.617 8.238 15.956 1.00 30.47 C

ATOM 403 CD1 TRP A 56 -9.716 9.174 15.538 1.00 33.49 C

ATOM 404 CD2 TRP A 56 -11.007 8.619 17.278 1.00 31.02 C

ATOM 405 NE1 TRP A 56 -9.528 10.119 16.516 1.00 33.29 N

ATOM 406 CE2 TRP A 56 -10.321 9.812 17.591 1.00 35.17 C

ATOM 407 CE3 TRP A 56 -11.880 8.066 18.236 1.00 33.00 C

ATOM 408 CZ2 TRP A 56 -10.464 10.454 18.828 1.00 35.15 C

ATOM 409 CZ3 TRP A 56 -12.026 8.707 19.459 1.00 34.67 C

ATOM 410 CH2 TRP A 56 -11.329 9.889 19.743 1.00 35.32 C

ATOM 411 N ASP A 57 -10.675 3.601 15.741 1.00 25.15 N

ATOM 412 CA ASP A 57 -11.258 2.304 15.444 1.00 25.01 C

ATOM 413 C ASP A 57 -12.745 2.374 15.685 1.00 29.61 C

ATOM 414 O ASP A 57 -13.219 3.148 16.519 1.00 29.79 O

ATOM 415 CB ASP A 57 -10.671 1.209 16.329 1.00 27.25 C

ATOM 416 CG ASP A 57 -10.842 -0.185 15.756 1.00 34.91 C

ATOM 417 OD1 ASP A 57 -11.251 -0.297 14.573 1.00 35.37 O

ATOM 418 OD2 ASP A 57 -10.562 -1.165 16.486 1.00 34.97 O

ATOM 419 N THR A 58 -13.489 1.562 14.952 1.00 26.55 N

ATOM 420 CA THR A 58 -14.932 1.525 15.070 1.00 25.50 C

ATOM 421 C THR A 58 -15.424 0.125 15.317 1.00 30.05 C

ATOM 422 O THR A 58 -14.744 -0.875 15.051 1.00 28.27 O

ATOM 423 CB THR A 58 -15.615 2.084 13.811 1.00 29.70 C

ATOM 424 OG1 THR A 58 -15.179 1.336 12.682 1.00 30.49 O

ATOM 425 CG2 THR A 58 -15.378 3.578 13.604 1.00 27.63 C

ATOM 426 N ALA A 59 -16.648 0.082 15.819 1.00 28.68 N

ATOM 427 CA ALA A 59 -17.432 -1.094 16.072 1.00 28.17 C

ATOM 428 C ALA A 59 -18.210 -1.297 14.753 1.00 30.69 C

ATOM 429 O ALA A 59 -19.104 -0.511 14.418 1.00 29.73 O

ATOM 430 CB ALA A 59 -18.349 -0.825 17.253 1.00 28.79 C

ATOM 431 N GLY A 60 -17.749 -2.277 13.974 1.00 27.40 N

ATOM 432 CA GLY A 60 -18.240 -2.591 12.634 1.00 27.50 C

ATOM 433 C GLY A 60 -19.541 -3.352 12.500 1.00 32.27 C

ATOM 434 O GLY A 60 -20.196 -3.241 11.457 1.00 31.63 O

ATOM 435 N GLN A 61 -19.927 -4.135 13.541 1.00 30.49 N

ATOM 436 CA GLN A 61 -21.155 -4.956 13.574 1.00 31.37 C

ATOM 437 C GLN A 61 -22.465 -4.135 13.327 1.00 37.06 C

ATOM 438 O GLN A 61 -22.484 -2.921 13.541 1.00 36.06 O

ATOM 439 CB GLN A 61 -21.225 -5.773 14.885 1.00 32.61 C

ATOM 440 CG GLN A 61 -22.185 -6.969 14.881 1.00 58.97 C

ATOM 441 CD GLN A 61 -22.572 -7.459 16.267 1.00 81.26 C

ATOM 442 OE1 GLN A 61 -22.734 -6.685 17.219 1.00 79.42 O

ATOM 443 NE2 GLN A 61 -22.797 -8.760 16.396 1.00 70.42 N

ATOM 444 N GLU A 62 -23.537 -4.818 12.854 1.00 34.87 N

ATOM 445 CA GLU A 62 -24.870 -4.269 12.565 1.00 35.31 C

ATOM 446 C GLU A 62 -25.474 -3.532 13.750 1.00 37.73 C

ATOM 447 O GLU A 62 -26.170 -2.547 13.546 1.00 35.98 O

ATOM 448 CB GLU A 62 -25.828 -5.404 12.161 1.00 37.47 C

ATOM 449 CG GLU A 62 -26.116 -5.487 10.671 1.00 53.69 C

ATOM 450 CD GLU A 62 -26.849 -6.749 10.249 1.00 79.35 C

ATOM 451 OE1 GLU A 62 -26.223 -7.597 9.573 1.00 73.01 O

ATOM 452 OE2 GLU A 62 -28.042 -6.898 10.602 1.00 73.70 O

ATOM 453 N ASP A 63 -25.223 -4.020 14.980 1.00 35.85 N

ATOM 454 CA ASP A 63 -25.732 -3.448 16.233 1.00 36.33 C

ATOM 455 C ASP A 63 -25.321 -1.988 16.507 1.00 40.73 C

ATOM 456 O ASP A 63 -26.037 -1.271 17.222 1.00 39.08 O

ATOM 457 CB ASP A 63 -25.340 -4.339 17.422 1.00 38.02 C

ATOM 458 CG ASP A 63 -26.049 -5.679 17.472 1.00 48.11 C

ATOM 459 OD1 ASP A 63 -26.965 -5.901 16.644 1.00 47.44 O

ATOM 460 OD2 ASP A 63 -25.711 -6.498 18.361 1.00 55.19 O

ATOM 461 N TYR A 64 -24.182 -1.558 15.936 1.00 38.48 N

ATOM 462 CA TYR A 64 -23.634 -0.219 16.123 1.00 39.30 C

ATOM 463 C TYR A 64 -23.913 0.731 14.976 1.00 42.25 C

ATOM 464 O TYR A 64 -23.342 1.823 14.966 1.00 42.07 O

ATOM 465 CB TYR A 64 -22.131 -0.279 16.425 1.00 42.26 C

ATOM 466 CG TYR A 64 -21.737 -1.528 17.171 1.00 47.23 C

ATOM 467 CD1 TYR A 64 -22.080 -1.702 18.507 1.00 50.17 C

ATOM 468 CD2 TYR A 64 -21.072 -2.567 16.527 1.00 48.85 C

ATOM 469 CE1 TYR A 64 -21.787 -2.888 19.177 1.00 53.14 C

ATOM 470 CE2 TYR A 64 -20.700 -3.721 17.212 1.00 50.26 C

ATOM 471 CZ TYR A 64 -21.075 -3.887 18.533 1.00 62.03 C

ATOM 472 OH TYR A 64 -20.740 -5.045 19.193 1.00 68.84 O

ATOM 473 N ASP A 65 -24.825 0.360 14.047 1.00 38.19 N

ATOM 474 CA ASP A 65 -25.179 1.180 12.882 1.00 38.32 C

ATOM 475 C ASP A 65 -25.641 2.613 13.230 1.00 41.59 C

ATOM 476 O ASP A 65 -25.360 3.539 12.464 1.00 42.07 O

ATOM 477 CB ASP A 65 -26.214 0.472 11.987 1.00 40.71 C

ATOM 478 CG ASP A 65 -25.664 -0.649 11.103 1.00 57.81 C

ATOM 479 OD1 ASP A 65 -24.524 -1.100 11.350 1.00 60.63 O

ATOM 480 OD2 ASP A 65 -26.402 -1.117 10.203 1.00 63.18 O

ATOM 481 N ARG A 66 -26.313 2.799 14.380 1.00 36.43 N

ATOM 482 CA ARG A 66 -26.810 4.114 14.814 1.00 35.58 C

ATOM 483 C ARG A 66 -25.774 4.926 15.608 1.00 36.57 C

ATOM 484 O ARG A 66 -25.769 6.155 15.529 1.00 35.51 O

ATOM 485 CB ARG A 66 -28.106 3.968 15.641 1.00 36.63 C

ATOM 486 CG ARG A 66 -29.273 3.290 14.927 1.00 52.49 C

ATOM 487 CD ARG A 66 -30.132 4.277 14.164 1.00 75.08 C

ATOM 488 NE ARG A 66 -31.173 3.608 13.379 1.00 97.82 N

ATOM 489 CZ ARG A 66 -31.007 3.141 12.143 1.00120.30 C

ATOM 490 NH1 ARG A 66 -29.828 3.244 11.537 1.00109.65 N

ATOM 491 NH2 ARG A 66 -32.015 2.558 11.507 1.00109.91 N

ATOM 492 N LEU A 67 -24.901 4.246 16.364 1.00 31.29 N

ATOM 493 CA LEU A 67 -23.926 4.904 17.225 1.00 30.32 C

ATOM 494 C LEU A 67 -22.566 5.136 16.603 1.00 35.30 C

ATOM 495 O LEU A 67 -21.854 6.035 17.055 1.00 36.19 O

ATOM 496 CB LEU A 67 -23.776 4.143 18.554 1.00 29.99 C

ATOM 497 CG LEU A 67 -25.040 3.986 19.421 1.00 33.19 C

ATOM 498 CD1 LEU A 67 -24.742 3.180 20.625 1.00 32.69 C

ATOM 499 CD2 LEU A 67 -25.615 5.325 19.828 1.00 33.13 C

ATOM 500 N ARG A 68 -22.174 4.310 15.618 1.00 31.05 N

ATOM 501 CA ARG A 68 -20.888 4.407 14.914 1.00 29.84 C

ATOM 502 C ARG A 68 -20.669 5.804 14.276 1.00 34.12 C

ATOM 503 O ARG A 68 -19.603 6.378 14.518 1.00 35.65 O

ATOM 504 CB ARG A 68 -20.774 3.297 13.859 1.00 26.58 C

ATOM 505 CG ARG A 68 -19.436 3.169 13.159 1.00 26.21 C

ATOM 506 CD ARG A 68 -19.544 2.205 11.990 1.00 32.60 C

ATOM 507 NE ARG A 68 -20.246 0.965 12.344 1.00 30.78 N

ATOM 508 CZ ARG A 68 -21.341 0.519 11.738 1.00 42.91 C

ATOM 509 NH1 ARG A 68 -21.868 1.192 10.717 1.00 17.13 N

ATOM 510 NH2 ARG A 68 -21.913 -0.608 12.137 1.00 32.42 N

ATOM 511 N PRO A 69 -21.634 6.408 13.521 1.00 28.32 N

ATOM 512 CA PRO A 69 -21.361 7.715 12.913 1.00 27.52 C

ATOM 513 C PRO A 69 -21.123 8.869 13.890 1.00 30.97 C

ATOM 514 O PRO A 69 -20.705 9.937 13.440 1.00 31.25 O

ATOM 515 CB PRO A 69 -22.578 7.940 12.016 1.00 28.88 C

ATOM 516 CG PRO A 69 -23.079 6.567 11.729 1.00 33.09 C

ATOM 517 CD PRO A 69 -22.946 5.911 13.059 1.00 29.19 C

ATOM 518 N LEU A 70 -21.311 8.644 15.217 1.00 25.58 N

ATOM 519 CA LEU A 70 -21.053 9.666 16.234 1.00 24.52 C

ATOM 520 C LEU A 70 -19.550 10.010 16.349 1.00 28.73 C

ATOM 521 O LEU A 70 -19.207 11.123 16.781 1.00 27.61 O

ATOM 522 CB LEU A 70 -21.633 9.272 17.601 1.00 24.04 C

ATOM 523 CG LEU A 70 -23.151 9.241 17.711 1.00 26.95 C

ATOM 524 CD1 LEU A 70 -23.573 8.655 19.030 1.00 26.34 C

ATOM 525 CD2 LEU A 70 -23.751 10.616 17.498 1.00 27.42 C

ATOM 526 N SER A 71 -18.664 9.059 15.927 1.00 24.69 N

ATOM 527 CA SER A 71 -17.206 9.231 15.911 1.00 24.38 C

ATOM 528 C SER A 71 -16.716 9.898 14.612 1.00 30.13 C

ATOM 529 O SER A 71 -15.572 10.348 14.551 1.00 29.15 O

ATOM 530 CB SER A 71 -16.490 7.902 16.140 1.00 28.26 C

ATOM 531 OG SER A 71 -17.143 6.791 15.544 1.00 43.46 O

ATOM 532 N TYR A 72 -17.587 9.980 13.585 1.00 27.84 N

ATOM 533 CA TYR A 72 -17.245 10.576 12.294 1.00 27.71 C

ATOM 534 C TYR A 72 -17.009 12.102 12.344 1.00 33.95 C

ATOM 535 O TYR A 72 -16.028 12.528 11.746 1.00 34.53 O

ATOM 536 CB TYR A 72 -18.267 10.222 11.190 1.00 28.03 C

ATOM 537 CG TYR A 72 -18.449 8.747 10.861 1.00 28.61 C

ATOM 538 CD1 TYR A 72 -17.593 7.775 11.382 1.00 30.14 C

ATOM 539 CD2 TYR A 72 -19.461 8.328 10.006 1.00 28.89 C

ATOM 540 CE1 TYR A 72 -17.770 6.423 11.089 1.00 30.83 C

ATOM 541 CE2 TYR A 72 -19.643 6.983 9.700 1.00 29.38 C

ATOM 542 CZ TYR A 72 -18.797 6.033 10.240 1.00 36.04 C

ATOM 543 OH TYR A 72 -19.010 4.713 9.919 1.00 33.72 O

ATOM 544 N PRO A 73 -17.809 12.957 13.034 1.00 30.64 N

ATOM 545 CA PRO A 73 -17.529 14.407 12.992 1.00 30.50 C

ATOM 546 C PRO A 73 -16.085 14.827 13.270 1.00 36.43 C

ATOM 547 O PRO A 73 -15.447 14.276 14.167 1.00 35.73 O

ATOM 548 CB PRO A 73 -18.499 14.969 14.021 1.00 31.86 C

ATOM 549 CG PRO A 73 -19.664 14.053 13.917 1.00 36.20 C

ATOM 550 CD PRO A 73 -19.047 12.695 13.799 1.00 31.71 C

ATOM 551 N GLN A 74 -15.576 15.801 12.461 1.00 35.22 N

ATOM 552 CA GLN A 74 -14.221 16.397 12.486 1.00 35.46 C

ATOM 553 C GLN A 74 -13.150 15.505 11.823 1.00 38.44 C

ATOM 554 O GLN A 74 -11.957 15.822 11.897 1.00 38.24 O

ATOM 555 CB GLN A 74 -13.784 16.826 13.912 1.00 37.51 C

ATOM 556 CG GLN A 74 -14.682 17.875 14.578 1.00 62.26 C

ATOM 557 CD GLN A 74 -14.362 18.043 16.048 1.00 88.25 C

ATOM 558 OE1 GLN A 74 -14.514 17.118 16.857 1.00 85.61 O

ATOM 559 NE2 GLN A 74 -13.940 19.239 16.437 1.00 81.51 N

ATOM 560 N THR A 75 -13.569 14.402 11.170 1.00 34.52 N

ATOM 561 CA THR A 75 -12.645 13.464 10.519 1.00 34.20 C

ATOM 562 C THR A 75 -11.996 14.085 9.278 1.00 37.35 C

ATOM 563 O THR A 75 -12.664 14.735 8.470 1.00 37.84 O

ATOM 564 CB THR A 75 -13.295 12.090 10.240 1.00 38.69 C

ATOM 565 OG1 THR A 75 -12.387 11.269 9.500 1.00 32.08 O

ATOM 566 CG2 THR A 75 -14.568 12.203 9.455 1.00 40.62 C

ATOM 567 N ASP A 76 -10.691 13.860 9.138 1.00 31.90 N

ATOM 568 CA ASP A 76 -9.897 14.376 8.039 1.00 30.59 C

ATOM 569 C ASP A 76 -9.817 13.402 6.864 1.00 33.18 C

ATOM 570 O ASP A 76 -9.770 13.855 5.718 1.00 32.34 O

ATOM 571 CB ASP A 76 -8.504 14.775 8.546 1.00 32.07 C

ATOM 572 CG ASP A 76 -8.552 15.893 9.572 1.00 45.07 C

ATOM 573 OD1 ASP A 76 -8.885 17.044 9.185 1.00 48.40 O

ATOM 574 OD2 ASP A 76 -8.295 15.615 10.773 1.00 45.10 O

ATOM 575 N VAL A 77 -9.794 12.072 7.148 1.00 28.77 N

ATOM 576 CA VAL A 77 -9.682 10.986 6.155 1.00 27.86 C

ATOM 577 C VAL A 77 -10.280 9.666 6.687 1.00 29.19 C

ATOM 578 O VAL A 77 -10.262 9.426 7.886 1.00 28.95 O

ATOM 579 CB VAL A 77 -8.207 10.831 5.648 1.00 32.44 C

ATOM 580 CG1 VAL A 77 -7.255 10.365 6.758 1.00 32.37 C

ATOM 581 CG2 VAL A 77 -8.108 9.924 4.423 1.00 32.42 C

ATOM 582 N PHE A 78 -10.797 8.824 5.795 1.00 25.08 N

ATOM 583 CA PHE A 78 -11.381 7.516 6.118 1.00 25.12 C

ATOM 584 C PHE A 78 -10.591 6.363 5.516 1.00 27.63 C

ATOM 585 O PHE A 78 -10.160 6.458 4.362 1.00 27.43 O

ATOM 586 CB PHE A 78 -12.822 7.411 5.561 1.00 27.07 C

ATOM 587 CG PHE A 78 -13.899 7.974 6.448 1.00 28.33 C

ATOM 588 CD1 PHE A 78 -14.237 9.321 6.388 1.00 30.69 C

ATOM 589 CD2 PHE A 78 -14.569 7.163 7.355 1.00 29.98 C

ATOM 590 CE1 PHE A 78 -15.224 9.846 7.217 1.00 30.99 C

ATOM 591 CE2 PHE A 78 -15.544 7.695 8.200 1.00 32.36 C

ATOM 592 CZ PHE A 78 -15.860 9.035 8.132 1.00 30.08 C

ATOM 593 N LEU A 79 -10.492 5.245 6.256 1.00 23.04 N

ATOM 594 CA LEU A 79 -9.926 3.996 5.754 1.00 22.79 C

ATOM 595 C LEU A 79 -11.052 2.986 5.732 1.00 26.59 C

ATOM 596 O LEU A 79 -11.486 2.507 6.779 1.00 25.32 O

ATOM 597 CB LEU A 79 -8.774 3.435 6.602 1.00 22.98 C

ATOM 598 CG LEU A 79 -7.491 4.235 6.739 1.00 27.71 C

ATOM 599 CD1 LEU A 79 -6.481 3.450 7.525 1.00 27.73 C

ATOM 600 CD2 LEU A 79 -6.907 4.642 5.387 1.00 29.10 C

ATOM 601 N ILE A 80 -11.571 2.710 4.549 1.00 24.63 N

ATOM 602 CA ILE A 80 -12.612 1.712 4.393 1.00 24.87 C

ATOM 603 C ILE A 80 -11.841 0.414 4.227 1.00 29.13 C

ATOM 604 O ILE A 80 -11.003 0.301 3.333 1.00 27.55 O

ATOM 605 CB ILE A 80 -13.595 2.027 3.221 1.00 28.00 C

ATOM 606 CG1 ILE A 80 -14.298 3.411 3.452 1.00 27.95 C

ATOM 607 CG2 ILE A 80 -14.609 0.859 3.033 1.00 27.68 C

ATOM 608 CD1 ILE A 80 -15.478 3.756 2.550 1.00 32.49 C

ATOM 609 N CYS A 81 -12.069 -0.532 5.137 1.00 27.17 N

ATOM 610 CA CYS A 81 -11.339 -1.787 5.129 1.00 27.50 C

ATOM 611 C CYS A 81 -12.183 -2.978 4.767 1.00 30.38 C

ATOM 612 O CYS A 81 -13.375 -3.019 5.092 1.00 30.29 O

ATOM 613 CB CYS A 81 -10.621 -1.996 6.455 1.00 28.22 C

ATOM 614 SG CYS A 81 -9.472 -0.666 6.865 1.00 32.59 S

ATOM 615 N PHE A 82 -11.547 -3.953 4.084 1.00 24.63 N

ATOM 616 CA PHE A 82 -12.159 -5.222 3.697 1.00 23.37 C

ATOM 617 C PHE A 82 -11.106 -6.317 3.663 1.00 27.53 C

ATOM 618 O PHE A 82 -9.943 -6.061 3.320 1.00 26.63 O

ATOM 619 CB PHE A 82 -12.910 -5.119 2.348 1.00 24.45 C

ATOM 620 CG PHE A 82 -12.044 -5.008 1.117 1.00 24.78 C

ATOM 621 CD1 PHE A 82 -11.594 -6.147 0.456 1.00 27.49 C

ATOM 622 CD2 PHE A 82 -11.684 -3.769 0.612 1.00 25.30 C

ATOM 623 CE1 PHE A 82 -10.767 -6.042 -0.669 1.00 27.44 C

ATOM 624 CE2 PHE A 82 -10.863 -3.667 -0.520 1.00 27.52 C

ATOM 625 CZ PHE A 82 -10.421 -4.804 -1.156 1.00 25.24 C

ATOM 626 N SER A 83 -11.512 -7.537 4.010 1.00 24.46 N

ATOM 627 CA SER A 83 -10.603 -8.660 3.962 1.00 24.58 C

ATOM 628 C SER A 83 -10.449 -9.095 2.509 1.00 31.65 C

ATOM 629 O SER A 83 -11.449 -9.331 1.817 1.00 31.37 O

ATOM 630 CB SER A 83 -11.114 -9.804 4.813 1.00 26.84 C

ATOM 631 OG SER A 83 -10.217 -10.897 4.718 1.00 36.17 O

ATOM 632 N LEU A 84 -9.189 -9.177 2.034 1.00 29.86 N

ATOM 633 CA LEU A 84 -8.907 -9.615 0.666 1.00 30.10 C

ATOM 634 C LEU A 84 -9.328 -11.070 0.469 1.00 36.42 C

ATOM 635 O LEU A 84 -9.573 -11.486 -0.664 1.00 37.45 O

ATOM 636 CB LEU A 84 -7.438 -9.386 0.283 1.00 29.79 C

ATOM 637 CG LEU A 84 -7.077 -7.943 -0.121 1.00 33.47 C

ATOM 638 CD1 LEU A 84 -5.590 -7.662 0.075 1.00 33.64 C

ATOM 639 CD2 LEU A 84 -7.511 -7.630 -1.553 1.00 33.92 C

ATOM 640 N VAL A 85 -9.508 -11.808 1.585 1.00 32.51 N

ATOM 641 CA VAL A 85 -9.950 -13.196 1.577 1.00 31.87 C

ATOM 642 C VAL A 85 -11.442 -13.315 1.997 1.00 33.70 C

ATOM 643 O VAL A 85 -11.942 -14.417 2.243 1.00 32.39 O

ATOM 644 CB VAL A 85 -8.992 -14.120 2.368 1.00 36.39 C

ATOM 645 CG1 VAL A 85 -7.571 -13.995 1.835 1.00 36.65 C

ATOM 646 CG2 VAL A 85 -9.030 -13.856 3.871 1.00 36.25 C

ATOM 647 N SER A 86 -12.157 -12.169 2.025 1.00 29.46 N

ATOM 648 CA SER A 86 -13.588 -12.127 2.317 1.00 28.01 C

ATOM 649 C SER A 86 -14.386 -11.328 1.245 1.00 32.96 C

ATOM 650 O SER A 86 -14.446 -10.093 1.282 1.00 32.20 O

ATOM 651 CB SER A 86 -13.867 -11.650 3.737 1.00 27.96 C

ATOM 652 OG SER A 86 -15.233 -11.330 3.946 1.00 34.17 O

ATOM 653 N PRO A 87 -15.042 -12.032 0.293 1.00 30.65 N

ATOM 654 CA PRO A 87 -15.850 -11.320 -0.713 1.00 30.50 C

ATOM 655 C PRO A 87 -17.065 -10.637 -0.090 1.00 32.00 C

ATOM 656 O PRO A 87 -17.445 -9.563 -0.559 1.00 32.28 O

ATOM 657 CB PRO A 87 -16.219 -12.420 -1.725 1.00 32.50 C

ATOM 658 CG PRO A 87 -15.273 -13.572 -1.410 1.00 37.15 C

ATOM 659 CD PRO A 87 -15.091 -13.488 0.070 1.00 32.43 C

ATOM 660 N ALA A 88 -17.630 -11.221 0.999 1.00 26.33 N

ATOM 661 CA ALA A 88 -18.755 -10.635 1.750 1.00 25.16 C

ATOM 662 C ALA A 88 -18.387 -9.227 2.249 1.00 29.29 C

ATOM 663 O ALA A 88 -19.171 -8.297 2.051 1.00 28.52 O

ATOM 664 CB ALA A 88 -19.146 -11.527 2.920 1.00 25.24 C

ATOM 665 N SER A 89 -17.158 -9.056 2.808 1.00 25.96 N

ATOM 666 CA SER A 89 -16.670 -7.755 3.287 1.00 26.28 C

ATOM 667 C SER A 89 -16.376 -6.762 2.161 1.00 31.48 C

ATOM 668 O SER A 89 -16.497 -5.552 2.368 1.00 29.19 O

ATOM 669 CB SER A 89 -15.446 -7.921 4.178 1.00 28.47 C

ATOM 670 OG SER A 89 -14.346 -8.416 3.440 1.00 31.66 O

ATOM 671 N PHE A 90 -15.988 -7.291 0.973 1.00 30.70 N

ATOM 672 CA PHE A 90 -15.686 -6.517 -0.224 1.00 30.64 C

ATOM 673 C PHE A 90 -16.963 -5.937 -0.820 1.00 33.99 C

ATOM 674 O PHE A 90 -16.965 -4.767 -1.191 1.00 33.91 O

ATOM 675 CB PHE A 90 -14.896 -7.354 -1.258 1.00 32.94 C

ATOM 676 CG PHE A 90 -14.565 -6.607 -2.539 1.00 34.94 C

ATOM 677 CD1 PHE A 90 -13.645 -5.562 -2.538 1.00 38.36 C

ATOM 678 CD2 PHE A 90 -15.225 -6.900 -3.725 1.00 37.15 C

ATOM 679 CE1 PHE A 90 -13.367 -4.848 -3.708 1.00 39.35 C

ATOM 680 CE2 PHE A 90 -14.940 -6.193 -4.899 1.00 40.34 C

ATOM 681 CZ PHE A 90 -14.011 -5.173 -4.882 1.00 38.64 C

ATOM 682 N GLU A 91 -18.054 -6.730 -0.893 1.00 30.80 N

ATOM 683 CA GLU A 91 -19.359 -6.259 -1.409 1.00 30.88 C

ATOM 684 C GLU A 91 -19.945 -5.180 -0.497 1.00 32.45 C

ATOM 685 O GLU A 91 -20.619 -4.259 -0.972 1.00 31.67 O

ATOM 686 CB GLU A 91 -20.359 -7.416 -1.554 1.00 32.73 C

ATOM 687 CG GLU A 91 -20.103 -8.278 -2.780 1.00 47.37 C

ATOM 688 CD GLU A 91 -20.817 -9.611 -2.746 1.00 80.31 C

ATOM 689 OE1 GLU A 91 -20.211 -10.593 -2.257 1.00 75.76 O

ATOM 690 OE2 GLU A 91 -21.983 -9.675 -3.204 1.00 82.70 O

ATOM 691 N ASN A 92 -19.637 -5.284 0.814 1.00 27.64 N

ATOM 692 CA ASN A 92 -20.064 -4.353 1.840 1.00 26.82 C

ATOM 693 C ASN A 92 -19.442 -2.970 1.701 1.00 30.50 C

ATOM 694 O ASN A 92 -19.955 -2.016 2.284 1.00 30.25 O

ATOM 695 CB ASN A 92 -19.848 -4.938 3.212 1.00 25.63 C

ATOM 696 CG ASN A 92 -21.083 -5.619 3.727 1.00 35.81 C

ATOM 697 OD1 ASN A 92 -22.209 -5.157 3.529 1.00 23.63 O

ATOM 698 ND2 ASN A 92 -20.909 -6.730 4.401 1.00 32.21 N

ATOM 699 N VAL A 93 -18.403 -2.842 0.862 1.00 27.22 N

ATOM 700 CA VAL A 93 -17.770 -1.563 0.577 1.00 27.09 C

ATOM 701 C VAL A 93 -18.800 -0.676 -0.107 1.00 31.52 C

ATOM 702 O VAL A 93 -19.130 0.375 0.433 1.00 31.42 O

ATOM 703 CB VAL A 93 -16.436 -1.706 -0.209 1.00 30.64 C

ATOM 704 CG1 VAL A 93 -15.888 -0.345 -0.641 1.00 30.14 C

ATOM 705 CG2 VAL A 93 -15.398 -2.462 0.619 1.00 30.36 C

ATOM 706 N ARG A 94 -19.370 -1.139 -1.236 1.00 29.01 N

ATOM 707 CA ARG A 94 -20.398 -0.404 -1.969 1.00 28.93 C

ATOM 708 C ARG A 94 -21.761 -0.509 -1.303 1.00 34.41 C

ATOM 709 O ARG A 94 -22.532 0.447 -1.378 1.00 36.00 O

ATOM 710 CB ARG A 94 -20.473 -0.820 -3.457 1.00 29.71 C

ATOM 711 CG ARG A 94 -20.813 -2.292 -3.725 1.00 41.70 C

ATOM 712 CD ARG A 94 -20.833 -2.596 -5.210 1.00 52.16 C

ATOM 713 NE ARG A 94 -20.374 -3.960 -5.488 1.00 62.24 N

ATOM 714 CZ ARG A 94 -19.636 -4.305 -6.540 1.00 74.03 C

ATOM 715 NH1 ARG A 94 -19.250 -3.386 -7.419 1.00 56.27 N

ATOM 716 NH2 ARG A 94 -19.261 -5.567 -6.711 1.00 60.01 N

ATOM 717 N ALA A 95 -22.083 -1.663 -0.690 1.00 30.06 N

ATOM 718 CA ALA A 95 -23.387 -1.868 -0.053 1.00 30.15 C

ATOM 719 C ALA A 95 -23.601 -1.023 1.187 1.00 34.49 C

ATOM 720 O ALA A 95 -24.622 -0.346 1.292 1.00 34.38 O

ATOM 721 CB ALA A 95 -23.593 -3.336 0.286 1.00 30.83 C

ATOM 722 N LYS A 96 -22.632 -1.050 2.115 1.00 31.02 N

ATOM 723 CA LYS A 96 -22.740 -0.397 3.406 1.00 29.63 C

ATOM 724 C LYS A 96 -21.793 0.793 3.651 1.00 31.02 C

ATOM 725 O LYS A 96 -22.259 1.888 3.980 1.00 31.05 O

ATOM 726 CB LYS A 96 -22.548 -1.474 4.500 1.00 31.43 C

ATOM 727 CG LYS A 96 -22.728 -0.985 5.944 1.00 40.82 C

ATOM 728 CD LYS A 96 -22.527 -2.116 6.934 1.00 47.74 C

ATOM 729 CE LYS A 96 -22.873 -1.708 8.345 1.00 61.76 C

ATOM 730 NZ LYS A 96 -22.295 -2.647 9.348 1.00 71.02 N

ATOM 731 N TRP A 97 -20.484 0.557 3.561 1.00 25.25 N

ATOM 732 CA TRP A 97 -19.456 1.487 3.986 1.00 24.85 C

ATOM 733 C TRP A 97 -19.349 2.773 3.193 1.00 29.64 C

ATOM 734 O TRP A 97 -19.352 3.826 3.831 1.00 29.22 O

ATOM 735 CB TRP A 97 -18.116 0.774 4.103 1.00 23.60 C

ATOM 736 CG TRP A 97 -18.226 -0.366 5.073 1.00 24.44 C

ATOM 737 CD1 TRP A 97 -18.135 -1.695 4.784 1.00 27.34 C

ATOM 738 CD2 TRP A 97 -18.650 -0.279 6.442 1.00 24.21 C

ATOM 739 NE1 TRP A 97 -18.461 -2.444 5.892 1.00 27.07 N

ATOM 740 CE2 TRP A 97 -18.788 -1.600 6.923 1.00 28.38 C

ATOM 741 CE3 TRP A 97 -18.929 0.793 7.315 1.00 25.45 C

ATOM 742 CZ2 TRP A 97 -19.158 -1.882 8.247 1.00 27.57 C

ATOM 743 CZ3 TRP A 97 -19.343 0.511 8.612 1.00 26.79 C

ATOM 744 CH2 TRP A 97 -19.440 -0.810 9.070 1.00 27.39 C

ATOM 745 N TYR A 98 -19.280 2.729 1.853 1.00 26.62 N

ATOM 746 CA TYR A 98 -19.203 3.959 1.070 1.00 27.01 C

ATOM 747 C TYR A 98 -20.514 4.785 1.197 1.00 31.26 C

ATOM 748 O TYR A 98 -20.398 5.960 1.523 1.00 31.19 O

ATOM 749 CB TYR A 98 -18.771 3.685 -0.381 1.00 29.00 C

ATOM 750 CG TYR A 98 -19.069 4.809 -1.353 1.00 30.46 C

ATOM 751 CD1 TYR A 98 -18.313 5.982 -1.349 1.00 32.09 C

ATOM 752 CD2 TYR A 98 -20.094 4.693 -2.290 1.00 30.73 C

ATOM 753 CE1 TYR A 98 -18.558 7.003 -2.269 1.00 31.63 C

ATOM 754 CE2 TYR A 98 -20.363 5.718 -3.197 1.00 31.70 C

ATOM 755 CZ TYR A 98 -19.594 6.872 -3.179 1.00 39.11 C

ATOM 756 OH TYR A 98 -19.852 7.888 -4.060 1.00 42.18 O

ATOM 757 N PRO A 99 -21.747 4.225 1.068 1.00 29.04 N

ATOM 758 CA PRO A 99 -22.944 5.065 1.299 1.00 29.25 C

ATOM 759 C PRO A 99 -22.985 5.724 2.684 1.00 34.85 C

ATOM 760 O PRO A 99 -23.262 6.926 2.753 1.00 34.01 O

ATOM 761 CB PRO A 99 -24.127 4.111 1.080 1.00 30.87 C

ATOM 762 CG PRO A 99 -23.558 2.742 1.072 1.00 35.50 C

ATOM 763 CD PRO A 99 -22.124 2.847 0.683 1.00 30.98 C

ATOM 764 N GLU A 100 -22.652 4.970 3.773 1.00 33.19 N

ATOM 765 CA GLU A 100 -22.653 5.503 5.147 1.00 33.74 C

ATOM 766 C GLU A 100 -21.627 6.610 5.382 1.00 39.76 C

ATOM 767 O GLU A 100 -21.938 7.585 6.071 1.00 39.92 O

ATOM 768 CB GLU A 100 -22.497 4.401 6.201 1.00 35.22 C

ATOM 769 CG GLU A 100 -22.906 4.877 7.589 1.00 45.37 C

ATOM 770 CD GLU A 100 -22.850 3.863 8.712 1.00 56.14 C

ATOM 771 OE1 GLU A 100 -23.878 3.682 9.408 1.00 50.83 O

ATOM 772 OE2 GLU A 100 -21.753 3.313 8.949 1.00 40.06 O

ATOM 773 N VAL A 101 -20.411 6.461 4.819 1.00 36.69 N

ATOM 774 CA VAL A 101 -19.348 7.459 4.953 1.00 36.29 C

ATOM 775 C VAL A 101 -19.784 8.744 4.239 1.00 42.67 C

ATOM 776 O VAL A 101 -19.733 9.819 4.844 1.00 42.96 O

ATOM 777 CB VAL A 101 -17.947 6.926 4.510 1.00 38.90 C

ATOM 778 CG1 VAL A 101 -16.925 8.048 4.396 1.00 38.44 C

ATOM 779 CG2 VAL A 101 -17.431 5.858 5.470 1.00 38.38 C

ATOM 780 N ARG A 102 -20.284 8.620 2.993 1.00 40.12 N

ATOM 781 CA ARG A 102 -20.749 9.760 2.197 1.00 40.19 C

ATOM 782 C ARG A 102 -21.982 10.456 2.779 1.00 44.86 C

ATOM 783 O ARG A 102 -22.105 11.679 2.658 1.00 43.94 O

ATOM 784 CB ARG A 102 -20.964 9.365 0.730 1.00 39.83 C

ATOM 785 CG ARG A 102 -19.649 9.203 -0.015 1.00 46.84 C

ATOM 786 CD ARG A 102 -18.939 10.520 -0.282 1.00 54.27 C

ATOM 787 NE ARG A 102 -17.536 10.312 -0.641 1.00 65.87 N

ATOM 788 CZ ARG A 102 -16.504 10.605 0.145 1.00 79.84 C

ATOM 789 NH1 ARG A 102 -16.703 11.137 1.346 1.00 58.41 N

ATOM 790 NH2 ARG A 102 -15.263 10.381 -0.269 1.00 71.53 N

ATOM 791 N HIS A 103 -22.862 9.687 3.445 1.00 42.72 N

ATOM 792 CA HIS A 103 -24.068 10.198 4.101 1.00 42.83 C

ATOM 793 C HIS A 103 -23.721 11.299 5.103 1.00 42.83 C

ATOM 794 O HIS A 103 -24.233 12.412 5.007 1.00 40.78 O

ATOM 795 CB HIS A 103 -24.822 9.050 4.801 1.00 44.61 C

ATOM 796 CG HIS A 103 -26.193 9.424 5.259 1.00 49.09 C

ATOM 797 ND1 HIS A 103 -26.412 9.976 6.513 1.00 51.47 N

ATOM 798 CD2 HIS A 103 -27.378 9.324 4.612 1.00 51.70 C

ATOM 799 CE1 HIS A 103 -27.718 10.190 6.588 1.00 51.21 C

ATOM 800 NE2 HIS A 103 -28.342 9.813 5.468 1.00 51.56 N

ATOM 801 N HIS A 104 -22.814 10.990 6.027 1.00 39.32 N

ATOM 802 CA HIS A 104 -22.389 11.881 7.103 1.00 39.13 C

ATOM 803 C HIS A 104 -21.257 12.839 6.699 1.00 44.92 C

ATOM 804 O HIS A 104 -21.220 13.974 7.182 1.00 43.96 O

ATOM 805 CB HIS A 104 -22.030 11.048 8.343 1.00 39.27 C

ATOM 806 CG HIS A 104 -23.149 10.149 8.777 1.00 42.42 C

ATOM 807 ND1 HIS A 104 -23.243 8.843 8.325 1.00 44.26 N

ATOM 808 CD2 HIS A 104 -24.222 10.416 9.559 1.00 43.83 C

ATOM 809 CE1 HIS A 104 -24.352 8.353 8.861 1.00 43.39 C

ATOM 810 NE2 HIS A 104 -24.967 9.257 9.622 1.00 43.62 N

ATOM 811 N CYS A 105 -20.351 12.391 5.811 1.00 43.56 N

ATOM 812 CA CYS A 105 -19.208 13.177 5.335 1.00 45.02 C

ATOM 813 C CYS A 105 -19.128 13.034 3.800 1.00 50.42 C

ATOM 814 O CYS A 105 -18.549 12.059 3.304 1.00 51.08 O

ATOM 815 CB CYS A 105 -17.912 12.725 6.013 1.00 45.68 C

ATOM 816 SG CYS A 105 -17.926 12.835 7.822 1.00 49.67 S

ATOM 817 N PRO A 106 -19.718 13.963 3.019 1.00 46.46 N

ATOM 818 CA PRO A 106 -19.724 13.784 1.556 1.00 45.93 C

ATOM 819 C PRO A 106 -18.472 14.257 0.818 1.00 49.02 C

ATOM 820 O PRO A 106 -18.293 13.892 -0.350 1.00 49.12 O

ATOM 821 CB PRO A 106 -20.977 14.556 1.108 1.00 47.52 C

ATOM 822 CG PRO A 106 -21.577 15.171 2.379 1.00 51.85 C

ATOM 823 CD PRO A 106 -20.497 15.156 3.404 1.00 47.71 C

ATOM 824 N ASN A 107 -17.617 15.061 1.477 1.00 43.73 N

ATOM 825 CA ASN A 107 -16.427 15.613 0.837 1.00 43.13 C

ATOM 826 C ASN A 107 -15.093 15.063 1.376 1.00 44.90 C

ATOM 827 O ASN A 107 -14.046 15.293 0.755 1.00 45.00 O

ATOM 828 CB ASN A 107 -16.459 17.149 0.903 1.00 46.88 C

ATOM 829 CG ASN A 107 -17.725 17.750 0.336 1.00 73.05 C

ATOM 830 OD1 ASN A 107 -18.613 18.198 1.074 1.00 62.76 O

ATOM 831 ND2 ASN A 107 -17.857 17.733 -0.988 1.00 67.57 N

ATOM 832 N THR A 108 -15.136 14.341 2.515 1.00 38.97 N

ATOM 833 CA THR A 108 -13.978 13.750 3.193 1.00 37.32 C

ATOM 834 C THR A 108 -13.304 12.666 2.319 1.00 37.39 C

ATOM 835 O THR A 108 -14.008 11.811 1.783 1.00 36.58 O

ATOM 836 CB THR A 108 -14.388 13.267 4.599 1.00 45.02 C

ATOM 837 OG1 THR A 108 -14.921 14.378 5.333 1.00 44.24 O

ATOM 838 CG2 THR A 108 -13.232 12.634 5.381 1.00 41.53 C

ATOM 839 N PRO A 109 -11.956 12.698 2.150 1.00 31.51 N

ATOM 840 CA PRO A 109 -11.297 11.691 1.289 1.00 31.17 C

ATOM 841 C PRO A 109 -11.317 10.258 1.828 1.00 32.91 C

ATOM 842 O PRO A 109 -11.257 10.049 3.031 1.00 30.86 O

ATOM 843 CB PRO A 109 -9.863 12.218 1.159 1.00 32.98 C

ATOM 844 CG PRO A 109 -9.650 13.041 2.393 1.00 37.11 C

ATOM 845 CD PRO A 109 -10.980 13.673 2.684 1.00 32.19 C

ATOM 846 N ILE A 110 -11.391 9.278 0.923 1.00 30.16 N

ATOM 847 CA ILE A 110 -11.395 7.859 1.268 1.00 30.44 C

ATOM 848 C ILE A 110 -10.193 7.143 0.655 1.00 34.60 C

ATOM 849 O ILE A 110 -9.869 7.368 -0.512 1.00 35.28 O

ATOM 850 CB ILE A 110 -12.718 7.170 0.827 1.00 33.70 C

ATOM 851 CG1 ILE A 110 -13.929 7.701 1.616 1.00 35.11 C

ATOM 852 CG2 ILE A 110 -12.619 5.630 0.907 1.00 33.05 C

ATOM 853 CD1 ILE A 110 -15.334 7.246 1.034 1.00 44.79 C

ATOM 854 N ILE A 111 -9.560 6.257 1.439 1.00 29.75 N

ATOM 855 CA ILE A 111 -8.501 5.355 0.992 1.00 28.72 C

ATOM 856 C ILE A 111 -9.118 3.960 1.181 1.00 32.34 C

ATOM 857 O ILE A 111 -9.548 3.626 2.293 1.00 31.88 O

ATOM 858 CB ILE A 111 -7.194 5.501 1.812 1.00 31.68 C

ATOM 859 CG1 ILE A 111 -6.675 6.973 1.876 1.00 32.02 C

ATOM 860 CG2 ILE A 111 -6.113 4.522 1.331 1.00 32.93 C

ATOM 861 CD1 ILE A 111 -6.065 7.607 0.617 1.00 41.84 C

ATOM 862 N LEU A 112 -9.250 3.186 0.090 1.00 27.76 N

ATOM 863 CA LEU A 112 -9.788 1.832 0.173 1.00 25.91 C

ATOM 864 C LEU A 112 -8.635 0.892 0.553 1.00 28.81 C

ATOM 865 O LEU A 112 -7.574 0.921 -0.085 1.00 29.97 O

ATOM 866 CB LEU A 112 -10.471 1.417 -1.131 1.00 25.56 C

ATOM 867 CG LEU A 112 -11.110 0.006 -1.176 1.00 29.71 C

ATOM 868 CD1 LEU A 112 -12.143 -0.198 -0.051 1.00 29.07 C

ATOM 869 CD2 LEU A 112 -11.751 -0.251 -2.533 1.00 29.92 C

ATOM 870 N VAL A 113 -8.816 0.132 1.643 1.00 21.96 N

ATOM 871 CA VAL A 113 -7.791 -0.749 2.177 1.00 20.84 C

ATOM 872 C VAL A 113 -8.208 -2.226 2.198 1.00 25.30 C

ATOM 873 O VAL A 113 -9.131 -2.604 2.914 1.00 25.86 O

ATOM 874 CB VAL A 113 -7.268 -0.288 3.582 1.00 23.73 C

ATOM 875 CG1 VAL A 113 -6.072 -1.133 4.028 1.00 23.29 C

ATOM 876 CG2 VAL A 113 -6.894 1.196 3.602 1.00 22.98 C

ATOM 877 N GLY A 114 -7.461 -3.043 1.468 1.00 22.14 N

ATOM 878 CA GLY A 114 -7.570 -4.496 1.484 1.00 22.48 C

ATOM 879 C GLY A 114 -6.571 -5.074 2.488 1.00 28.46 C

ATOM 880 O GLY A 114 -5.366 -4.823 2.410 1.00 27.33 O

ATOM 881 N THR A 115 -7.075 -5.812 3.470 1.00 27.15 N

ATOM 882 CA THR A 115 -6.301 -6.429 4.552 1.00 27.41 C

ATOM 883 C THR A 115 -6.133 -7.947 4.326 1.00 31.37 C

ATOM 884 O THR A 115 -6.773 -8.510 3.436 1.00 30.43 O

ATOM 885 CB THR A 115 -7.039 -6.193 5.881 1.00 39.67 C

ATOM 886 OG1 THR A 115 -8.368 -6.705 5.743 1.00 37.91 O

ATOM 887 CG2 THR A 115 -7.079 -4.708 6.288 1.00 41.08 C

ATOM 888 N LYS A 116 -5.308 -8.603 5.174 1.00 28.45 N

ATOM 889 CA LYS A 116 -5.015 -10.041 5.147 1.00 28.70 C

ATOM 890 C LYS A 116 -4.424 -10.471 3.795 1.00 34.12 C

ATOM 891 O LYS A 116 -4.742 -11.539 3.272 1.00 33.75 O

ATOM 892 CB LYS A 116 -6.233 -10.902 5.575 1.00 31.10 C

ATOM 893 CG LYS A 116 -6.968 -10.402 6.825 1.00 37.76 C

ATOM 894 CD LYS A 116 -7.890 -11.468 7.387 1.00 40.79 C

ATOM 895 CE LYS A 116 -8.848 -10.904 8.398 1.00 39.17 C

ATOM 896 NZ LYS A 116 -9.552 -11.984 9.148 1.00 40.14 N

ATOM 897 N LEU A 117 -3.532 -9.620 3.256 1.00 32.29 N

ATOM 898 CA LEU A 117 -2.799 -9.800 2.002 1.00 32.37 C

ATOM 899 C LEU A 117 -1.977 -11.080 2.044 1.00 36.19 C

ATOM 900 O LEU A 117 -1.832 -11.734 1.012 1.00 37.38 O

ATOM 901 CB LEU A 117 -1.900 -8.575 1.770 1.00 32.52 C

ATOM 902 CG LEU A 117 -0.919 -8.549 0.597 1.00 37.60 C

ATOM 903 CD1 LEU A 117 -1.642 -8.435 -0.738 1.00 38.04 C

ATOM 904 CD2 LEU A 117 0.057 -7.391 0.748 1.00 39.27 C

ATOM 905 N ASP A 118 -1.485 -11.459 3.239 1.00 31.63 N

ATOM 906 CA ASP A 118 -0.707 -12.685 3.478 1.00 31.51 C

ATOM 907 C ASP A 118 -1.535 -13.971 3.248 1.00 36.90 C

ATOM 908 O ASP A 118 -0.967 -15.027 2.957 1.00 36.89 O

ATOM 909 CB ASP A 118 -0.135 -12.681 4.906 1.00 32.55 C

ATOM 910 CG ASP A 118 -1.199 -12.706 5.980 1.00 36.75 C

ATOM 911 OD1 ASP A 118 -1.963 -11.720 6.085 1.00 36.35 O

ATOM 912 OD2 ASP A 118 -1.302 -13.729 6.690 1.00 41.21 O

ATOM 913 N LEU A 119 -2.860 -13.883 3.394 1.00 33.66 N

ATOM 914 CA LEU A 119 -3.722 -15.037 3.207 1.00 34.03 C

ATOM 915 C LEU A 119 -4.130 -15.269 1.755 1.00 40.48 C

ATOM 916 O LEU A 119 -4.625 -16.356 1.464 1.00 40.64 O

ATOM 917 CB LEU A 119 -4.969 -14.958 4.107 1.00 33.59 C

ATOM 918 CG LEU A 119 -4.760 -15.137 5.596 1.00 37.79 C

ATOM 919 CD1 LEU A 119 -5.987 -14.712 6.355 1.00 37.74 C

ATOM 920 CD2 LEU A 119 -4.398 -16.575 5.947 1.00 40.91 C

ATOM 921 N ARG A 120 -3.922 -14.284 0.844 1.00 38.02 N

ATOM 922 CA ARG A 120 -4.326 -14.423 -0.566 1.00 39.34 C

ATOM 923 C ARG A 120 -3.635 -15.579 -1.316 1.00 47.47 C

ATOM 924 O ARG A 120 -4.229 -16.137 -2.235 1.00 47.00 O

ATOM 925 CB ARG A 120 -4.184 -13.109 -1.344 1.00 37.77 C

ATOM 926 CG ARG A 120 -5.107 -13.028 -2.567 1.00 39.26 C

ATOM 927 CD ARG A 120 -5.313 -11.614 -3.072 1.00 40.16 C

ATOM 928 NE ARG A 120 -4.046 -10.939 -3.366 1.00 39.14 N

ATOM 929 CZ ARG A 120 -3.938 -9.666 -3.727 1.00 44.01 C

ATOM 930 NH1 ARG A 120 -5.020 -8.920 -3.885 1.00 19.21 N

ATOM 931 NH2 ARG A 120 -2.745 -9.136 -3.957 1.00 38.30 N

ATOM 932 N ASP A 121 -2.414 -15.948 -0.909 1.00 48.01 N

ATOM 933 CA ASP A 121 -1.658 -17.053 -1.509 1.00 49.42 C

ATOM 934 C ASP A 121 -1.713 -18.330 -0.639 1.00 54.80 C

ATOM 935 O ASP A 121 -1.088 -19.336 -0.998 1.00 54.59 O

ATOM 936 CB ASP A 121 -0.198 -16.633 -1.796 1.00 51.90 C

ATOM 937 CG ASP A 121 0.011 -15.815 -3.068 1.00 67.47 C

ATOM 938 OD1 ASP A 121 -0.676 -16.102 -4.088 1.00 69.25 O

ATOM 939 OD2 ASP A 121 0.911 -14.936 -3.070 1.00 72.05 O

ATOM 940 N ASP A 122 -2.480 -18.295 0.481 1.00 51.56 N

ATOM 941 CA ASP A 122 -2.636 -19.418 1.421 1.00 51.62 C

ATOM 942 C ASP A 122 -3.574 -20.506 0.867 1.00 54.63 C

ATOM 943 O ASP A 122 -4.778 -20.273 0.744 1.00 54.16 O

ATOM 944 CB ASP A 122 -3.106 -18.909 2.806 1.00 53.66 C

ATOM 945 CG ASP A 122 -3.106 -19.930 3.932 1.00 66.79 C

ATOM 946 OD1 ASP A 122 -2.228 -20.825 3.928 1.00 68.10 O

ATOM 947 OD2 ASP A 122 -3.944 -19.797 4.853 1.00 73.29 O

ATOM 948 N LYS A 123 -3.004 -21.687 0.529 1.00 50.77 N

ATOM 949 CA LYS A 123 -3.693 -22.859 -0.036 1.00 50.80 C

ATOM 950 C LYS A 123 -4.999 -23.231 0.718 1.00 55.93 C

ATOM 951 O LYS A 123 -6.031 -23.437 0.068 1.00 56.08 O

ATOM 952 CB LYS A 123 -2.723 -24.058 -0.126 1.00 52.85 C

ATOM 953 CG LYS A 123 -3.313 -25.331 -0.749 1.00 61.82 C

ATOM 954 CD LYS A 123 -2.719 -26.619 -0.161 1.00 66.26 C

ATOM 955 CE LYS A 123 -3.224 -26.967 1.227 1.00 77.07 C

ATOM 956 NZ LYS A 123 -4.655 -27.384 1.231 1.00 87.22 N

ATOM 957 N ASP A 124 -4.952 -23.281 2.072 1.00 51.83 N

ATOM 958 CA ASP A 124 -6.105 -23.591 2.928 1.00 51.82 C

ATOM 959 C ASP A 124 -7.232 -22.560 2.776 1.00 54.00 C

ATOM 960 O ASP A 124 -8.402 -22.938 2.668 1.00 53.00 O

ATOM 961 CB ASP A 124 -5.681 -23.716 4.405 1.00 54.37 C

ATOM 962 CG ASP A 124 -4.619 -24.774 4.654 1.00 70.73 C

ATOM 963 OD1 ASP A 124 -3.421 -24.468 4.464 1.00 72.85 O

ATOM 964 OD2 ASP A 124 -4.988 -25.911 5.040 1.00 76.55 O

ATOM 965 N THR A 125 -6.868 -21.264 2.744 1.00 49.91 N

ATOM 966 CA THR A 125 -7.791 -20.135 2.596 1.00 49.41 C

ATOM 967 C THR A 125 -8.437 -20.130 1.205 1.00 52.97 C

ATOM 968 O THR A 125 -9.620 -19.806 1.081 1.00 51.58 O

ATOM 969 CB THR A 125 -7.079 -18.815 2.951 1.00 56.27 C

ATOM 970 OG1 THR A 125 -6.457 -18.940 4.235 1.00 55.06 O

ATOM 971 CG2 THR A 125 -8.023 -17.620 2.960 1.00 52.99 C

ATOM 972 N ILE A 126 -7.659 -20.490 0.165 1.00 50.81 N

ATOM 973 CA ILE A 126 -8.138 -20.561 -1.222 1.00 50.92 C

ATOM 974 C ILE A 126 -9.129 -21.731 -1.364 1.00 54.16 C

ATOM 975 O ILE A 126 -10.181 -21.560 -1.988 1.00 53.24 O

ATOM 976 CB ILE A 126 -6.965 -20.605 -2.251 1.00 54.18 C

ATOM 977 CG1 ILE A 126 -6.057 -19.357 -2.118 1.00 54.52 C

ATOM 978 CG2 ILE A 126 -7.487 -20.736 -3.693 1.00 55.06 C

ATOM 979 CD1 ILE A 126 -4.586 -19.569 -2.526 1.00 61.39 C

ATOM 980 N GLU A 127 -8.808 -22.892 -0.740 1.00 50.62 N

ATOM 981 CA GLU A 127 -9.647 -24.094 -0.741 1.00 50.29 C

ATOM 982 C GLU A 127 -10.990 -23.830 -0.074 1.00 52.16 C

ATOM 983 O GLU A 127 -12.018 -24.163 -0.667 1.00 53.16 O

ATOM 984 CB GLU A 127 -8.930 -25.289 -0.084 1.00 52.12 C

ATOM 985 CG GLU A 127 -8.278 -26.247 -1.077 1.00 66.20 C

ATOM 986 CD GLU A 127 -9.185 -27.298 -1.697 1.00 91.39 C

ATOM 987 OE1 GLU A 127 -9.649 -28.199 -0.959 1.00 84.72 O

ATOM 988 OE2 GLU A 127 -9.384 -27.256 -2.933 1.00 83.42 O

ATOM 989 N LYS A 128 -10.991 -23.193 1.126 1.00 45.24 N

ATOM 990 CA LYS A 128 -12.215 -22.850 1.871 1.00 43.67 C

ATOM 991 C LYS A 128 -13.144 -21.945 1.050 1.00 46.05 C

ATOM 992 O LYS A 128 -14.360 -22.173 1.019 1.00 45.87 O

ATOM 993 CB LYS A 128 -11.889 -22.208 3.232 1.00 45.58 C

ATOM 994 CG LYS A 128 -11.348 -23.193 4.271 1.00 62.31 C

ATOM 995 CD LYS A 128 -11.076 -22.549 5.642 1.00 73.77 C

ATOM 996 CE LYS A 128 -9.718 -21.887 5.772 1.00 82.13 C

ATOM 997 NZ LYS A 128 -9.804 -20.404 5.657 1.00 87.15 N

ATOM 998 N LEU A 129 -12.561 -20.939 0.370 1.00 40.98 N

ATOM 999 CA LEU A 129 -13.275 -19.996 -0.488 1.00 40.25 C

ATOM 1000 C LEU A 129 -13.886 -20.690 -1.714 1.00 44.53 C

ATOM 1001 O LEU A 129 -15.018 -20.369 -2.089 1.00 43.46 O

ATOM 1002 CB LEU A 129 -12.336 -18.874 -0.962 1.00 39.66 C

ATOM 1003 CG LEU A 129 -12.182 -17.663 -0.068 1.00 43.33 C

ATOM 1004 CD1 LEU A 129 -10.982 -16.840 -0.496 1.00 43.22 C

ATOM 1005 CD2 LEU A 129 -13.420 -16.801 -0.097 1.00 44.33 C

ATOM 1006 N LYS A 130 -13.117 -21.614 -2.345 1.00 41.24 N

ATOM 1007 CA LYS A 130 -13.516 -22.364 -3.533 1.00 41.73 C

ATOM 1008 C LYS A 130 -14.728 -23.251 -3.261 1.00 46.77 C

ATOM 1009 O LYS A 130 -15.591 -23.364 -4.134 1.00 47.25 O

ATOM 1010 CB LYS A 130 -12.337 -23.173 -4.102 1.00 44.84 C

ATOM 1011 CG LYS A 130 -12.536 -23.630 -5.554 1.00 61.27 C

ATOM 1012 CD LYS A 130 -11.580 -24.768 -5.980 1.00 75.02 C

ATOM 1013 CE LYS A 130 -11.624 -26.034 -5.132 1.00 88.52 C

ATOM 1014 NZ LYS A 130 -12.992 -26.619 -5.021 1.00 99.39 N

ATOM 1015 N GLU A 131 -14.816 -23.838 -2.042 1.00 43.09 N

ATOM 1016 CA GLU A 131 -15.953 -24.665 -1.610 1.00 42.84 C

ATOM 1017 C GLU A 131 -17.252 -23.834 -1.594 1.00 47.66 C

ATOM 1018 O GLU A 131 -18.312 -24.337 -1.973 1.00 48.78 O

ATOM 1019 CB GLU A 131 -15.699 -25.311 -0.231 1.00 44.13 C

ATOM 1020 CG GLU A 131 -14.509 -26.265 -0.150 1.00 56.89 C

ATOM 1021 CD GLU A 131 -14.435 -27.449 -1.104 1.00 81.36 C

ATOM 1022 OE1 GLU A 131 -15.489 -28.054 -1.408 1.00 79.37 O

ATOM 1023 OE2 GLU A 131 -13.303 -27.800 -1.511 1.00 74.52 O

ATOM 1024 N LYS A 132 -17.149 -22.552 -1.213 1.00 43.59 N

ATOM 1025 CA LYS A 132 -18.264 -21.605 -1.181 1.00 43.34 C

ATOM 1026 C LYS A 132 -18.399 -20.858 -2.539 1.00 49.20 C

ATOM 1027 O LYS A 132 -19.099 -19.837 -2.620 1.00 49.13 O

ATOM 1028 CB LYS A 132 -18.091 -20.636 0.001 1.00 45.18 C

ATOM 1029 CG LYS A 132 -18.121 -21.332 1.360 1.00 53.57 C

ATOM 1030 CD LYS A 132 -17.492 -20.513 2.469 1.00 59.91 C

ATOM 1031 CE LYS A 132 -17.308 -21.368 3.703 1.00 72.48 C

ATOM 1032 NZ LYS A 132 -16.763 -20.598 4.854 1.00 80.41 N

ATOM 1033 N LYS A 133 -17.735 -21.395 -3.607 1.00 46.45 N

ATOM 1034 CA LYS A 133 -17.704 -20.888 -4.998 1.00 46.72 C

ATOM 1035 C LYS A 133 -17.172 -19.440 -5.115 1.00 50.87 C

ATOM 1036 O LYS A 133 -17.499 -18.725 -6.069 1.00 50.54 O

ATOM 1037 CB LYS A 133 -19.076 -21.052 -5.693 1.00 49.37 C

ATOM 1038 CG LYS A 133 -19.331 -22.464 -6.208 1.00 62.71 C

ATOM 1039 CD LYS A 133 -20.822 -22.792 -6.245 1.00 69.61 C

ATOM 1040 CE LYS A 133 -21.106 -24.145 -6.857 1.00 73.48 C

ATOM 1041 NZ LYS A 133 -20.756 -25.262 -5.945 1.00 77.62 N

ATOM 1042 N LEU A 134 -16.317 -19.036 -4.160 1.00 47.50 N

ATOM 1043 CA LEU A 134 -15.730 -17.696 -4.103 1.00 47.07 C

ATOM 1044 C LEU A 134 -14.220 -17.695 -4.306 1.00 49.74 C

ATOM 1045 O LEU A 134 -13.551 -18.690 -4.042 1.00 49.83 O

ATOM 1046 CB LEU A 134 -16.111 -16.970 -2.794 1.00 46.88 C

ATOM 1047 CG LEU A 134 -17.574 -16.538 -2.654 1.00 51.43 C

ATOM 1048 CD1 LEU A 134 -17.931 -16.281 -1.202 1.00 51.81 C

ATOM 1049 CD2 LEU A 134 -17.892 -15.320 -3.514 1.00 53.41 C

ATOM 1050 N THR A 135 -13.700 -16.572 -4.800 1.00 44.34 N

ATOM 1051 CA THR A 135 -12.284 -16.348 -5.065 1.00 43.31 C

ATOM 1052 C THR A 135 -11.778 -15.143 -4.215 1.00 44.19 C

ATOM 1053 O THR A 135 -12.573 -14.225 -3.954 1.00 43.94 O

ATOM 1054 CB THR A 135 -12.076 -16.167 -6.596 1.00 52.05 C

ATOM 1055 OG1 THR A 135 -10.684 -16.191 -6.905 1.00 52.60 O

ATOM 1056 CG2 THR A 135 -12.708 -14.880 -7.151 1.00 50.31 C

ATOM 1057 N PRO A 136 -10.486 -15.090 -3.782 1.00 37.04 N

ATOM 1058 CA PRO A 136 -10.022 -13.892 -3.059 1.00 35.27 C

ATOM 1059 C PRO A 136 -10.080 -12.662 -3.971 1.00 36.31 C

ATOM 1060 O PRO A 136 -10.127 -12.804 -5.202 1.00 36.88 O

ATOM 1061 CB PRO A 136 -8.575 -14.228 -2.685 1.00 37.05 C

ATOM 1062 CG PRO A 136 -8.410 -15.679 -2.928 1.00 41.96 C

ATOM 1063 CD PRO A 136 -9.383 -16.050 -3.987 1.00 37.96 C

ATOM 1064 N ILE A 137 -10.117 -11.463 -3.380 1.00 29.22 N

ATOM 1065 CA ILE A 137 -10.169 -10.227 -4.160 1.00 27.75 C

ATOM 1066 C ILE A 137 -8.790 -9.925 -4.746 1.00 31.88 C

ATOM 1067 O ILE A 137 -7.793 -9.915 -4.028 1.00 31.40 O

ATOM 1068 CB ILE A 137 -10.772 -9.056 -3.338 1.00 30.32 C

ATOM 1069 CG1 ILE A 137 -12.229 -9.362 -2.855 1.00 30.96 C

ATOM 1070 CG2 ILE A 137 -10.676 -7.702 -4.062 1.00 30.47 C

ATOM 1071 CD1 ILE A 137 -13.304 -9.758 -3.931 1.00 39.71 C

ATOM 1072 N THR A 138 -8.738 -9.724 -6.052 1.00 29.44 N

ATOM 1073 CA THR A 138 -7.495 -9.429 -6.762 1.00 30.77 C

ATOM 1074 C THR A 138 -7.254 -7.906 -6.763 1.00 37.41 C

ATOM 1075 O THR A 138 -8.208 -7.140 -6.581 1.00 37.82 O

ATOM 1076 CB THR A 138 -7.569 -10.010 -8.188 1.00 40.04 C

ATOM 1077 OG1 THR A 138 -8.574 -9.313 -8.937 1.00 41.07 O

ATOM 1078 CG2 THR A 138 -7.861 -11.508 -8.198 1.00 36.96 C

ATOM 1079 N TYR A 139 -5.994 -7.467 -6.973 1.00 34.85 N

ATOM 1080 CA TYR A 139 -5.660 -6.038 -7.049 1.00 34.95 C

ATOM 1081 C TYR A 139 -6.551 -5.311 -8.100 1.00 39.83 C

ATOM 1082 O TYR A 139 -7.140 -4.278 -7.740 1.00 38.23 O

ATOM 1083 CB TYR A 139 -4.152 -5.801 -7.290 1.00 35.93 C

ATOM 1084 CG TYR A 139 -3.735 -4.351 -7.138 1.00 38.39 C

ATOM 1085 CD1 TYR A 139 -3.801 -3.468 -8.214 1.00 40.54 C

ATOM 1086 CD2 TYR A 139 -3.249 -3.868 -5.927 1.00 39.31 C

ATOM 1087 CE1 TYR A 139 -3.438 -2.127 -8.074 1.00 41.50 C

ATOM 1088 CE2 TYR A 139 -2.878 -2.532 -5.776 1.00 40.58 C

ATOM 1089 CZ TYR A 139 -2.969 -1.666 -6.855 1.00 48.72 C

ATOM 1090 OH TYR A 139 -2.599 -0.350 -6.711 1.00 49.41 O

ATOM 1091 N PRO A 140 -6.752 -5.856 -9.345 1.00 38.09 N

ATOM 1092 CA PRO A 140 -7.637 -5.169 -10.308 1.00 38.37 C

ATOM 1093 C PRO A 140 -9.079 -4.993 -9.815 1.00 41.53 C

ATOM 1094 O PRO A 140 -9.658 -3.928 -10.059 1.00 39.75 O

ATOM 1095 CB PRO A 140 -7.545 -6.048 -11.564 1.00 39.96 C

ATOM 1096 CG PRO A 140 -6.277 -6.783 -11.422 1.00 43.76 C

ATOM 1097 CD PRO A 140 -6.160 -7.060 -9.968 1.00 39.41 C

ATOM 1098 N GLN A 141 -9.637 -6.015 -9.090 1.00 38.78 N

ATOM 1099 CA GLN A 141 -10.987 -5.968 -8.483 1.00 38.69 C

ATOM 1100 C GLN A 141 -11.044 -4.813 -7.470 1.00 41.69 C

ATOM 1101 O GLN A 141 -11.902 -3.936 -7.589 1.00 41.14 O

ATOM 1102 CB GLN A 141 -11.336 -7.281 -7.755 1.00 39.93 C

ATOM 1103 CG GLN A 141 -11.774 -8.429 -8.642 1.00 52.11 C

ATOM 1104 CD GLN A 141 -12.214 -9.609 -7.809 1.00 69.41 C

ATOM 1105 OE1 GLN A 141 -11.417 -10.480 -7.431 1.00 57.80 O

ATOM 1106 NE2 GLN A 141 -13.499 -9.647 -7.479 1.00 69.65 N

ATOM 1107 N GLY A 142 -10.104 -4.812 -6.522 1.00 37.64 N

ATOM 1108 CA GLY A 142 -9.977 -3.782 -5.497 1.00 37.33 C

ATOM 1109 C GLY A 142 -9.823 -2.365 -6.028 1.00 40.29 C

ATOM 1110 O GLY A 142 -10.465 -1.445 -5.514 1.00 38.82 O

ATOM 1111 N LEU A 143 -8.982 -2.179 -7.072 1.00 37.53 N

ATOM 1112 CA LEU A 143 -8.752 -0.870 -7.707 1.00 37.37 C

ATOM 1113 C LEU A 143 -10.007 -0.350 -8.436 1.00 40.12 C

ATOM 1114 O LEU A 143 -10.245 0.859 -8.459 1.00 40.15 O

ATOM 1115 CB LEU A 143 -7.547 -0.923 -8.660 1.00 37.59 C

ATOM 1116 CG LEU A 143 -7.131 0.406 -9.276 1.00 42.60 C

ATOM 1117 CD1 LEU A 143 -6.005 1.049 -8.492 1.00 43.32 C

ATOM 1118 CD2 LEU A 143 -6.760 0.231 -10.714 1.00 45.45 C

ATOM 1119 N ALA A 144 -10.797 -1.260 -9.026 1.00 35.63 N

ATOM 1120 CA ALA A 144 -12.039 -0.917 -9.717 1.00 35.18 C

ATOM 1121 C ALA A 144 -13.078 -0.367 -8.708 1.00 39.58 C

ATOM 1122 O ALA A 144 -13.650 0.706 -8.941 1.00 39.03 O

ATOM 1123 CB ALA A 144 -12.582 -2.135 -10.441 1.00 35.61 C

ATOM 1124 N MET A 145 -13.256 -1.072 -7.562 1.00 36.36 N

ATOM 1125 CA MET A 145 -14.157 -0.690 -6.468 1.00 36.16 C

ATOM 1126 C MET A 145 -13.757 0.668 -5.917 1.00 39.77 C

ATOM 1127 O MET A 145 -14.628 1.498 -5.656 1.00 39.32 O

ATOM 1128 CB MET A 145 -14.140 -1.747 -5.355 1.00 38.56 C

ATOM 1129 CG MET A 145 -15.086 -1.447 -4.191 1.00 42.76 C

ATOM 1130 SD MET A 145 -16.859 -1.548 -4.577 1.00 47.10 S

ATOM 1131 CE MET A 145 -17.143 -3.273 -4.249 1.00 43.45 C

ATOM 1132 N ALA A 146 -12.439 0.899 -5.788 1.00 36.54 N

ATOM 1133 CA ALA A 146 -11.857 2.141 -5.302 1.00 36.10 C

ATOM 1134 C ALA A 146 -12.293 3.295 -6.174 1.00 40.86 C

ATOM 1135 O ALA A 146 -12.930 4.217 -5.669 1.00 39.91 O

ATOM 1136 CB ALA A 146 -10.344 2.034 -5.265 1.00 36.39 C

ATOM 1137 N LYS A 147 -12.052 3.188 -7.498 1.00 39.01 N

ATOM 1138 CA LYS A 147 -12.417 4.202 -8.498 1.00 38.58 C

ATOM 1139 C LYS A 147 -13.924 4.405 -8.556 1.00 40.61 C

ATOM 1140 O LYS A 147 -14.370 5.548 -8.662 1.00 40.17 O

ATOM 1141 CB LYS A 147 -11.832 3.853 -9.878 1.00 41.27 C

ATOM 1142 CG LYS A 147 -10.325 4.061 -9.950 1.00 58.69 C

ATOM 1143 CD LYS A 147 -9.738 3.645 -11.289 1.00 72.49 C

ATOM 1144 CE LYS A 147 -8.251 3.915 -11.364 1.00 86.66 C

ATOM 1145 NZ LYS A 147 -7.941 5.367 -11.507 1.00 95.56 N

ATOM 1146 N GLU A 148 -14.702 3.302 -8.417 1.00 35.86 N

ATOM 1147 CA GLU A 148 -16.165 3.327 -8.394 1.00 35.61 C

ATOM 1148 C GLU A 148 -16.673 4.166 -7.226 1.00 39.59 C

ATOM 1149 O GLU A 148 -17.470 5.076 -7.445 1.00 39.44 O

ATOM 1150 CB GLU A 148 -16.736 1.906 -8.310 1.00 37.17 C

ATOM 1151 CG GLU A 148 -18.253 1.846 -8.387 1.00 47.74 C

ATOM 1152 CD GLU A 148 -18.901 0.930 -7.370 1.00 66.59 C

ATOM 1153 OE1 GLU A 148 -18.762 -0.307 -7.508 1.00 60.65 O

ATOM 1154 OE2 GLU A 148 -19.558 1.450 -6.439 1.00 60.39 O

ATOM 1155 N ILE A 149 -16.178 3.882 -5.992 1.00 35.82 N

ATOM 1156 CA ILE A 149 -16.556 4.587 -4.755 1.00 34.93 C

ATOM 1157 C ILE A 149 -15.781 5.916 -4.595 1.00 39.25 C

ATOM 1158 O ILE A 149 -15.829 6.535 -3.532 1.00 39.01 O

ATOM 1159 CB ILE A 149 -16.469 3.669 -3.485 1.00 37.81 C

ATOM 1160 CG1 ILE A 149 -15.010 3.382 -3.044 1.00 38.39 C

ATOM 1161 CG2 ILE A 149 -17.272 2.367 -3.637 1.00 37.57 C

ATOM 1162 CD1 ILE A 149 -14.654 4.018 -1.775 1.00 43.71 C

ATOM 1163 N GLY A 150 -15.074 6.327 -5.647 1.00 36.25 N

ATOM 1164 CA GLY A 150 -14.312 7.570 -5.694 1.00 36.20 C

ATOM 1165 C GLY A 150 -13.151 7.687 -4.727 1.00 41.19 C

ATOM 1166 O GLY A 150 -12.786 8.803 -4.343 1.00 41.69 O

ATOM 1167 N ALA A 151 -12.550 6.548 -4.331 1.00 37.79 N

ATOM 1168 CA ALA A 151 -11.403 6.537 -3.414 1.00 37.66 C

ATOM 1169 C ALA A 151 -10.214 7.248 -4.041 1.00 41.72 C

ATOM 1170 O ALA A 151 -10.000 7.162 -5.257 1.00 42.14 O

ATOM 1171 CB ALA A 151 -11.021 5.114 -3.023 1.00 38.05 C

ATOM 1172 N VAL A 152 -9.496 8.009 -3.209 1.00 36.70 N

ATOM 1173 CA VAL A 152 -8.304 8.778 -3.555 1.00 35.88 C

ATOM 1174 C VAL A 152 -7.169 7.806 -3.958 1.00 37.34 C

ATOM 1175 O VAL A 152 -6.394 8.097 -4.867 1.00 37.91 O

ATOM 1176 CB VAL A 152 -7.947 9.687 -2.338 1.00 40.12 C

ATOM 1177 CG1 VAL A 152 -6.461 10.057 -2.282 1.00 40.13 C

ATOM 1178 CG2 VAL A 152 -8.818 10.937 -2.319 1.00 39.74 C

ATOM 1179 N LYS A 153 -7.115 6.644 -3.295 1.00 30.95 N

ATOM 1180 CA LYS A 153 -6.108 5.607 -3.462 1.00 29.10 C

ATOM 1181 C LYS A 153 -6.680 4.243 -3.036 1.00 30.84 C

ATOM 1182 O LYS A 153 -7.660 4.185 -2.287 1.00 29.61 O

ATOM 1183 CB LYS A 153 -4.885 5.972 -2.588 1.00 30.67 C

ATOM 1184 CG LYS A 153 -3.639 5.118 -2.792 1.00 36.80 C

ATOM 1185 CD LYS A 153 -2.502 5.900 -3.412 1.00 45.00 C

ATOM 1186 CE LYS A 153 -1.207 5.140 -3.268 1.00 59.40 C

ATOM 1187 NZ LYS A 153 -0.037 5.935 -3.726 1.00 69.39 N

ATOM 1188 N TYR A 154 -6.069 3.153 -3.546 1.00 27.11 N

ATOM 1189 CA TYR A 154 -6.360 1.755 -3.215 1.00 26.13 C

ATOM 1190 C TYR A 154 -5.048 1.132 -2.774 1.00 30.13 C

ATOM 1191 O TYR A 154 -4.053 1.231 -3.486 1.00 31.30 O

ATOM 1192 CB TYR A 154 -6.970 0.971 -4.388 1.00 25.80 C

ATOM 1193 CG TYR A 154 -7.062 -0.515 -4.114 1.00 26.75 C

ATOM 1194 CD1 TYR A 154 -7.970 -1.019 -3.187 1.00 28.44 C

ATOM 1195 CD2 TYR A 154 -6.194 -1.414 -4.733 1.00 27.39 C

ATOM 1196 CE1 TYR A 154 -8.028 -2.381 -2.897 1.00 28.80 C

ATOM 1197 CE2 TYR A 154 -6.243 -2.781 -4.449 1.00 27.61 C

ATOM 1198 CZ TYR A 154 -7.162 -3.259 -3.531 1.00 33.24 C

ATOM 1199 OH TYR A 154 -7.234 -4.601 -3.260 1.00 32.73 O

ATOM 1200 N LEU A 155 -5.049 0.510 -1.602 1.00 25.76 N

ATOM 1201 CA LEU A 155 -3.872 -0.088 -0.996 1.00 25.31 C

ATOM 1202 C LEU A 155 -4.187 -1.430 -0.398 1.00 29.75 C

ATOM 1203 O LEU A 155 -5.281 -1.644 0.121 1.00 31.60 O

ATOM 1204 CB LEU A 155 -3.328 0.824 0.119 1.00 25.50 C

ATOM 1205 CG LEU A 155 -2.698 2.160 -0.296 1.00 30.65 C

ATOM 1206 CD1 LEU A 155 -2.621 3.094 0.878 1.00 31.51 C

ATOM 1207 CD2 LEU A 155 -1.317 1.980 -0.899 1.00 30.12 C

ATOM 1208 N GLU A 156 -3.211 -2.319 -0.434 1.00 25.26 N

ATOM 1209 CA GLU A 156 -3.286 -3.651 0.144 1.00 25.10 C

ATOM 1210 C GLU A 156 -2.260 -3.713 1.267 1.00 27.77 C

ATOM 1211 O GLU A 156 -1.312 -2.925 1.265 1.00 26.40 O

ATOM 1212 CB GLU A 156 -3.039 -4.736 -0.919 1.00 26.68 C

ATOM 1213 CG GLU A 156 -4.129 -4.767 -1.990 1.00 40.34 C

ATOM 1214 CD GLU A 156 -4.056 -5.853 -3.051 1.00 57.56 C

ATOM 1215 OE1 GLU A 156 -2.964 -6.434 -3.251 1.00 62.41 O

ATOM 1216 OE2 GLU A 156 -5.095 -6.113 -3.698 1.00 36.56 O

ATOM 1217 N CYS A 157 -2.480 -4.588 2.257 1.00 25.16 N

ATOM 1218 CA CYS A 157 -1.583 -4.731 3.400 1.00 26.57 C

ATOM 1219 C CYS A 157 -1.826 -6.008 4.185 1.00 31.19 C

ATOM 1220 O CYS A 157 -2.843 -6.676 4.016 1.00 29.53 O

ATOM 1221 CB CYS A 157 -1.660 -3.507 4.315 1.00 27.67 C

ATOM 1222 SG CYS A 157 -3.258 -3.310 5.151 1.00 32.25 S

ATOM 1223 N SER A 158 -0.900 -6.305 5.090 1.00 29.54 N

ATOM 1224 CA SER A 158 -1.008 -7.403 6.028 1.00 30.08 C

ATOM 1225 C SER A 158 -0.495 -6.886 7.359 1.00 35.61 C

ATOM 1226 O SER A 158 0.627 -6.367 7.417 1.00 37.02 O

ATOM 1227 CB SER A 158 -0.190 -8.607 5.574 1.00 33.55 C

ATOM 1228 OG SER A 158 -0.259 -9.646 6.540 1.00 40.25 O

ATOM 1229 N ALA A 159 -1.314 -6.994 8.419 1.00 30.00 N

ATOM 1230 CA ALA A 159 -0.892 -6.590 9.755 1.00 29.24 C

ATOM 1231 C ALA A 159 0.018 -7.697 10.322 1.00 32.45 C

ATOM 1232 O ALA A 159 0.855 -7.426 11.187 1.00 31.53 O

ATOM 1233 CB ALA A 159 -2.104 -6.385 10.651 1.00 29.92 C

ATOM 1234 N LEU A 160 -0.159 -8.948 9.825 1.00 28.73 N

ATOM 1235 CA LEU A 160 0.617 -10.121 10.225 1.00 28.50 C

ATOM 1236 C LEU A 160 2.088 -9.978 9.781 1.00 35.19 C

ATOM 1237 O LEU A 160 2.994 -10.172 10.599 1.00 34.57 O

ATOM 1238 CB LEU A 160 -0.017 -11.418 9.654 1.00 27.87 C

ATOM 1239 CG LEU A 160 0.416 -12.747 10.298 1.00 31.46 C

ATOM 1240 CD1 LEU A 160 -0.646 -13.789 10.138 1.00 31.64 C

ATOM 1241 CD2 LEU A 160 1.710 -13.278 9.690 1.00 33.97 C

ATOM 1242 N THR A 161 2.319 -9.665 8.490 1.00 33.29 N

ATOM 1243 CA THR A 161 3.676 -9.527 7.961 1.00 33.71 C

ATOM 1244 C THR A 161 4.154 -8.067 7.958 1.00 38.39 C

ATOM 1245 O THR A 161 5.329 -7.821 7.672 1.00 37.88 O

ATOM 1246 CB THR A 161 3.802 -10.201 6.582 1.00 41.30 C

ATOM 1247 OG1 THR A 161 3.247 -9.348 5.579 1.00 43.26 O

ATOM 1248 CG2 THR A 161 3.158 -11.586 6.538 1.00 37.54 C

ATOM 1249 N GLN A 162 3.240 -7.105 8.267 1.00 35.69 N

ATOM 1250 CA GLN A 162 3.461 -5.642 8.287 1.00 35.86 C

ATOM 1251 C GLN A 162 3.757 -5.051 6.882 1.00 40.10 C

ATOM 1252 O GLN A 162 4.101 -3.875 6.765 1.00 40.02 O

ATOM 1253 CB GLN A 162 4.519 -5.218 9.327 1.00 37.03 C

ATOM 1254 CG GLN A 162 4.022 -5.357 10.761 1.00 49.93 C

ATOM 1255 CD GLN A 162 4.607 -4.316 11.673 1.00 71.51 C

ATOM 1256 OE1 GLN A 162 4.587 -3.109 11.403 1.00 65.45 O

ATOM 1257 NE2 GLN A 162 5.119 -4.764 12.795 1.00 70.50 N

ATOM 1258 N ARG A 163 3.567 -5.858 5.827 1.00 36.85 N

ATOM 1259 CA ARG A 163 3.799 -5.485 4.435 1.00 37.10 C

ATOM 1260 C ARG A 163 2.693 -4.544 3.959 1.00 40.26 C

ATOM 1261 O ARG A 163 1.518 -4.909 4.021 1.00 41.33 O

ATOM 1262 CB ARG A 163 3.843 -6.755 3.556 1.00 39.00 C

ATOM 1263 CG ARG A 163 4.463 -6.559 2.167 1.00 50.80 C

ATOM 1264 CD ARG A 163 4.429 -7.829 1.323 1.00 61.00 C

ATOM 1265 NE ARG A 163 5.050 -8.968 2.007 1.00 73.38 N

ATOM 1266 CZ ARG A 163 6.347 -9.265 1.964 1.00 91.34 C

ATOM 1267 NH1 ARG A 163 7.183 -8.524 1.242 1.00 74.75 N

ATOM 1268 NH2 ARG A 163 6.817 -10.309 2.636 1.00 81.23 N

ATOM 1269 N GLY A 164 3.081 -3.358 3.493 1.00 34.06 N

ATOM 1270 CA GLY A 164 2.165 -2.346 2.975 1.00 32.84 C

ATOM 1271 C GLY A 164 1.505 -1.466 4.020 1.00 34.45 C

ATOM 1272 O GLY A 164 0.810 -0.515 3.670 1.00 32.96 O

ATOM 1273 N LEU A 165 1.745 -1.767 5.305 1.00 31.36 N

ATOM 1274 CA LEU A 165 1.185 -1.104 6.485 1.00 31.43 C

ATOM 1275 C LEU A 165 1.618 0.376 6.661 1.00 36.11 C

ATOM 1276 O LEU A 165 0.762 1.251 6.844 1.00 35.14 O

ATOM 1277 CB LEU A 165 1.532 -1.953 7.711 1.00 31.55 C

ATOM 1278 CG LEU A 165 0.940 -1.567 9.042 1.00 37.85 C

ATOM 1279 CD1 LEU A 165 -0.577 -1.726 9.047 1.00 38.33 C

ATOM 1280 CD2 LEU A 165 1.523 -2.428 10.127 1.00 42.16 C

ATOM 1281 N LYS A 166 2.936 0.650 6.594 1.00 33.26 N

ATOM 1282 CA LYS A 166 3.487 2.001 6.715 1.00 33.04 C

ATOM 1283 C LYS A 166 2.929 2.890 5.596 1.00 37.61 C

ATOM 1284 O LYS A 166 2.722 4.083 5.820 1.00 38.65 O

ATOM 1285 CB LYS A 166 5.026 1.962 6.646 1.00 35.74 C

ATOM 1286 CG LYS A 166 5.722 3.240 7.119 1.00 54.81 C

ATOM 1287 CD LYS A 166 6.984 3.549 6.289 1.00 64.60 C

ATOM 1288 CE LYS A 166 7.828 4.687 6.832 1.00 66.01 C

ATOM 1289 NZ LYS A 166 7.109 5.993 6.832 1.00 69.94 N

ATOM 1290 N THR A 167 2.675 2.297 4.402 1.00 32.91 N

ATOM 1291 CA THR A 167 2.167 2.997 3.219 1.00 31.70 C

ATOM 1292 C THR A 167 0.763 3.512 3.462 1.00 32.39 C

ATOM 1293 O THR A 167 0.500 4.681 3.207 1.00 31.20 O

ATOM 1294 CB THR A 167 2.299 2.130 1.942 1.00 37.86 C

ATOM 1295 OG1 THR A 167 3.628 1.608 1.853 1.00 41.36 O

ATOM 1296 CG2 THR A 167 1.998 2.910 0.674 1.00 31.39 C

ATOM 1297 N VAL A 168 -0.117 2.637 3.971 1.00 27.98 N

ATOM 1298 CA VAL A 168 -1.520 2.893 4.294 1.00 27.11 C

ATOM 1299 C VAL A 168 -1.680 4.226 5.039 1.00 34.08 C

ATOM 1300 O VAL A 168 -2.406 5.104 4.558 1.00 34.40 O

ATOM 1301 CB VAL A 168 -2.140 1.687 5.069 1.00 29.49 C

ATOM 1302 CG1 VAL A 168 -3.454 2.057 5.766 1.00 28.97 C

ATOM 1303 CG2 VAL A 168 -2.326 0.473 4.165 1.00 28.74 C

ATOM 1304 N PHE A 169 -0.955 4.389 6.176 1.00 30.99 N

ATOM 1305 CA PHE A 169 -1.058 5.565 7.031 1.00 30.74 C

ATOM 1306 C PHE A 169 -0.239 6.742 6.493 1.00 36.12 C

ATOM 1307 O PHE A 169 -0.594 7.889 6.781 1.00 35.52 O

ATOM 1308 CB PHE A 169 -0.761 5.220 8.504 1.00 32.17 C

ATOM 1309 CG PHE A 169 -1.635 4.077 8.998 1.00 33.38 C

ATOM 1310 CD1 PHE A 169 -2.971 4.289 9.320 1.00 36.08 C

ATOM 1311 CD2 PHE A 169 -1.162 2.770 9.003 1.00 34.64 C

ATOM 1312 CE1 PHE A 169 -3.791 3.225 9.714 1.00 36.69 C

ATOM 1313 CE2 PHE A 169 -1.982 1.708 9.394 1.00 36.80 C

ATOM 1314 CZ PHE A 169 -3.288 1.941 9.752 1.00 35.30 C

ATOM 1315 N ASP A 170 0.773 6.474 5.633 1.00 33.71 N

ATOM 1316 CA ASP A 170 1.550 7.528 4.961 1.00 33.89 C

ATOM 1317 C ASP A 170 0.657 8.224 3.930 1.00 37.26 C

ATOM 1318 O ASP A 170 0.701 9.448 3.808 1.00 36.65 O

ATOM 1319 CB ASP A 170 2.790 6.947 4.262 1.00 36.24 C

ATOM 1320 CG ASP A 170 4.124 7.203 4.949 1.00 52.04 C

ATOM 1321 OD1 ASP A 170 4.129 7.837 6.034 1.00 52.95 O

ATOM 1322 OD2 ASP A 170 5.168 6.775 4.397 1.00 60.56 O

ATOM 1323 N GLU A 171 -0.188 7.432 3.233 1.00 34.21 N

ATOM 1324 CA GLU A 171 -1.145 7.868 2.212 1.00 34.15 C

ATOM 1325 C GLU A 171 -2.367 8.512 2.837 1.00 39.18 C

ATOM 1326 O GLU A 171 -3.026 9.352 2.207 1.00 37.04 O

ATOM 1327 CB GLU A 171 -1.542 6.710 1.277 1.00 35.49 C

ATOM 1328 CG GLU A 171 -0.438 6.263 0.325 1.00 46.34 C

ATOM 1329 CD GLU A 171 0.099 7.286 -0.666 1.00 75.53 C

ATOM 1330 OE1 GLU A 171 -0.681 8.153 -1.127 1.00 73.83 O

ATOM 1331 OE2 GLU A 171 1.299 7.192 -1.014 1.00 73.81 O

ATOM 1332 N ALA A 172 -2.659 8.126 4.092 1.00 38.32 N

ATOM 1333 CA ALA A 172 -3.740 8.709 4.882 1.00 38.61 C

ATOM 1334 C ALA A 172 -3.353 10.179 5.113 1.00 43.28 C

ATOM 1335 O ALA A 172 -4.158 11.075 4.856 1.00 43.20 O

ATOM 1336 CB ALA A 172 -3.870 7.974 6.207 1.00 39.21 C

ATOM 1337 N ILE A 173 -2.083 10.415 5.500 1.00 40.03 N

ATOM 1338 CA ILE A 173 -1.534 11.753 5.703 1.00 39.90 C

ATOM 1339 C ILE A 173 -1.498 12.492 4.358 1.00 45.54 C

ATOM 1340 O ILE A 173 -1.952 13.632 4.285 1.00 44.73 O

ATOM 1341 CB ILE A 173 -0.178 11.693 6.459 1.00 42.31 C

ATOM 1342 CG1 ILE A 173 -0.398 11.148 7.884 1.00 42.12 C

ATOM 1343 CG2 ILE A 173 0.512 13.066 6.511 1.00 42.89 C

ATOM 1344 CD1 ILE A 173 0.728 10.347 8.443 1.00 49.05 C

ATOM 1345 N ARG A 174 -1.065 11.801 3.288 1.00 44.37 N

ATOM 1346 CA ARG A 174 -1.016 12.336 1.917 1.00 45.45 C

ATOM 1347 C ARG A 174 -2.387 12.744 1.347 1.00 51.56 C

ATOM 1348 O ARG A 174 -2.439 13.664 0.531 1.00 51.07 O

ATOM 1349 CB ARG A 174 -0.310 11.361 0.962 1.00 46.12 C

ATOM 1350 CG ARG A 174 1.212 11.360 1.087 1.00 58.44 C

ATOM 1351 CD ARG A 174 1.842 10.411 0.086 1.00 72.90 C

ATOM 1352 NE ARG A 174 3.302 10.517 0.073 1.00 84.18 N

ATOM 1353 CZ ARG A 174 4.121 9.685 0.706 1.00 97.89 C

ATOM 1354 NH1 ARG A 174 3.636 8.669 1.409 1.00 80.24 N

ATOM 1355 NH2 ARG A 174 5.435 9.856 0.631 1.00 87.07 N

ATOM 1356 N ALA A 175 -3.489 12.070 1.772 1.00 49.83 N

ATOM 1357 CA ALA A 175 -4.859 12.376 1.324 1.00 49.96 C

ATOM 1358 C ALA A 175 -5.338 13.702 1.914 1.00 55.92 C

ATOM 1359 O ALA A 175 -6.208 14.366 1.338 1.00 55.40 O

ATOM 1360 CB ALA A 175 -5.807 11.258 1.706 1.00 50.50 C

ATOM 1361 N VAL A 176 -4.743 14.080 3.064 1.00 53.69 N

ATOM 1362 CA VAL A 176 -4.936 15.348 3.778 1.00 54.11 C

ATOM 1363 C VAL A 176 -3.698 16.176 3.338 1.00 59.92 C

ATOM 1364 O VAL A 176 -2.982 15.726 2.440 1.00 60.21 O

ATOM 1365 CB VAL A 176 -5.003 15.118 5.329 1.00 57.65 C

ATOM 1366 CG1 VAL A 176 -5.503 16.359 6.060 1.00 57.30 C

ATOM 1367 CG2 VAL A 176 -5.887 13.922 5.678 1.00 57.36 C

ATOM 1368 N LEU A 177 -3.442 17.359 3.939 1.00 57.04 N

ATOM 1369 CA LEU A 177 -2.272 18.210 3.661 1.00 79.27 C

ATOM 1370 C LEU A 177 -2.123 18.578 2.183 1.00 96.21 C

ATOM 1371 O LEU A 177 -1.963 19.748 1.849 1.00 55.06 O

ATOM 1372 CB LEU A 177 -0.982 17.537 4.186 1.00 79.25 C

ATOM 1373 CG LEU A 177 -0.562 17.807 5.641 1.00 84.07 C

ATOM 1374 CD1 LEU A 177 -1.575 17.280 6.646 1.00 84.05 C

ATOM 1375 CD2 LEU A 177 0.786 17.175 5.932 1.00 86.77 C

ATOM 1376 PB GDP A 201 -11.854 -6.295 13.331 1.00 24.55 P

ATOM 1377 O1B GDP A 201 -11.990 -5.754 11.921 1.00 24.29 O

ATOM 1378 O2B GDP A 201 -11.219 -5.214 14.201 1.00 27.74 O

ATOM 1379 O3B GDP A 201 -13.105 -6.829 13.858 1.00 23.58 O

ATOM 1380 O3A GDP A 201 -10.786 -7.475 13.286 1.00 24.94 O

ATOM 1381 PA GDP A 201 -9.542 -7.862 14.201 1.00 24.48 P

ATOM 1382 O1A GDP A 201 -8.541 -6.761 14.218 1.00 23.11 O

ATOM 1383 O2A GDP A 201 -10.106 -8.096 15.595 1.00 25.22 O

ATOM 1384 O5* GDP A 201 -8.972 -9.255 13.662 1.00 24.52 O

ATOM 1385 C5* GDP A 201 -9.837 -10.152 12.944 1.00 28.42 C

ATOM 1386 C4* GDP A 201 -9.212 -11.512 12.753 1.00 31.11 C

ATOM 1387 O4* GDP A 201 -8.282 -11.467 11.650 1.00 32.61 O

ATOM 1388 C3* GDP A 201 -8.422 -12.067 13.933 1.00 32.41 C

ATOM 1389 O3* GDP A 201 -8.513 -13.488 13.972 1.00 34.07 O

ATOM 1390 C2* GDP A 201 -6.996 -11.610 13.619 1.00 31.23 C

ATOM 1391 O2* GDP A 201 -6.022 -12.432 14.250 1.00 31.53 O

ATOM 1392 C1* GDP A 201 -6.971 -11.756 12.099 1.00 31.44 C

ATOM 1393 N9 GDP A 201 -6.066 -10.835 11.411 1.00 31.87 N

ATOM 1394 C8 GDP A 201 -6.136 -9.467 11.435 1.00 32.21 C

ATOM 1395 N7 GDP A 201 -5.381 -8.879 10.535 1.00 31.01 N

ATOM 1396 C5 GDP A 201 -4.765 -9.929 9.880 1.00 31.18 C

ATOM 1397 C6 GDP A 201 -3.905 -9.950 8.740 1.00 30.56 C

ATOM 1398 O6 GDP A 201 -3.456 -8.983 8.132 1.00 31.02 O

ATOM 1399 N1 GDP A 201 -3.599 -11.238 8.338 1.00 31.40 N

ATOM 1400 C2 GDP A 201 -4.027 -12.387 8.959 1.00 30.84 C

ATOM 1401 N2 GDP A 201 -3.641 -13.543 8.422 1.00 29.64 N

ATOM 1402 N3 GDP A 201 -4.813 -12.392 10.037 1.00 31.79 N

ATOM 1403 C4 GDP A 201 -5.161 -11.147 10.430 1.00 32.67 C

ATOM 1404 H50 GDP A 201 -10.808 -10.207 13.434 1.00 28.90 H

ATOM 1405 H51 GDP A 201 -9.979 -9.632 11.998 1.00 28.23 H

ATOM 1406 H40 GDP A 201 -9.968 -12.217 12.409 1.00 30.96 H

ATOM 1407 H30 GDP A 201 -8.787 -11.669 14.878 1.00 32.69 H

ATOM 1408 H3* GDP A 201 -8.726 -13.744 14.908 1.00 34.06 H

ATOM 1409 H20 GDP A 201 -6.853 -10.571 13.908 1.00 31.37 H

ATOM 1410 H2* GDP A 201 -6.287 -13.368 14.046 1.00 31.87 H

ATOM 1411 H10 GDP A 201 -6.726 -12.772 11.792 1.00 31.23 H

ATOM 1412 H80 GDP A 201 -6.757 -8.925 12.145 1.00 32.46 H

ATOM 1413 H1N GDP A 201 -3.029 -11.295 7.502 1.00 31.69 H

ATOM 1414 H21 GDP A 201 -3.931 -14.421 8.841 1.00 30.03 H

ATOM 1415 H22 GDP A 201 -3.057 -13.617 7.597 1.00 29.35 H

ATOM 1416 MC MG2 A 202 -11.915 -4.482 16.211 1.00 20.12 MG

ATOM 1416 DU1 MG2 A 202 -12.005 -4.482 16.211 1.00 20.12 MG

ATOM 1416 DU2 MG2 A 202 -11.825 -4.482 16.211 1.00 20.12 MG

ATOM 1416 DU3 MG2 A 202 -11.915 -4.572 16.211 1.00 20.12 MG

ATOM 1416 DU4 MG2 A 202 -11.915 -4.392 16.211 1.00 20.12 MG

ATOM 1416 DU5 MG2 A 202 -11.915 -4.482 16.301 1.00 20.12 MG

ATOM 1416 DU6 MG2 A 202 -11.915 -4.482 16.121 1.00 20.12 MG

ATOM 1417 O HOH A 301 -11.769 -12.426 -7.256 1.00 30.00 O

ATOM 1418 O HOH A 302 -18.097 4.920 15.836 1.00 30.00 O

ATOM 1419 O HOH A 303 -8.328 -13.776 -5.957 1.00 30.00 O

ATOM 1420 O HOH A 304 -14.813 1.992 23.553 1.00 26.73 O

ATOM 1421 O HOH A 305 -13.671 -5.260 15.955 1.00 24.83 O

ATOM 1422 O HOH A 306 -0.033 -0.555 0.933 1.00 24.76 O

ATOM 1423 O HOH A 307 -10.846 -3.084 20.189 1.00 10.56 O

ATOM 1424 O HOH A 308 -11.242 -6.144 17.151 1.00 24.43 O

ATOM 1425 O HOH A 309 -12.249 -3.664 18.000 1.00 8.25 O

ATOM 1426 O HOH A 310 -15.832 -4.224 5.121 1.00 8.64 O

ATOM 1427 O HOH A 311 -18.612 -10.063 6.337 1.00 21.87 O

ATOM 1428 O HOH A 312 -24.291 -11.021 -3.971 1.00 30.00 O

ATOM 1429 O HOH A 313 -12.563 -2.824 15.324 1.00 19.70 O

ATOM 1430 O HOH A 314 -9.717 -0.688 19.194 1.00 30.00 O

ATOM 1431 O HOH A 315 2.200 -10.464 2.975 1.00 37.72 O

ATOM 1432 O HOH A 316 -16.657 -13.776 2.647 1.00 30.00 O

ATOM 1433 O HOH A 317 -17.730 2.956 16.471 1.00 16.35 O

ATOM 1434 O HOH A 318 4.599 7.803 17.670 1.00 31.75 O

ATOM 1435 O HOH A 319 3.474 5.146 17.386 1.00 21.75 O

ATOM 1436 O HOH A 320 -4.146 -10.107 -7.392 1.00 22.14 O

ATOM 1437 O HOH A 321 1.166 -12.955 -0.480 1.00 38.92 O

ATOM 1438 O HOH A 322 2.776 16.531 17.209 1.00 30.00 O

ATOM 1439 O HOH A 323 -9.901 18.677 12.505 1.00 33.86 O

ATOM 1377 C10 MOL X 1 -13.969 5.609 19.660 -0.25 -0.02 C

ATOM 1378 N3 MOL X 1 -13.078 5.171 20.641 -0.34 0.01 N

ATOM 1379 C11 MOL X 1 -14.706 6.776 19.859 -0.23 -0.00 C

ATOM 1380 C13 MOL X 1 -14.553 7.505 21.039 -0.26 -0.00 C

ATOM 1381 C14 MOL X 1 -15.366 8.795 21.258 -0.26 -0.00 C

ATOM 1382 N4 MOL X 1 -13.662 7.067 22.019 -0.34 0.00 N

ATOM 1383 C12 MOL X 1 -12.926 5.899 21.820 0.01 -0.10 C

ATOM 1384 N2 MOL X 1 -14.130 4.841 18.418 -0.19 0.01 N

ATOM 1385 C MOL X 1 -15.403 4.106 18.457 -0.21 -0.00 C

ATOM 1386 C5 MOL X 1 -15.391 2.731 18.793 -0.12 -0.00 C

ATOM 1387 C4 MOL X 1 -16.563 2.017 18.839 -0.08 -0.00 C

ATOM 1388 C3 MOL X 1 -17.803 2.649 18.550 -0.12 -0.00 C

ATOM 1389 N MOL X 1 -19.032 1.935 18.589 -0.06 0.02 N

ATOM 1390 C2 MOL X 1 -17.816 4.027 18.212 -0.25 -0.00 C

ATOM 1391 C7 MOL X 1 -20.216 2.570 18.304 -0.25 -0.00 C

ATOM 1392 C9 MOL X 1 -21.549 1.800 18.345 -0.26 -0.01 C

ATOM 1393 C8 MOL X 1 -20.229 3.946 17.968 -0.25 0.00 C

ATOM 1394 C6 MOL X 1 -19.056 4.658 17.923 -0.34 0.00 C

ATOM 1395 C1 MOL X 1 -16.587 4.741 18.173 -0.23 -0.00 C

ATOM 1396 N1 MOL X 1 -19.056 6.087 17.575 -0.15 -0.05 N

ATOM 1397 N5 MOL X 1 -11.987 5.437 22.854 -0.15 0.06 N

ATOM 1398 C15 MOL X 1 -12.742 4.878 23.985 -0.30 -0.01 C

ATOM 1399 C16 MOL X 1 -12.159 3.503 24.361 -0.41 -0.01 C

ATOM 1400 C17 MOL X 1 -14.221 4.718 23.587 -0.48 0.00 C

ATOM 1401 C18 MOL X 1 -14.984 4.011 24.723 -0.39 -0.00 C

ATOM 1402 C19 MOL X 1 -16.071 3.100 24.122 -0.12 -0.02 C

ATOM 1403 N6 MOL X 1 -15.439 1.935 23.487 -0.14 0.01 N

ATOM 1404 C20 MOL X 1 -16.308 1.430 22.414 -0.04 -0.03 C

ATOM 1405 C22 MOL X 1 -17.506 2.381 22.233 -0.05 -0.00 C

ATOM 1406 C21 MOL X 1 -15.234 0.881 24.490 -0.18 -0.03 C

ATOM 1407 C23 MOL X 1 -16.117 -0.332 24.147 -0.28 -0.00 C

Rac1-NSC23766 structure from Autodock Vina

ATOM 1 N GLN A 2 0.578 19.705 12.487 0.00 0.00 N

ATOM 2 CA GLN A 2 -0.825 19.520 12.140 0.00 0.00 C

ATOM 3 C GLN A 2 -1.331 18.147 12.607 0.00 0.00 C

ATOM 4 O GLN A 2 -0.743 17.125 12.243 0.00 0.00 OA

ATOM 5 CB GLN A 2 -1.050 19.726 10.627 0.00 0.00 C

ATOM 6 CG GLN A 2 -2.530 19.905 10.242 0.00 0.00 C

ATOM 7 CD GLN A 2 -2.781 20.211 8.776 0.00 0.00 C

ATOM 8 OE1 GLN A 2 -1.863 20.308 7.953 0.00 0.00 OA

ATOM 9 NE2 GLN A 2 -4.046 20.386 8.416 0.00 0.00 N

ATOM 10 N ALA A 3 -2.418 18.135 13.426 0.00 0.00 N

ATOM 11 CA ALA A 3 -3.041 16.921 13.975 0.00 0.00 C

ATOM 12 C ALA A 3 -4.173 16.436 13.064 0.00 0.00 C

ATOM 13 O ALA A 3 -5.178 17.138 12.894 0.00 0.00 OA

ATOM 14 CB ALA A 3 -3.559 17.173 15.388 0.00 0.00 C

ATOM 15 N ILE A 4 -3.987 15.250 12.455 0.00 0.00 N

ATOM 16 CA ILE A 4 -4.940 14.625 11.535 0.00 0.00 C

ATOM 17 C ILE A 4 -5.801 13.595 12.277 0.00 0.00 C

ATOM 18 O ILE A 4 -5.348 13.017 13.259 0.00 0.00 OA

ATOM 19 CB ILE A 4 -4.181 13.991 10.336 0.00 0.00 C

ATOM 20 CG1 ILE A 4 -3.220 14.997 9.673 0.00 0.00 C

ATOM 21 CG2 ILE A 4 -5.136 13.363 9.311 0.00 0.00 C

ATOM 22 CD1 ILE A 4 -2.371 14.399 8.620 0.00 0.00 C

ATOM 23 N LYS A 5 -7.046 13.387 11.815 0.00 0.00 N

ATOM 24 CA LYS A 5 -7.990 12.407 12.359 0.00 0.00 C

ATOM 25 C LYS A 5 -8.423 11.451 11.244 0.00 0.00 C

ATOM 26 O LYS A 5 -9.010 11.866 10.241 0.00 0.00 OA

ATOM 27 CB LYS A 5 -9.193 13.082 13.015 0.00 0.00 C

ATOM 28 CG LYS A 5 -10.119 12.094 13.698 0.00 0.00 C

ATOM 29 CD LYS A 5 -11.454 12.699 14.010 0.00 0.00 C

ATOM 30 CE LYS A 5 -12.456 11.661 14.403 0.00 0.00 C

ATOM 31 NZ LYS A 5 -13.690 12.296 14.948 0.00 0.00 N1+

ATOM 32 N CYS A 6 -8.108 10.165 11.440 0.00 0.00 N

ATOM 33 CA CYS A 6 -8.360 9.088 10.493 0.00 0.00 C

ATOM 34 C CYS A 6 -9.274 8.037 11.084 0.00 0.00 C

ATOM 35 O CYS A 6 -8.880 7.301 11.990 0.00 0.00 OA

ATOM 36 CB CYS A 6 -7.037 8.491 10.004 0.00 0.00 C

ATOM 37 SG CYS A 6 -7.200 6.942 9.070 0.00 0.00 SA

ATOM 38 N VAL A 7 -10.493 7.956 10.562 0.00 0.00 N

ATOM 39 CA VAL A 7 -11.449 6.951 11.017 0.00 0.00 C

ATOM 40 C VAL A 7 -11.292 5.670 10.177 0.00 0.00 C

ATOM 41 O VAL A 7 -11.189 5.732 8.949 0.00 0.00 OA

ATOM 42 CB VAL A 7 -12.914 7.479 11.056 0.00 0.00 C

ATOM 43 CG1 VAL A 7 -13.853 6.458 11.689 0.00 0.00 C

ATOM 44 CG2 VAL A 7 -12.994 8.791 11.823 0.00 0.00 C

ATOM 45 N VAL A 8 -11.251 4.519 10.855 0.00 0.00 N

ATOM 46 CA VAL A 8 -11.146 3.207 10.223 0.00 0.00 C

ATOM 47 C VAL A 8 -12.533 2.538 10.320 0.00 0.00 C

ATOM 48 O VAL A 8 -13.055 2.366 11.426 0.00 0.00 OA

ATOM 49 CB VAL A 8 -10.058 2.337 10.885 0.00 0.00 C

ATOM 50 CG1 VAL A 8 -9.807 1.079 10.072 0.00 0.00 C

ATOM 51 CG2 VAL A 8 -8.764 3.116 11.084 0.00 0.00 C

ATOM 52 N VAL A 9 -13.130 2.198 9.165 0.00 0.00 N

ATOM 53 CA VAL A 9 -14.451 1.560 9.048 0.00 0.00 C

ATOM 54 C VAL A 9 -14.353 0.270 8.227 0.00 0.00 C

ATOM 55 O VAL A 9 -13.391 0.102 7.477 0.00 0.00 OA

ATOM 56 CB VAL A 9 -15.558 2.517 8.512 0.00 0.00 C

ATOM 57 CG1 VAL A 9 -15.870 3.618 9.520 0.00 0.00 C

ATOM 58 CG2 VAL A 9 -15.198 3.106 7.147 0.00 0.00 C

ATOM 59 N GLY A 10 -15.337 -0.615 8.379 0.00 0.00 N

ATOM 60 CA GLY A 10 -15.372 -1.906 7.700 0.00 0.00 C

ATOM 61 C GLY A 10 -16.065 -2.970 8.518 0.00 0.00 C

ATOM 62 O GLY A 10 -16.413 -2.724 9.678 0.00 0.00 OA

ATOM 63 N ASP A 11 -16.300 -4.159 7.919 0.00 0.00 N

ATOM 64 CA ASP A 11 -17.002 -5.279 8.589 0.00 0.00 C

ATOM 65 C ASP A 11 -16.218 -5.798 9.783 0.00 0.00 C

ATOM 66 O ASP A 11 -15.016 -5.609 9.851 0.00 0.00 OA

ATOM 67 CB ASP A 11 -17.244 -6.470 7.621 0.00 0.00 C

ATOM 68 CG ASP A 11 -18.279 -6.317 6.507 0.00 0.00 C

ATOM 69 OD1 ASP A 11 -18.784 -5.179 6.301 0.00 0.00 OA

ATOM 70 OD2 ASP A 11 -18.573 -7.333 5.829 0.00 0.00 OA1-

ATOM 71 N GLY A 12 -16.911 -6.470 10.701 0.00 0.00 N

ATOM 72 CA GLY A 12 -16.284 -7.158 11.821 0.00 0.00 C

ATOM 73 C GLY A 12 -15.379 -8.252 11.265 0.00 0.00 C

ATOM 74 O GLY A 12 -15.650 -8.799 10.184 0.00 0.00 OA

ATOM 75 N ALA A 13 -14.251 -8.505 11.962 0.00 0.00 N

ATOM 76 CA ALA A 13 -13.217 -9.496 11.673 0.00 0.00 C

ATOM 77 C ALA A 13 -12.358 -9.186 10.422 0.00 0.00 C

ATOM 78 O ALA A 13 -11.515 -10.007 10.064 0.00 0.00 OA

ATOM 79 CB ALA A 13 -13.810 -10.901 11.604 0.00 0.00 C

ATOM 80 N VAL A 14 -12.498 -7.988 9.802 0.00 0.00 N

ATOM 81 CA VAL A 14 -11.665 -7.613 8.631 0.00 0.00 C

ATOM 82 C VAL A 14 -10.207 -7.383 9.029 0.00 0.00 C

ATOM 83 O VAL A 14 -9.338 -7.352 8.171 0.00 0.00 OA

ATOM 84 CB VAL A 14 -12.198 -6.439 7.759 0.00 0.00 C

ATOM 85 CG1 VAL A 14 -13.527 -6.775 7.109 0.00 0.00 C

ATOM 86 CG2 VAL A 14 -12.272 -5.133 8.540 0.00 0.00 C

ATOM 87 N GLY A 15 -9.963 -7.201 10.319 0.00 0.00 N

ATOM 88 CA GLY A 15 -8.623 -7.003 10.845 0.00 0.00 C

ATOM 89 C GLY A 15 -8.262 -5.567 11.141 0.00 0.00 C

ATOM 90 O GLY A 15 -7.068 -5.249 11.184 0.00 0.00 OA

ATOM 91 N LYS A 16 -9.292 -4.700 11.346 0.00 0.00 N

ATOM 92 CA LYS A 16 -9.158 -3.267 11.653 0.00 0.00 C

ATOM 93 C LYS A 16 -8.323 -2.986 12.912 0.00 0.00 C

ATOM 94 O LYS A 16 -7.422 -2.151 12.860 0.00 0.00 OA

ATOM 95 CB LYS A 16 -10.539 -2.604 11.828 0.00 0.00 C

ATOM 96 CG LYS A 16 -11.284 -2.296 10.533 0.00 0.00 C

ATOM 97 CD LYS A 16 -12.641 -1.584 10.752 0.00 0.00 C

ATOM 98 CE LYS A 16 -13.580 -2.119 11.830 0.00 0.00 C

ATOM 99 NZ LYS A 16 -13.945 -3.549 11.645 0.00 0.00 N1+

ATOM 100 N THR A 17 -8.635 -3.649 14.037 0.00 0.00 N

ATOM 101 CA THR A 17 -7.923 -3.434 15.297 0.00 0.00 C

ATOM 102 C THR A 17 -6.478 -3.934 15.239 0.00 0.00 C

ATOM 103 O THR A 17 -5.594 -3.302 15.829 0.00 0.00 OA

ATOM 104 CB THR A 17 -8.696 -4.031 16.483 0.00 0.00 C

ATOM 105 OG1 THR A 17 -10.100 -3.812 16.317 0.00 0.00 OA

ATOM 106 CG2 THR A 17 -8.204 -3.504 17.838 0.00 0.00 C

ATOM 107 N CYS A 18 -6.240 -5.066 14.539 0.00 0.00 N

ATOM 108 CA CYS A 18 -4.898 -5.631 14.360 0.00 0.00 C

ATOM 109 C CYS A 18 -4.049 -4.709 13.502 0.00 0.00 C

ATOM 110 O CYS A 18 -2.856 -4.588 13.742 0.00 0.00 OA

ATOM 111 CB CYS A 18 -4.971 -7.026 13.750 0.00 0.00 C

ATOM 112 SG CYS A 18 -5.387 -8.329 14.931 0.00 0.00 SA

ATOM 113 N LEU A 19 -4.666 -4.070 12.507 0.00 0.00 N

ATOM 114 CA LEU A 19 -4.032 -3.135 11.586 0.00 0.00 C

ATOM 115 C LEU A 19 -3.485 -1.925 12.386 0.00 0.00 C

ATOM 116 O LEU A 19 -2.300 -1.602 12.289 0.00 0.00 OA

ATOM 117 CB LEU A 19 -5.086 -2.719 10.554 0.00 0.00 C

ATOM 118 CG LEU A 19 -4.725 -1.714 9.497 0.00 0.00 C

ATOM 119 CD1 LEU A 19 -3.998 -2.374 8.376 0.00 0.00 C

ATOM 120 CD2 LEU A 19 -5.979 -1.079 8.932 0.00 0.00 C

ATOM 121 N LEU A 20 -4.344 -1.316 13.210 0.00 0.00 N

ATOM 122 CA LEU A 20 -4.022 -0.184 14.079 0.00 0.00 C

ATOM 123 C LEU A 20 -3.001 -0.528 15.168 0.00 0.00 C

ATOM 124 O LEU A 20 -2.127 0.303 15.435 0.00 0.00 OA

ATOM 125 CB LEU A 20 -5.303 0.413 14.705 0.00 0.00 C

ATOM 126 CG LEU A 20 -6.288 0.998 13.711 0.00 0.00 C

ATOM 127 CD1 LEU A 20 -7.650 1.098 14.295 0.00 0.00 C

ATOM 128 CD2 LEU A 20 -5.835 2.325 13.216 0.00 0.00 C

ATOM 129 N ILE A 21 -3.097 -1.728 15.799 0.00 0.00 N

ATOM 130 CA ILE A 21 -2.129 -2.114 16.831 0.00 0.00 C

ATOM 131 C ILE A 21 -0.778 -2.405 16.182 0.00 0.00 C

ATOM 132 O ILE A 21 0.210 -1.846 16.634 0.00 0.00 OA

ATOM 133 CB ILE A 21 -2.637 -3.227 17.797 0.00 0.00 C

ATOM 134 CG1 ILE A 21 -3.434 -2.601 18.956 0.00 0.00 C

ATOM 135 CG2 ILE A 21 -1.498 -4.078 18.382 0.00 0.00 C

ATOM 136 CD1 ILE A 21 -4.801 -2.447 18.728 0.00 0.00 C

ATOM 137 N SER A 22 -0.744 -3.183 15.078 0.00 0.00 N

ATOM 138 CA SER A 22 0.490 -3.471 14.336 0.00 0.00 C

ATOM 139 C SER A 22 1.229 -2.205 13.971 0.00 0.00 C

ATOM 140 O SER A 22 2.413 -2.104 14.237 0.00 0.00 OA

ATOM 141 CB SER A 22 0.202 -4.268 13.068 0.00 0.00 C

ATOM 142 OG SER A 22 -0.279 -5.559 13.393 0.00 0.00 OA

ATOM 143 N TYR A 23 0.531 -1.234 13.394 0.00 0.00 N

ATOM 144 CA TYR A 23 1.121 0.019 12.954 0.00 0.00 C

ATOM 145 C TYR A 23 1.744 0.852 14.068 0.00 0.00 C

ATOM 146 O TYR A 23 2.887 1.279 13.931 0.00 0.00 OA

ATOM 147 CB TYR A 23 0.102 0.854 12.167 0.00 0.00 C

ATOM 148 CG TYR A 23 0.660 2.199 11.750 0.00 0.00 A

ATOM 149 CD1 TYR A 23 1.692 2.288 10.823 0.00 0.00 A

ATOM 150 CD2 TYR A 23 0.159 3.381 12.288 0.00 0.00 A

ATOM 151 CE1 TYR A 23 2.213 3.518 10.438 0.00 0.00 A

ATOM 152 CE2 TYR A 23 0.690 4.619 11.926 0.00 0.00 A

ATOM 153 CZ TYR A 23 1.722 4.680 11.003 0.00 0.00 A

ATOM 154 OH TYR A 23 2.242 5.885 10.606 0.00 0.00 OA

ATOM 155 N THR A 24 0.985 1.101 15.140 0.00 0.00 N

ATOM 156 CA THR A 24 1.389 1.942 16.268 0.00 0.00 C

ATOM 157 C THR A 24 2.380 1.278 17.203 0.00 0.00 C

ATOM 158 O THR A 24 3.267 1.957 17.724 0.00 0.00 OA

ATOM 159 CB THR A 24 0.148 2.421 17.047 0.00 0.00 C

ATOM 160 OG1 THR A 24 -0.599 1.282 17.482 0.00 0.00 OA

ATOM 161 CG2 THR A 24 -0.740 3.358 16.238 0.00 0.00 C

ATOM 162 N THR A 25 2.211 -0.038 17.432 0.00 0.00 N

ATOM 163 CA THR A 25 2.968 -0.871 18.373 0.00 0.00 C

ATOM 164 C THR A 25 4.183 -1.560 17.717 0.00 0.00 C

ATOM 165 O THR A 25 5.132 -1.914 18.426 0.00 0.00 OA

ATOM 166 CB THR A 25 1.950 -1.838 19.038 0.00 0.00 C

ATOM 167 OG1 THR A 25 1.402 -1.224 20.207 0.00 0.00 OA

ATOM 168 CG2 THR A 25 2.489 -3.232 19.346 0.00 0.00 C

ATOM 169 N ASN A 26 4.169 -1.711 16.376 0.00 0.00 N

ATOM 170 CA ASN A 26 5.192 -2.409 15.578 0.00 0.00 C

ATOM 171 C ASN A 26 5.116 -3.931 15.777 0.00 0.00 C

ATOM 172 O ASN A 26 6.059 -4.636 15.417 0.00 0.00 OA

ATOM 173 CB ASN A 26 6.617 -1.857 15.791 0.00 0.00 C

ATOM 174 CG ASN A 26 6.916 -0.645 14.956 0.00 0.00 C

ATOM 175 OD1 ASN A 26 7.303 -0.754 13.786 0.00 0.00 OA

ATOM 176 ND2 ASN A 26 6.717 0.537 15.530 0.00 0.00 N

ATOM 177 N ALA A 27 3.976 -4.441 16.304 0.00 0.00 N

ATOM 178 CA ALA A 27 3.783 -5.871 16.548 0.00 0.00 C

ATOM 179 C ALA A 27 2.376 -6.345 16.265 0.00 0.00 C

ATOM 180 O ALA A 27 1.417 -5.613 16.512 0.00 0.00 OA

ATOM 181 CB ALA A 27 4.167 -6.225 17.981 0.00 0.00 C

ATOM 182 N PHE A 28 2.249 -7.590 15.772 0.00 0.00 N

ATOM 183 CA PHE A 28 0.946 -8.197 15.504 0.00 0.00 C

ATOM 184 C PHE A 28 0.346 -8.754 16.818 0.00 0.00 C

ATOM 185 O PHE A 28 0.974 -9.614 17.440 0.00 0.00 OA

ATOM 186 CB PHE A 28 1.046 -9.273 14.411 0.00 0.00 C

ATOM 187 CG PHE A 28 -0.300 -9.804 13.978 0.00 0.00 A

ATOM 188 CD1 PHE A 28 -1.181 -9.008 13.252 0.00 0.00 A

ATOM 189 CD2 PHE A 28 -0.699 -11.095 14.316 0.00 0.00 A

ATOM 190 CE1 PHE A 28 -2.424 -9.499 12.856 0.00 0.00 A

ATOM 191 CE2 PHE A 28 -1.951 -11.580 13.927 0.00 0.00 A

ATOM 192 CZ PHE A 28 -2.804 -10.779 13.201 0.00 0.00 A

ATOM 193 N PRO A 29 -0.854 -8.291 17.256 0.00 0.00 N

ATOM 194 CA PRO A 29 -1.413 -8.757 18.543 0.00 0.00 C

ATOM 195 C PRO A 29 -1.809 -10.237 18.662 0.00 0.00 C

ATOM 196 O PRO A 29 -2.305 -10.634 19.714 0.00 0.00 OA

ATOM 197 CB PRO A 29 -2.632 -7.852 18.753 0.00 0.00 C

ATOM 198 CG PRO A 29 -2.985 -7.383 17.430 0.00 0.00 C

ATOM 199 CD PRO A 29 -1.712 -7.256 16.657 0.00 0.00 C

ATOM 200 N GLY A 30 -1.578 -11.027 17.616 0.00 0.00 N

ATOM 201 CA GLY A 30 -1.886 -12.452 17.597 0.00 0.00 C

ATOM 202 C GLY A 30 -3.322 -12.797 17.942 0.00 0.00 C

ATOM 203 O GLY A 30 -4.256 -12.302 17.297 0.00 0.00 OA

ATOM 204 N GLU A 31 -3.499 -13.645 18.982 0.00 0.00 N

ATOM 205 CA GLU A 31 -4.817 -14.106 19.439 0.00 0.00 C

ATOM 206 C GLU A 31 -5.517 -13.080 20.338 0.00 0.00 C

ATOM 207 O GLU A 31 -6.657 -12.710 20.026 0.00 0.00 OA

ATOM 208 CB GLU A 31 -4.774 -15.514 20.083 0.00 0.00 C

ATOM 209 CG GLU A 31 -3.807 -15.682 21.252 0.00 0.00 C

ATOM 210 CD GLU A 31 -4.047 -16.858 22.184 0.00 0.00 C

ATOM 211 OE1 GLU A 31 -5.113 -17.509 22.083 0.00 0.00 OA

ATOM 212 OE2 GLU A 31 -3.171 -17.106 23.044 0.00 0.00 OA1-

ATOM 213 N TYR A 32 -4.857 -12.598 21.426 0.00 0.00 N

ATOM 214 CA TYR A 32 -5.517 -11.594 22.262 0.00 0.00 C

ATOM 215 C TYR A 32 -5.366 -10.188 21.694 0.00 0.00 C

ATOM 216 O TYR A 32 -4.346 -9.526 21.893 0.00 0.00 OA

ATOM 217 CB TYR A 32 -5.164 -11.637 23.771 0.00 0.00 C

ATOM 218 CG TYR A 32 -6.023 -10.655 24.549 0.00 0.00 A

ATOM 219 CD1 TYR A 32 -7.368 -10.920 24.795 0.00 0.00 A

ATOM 220 CD2 TYR A 32 -5.537 -9.394 24.894 0.00 0.00 A

ATOM 221 CE1 TYR A 32 -8.191 -9.980 25.417 0.00 0.00 A

ATOM 222 CE2 TYR A 32 -6.355 -8.441 25.502 0.00 0.00 A

ATOM 223 CZ TYR A 32 -7.679 -8.741 25.768 0.00 0.00 A

ATOM 224 OH TYR A 32 -8.480 -7.814 26.389 0.00 0.00 OA

ATOM 225 N ILE A 33 -6.432 -9.743 21.021 0.00 0.00 N

ATOM 226 CA ILE A 33 -6.607 -8.421 20.424 0.00 0.00 C

ATOM 227 C ILE A 33 -7.422 -7.615 21.447 0.00 0.00 C

ATOM 228 O ILE A 33 -8.402 -8.159 21.960 0.00 0.00 OA

ATOM 229 CB ILE A 33 -7.379 -8.561 19.077 0.00 0.00 C

ATOM 230 CG1 ILE A 33 -6.716 -9.592 18.139 0.00 0.00 C

ATOM 231 CG2 ILE A 33 -7.563 -7.224 18.370 0.00 0.00 C

ATOM 232 CD1 ILE A 33 -7.680 -10.614 17.539 0.00 0.00 C

ATOM 233 N PRO A 34 -7.073 -6.343 21.777 0.00 0.00 N

ATOM 234 CA PRO A 34 -7.923 -5.578 22.715 0.00 0.00 C

ATOM 235 C PRO A 34 -9.373 -5.517 22.231 0.00 0.00 C

ATOM 236 O PRO A 34 -9.641 -5.416 21.031 0.00 0.00 OA

ATOM 237 CB PRO A 34 -7.282 -4.182 22.742 0.00 0.00 C

ATOM 238 CG PRO A 34 -6.419 -4.117 21.529 0.00 0.00 C

ATOM 239 CD PRO A 34 -5.949 -5.519 21.278 0.00 0.00 C

ATOM 240 N THR A 35 -10.307 -5.625 23.174 0.00 0.00 N

ATOM 241 CA THR A 35 -11.735 -5.654 22.857 0.00 0.00 C

ATOM 242 C THR A 35 -12.388 -4.271 23.026 0.00 0.00 C

ATOM 243 O THR A 35 -13.414 -4.010 22.410 0.00 0.00 OA

ATOM 244 CB THR A 35 -12.411 -6.772 23.637 0.00 0.00 C

ATOM 245 OG1 THR A 35 -12.150 -6.562 25.026 0.00 0.00 OA

ATOM 246 CG2 THR A 35 -11.901 -8.151 23.221 0.00 0.00 C

ATOM 247 N VAL A 36 -11.760 -3.375 23.795 0.00 0.00 N

ATOM 248 CA VAL A 36 -12.232 -1.996 23.974 0.00 0.00 C

ATOM 249 C VAL A 36 -11.975 -1.234 22.668 0.00 0.00 C

ATOM 250 O VAL A 36 -10.835 -1.230 22.178 0.00 0.00 OA

ATOM 251 CB VAL A 36 -11.548 -1.303 25.194 0.00 0.00 C

ATOM 252 CG1 VAL A 36 -11.923 0.180 25.284 0.00 0.00 C

ATOM 253 CG2 VAL A 36 -11.888 -2.027 26.498 0.00 0.00 C

ATOM 254 N PHE A 37 -13.023 -0.620 22.086 0.00 0.00 N

ATOM 255 CA PHE A 37 -12.844 0.148 20.852 0.00 0.00 C

ATOM 256 C PHE A 37 -12.183 1.460 21.232 0.00 0.00 C

ATOM 257 O PHE A 37 -12.756 2.263 21.964 0.00 0.00 OA

ATOM 258 CB PHE A 37 -14.155 0.356 20.085 0.00 0.00 C

ATOM 259 CG PHE A 37 -14.915 -0.909 19.760 0.00 0.00 A

ATOM 260 CD1 PHE A 37 -14.669 -1.606 18.576 0.00 0.00 A

ATOM 261 CD2 PHE A 37 -15.901 -1.392 20.623 0.00 0.00 A

ATOM 262 CE1 PHE A 37 -15.387 -2.770 18.270 0.00 0.00 A

ATOM 263 CE2 PHE A 37 -16.622 -2.544 20.308 0.00 0.00 A

ATOM 264 CZ PHE A 37 -16.363 -3.220 19.131 0.00 0.00 A

ATOM 265 N ASP A 38 -10.941 1.624 20.830 0.00 0.00 N

ATOM 266 CA ASP A 38 -10.191 2.781 21.232 0.00 0.00 C

ATOM 267 C ASP A 38 -9.717 3.603 20.051 0.00 0.00 C

ATOM 268 O ASP A 38 -10.070 3.326 18.907 0.00 0.00 OA

ATOM 269 CB ASP A 38 -9.002 2.314 22.104 0.00 0.00 C

ATOM 270 CG ASP A 38 -8.596 3.254 23.238 0.00 0.00 C

ATOM 271 OD1 ASP A 38 -9.012 4.441 23.214 0.00 0.00 OA

ATOM 272 OD2 ASP A 38 -7.824 2.818 24.119 0.00 0.00 OA1-

ATOM 273 N ASN A 39 -8.966 4.663 20.357 0.00 0.00 N

ATOM 274 CA ASN A 39 -8.305 5.552 19.418 0.00 0.00 C

ATOM 275 C ASN A 39 -6.831 5.464 19.707 0.00 0.00 C

ATOM 276 O ASN A 39 -6.425 5.254 20.842 0.00 0.00 OA

ATOM 277 CB ASN A 39 -8.800 6.999 19.526 0.00 0.00 C

ATOM 278 CG ASN A 39 -8.790 7.600 20.915 0.00 0.00 C

ATOM 279 OD1 ASN A 39 -9.794 7.567 21.638 0.00 0.00 OA

ATOM 280 ND2 ASN A 39 -7.668 8.208 21.299 0.00 0.00 N

ATOM 281 N TYR A 40 -6.036 5.612 18.682 0.00 0.00 N

ATOM 282 CA TYR A 40 -4.586 5.507 18.758 0.00 0.00 C

ATOM 283 C TYR A 40 -3.947 6.764 18.173 0.00 0.00 C

ATOM 284 O TYR A 40 -4.649 7.622 17.641 0.00 0.00 OA

ATOM 285 CB TYR A 40 -4.126 4.220 18.046 0.00 0.00 C

ATOM 286 CG TYR A 40 -4.869 2.992 18.530 0.00 0.00 A

ATOM 287 CD1 TYR A 40 -4.534 2.380 19.737 0.00 0.00 A

ATOM 288 CD2 TYR A 40 -5.968 2.497 17.832 0.00 0.00 A

ATOM 289 CE1 TYR A 40 -5.228 1.264 20.200 0.00 0.00 A

ATOM 290 CE2 TYR A 40 -6.673 1.382 18.287 0.00 0.00 A

ATOM 291 CZ TYR A 40 -6.295 0.764 19.468 0.00 0.00 A

ATOM 292 OH TYR A 40 -6.985 -0.335 19.918 0.00 0.00 OA

ATOM 293 N SER A 41 -2.631 6.888 18.313 0.00 0.00 N

ATOM 294 CA SER A 41 -1.900 8.057 17.855 0.00 0.00 C

ATOM 295 C SER A 41 -0.550 7.663 17.305 0.00 0.00 C

ATOM 296 O SER A 41 0.046 6.699 17.775 0.00 0.00 OA

ATOM 297 CB SER A 41 -1.715 9.039 19.014 0.00 0.00 C

ATOM 298 OG SER A 41 -1.047 10.224 18.615 0.00 0.00 OA

ATOM 299 N ALA A 42 -0.058 8.432 16.338 0.00 0.00 N

ATOM 300 CA ALA A 42 1.277 8.256 15.782 0.00 0.00 C

ATOM 301 C ALA A 42 1.875 9.613 15.359 0.00 0.00 C

ATOM 302 O ALA A 42 1.262 10.338 14.568 0.00 0.00 OA

ATOM 303 CB ALA A 42 1.254 7.280 14.615 0.00 0.00 C

ATOM 304 N ASN A 43 3.040 9.972 15.931 0.00 0.00 N

ATOM 305 CA ASN A 43 3.778 11.188 15.569 0.00 0.00 C

ATOM 306 C ASN A 43 4.694 10.793 14.428 0.00 0.00 C

ATOM 307 O ASN A 43 5.680 10.075 14.634 0.00 0.00 OA

ATOM 308 CB ASN A 43 4.594 11.723 16.741 0.00 0.00 C

ATOM 309 CG ASN A 43 3.758 12.302 17.840 0.00 0.00 C

ATOM 310 OD1 ASN A 43 2.948 13.210 17.630 0.00 0.00 OA

ATOM 311 ND2 ASN A 43 3.995 11.840 19.053 0.00 0.00 N

ATOM 312 N VAL A 44 4.324 11.185 13.212 0.00 0.00 N

ATOM 313 CA VAL A 44 5.079 10.826 12.018 0.00 0.00 C

ATOM 314 C VAL A 44 5.333 12.015 11.119 0.00 0.00 C

ATOM 315 O VAL A 44 4.470 12.875 10.978 0.00 0.00 OA

ATOM 316 CB VAL A 44 4.444 9.638 11.245 0.00 0.00 C

ATOM 317 CG1 VAL A 44 4.464 8.368 12.089 0.00 0.00 C

ATOM 318 CG2 VAL A 44 3.023 9.955 10.784 0.00 0.00 C

ATOM 319 N MET A 45 6.523 12.069 10.515 0.00 0.00 N

ATOM 320 CA MET A 45 6.878 13.139 9.588 0.00 0.00 C

ATOM 321 C MET A 45 7.274 12.568 8.238 0.00 0.00 C

ATOM 322 O MET A 45 8.344 11.978 8.107 0.00 0.00 OA

ATOM 323 CB MET A 45 7.928 14.105 10.170 0.00 0.00 C

ATOM 324 CG MET A 45 9.304 13.501 10.365 0.00 0.00 C

ATOM 325 SD MET A 45 10.338 14.574 11.386 0.00 0.00 SA

ATOM 326 CE MET A 45 9.908 13.980 13.001 0.00 0.00 C

ATOM 327 N VAL A 46 6.363 12.668 7.253 0.00 0.00 N

ATOM 328 CA VAL A 46 6.588 12.178 5.889 0.00 0.00 C

ATOM 329 C VAL A 46 6.893 13.386 4.975 0.00 0.00 C

ATOM 330 O VAL A 46 5.986 14.134 4.594 0.00 0.00 OA

ATOM 331 CB VAL A 46 5.481 11.207 5.365 0.00 0.00 C

ATOM 332 CG1 VAL A 46 4.078 11.817 5.432 0.00 0.00 C

ATOM 333 CG2 VAL A 46 5.800 10.689 3.966 0.00 0.00 C

ATOM 334 N ASP A 47 8.206 13.588 4.693 0.00 0.00 N

ATOM 335 CA ASP A 47 8.820 14.672 3.906 0.00 0.00 C

ATOM 336 C ASP A 47 8.263 16.072 4.284 0.00 0.00 C

ATOM 337 O ASP A 47 7.685 16.785 3.453 0.00 0.00 OA

ATOM 338 CB ASP A 47 8.796 14.410 2.380 0.00 0.00 C

ATOM 339 CG ASP A 47 7.461 14.018 1.779 0.00 0.00 C

ATOM 340 OD1 ASP A 47 6.644 14.923 1.510 0.00 0.00 OA

ATOM 341 OD2 ASP A 47 7.252 12.811 1.539 0.00 0.00 OA1-

ATOM 342 N GLY A 48 8.459 16.431 5.552 0.00 0.00 N

ATOM 343 CA GLY A 48 8.025 17.705 6.104 0.00 0.00 C

ATOM 344 C GLY A 48 8.401 17.864 7.557 0.00 0.00 C

ATOM 345 O GLY A 48 9.527 17.548 7.955 0.00 0.00 OA

ATOM 346 N LYS A 49 7.449 18.371 8.352 0.00 0.00 N

ATOM 347 CA LYS A 49 7.578 18.594 9.795 0.00 0.00 C

ATOM 348 C LYS A 49 6.695 17.570 10.558 0.00 0.00 C

ATOM 349 O LYS A 49 5.936 16.854 9.890 0.00 0.00 OA

ATOM 350 CB LYS A 49 7.218 20.054 10.137 0.00 0.00 C

ATOM 351 CG LYS A 49 8.377 21.018 9.908 0.00 0.00 C

ATOM 352 CD LYS A 49 8.099 22.404 10.462 0.00 0.00 C

ATOM 353 CE LYS A 49 9.290 23.315 10.295 0.00 0.00 C

ATOM 354 NZ LYS A 49 9.033 24.669 10.853 0.00 0.00 N1+

ATOM 355 N PRO A 50 6.796 17.424 11.914 0.00 0.00 N

ATOM 356 CA PRO A 50 5.971 16.404 12.604 0.00 0.00 C

ATOM 357 C PRO A 50 4.458 16.556 12.439 0.00 0.00 C

ATOM 358 O PRO A 50 3.924 17.673 12.403 0.00 0.00 OA

ATOM 359 CB PRO A 50 6.392 16.516 14.077 0.00 0.00 C

ATOM 360 CG PRO A 50 7.702 17.223 14.060 0.00 0.00 C

ATOM 361 CD PRO A 50 7.664 18.141 12.875 0.00 0.00 C

ATOM 362 N VAL A 51 3.777 15.415 12.316 0.00 0.00 N

ATOM 363 CA VAL A 51 2.329 15.343 12.160 0.00 0.00 C

ATOM 364 C VAL A 51 1.804 14.286 13.138 0.00 0.00 C

ATOM 365 O VAL A 51 2.350 13.185 13.189 0.00 0.00 OA

ATOM 366 CB VAL A 51 1.945 15.026 10.684 0.00 0.00 C

ATOM 367 CG1 VAL A 51 0.535 14.464 10.565 0.00 0.00 C

ATOM 368 CG2 VAL A 51 2.124 16.244 9.783 0.00 0.00 C

ATOM 369 N ASN A 52 0.765 14.625 13.922 0.00 0.00 N

ATOM 370 CA ASN A 52 0.142 13.676 14.837 0.00 0.00 C

ATOM 371 C ASN A 52 -1.114 13.082 14.193 0.00 0.00 C

ATOM 372 O ASN A 52 -2.078 13.796 13.900 0.00 0.00 OA

ATOM 373 CB ASN A 52 -0.165 14.292 16.200 0.00 0.00 C

ATOM 374 CG ASN A 52 -0.652 13.262 17.178 0.00 0.00 C

ATOM 375 OD1 ASN A 52 -1.852 13.112 17.397 0.00 0.00 OA

ATOM 376 ND2 ASN A 52 0.260 12.451 17.692 0.00 0.00 N

ATOM 377 N LEU A 53 -1.080 11.774 13.957 0.00 0.00 N

ATOM 378 CA LEU A 53 -2.176 11.056 13.331 0.00 0.00 C

ATOM 379 C LEU A 53 -3.036 10.322 14.364 0.00 0.00 C

ATOM 380 O LEU A 53 -2.641 9.278 14.881 0.00 0.00 OA

ATOM 381 CB LEU A 53 -1.621 10.098 12.244 0.00 0.00 C

ATOM 382 CG LEU A 53 -2.608 9.242 11.424 0.00 0.00 C

ATOM 383 CD1 LEU A 53 -3.575 10.104 10.603 0.00 0.00 C

ATOM 384 CD2 LEU A 53 -1.860 8.315 10.491 0.00 0.00 C

ATOM 385 N GLY A 54 -4.199 10.888 14.647 0.00 0.00 N

ATOM 386 CA GLY A 54 -5.206 10.295 15.515 0.00 0.00 C

ATOM 387 C GLY A 54 -5.941 9.234 14.720 0.00 0.00 C

ATOM 388 O GLY A 54 -6.505 9.528 13.662 0.00 0.00 OA

ATOM 389 N LEU A 55 -5.866 7.980 15.176 0.00 0.00 N

ATOM 390 CA LEU A 55 -6.452 6.820 14.494 0.00 0.00 C

ATOM 391 C LEU A 55 -7.630 6.328 15.269 0.00 0.00 C

ATOM 392 O LEU A 55 -7.501 6.036 16.448 0.00 0.00 OA

ATOM 393 CB LEU A 55 -5.401 5.714 14.348 0.00 0.00 C

ATOM 394 CG LEU A 55 -4.180 6.100 13.521 0.00 0.00 C

ATOM 395 CD1 LEU A 55 -2.931 5.446 14.025 0.00 0.00 C

ATOM 396 CD2 LEU A 55 -4.408 5.851 12.079 0.00 0.00 C

ATOM 397 N TRP A 56 -8.793 6.275 14.631 0.00 0.00 N

ATOM 398 CA TRP A 56 -10.020 5.880 15.310 0.00 0.00 C

ATOM 399 C TRP A 56 -10.575 4.562 14.831 0.00 0.00 C

ATOM 400 O TRP A 56 -10.865 4.403 13.652 0.00 0.00 OA

ATOM 401 CB TRP A 56 -11.054 7.007 15.217 0.00 0.00 C

ATOM 402 CG TRP A 56 -10.617 8.238 15.956 0.00 0.00 A

ATOM 403 CD1 TRP A 56 -9.716 9.174 15.538 0.00 0.00 A

ATOM 404 CD2 TRP A 56 -11.007 8.619 17.278 0.00 0.00 A

ATOM 405 NE1 TRP A 56 -9.528 10.119 16.516 0.00 0.00 N

ATOM 406 CE2 TRP A 56 -10.321 9.812 17.591 0.00 0.00 A

ATOM 407 CE3 TRP A 56 -11.880 8.066 18.236 0.00 0.00 A

ATOM 408 CZ2 TRP A 56 -10.464 10.454 18.828 0.00 0.00 A

ATOM 409 CZ3 TRP A 56 -12.026 8.707 19.459 0.00 0.00 A

ATOM 410 CH2 TRP A 56 -11.329 9.889 19.743 0.00 0.00 A

ATOM 411 N ASP A 57 -10.675 3.601 15.741 0.00 0.00 N

ATOM 412 CA ASP A 57 -11.258 2.304 15.444 0.00 0.00 C

ATOM 413 C ASP A 57 -12.745 2.374 15.685 0.00 0.00 C

ATOM 414 O ASP A 57 -13.219 3.148 16.519 0.00 0.00 OA

ATOM 415 CB ASP A 57 -10.671 1.209 16.329 0.00 0.00 C

ATOM 416 CG ASP A 57 -10.842 -0.185 15.756 0.00 0.00 C

ATOM 417 OD1 ASP A 57 -11.251 -0.297 14.573 0.00 0.00 OA

ATOM 418 OD2 ASP A 57 -10.562 -1.165 16.486 0.00 0.00 OA1-

ATOM 419 N THR A 58 -13.489 1.562 14.952 0.00 0.00 N

ATOM 420 CA THR A 58 -14.932 1.525 15.070 0.00 0.00 C

ATOM 421 C THR A 58 -15.424 0.125 15.317 0.00 0.00 C

ATOM 422 O THR A 58 -14.744 -0.875 15.051 0.00 0.00 OA

ATOM 423 CB THR A 58 -15.615 2.084 13.811 0.00 0.00 C

ATOM 424 OG1 THR A 58 -15.179 1.336 12.682 0.00 0.00 OA

ATOM 425 CG2 THR A 58 -15.378 3.578 13.604 0.00 0.00 C

ATOM 426 N ALA A 59 -16.648 0.082 15.819 0.00 0.00 N

ATOM 427 CA ALA A 59 -17.432 -1.094 16.072 0.00 0.00 C

ATOM 428 C ALA A 59 -18.210 -1.297 14.753 0.00 0.00 C

ATOM 429 O ALA A 59 -19.104 -0.511 14.418 0.00 0.00 OA

ATOM 430 CB ALA A 59 -18.349 -0.825 17.253 0.00 0.00 C

ATOM 431 N GLY A 60 -17.749 -2.277 13.974 0.00 0.00 N

ATOM 432 CA GLY A 60 -18.240 -2.591 12.634 0.00 0.00 C

ATOM 433 C GLY A 60 -19.541 -3.352 12.500 0.00 0.00 C

ATOM 434 O GLY A 60 -20.196 -3.241 11.457 0.00 0.00 OA

ATOM 435 N GLN A 61 -19.927 -4.135 13.541 0.00 0.00 N

ATOM 436 CA GLN A 61 -21.155 -4.956 13.574 0.00 0.00 C

ATOM 437 C GLN A 61 -22.465 -4.135 13.327 0.00 0.00 C

ATOM 438 O GLN A 61 -22.484 -2.921 13.541 0.00 0.00 OA

ATOM 439 CB GLN A 61 -21.225 -5.773 14.885 0.00 0.00 C

ATOM 440 CG GLN A 61 -22.185 -6.969 14.881 0.00 0.00 C

ATOM 441 CD GLN A 61 -22.572 -7.459 16.267 0.00 0.00 C

ATOM 442 OE1 GLN A 61 -22.734 -6.685 17.219 0.00 0.00 OA

ATOM 443 NE2 GLN A 61 -22.797 -8.760 16.396 0.00 0.00 N

ATOM 444 N GLU A 62 -23.537 -4.818 12.854 0.00 0.00 N

ATOM 445 CA GLU A 62 -24.870 -4.269 12.565 0.00 0.00 C

ATOM 446 C GLU A 62 -25.474 -3.532 13.750 0.00 0.00 C

ATOM 447 O GLU A 62 -26.170 -2.547 13.546 0.00 0.00 OA

ATOM 448 CB GLU A 62 -25.828 -5.404 12.161 0.00 0.00 C

ATOM 449 CG GLU A 62 -26.116 -5.487 10.671 0.00 0.00 C

ATOM 450 CD GLU A 62 -26.849 -6.749 10.249 0.00 0.00 C

ATOM 451 OE1 GLU A 62 -26.223 -7.597 9.573 0.00 0.00 OA

ATOM 452 OE2 GLU A 62 -28.042 -6.898 10.602 0.00 0.00 OA1-

ATOM 453 N ASP A 63 -25.223 -4.020 14.980 0.00 0.00 N

ATOM 454 CA ASP A 63 -25.732 -3.448 16.233 0.00 0.00 C

ATOM 455 C ASP A 63 -25.321 -1.988 16.507 0.00 0.00 C

ATOM 456 O ASP A 63 -26.037 -1.271 17.222 0.00 0.00 OA

ATOM 457 CB ASP A 63 -25.340 -4.339 17.422 0.00 0.00 C

ATOM 458 CG ASP A 63 -26.049 -5.679 17.472 0.00 0.00 C

ATOM 459 OD1 ASP A 63 -26.965 -5.901 16.644 0.00 0.00 OA

ATOM 460 OD2 ASP A 63 -25.711 -6.498 18.361 0.00 0.00 OA1-

ATOM 461 N TYR A 64 -24.182 -1.558 15.936 0.00 0.00 N

ATOM 462 CA TYR A 64 -23.634 -0.219 16.123 0.00 0.00 C

ATOM 463 C TYR A 64 -23.913 0.731 14.976 0.00 0.00 C

ATOM 464 O TYR A 64 -23.342 1.823 14.966 0.00 0.00 OA

ATOM 465 CB TYR A 64 -22.131 -0.279 16.425 0.00 0.00 C

ATOM 466 CG TYR A 64 -21.737 -1.528 17.171 0.00 0.00 A

ATOM 467 CD1 TYR A 64 -22.080 -1.702 18.507 0.00 0.00 A

ATOM 468 CD2 TYR A 64 -21.072 -2.567 16.527 0.00 0.00 A

ATOM 469 CE1 TYR A 64 -21.787 -2.888 19.177 0.00 0.00 A

ATOM 470 CE2 TYR A 64 -20.700 -3.721 17.212 0.00 0.00 A

ATOM 471 CZ TYR A 64 -21.075 -3.887 18.533 0.00 0.00 A

ATOM 472 OH TYR A 64 -20.740 -5.045 19.193 0.00 0.00 OA

ATOM 473 N ASP A 65 -24.825 0.360 14.047 0.00 0.00 N

ATOM 474 CA ASP A 65 -25.179 1.180 12.882 0.00 0.00 C

ATOM 475 C ASP A 65 -25.641 2.613 13.230 0.00 0.00 C

ATOM 476 O ASP A 65 -25.360 3.539 12.464 0.00 0.00 OA

ATOM 477 CB ASP A 65 -26.214 0.472 11.987 0.00 0.00 C

ATOM 478 CG ASP A 65 -25.664 -0.649 11.103 0.00 0.00 C

ATOM 479 OD1 ASP A 65 -24.524 -1.100 11.350 0.00 0.00 OA

ATOM 480 OD2 ASP A 65 -26.402 -1.117 10.203 0.00 0.00 OA1-

ATOM 481 N ARG A 66 -26.313 2.799 14.380 0.00 0.00 N

ATOM 482 CA ARG A 66 -26.810 4.114 14.814 0.00 0.00 C

ATOM 483 C ARG A 66 -25.774 4.926 15.608 0.00 0.00 C

ATOM 484 O ARG A 66 -25.769 6.155 15.529 0.00 0.00 OA

ATOM 485 CB ARG A 66 -28.106 3.968 15.641 0.00 0.00 C

ATOM 486 CG ARG A 66 -29.273 3.290 14.927 0.00 0.00 C

ATOM 487 CD ARG A 66 -30.132 4.277 14.164 0.00 0.00 C

ATOM 488 NE ARG A 66 -31.173 3.608 13.379 0.00 0.00 N

ATOM 489 CZ ARG A 66 -31.007 3.141 12.143 0.00 0.00 C

ATOM 490 NH1 ARG A 66 -29.828 3.244 11.537 0.00 0.00 N1+

ATOM 491 NH2 ARG A 66 -32.015 2.558 11.507 0.00 0.00 N

ATOM 492 N LEU A 67 -24.901 4.246 16.364 0.00 0.00 N

ATOM 493 CA LEU A 67 -23.926 4.904 17.225 0.00 0.00 C

ATOM 494 C LEU A 67 -22.566 5.136 16.603 0.00 0.00 C

ATOM 495 O LEU A 67 -21.854 6.035 17.055 0.00 0.00 OA

ATOM 496 CB LEU A 67 -23.776 4.143 18.554 0.00 0.00 C

ATOM 497 CG LEU A 67 -25.040 3.986 19.421 0.00 0.00 C

ATOM 498 CD1 LEU A 67 -24.742 3.180 20.625 0.00 0.00 C

ATOM 499 CD2 LEU A 67 -25.615 5.325 19.828 0.00 0.00 C

ATOM 500 N ARG A 68 -22.174 4.310 15.618 0.00 0.00 N

ATOM 501 CA ARG A 68 -20.888 4.407 14.914 0.00 0.00 C

ATOM 502 C ARG A 68 -20.669 5.804 14.276 0.00 0.00 C

ATOM 503 O ARG A 68 -19.603 6.378 14.518 0.00 0.00 OA

ATOM 504 CB ARG A 68 -20.774 3.297 13.859 0.00 0.00 C

ATOM 505 CG ARG A 68 -19.436 3.169 13.159 0.00 0.00 C

ATOM 506 CD ARG A 68 -19.544 2.205 11.990 0.00 0.00 C

ATOM 507 NE ARG A 68 -20.246 0.965 12.344 0.00 0.00 N

ATOM 508 CZ ARG A 68 -21.341 0.519 11.738 0.00 0.00 C

ATOM 509 NH1 ARG A 68 -21.868 1.192 10.717 0.00 0.00 N1+

ATOM 510 NH2 ARG A 68 -21.913 -0.608 12.137 0.00 0.00 N

ATOM 511 N PRO A 69 -21.634 6.408 13.521 0.00 0.00 N

ATOM 512 CA PRO A 69 -21.361 7.715 12.913 0.00 0.00 C

ATOM 513 C PRO A 69 -21.123 8.869 13.890 0.00 0.00 C

ATOM 514 O PRO A 69 -20.705 9.937 13.440 0.00 0.00 OA

ATOM 515 CB PRO A 69 -22.578 7.940 12.016 0.00 0.00 C

ATOM 516 CG PRO A 69 -23.079 6.567 11.729 0.00 0.00 C

ATOM 517 CD PRO A 69 -22.946 5.911 13.059 0.00 0.00 C

ATOM 518 N LEU A 70 -21.311 8.644 15.217 0.00 0.00 N

ATOM 519 CA LEU A 70 -21.053 9.666 16.234 0.00 0.00 C

ATOM 520 C LEU A 70 -19.550 10.010 16.349 0.00 0.00 C

ATOM 521 O LEU A 70 -19.207 11.123 16.781 0.00 0.00 OA

ATOM 522 CB LEU A 70 -21.633 9.272 17.601 0.00 0.00 C

ATOM 523 CG LEU A 70 -23.151 9.241 17.711 0.00 0.00 C

ATOM 524 CD1 LEU A 70 -23.573 8.655 19.030 0.00 0.00 C

ATOM 525 CD2 LEU A 70 -23.751 10.616 17.498 0.00 0.00 C

ATOM 526 N SER A 71 -18.664 9.059 15.927 0.00 0.00 N

ATOM 527 CA SER A 71 -17.206 9.231 15.911 0.00 0.00 C

ATOM 528 C SER A 71 -16.716 9.898 14.612 0.00 0.00 C

ATOM 529 O SER A 71 -15.572 10.348 14.551 0.00 0.00 OA

ATOM 530 CB SER A 71 -16.490 7.902 16.140 0.00 0.00 C

ATOM 531 OG SER A 71 -17.143 6.791 15.544 0.00 0.00 OA

ATOM 532 N TYR A 72 -17.587 9.980 13.585 0.00 0.00 N

ATOM 533 CA TYR A 72 -17.245 10.576 12.294 0.00 0.00 C

ATOM 534 C TYR A 72 -17.009 12.102 12.344 0.00 0.00 C

ATOM 535 O TYR A 72 -16.028 12.528 11.746 0.00 0.00 OA

ATOM 536 CB TYR A 72 -18.267 10.222 11.190 0.00 0.00 C

ATOM 537 CG TYR A 72 -18.449 8.747 10.861 0.00 0.00 A

ATOM 538 CD1 TYR A 72 -17.593 7.775 11.382 0.00 0.00 A

ATOM 539 CD2 TYR A 72 -19.461 8.328 10.006 0.00 0.00 A

ATOM 540 CE1 TYR A 72 -17.770 6.423 11.089 0.00 0.00 A

ATOM 541 CE2 TYR A 72 -19.643 6.983 9.700 0.00 0.00 A

ATOM 542 CZ TYR A 72 -18.797 6.033 10.240 0.00 0.00 A

ATOM 543 OH TYR A 72 -19.010 4.713 9.919 0.00 0.00 OA

ATOM 544 N PRO A 73 -17.809 12.957 13.034 0.00 0.00 N

ATOM 545 CA PRO A 73 -17.529 14.407 12.992 0.00 0.00 C

ATOM 546 C PRO A 73 -16.085 14.827 13.270 0.00 0.00 C

ATOM 547 O PRO A 73 -15.447 14.276 14.167 0.00 0.00 OA

ATOM 548 CB PRO A 73 -18.499 14.969 14.021 0.00 0.00 C

ATOM 549 CG PRO A 73 -19.664 14.053 13.917 0.00 0.00 C

ATOM 550 CD PRO A 73 -19.047 12.695 13.799 0.00 0.00 C

ATOM 551 N GLN A 74 -15.576 15.801 12.461 0.00 0.00 N

ATOM 552 CA GLN A 74 -14.221 16.397 12.486 0.00 0.00 C

ATOM 553 C GLN A 74 -13.150 15.505 11.823 0.00 0.00 C

ATOM 554 O GLN A 74 -11.957 15.822 11.897 0.00 0.00 OA

ATOM 555 CB GLN A 74 -13.784 16.826 13.912 0.00 0.00 C

ATOM 556 CG GLN A 74 -14.682 17.875 14.578 0.00 0.00 C

ATOM 557 CD GLN A 74 -14.362 18.043 16.048 0.00 0.00 C

ATOM 558 OE1 GLN A 74 -14.514 17.118 16.857 0.00 0.00 OA

ATOM 559 NE2 GLN A 74 -13.940 19.239 16.437 0.00 0.00 N

ATOM 560 N THR A 75 -13.569 14.402 11.170 0.00 0.00 N

ATOM 561 CA THR A 75 -12.645 13.464 10.519 0.00 0.00 C

ATOM 562 C THR A 75 -11.996 14.085 9.278 0.00 0.00 C

ATOM 563 O THR A 75 -12.664 14.735 8.470 0.00 0.00 OA

ATOM 564 CB THR A 75 -13.295 12.090 10.240 0.00 0.00 C

ATOM 565 OG1 THR A 75 -12.387 11.269 9.500 0.00 0.00 OA

ATOM 566 CG2 THR A 75 -14.568 12.203 9.455 0.00 0.00 C

ATOM 567 N ASP A 76 -10.691 13.860 9.138 0.00 0.00 N

ATOM 568 CA ASP A 76 -9.897 14.376 8.039 0.00 0.00 C

ATOM 569 C ASP A 76 -9.817 13.402 6.864 0.00 0.00 C

ATOM 570 O ASP A 76 -9.770 13.855 5.718 0.00 0.00 OA

ATOM 571 CB ASP A 76 -8.504 14.775 8.546 0.00 0.00 C

ATOM 572 CG ASP A 76 -8.552 15.893 9.572 0.00 0.00 C

ATOM 573 OD1 ASP A 76 -8.885 17.044 9.185 0.00 0.00 OA

ATOM 574 OD2 ASP A 76 -8.295 15.615 10.773 0.00 0.00 OA1-

ATOM 575 N VAL A 77 -9.794 12.072 7.148 0.00 0.00 N

ATOM 576 CA VAL A 77 -9.682 10.986 6.155 0.00 0.00 C

ATOM 577 C VAL A 77 -10.280 9.666 6.687 0.00 0.00 C

ATOM 578 O VAL A 77 -10.262 9.426 7.886 0.00 0.00 OA

ATOM 579 CB VAL A 77 -8.207 10.831 5.648 0.00 0.00 C

ATOM 580 CG1 VAL A 77 -7.255 10.365 6.758 0.00 0.00 C

ATOM 581 CG2 VAL A 77 -8.108 9.924 4.423 0.00 0.00 C

ATOM 582 N PHE A 78 -10.797 8.824 5.795 0.00 0.00 N

ATOM 583 CA PHE A 78 -11.381 7.516 6.118 0.00 0.00 C

ATOM 584 C PHE A 78 -10.591 6.363 5.516 0.00 0.00 C

ATOM 585 O PHE A 78 -10.160 6.458 4.362 0.00 0.00 OA

ATOM 586 CB PHE A 78 -12.822 7.411 5.561 0.00 0.00 C

ATOM 587 CG PHE A 78 -13.899 7.974 6.448 0.00 0.00 A

ATOM 588 CD1 PHE A 78 -14.237 9.321 6.388 0.00 0.00 A

ATOM 589 CD2 PHE A 78 -14.569 7.163 7.355 0.00 0.00 A

ATOM 590 CE1 PHE A 78 -15.224 9.846 7.217 0.00 0.00 A

ATOM 591 CE2 PHE A 78 -15.544 7.695 8.200 0.00 0.00 A

ATOM 592 CZ PHE A 78 -15.860 9.035 8.132 0.00 0.00 A

ATOM 593 N LEU A 79 -10.492 5.245 6.256 0.00 0.00 N

ATOM 594 CA LEU A 79 -9.926 3.996 5.754 0.00 0.00 C

ATOM 595 C LEU A 79 -11.052 2.986 5.732 0.00 0.00 C

ATOM 596 O LEU A 79 -11.486 2.507 6.779 0.00 0.00 OA

ATOM 597 CB LEU A 79 -8.774 3.435 6.602 0.00 0.00 C

ATOM 598 CG LEU A 79 -7.491 4.235 6.739 0.00 0.00 C

ATOM 599 CD1 LEU A 79 -6.481 3.450 7.525 0.00 0.00 C

ATOM 600 CD2 LEU A 79 -6.907 4.642 5.387 0.00 0.00 C

ATOM 601 N ILE A 80 -11.571 2.710 4.549 0.00 0.00 N

ATOM 602 CA ILE A 80 -12.612 1.712 4.393 0.00 0.00 C

ATOM 603 C ILE A 80 -11.841 0.414 4.227 0.00 0.00 C

ATOM 604 O ILE A 80 -11.003 0.301 3.333 0.00 0.00 OA

ATOM 605 CB ILE A 80 -13.595 2.027 3.221 0.00 0.00 C

ATOM 606 CG1 ILE A 80 -14.298 3.411 3.452 0.00 0.00 C

ATOM 607 CG2 ILE A 80 -14.609 0.859 3.033 0.00 0.00 C

ATOM 608 CD1 ILE A 80 -15.478 3.756 2.550 0.00 0.00 C

ATOM 609 N CYS A 81 -12.069 -0.532 5.137 0.00 0.00 N

ATOM 610 CA CYS A 81 -11.339 -1.787 5.129 0.00 0.00 C

ATOM 611 C CYS A 81 -12.183 -2.978 4.767 0.00 0.00 C

ATOM 612 O CYS A 81 -13.375 -3.019 5.092 0.00 0.00 OA

ATOM 613 CB CYS A 81 -10.621 -1.996 6.455 0.00 0.00 C

ATOM 614 SG CYS A 81 -9.472 -0.666 6.865 0.00 0.00 SA

ATOM 615 N PHE A 82 -11.547 -3.953 4.084 0.00 0.00 N

ATOM 616 CA PHE A 82 -12.159 -5.222 3.697 0.00 0.00 C

ATOM 617 C PHE A 82 -11.106 -6.317 3.663 0.00 0.00 C

ATOM 618 O PHE A 82 -9.943 -6.061 3.320 0.00 0.00 OA

ATOM 619 CB PHE A 82 -12.910 -5.119 2.348 0.00 0.00 C

ATOM 620 CG PHE A 82 -12.044 -5.008 1.117 0.00 0.00 A

ATOM 621 CD1 PHE A 82 -11.594 -6.147 0.456 0.00 0.00 A

ATOM 622 CD2 PHE A 82 -11.684 -3.769 0.612 0.00 0.00 A

ATOM 623 CE1 PHE A 82 -10.767 -6.042 -0.669 0.00 0.00 A

ATOM 624 CE2 PHE A 82 -10.863 -3.667 -0.520 0.00 0.00 A

ATOM 625 CZ PHE A 82 -10.421 -4.804 -1.156 0.00 0.00 A

ATOM 626 N SER A 83 -11.512 -7.537 4.010 0.00 0.00 N

ATOM 627 CA SER A 83 -10.603 -8.660 3.962 0.00 0.00 C

ATOM 628 C SER A 83 -10.449 -9.095 2.509 0.00 0.00 C

ATOM 629 O SER A 83 -11.449 -9.331 1.817 0.00 0.00 OA

ATOM 630 CB SER A 83 -11.114 -9.804 4.813 0.00 0.00 C

ATOM 631 OG SER A 83 -10.217 -10.897 4.718 0.00 0.00 OA

ATOM 632 N LEU A 84 -9.189 -9.177 2.034 0.00 0.00 N

ATOM 633 CA LEU A 84 -8.907 -9.615 0.666 0.00 0.00 C

ATOM 634 C LEU A 84 -9.328 -11.070 0.469 0.00 0.00 C

ATOM 635 O LEU A 84 -9.573 -11.486 -0.664 0.00 0.00 OA

ATOM 636 CB LEU A 84 -7.438 -9.386 0.283 0.00 0.00 C

ATOM 637 CG LEU A 84 -7.077 -7.943 -0.121 0.00 0.00 C

ATOM 638 CD1 LEU A 84 -5.590 -7.662 0.075 0.00 0.00 C

ATOM 639 CD2 LEU A 84 -7.511 -7.630 -1.553 0.00 0.00 C

ATOM 640 N VAL A 85 -9.508 -11.808 1.585 0.00 0.00 N

ATOM 641 CA VAL A 85 -9.950 -13.196 1.577 0.00 0.00 C

ATOM 642 C VAL A 85 -11.442 -13.315 1.997 0.00 0.00 C

ATOM 643 O VAL A 85 -11.942 -14.417 2.243 0.00 0.00 OA

ATOM 644 CB VAL A 85 -8.992 -14.120 2.368 0.00 0.00 C

ATOM 645 CG1 VAL A 85 -7.571 -13.995 1.835 0.00 0.00 C

ATOM 646 CG2 VAL A 85 -9.030 -13.856 3.871 0.00 0.00 C

ATOM 647 N SER A 86 -12.157 -12.169 2.025 0.00 0.00 N

ATOM 648 CA SER A 86 -13.588 -12.127 2.317 0.00 0.00 C

ATOM 649 C SER A 86 -14.386 -11.328 1.245 0.00 0.00 C

ATOM 650 O SER A 86 -14.446 -10.093 1.282 0.00 0.00 OA

ATOM 651 CB SER A 86 -13.867 -11.650 3.737 0.00 0.00 C

ATOM 652 OG SER A 86 -15.233 -11.330 3.946 0.00 0.00 OA

ATOM 653 N PRO A 87 -15.042 -12.032 0.293 0.00 0.00 N

ATOM 654 CA PRO A 87 -15.850 -11.320 -0.713 0.00 0.00 C

ATOM 655 C PRO A 87 -17.065 -10.637 -0.090 0.00 0.00 C

ATOM 656 O PRO A 87 -17.445 -9.563 -0.559 0.00 0.00 OA

ATOM 657 CB PRO A 87 -16.219 -12.420 -1.725 0.00 0.00 C

ATOM 658 CG PRO A 87 -15.273 -13.572 -1.410 0.00 0.00 C

ATOM 659 CD PRO A 87 -15.091 -13.488 0.070 0.00 0.00 C

ATOM 660 N ALA A 88 -17.630 -11.221 0.999 0.00 0.00 N

ATOM 661 CA ALA A 88 -18.755 -10.635 1.750 0.00 0.00 C

ATOM 662 C ALA A 88 -18.387 -9.227 2.249 0.00 0.00 C

ATOM 663 O ALA A 88 -19.171 -8.297 2.051 0.00 0.00 OA

ATOM 664 CB ALA A 88 -19.146 -11.527 2.920 0.00 0.00 C

ATOM 665 N SER A 89 -17.158 -9.056 2.808 0.00 0.00 N

ATOM 666 CA SER A 89 -16.670 -7.755 3.287 0.00 0.00 C

ATOM 667 C SER A 89 -16.376 -6.762 2.161 0.00 0.00 C

ATOM 668 O SER A 89 -16.497 -5.552 2.368 0.00 0.00 OA

ATOM 669 CB SER A 89 -15.446 -7.921 4.178 0.00 0.00 C

ATOM 670 OG SER A 89 -14.346 -8.416 3.440 0.00 0.00 OA

ATOM 671 N PHE A 90 -15.988 -7.291 0.973 0.00 0.00 N

ATOM 672 CA PHE A 90 -15.686 -6.517 -0.224 0.00 0.00 C

ATOM 673 C PHE A 90 -16.963 -5.937 -0.820 0.00 0.00 C

ATOM 674 O PHE A 90 -16.965 -4.767 -1.191 0.00 0.00 OA

ATOM 675 CB PHE A 90 -14.896 -7.354 -1.258 0.00 0.00 C

ATOM 676 CG PHE A 90 -14.565 -6.607 -2.539 0.00 0.00 A

ATOM 677 CD1 PHE A 90 -13.645 -5.562 -2.538 0.00 0.00 A

ATOM 678 CD2 PHE A 90 -15.225 -6.900 -3.725 0.00 0.00 A

ATOM 679 CE1 PHE A 90 -13.367 -4.848 -3.708 0.00 0.00 A

ATOM 680 CE2 PHE A 90 -14.940 -6.193 -4.899 0.00 0.00 A

ATOM 681 CZ PHE A 90 -14.011 -5.173 -4.882 0.00 0.00 A

ATOM 682 N GLU A 91 -18.054 -6.730 -0.893 0.00 0.00 N

ATOM 683 CA GLU A 91 -19.359 -6.259 -1.409 0.00 0.00 C

ATOM 684 C GLU A 91 -19.945 -5.180 -0.497 0.00 0.00 C

ATOM 685 O GLU A 91 -20.619 -4.259 -0.972 0.00 0.00 OA

ATOM 686 CB GLU A 91 -20.359 -7.416 -1.554 0.00 0.00 C

ATOM 687 CG GLU A 91 -20.103 -8.278 -2.780 0.00 0.00 C

ATOM 688 CD GLU A 91 -20.817 -9.611 -2.746 0.00 0.00 C

ATOM 689 OE1 GLU A 91 -20.211 -10.593 -2.257 0.00 0.00 OA

ATOM 690 OE2 GLU A 91 -21.983 -9.675 -3.204 0.00 0.00 OA1-

ATOM 691 N ASN A 92 -19.637 -5.284 0.814 0.00 0.00 N

ATOM 692 CA ASN A 92 -20.064 -4.353 1.840 0.00 0.00 C

ATOM 693 C ASN A 92 -19.442 -2.970 1.701 0.00 0.00 C

ATOM 694 O ASN A 92 -19.955 -2.016 2.284 0.00 0.00 OA

ATOM 695 CB ASN A 92 -19.848 -4.938 3.212 0.00 0.00 C

ATOM 696 CG ASN A 92 -21.083 -5.619 3.727 0.00 0.00 C

ATOM 697 OD1 ASN A 92 -22.209 -5.157 3.529 0.00 0.00 OA

ATOM 698 ND2 ASN A 92 -20.909 -6.730 4.401 0.00 0.00 N

ATOM 699 N VAL A 93 -18.403 -2.842 0.862 0.00 0.00 N

ATOM 700 CA VAL A 93 -17.770 -1.563 0.577 0.00 0.00 C

ATOM 701 C VAL A 93 -18.800 -0.676 -0.107 0.00 0.00 C

ATOM 702 O VAL A 93 -19.130 0.375 0.433 0.00 0.00 OA

ATOM 703 CB VAL A 93 -16.436 -1.706 -0.209 0.00 0.00 C

ATOM 704 CG1 VAL A 93 -15.888 -0.345 -0.641 0.00 0.00 C

ATOM 705 CG2 VAL A 93 -15.398 -2.462 0.619 0.00 0.00 C

ATOM 706 N ARG A 94 -19.370 -1.139 -1.236 0.00 0.00 N

ATOM 707 CA ARG A 94 -20.398 -0.404 -1.969 0.00 0.00 C

ATOM 708 C ARG A 94 -21.761 -0.509 -1.303 0.00 0.00 C

ATOM 709 O ARG A 94 -22.532 0.447 -1.378 0.00 0.00 OA

ATOM 710 CB ARG A 94 -20.473 -0.820 -3.457 0.00 0.00 C

ATOM 711 CG ARG A 94 -20.813 -2.292 -3.725 0.00 0.00 C

ATOM 712 CD ARG A 94 -20.833 -2.596 -5.210 0.00 0.00 C

ATOM 713 NE ARG A 94 -20.374 -3.960 -5.488 0.00 0.00 N

ATOM 714 CZ ARG A 94 -19.636 -4.305 -6.540 0.00 0.00 C

ATOM 715 NH1 ARG A 94 -19.250 -3.386 -7.419 0.00 0.00 N1+

ATOM 716 NH2 ARG A 94 -19.261 -5.567 -6.711 0.00 0.00 N

ATOM 717 N ALA A 95 -22.083 -1.663 -0.690 0.00 0.00 N

ATOM 718 CA ALA A 95 -23.387 -1.868 -0.053 0.00 0.00 C

ATOM 719 C ALA A 95 -23.601 -1.023 1.187 0.00 0.00 C

ATOM 720 O ALA A 95 -24.622 -0.346 1.292 0.00 0.00 OA

ATOM 721 CB ALA A 95 -23.593 -3.336 0.286 0.00 0.00 C

ATOM 722 N LYS A 96 -22.632 -1.050 2.115 0.00 0.00 N

ATOM 723 CA LYS A 96 -22.740 -0.397 3.406 0.00 0.00 C

ATOM 724 C LYS A 96 -21.793 0.793 3.651 0.00 0.00 C

ATOM 725 O LYS A 96 -22.259 1.888 3.980 0.00 0.00 OA

ATOM 726 CB LYS A 96 -22.548 -1.474 4.500 0.00 0.00 C

ATOM 727 CG LYS A 96 -22.728 -0.985 5.944 0.00 0.00 C

ATOM 728 CD LYS A 96 -22.527 -2.116 6.934 0.00 0.00 C

ATOM 729 CE LYS A 96 -22.873 -1.708 8.345 0.00 0.00 C

ATOM 730 NZ LYS A 96 -22.295 -2.647 9.348 0.00 0.00 N1+

ATOM 731 N TRP A 97 -20.484 0.557 3.561 0.00 0.00 N

ATOM 732 CA TRP A 97 -19.456 1.487 3.986 0.00 0.00 C

ATOM 733 C TRP A 97 -19.349 2.773 3.193 0.00 0.00 C

ATOM 734 O TRP A 97 -19.352 3.826 3.831 0.00 0.00 OA

ATOM 735 CB TRP A 97 -18.116 0.774 4.103 0.00 0.00 C

ATOM 736 CG TRP A 97 -18.226 -0.366 5.073 0.00 0.00 A

ATOM 737 CD1 TRP A 97 -18.135 -1.695 4.784 0.00 0.00 A

ATOM 738 CD2 TRP A 97 -18.650 -0.279 6.442 0.00 0.00 A

ATOM 739 NE1 TRP A 97 -18.461 -2.444 5.892 0.00 0.00 N

ATOM 740 CE2 TRP A 97 -18.788 -1.600 6.923 0.00 0.00 A

ATOM 741 CE3 TRP A 97 -18.929 0.793 7.315 0.00 0.00 A

ATOM 742 CZ2 TRP A 97 -19.158 -1.882 8.247 0.00 0.00 A

ATOM 743 CZ3 TRP A 97 -19.343 0.511 8.612 0.00 0.00 A

ATOM 744 CH2 TRP A 97 -19.440 -0.810 9.070 0.00 0.00 A

ATOM 745 N TYR A 98 -19.280 2.729 1.853 0.00 0.00 N

ATOM 746 CA TYR A 98 -19.203 3.959 1.070 0.00 0.00 C

ATOM 747 C TYR A 98 -20.514 4.785 1.197 0.00 0.00 C

ATOM 748 O TYR A 98 -20.398 5.960 1.523 0.00 0.00 OA

ATOM 749 CB TYR A 98 -18.771 3.685 -0.381 0.00 0.00 C

ATOM 750 CG TYR A 98 -19.069 4.809 -1.353 0.00 0.00 A

ATOM 751 CD1 TYR A 98 -18.313 5.982 -1.349 0.00 0.00 A

ATOM 752 CD2 TYR A 98 -20.094 4.693 -2.290 0.00 0.00 A

ATOM 753 CE1 TYR A 98 -18.558 7.003 -2.269 0.00 0.00 A

ATOM 754 CE2 TYR A 98 -20.363 5.718 -3.197 0.00 0.00 A

ATOM 755 CZ TYR A 98 -19.594 6.872 -3.179 0.00 0.00 A

ATOM 756 OH TYR A 98 -19.852 7.888 -4.060 0.00 0.00 OA

ATOM 757 N PRO A 99 -21.747 4.225 1.068 0.00 0.00 N

ATOM 758 CA PRO A 99 -22.944 5.065 1.299 0.00 0.00 C

ATOM 759 C PRO A 99 -22.985 5.724 2.684 0.00 0.00 C

ATOM 760 O PRO A 99 -23.262 6.926 2.753 0.00 0.00 OA

ATOM 761 CB PRO A 99 -24.127 4.111 1.080 0.00 0.00 C

ATOM 762 CG PRO A 99 -23.558 2.742 1.072 0.00 0.00 C

ATOM 763 CD PRO A 99 -22.124 2.847 0.683 0.00 0.00 C

ATOM 764 N GLU A 100 -22.652 4.970 3.773 0.00 0.00 N

ATOM 765 CA GLU A 100 -22.653 5.503 5.147 0.00 0.00 C

ATOM 766 C GLU A 100 -21.627 6.610 5.382 0.00 0.00 C

ATOM 767 O GLU A 100 -21.938 7.585 6.071 0.00 0.00 OA

ATOM 768 CB GLU A 100 -22.497 4.401 6.201 0.00 0.00 C

ATOM 769 CG GLU A 100 -22.906 4.877 7.589 0.00 0.00 C

ATOM 770 CD GLU A 100 -22.850 3.863 8.712 0.00 0.00 C

ATOM 771 OE1 GLU A 100 -23.878 3.682 9.408 0.00 0.00 OA

ATOM 772 OE2 GLU A 100 -21.753 3.313 8.949 0.00 0.00 OA1-

ATOM 773 N VAL A 101 -20.411 6.461 4.819 0.00 0.00 N

ATOM 774 CA VAL A 101 -19.348 7.459 4.953 0.00 0.00 C

ATOM 775 C VAL A 101 -19.784 8.744 4.239 0.00 0.00 C

ATOM 776 O VAL A 101 -19.733 9.819 4.844 0.00 0.00 OA

ATOM 777 CB VAL A 101 -17.947 6.926 4.510 0.00 0.00 C

ATOM 778 CG1 VAL A 101 -16.925 8.048 4.396 0.00 0.00 C

ATOM 779 CG2 VAL A 101 -17.431 5.858 5.470 0.00 0.00 C

ATOM 780 N ARG A 102 -20.284 8.620 2.993 0.00 0.00 N

ATOM 781 CA ARG A 102 -20.749 9.760 2.197 0.00 0.00 C

ATOM 782 C ARG A 102 -21.982 10.456 2.779 0.00 0.00 C

ATOM 783 O ARG A 102 -22.105 11.679 2.658 0.00 0.00 OA

ATOM 784 CB ARG A 102 -20.964 9.365 0.730 0.00 0.00 C

ATOM 785 CG ARG A 102 -19.649 9.203 -0.015 0.00 0.00 C

ATOM 786 CD ARG A 102 -18.939 10.520 -0.282 0.00 0.00 C

ATOM 787 NE ARG A 102 -17.536 10.312 -0.641 0.00 0.00 N

ATOM 788 CZ ARG A 102 -16.504 10.605 0.145 0.00 0.00 C

ATOM 789 NH1 ARG A 102 -16.703 11.137 1.346 0.00 0.00 N1+

ATOM 790 NH2 ARG A 102 -15.263 10.381 -0.269 0.00 0.00 N

ATOM 791 N HIS A 103 -22.862 9.687 3.445 0.00 0.00 N

ATOM 792 CA HIS A 103 -24.068 10.198 4.101 0.00 0.00 C

ATOM 793 C HIS A 103 -23.721 11.299 5.103 0.00 0.00 C

ATOM 794 O HIS A 103 -24.233 12.412 5.007 0.00 0.00 OA

ATOM 795 CB HIS A 103 -24.822 9.050 4.801 0.00 0.00 C

ATOM 796 CG HIS A 103 -26.193 9.424 5.259 0.00 0.00 A

ATOM 797 ND1 HIS A 103 -26.412 9.976 6.513 0.00 0.00 N

ATOM 798 CD2 HIS A 103 -27.378 9.324 4.612 0.00 0.00 A

ATOM 799 CE1 HIS A 103 -27.718 10.190 6.588 0.00 0.00 A

ATOM 800 NE2 HIS A 103 -28.342 9.813 5.468 0.00 0.00 NA

ATOM 801 N HIS A 104 -22.814 10.990 6.027 0.00 0.00 N

ATOM 802 CA HIS A 104 -22.389 11.881 7.103 0.00 0.00 C

ATOM 803 C HIS A 104 -21.257 12.839 6.699 0.00 0.00 C

ATOM 804 O HIS A 104 -21.220 13.974 7.182 0.00 0.00 OA

ATOM 805 CB HIS A 104 -22.030 11.048 8.343 0.00 0.00 C

ATOM 806 CG HIS A 104 -23.149 10.149 8.777 0.00 0.00 A

ATOM 807 ND1 HIS A 104 -23.243 8.843 8.325 0.00 0.00 N

ATOM 808 CD2 HIS A 104 -24.222 10.416 9.559 0.00 0.00 A

ATOM 809 CE1 HIS A 104 -24.352 8.353 8.861 0.00 0.00 A

ATOM 810 NE2 HIS A 104 -24.967 9.257 9.622 0.00 0.00 NA

ATOM 811 N CYS A 105 -20.351 12.391 5.811 0.00 0.00 N

ATOM 812 CA CYS A 105 -19.208 13.177 5.335 0.00 0.00 C

ATOM 813 C CYS A 105 -19.128 13.034 3.800 0.00 0.00 C

ATOM 814 O CYS A 105 -18.549 12.059 3.304 0.00 0.00 OA

ATOM 815 CB CYS A 105 -17.912 12.725 6.013 0.00 0.00 C

ATOM 816 SG CYS A 105 -17.926 12.835 7.822 0.00 0.00 SA

ATOM 817 N PRO A 106 -19.718 13.963 3.019 0.00 0.00 N

ATOM 818 CA PRO A 106 -19.724 13.784 1.556 0.00 0.00 C

ATOM 819 C PRO A 106 -18.472 14.257 0.818 0.00 0.00 C

ATOM 820 O PRO A 106 -18.293 13.892 -0.350 0.00 0.00 OA

ATOM 821 CB PRO A 106 -20.977 14.556 1.108 0.00 0.00 C

ATOM 822 CG PRO A 106 -21.577 15.171 2.379 0.00 0.00 C

ATOM 823 CD PRO A 106 -20.497 15.156 3.404 0.00 0.00 C

ATOM 824 N ASN A 107 -17.617 15.061 1.477 0.00 0.00 N

ATOM 825 CA ASN A 107 -16.427 15.613 0.837 0.00 0.00 C

ATOM 826 C ASN A 107 -15.093 15.063 1.376 0.00 0.00 C

ATOM 827 O ASN A 107 -14.046 15.293 0.755 0.00 0.00 OA

ATOM 828 CB ASN A 107 -16.459 17.149 0.903 0.00 0.00 C

ATOM 829 CG ASN A 107 -17.725 17.750 0.336 0.00 0.00 C

ATOM 830 OD1 ASN A 107 -18.613 18.198 1.074 0.00 0.00 OA

ATOM 831 ND2 ASN A 107 -17.857 17.733 -0.988 0.00 0.00 N

ATOM 832 N THR A 108 -15.136 14.341 2.515 0.00 0.00 N

ATOM 833 CA THR A 108 -13.978 13.750 3.193 0.00 0.00 C

ATOM 834 C THR A 108 -13.304 12.666 2.319 0.00 0.00 C

ATOM 835 O THR A 108 -14.008 11.811 1.783 0.00 0.00 OA

ATOM 836 CB THR A 108 -14.388 13.267 4.599 0.00 0.00 C

ATOM 837 OG1 THR A 108 -14.921 14.378 5.333 0.00 0.00 OA

ATOM 838 CG2 THR A 108 -13.232 12.634 5.381 0.00 0.00 C

ATOM 839 N PRO A 109 -11.956 12.698 2.150 0.00 0.00 N

ATOM 840 CA PRO A 109 -11.297 11.691 1.289 0.00 0.00 C

ATOM 841 C PRO A 109 -11.317 10.258 1.828 0.00 0.00 C

ATOM 842 O PRO A 109 -11.257 10.049 3.031 0.00 0.00 OA

ATOM 843 CB PRO A 109 -9.863 12.218 1.159 0.00 0.00 C

ATOM 844 CG PRO A 109 -9.650 13.041 2.393 0.00 0.00 C

ATOM 845 CD PRO A 109 -10.980 13.673 2.684 0.00 0.00 C

ATOM 846 N ILE A 110 -11.391 9.278 0.923 0.00 0.00 N

ATOM 847 CA ILE A 110 -11.395 7.859 1.268 0.00 0.00 C

ATOM 848 C ILE A 110 -10.193 7.143 0.655 0.00 0.00 C

ATOM 849 O ILE A 110 -9.869 7.368 -0.512 0.00 0.00 OA

ATOM 850 CB ILE A 110 -12.718 7.170 0.827 0.00 0.00 C

ATOM 851 CG1 ILE A 110 -13.929 7.701 1.616 0.00 0.00 C

ATOM 852 CG2 ILE A 110 -12.619 5.630 0.907 0.00 0.00 C

ATOM 853 CD1 ILE A 110 -15.334 7.246 1.034 0.00 0.00 C

ATOM 854 N ILE A 111 -9.560 6.257 1.439 0.00 0.00 N

ATOM 855 CA ILE A 111 -8.501 5.355 0.992 0.00 0.00 C

ATOM 856 C ILE A 111 -9.118 3.960 1.181 0.00 0.00 C

ATOM 857 O ILE A 111 -9.548 3.626 2.293 0.00 0.00 OA

ATOM 858 CB ILE A 111 -7.194 5.501 1.812 0.00 0.00 C

ATOM 859 CG1 ILE A 111 -6.675 6.973 1.876 0.00 0.00 C

ATOM 860 CG2 ILE A 111 -6.113 4.522 1.331 0.00 0.00 C

ATOM 861 CD1 ILE A 111 -6.065 7.607 0.617 0.00 0.00 C

ATOM 862 N LEU A 112 -9.250 3.186 0.090 0.00 0.00 N

ATOM 863 CA LEU A 112 -9.788 1.832 0.173 0.00 0.00 C

ATOM 864 C LEU A 112 -8.635 0.892 0.553 0.00 0.00 C

ATOM 865 O LEU A 112 -7.574 0.921 -0.085 0.00 0.00 OA

ATOM 866 CB LEU A 112 -10.471 1.417 -1.131 0.00 0.00 C

ATOM 867 CG LEU A 112 -11.110 0.006 -1.176 0.00 0.00 C

ATOM 868 CD1 LEU A 112 -12.143 -0.198 -0.051 0.00 0.00 C

ATOM 869 CD2 LEU A 112 -11.751 -0.251 -2.533 0.00 0.00 C

ATOM 870 N VAL A 113 -8.816 0.132 1.643 0.00 0.00 N

ATOM 871 CA VAL A 113 -7.791 -0.749 2.177 0.00 0.00 C

ATOM 872 C VAL A 113 -8.208 -2.226 2.198 0.00 0.00 C

ATOM 873 O VAL A 113 -9.131 -2.604 2.914 0.00 0.00 OA

ATOM 874 CB VAL A 113 -7.268 -0.288 3.582 0.00 0.00 C

ATOM 875 CG1 VAL A 113 -6.072 -1.133 4.028 0.00 0.00 C

ATOM 876 CG2 VAL A 113 -6.894 1.196 3.602 0.00 0.00 C

ATOM 877 N GLY A 114 -7.461 -3.043 1.468 0.00 0.00 N

ATOM 878 CA GLY A 114 -7.570 -4.496 1.484 0.00 0.00 C

ATOM 879 C GLY A 114 -6.571 -5.074 2.488 0.00 0.00 C

ATOM 880 O GLY A 114 -5.366 -4.823 2.410 0.00 0.00 OA

ATOM 881 N THR A 115 -7.075 -5.812 3.470 0.00 0.00 N

ATOM 882 CA THR A 115 -6.301 -6.429 4.552 0.00 0.00 C

ATOM 883 C THR A 115 -6.133 -7.947 4.326 0.00 0.00 C

ATOM 884 O THR A 115 -6.773 -8.510 3.436 0.00 0.00 OA

ATOM 885 CB THR A 115 -7.039 -6.193 5.881 0.00 0.00 C

ATOM 886 OG1 THR A 115 -8.368 -6.705 5.743 0.00 0.00 OA

ATOM 887 CG2 THR A 115 -7.079 -4.708 6.288 0.00 0.00 C

ATOM 888 N LYS A 116 -5.308 -8.603 5.174 0.00 0.00 N

ATOM 889 CA LYS A 116 -5.015 -10.041 5.147 0.00 0.00 C

ATOM 890 C LYS A 116 -4.424 -10.471 3.795 0.00 0.00 C

ATOM 891 O LYS A 116 -4.742 -11.539 3.272 0.00 0.00 OA

ATOM 892 CB LYS A 116 -6.233 -10.902 5.575 0.00 0.00 C

ATOM 893 CG LYS A 116 -6.968 -10.402 6.825 0.00 0.00 C

ATOM 894 CD LYS A 116 -7.890 -11.468 7.387 0.00 0.00 C

ATOM 895 CE LYS A 116 -8.848 -10.904 8.398 0.00 0.00 C

ATOM 896 NZ LYS A 116 -9.552 -11.984 9.148 0.00 0.00 N1+

ATOM 897 N LEU A 117 -3.532 -9.620 3.256 0.00 0.00 N

ATOM 898 CA LEU A 117 -2.799 -9.800 2.002 0.00 0.00 C

ATOM 899 C LEU A 117 -1.977 -11.080 2.044 0.00 0.00 C

ATOM 900 O LEU A 117 -1.832 -11.734 1.012 0.00 0.00 OA

ATOM 901 CB LEU A 117 -1.900 -8.575 1.770 0.00 0.00 C

ATOM 902 CG LEU A 117 -0.919 -8.549 0.597 0.00 0.00 C

ATOM 903 CD1 LEU A 117 -1.642 -8.435 -0.738 0.00 0.00 C

ATOM 904 CD2 LEU A 117 0.057 -7.391 0.748 0.00 0.00 C

ATOM 905 N ASP A 118 -1.485 -11.459 3.239 0.00 0.00 N

ATOM 906 CA ASP A 118 -0.707 -12.685 3.478 0.00 0.00 C

ATOM 907 C ASP A 118 -1.535 -13.971 3.248 0.00 0.00 C

ATOM 908 O ASP A 118 -0.967 -15.027 2.957 0.00 0.00 OA

ATOM 909 CB ASP A 118 -0.135 -12.681 4.906 0.00 0.00 C

ATOM 910 CG ASP A 118 -1.199 -12.706 5.980 0.00 0.00 C

ATOM 911 OD1 ASP A 118 -1.963 -11.720 6.085 0.00 0.00 OA

ATOM 912 OD2 ASP A 118 -1.302 -13.729 6.690 0.00 0.00 OA1-

ATOM 913 N LEU A 119 -2.860 -13.883 3.394 0.00 0.00 N

ATOM 914 CA LEU A 119 -3.722 -15.037 3.207 0.00 0.00 C

ATOM 915 C LEU A 119 -4.130 -15.269 1.755 0.00 0.00 C

ATOM 916 O LEU A 119 -4.625 -16.356 1.464 0.00 0.00 OA

ATOM 917 CB LEU A 119 -4.969 -14.958 4.107 0.00 0.00 C

ATOM 918 CG LEU A 119 -4.760 -15.137 5.596 0.00 0.00 C

ATOM 919 CD1 LEU A 119 -5.987 -14.712 6.355 0.00 0.00 C

ATOM 920 CD2 LEU A 119 -4.398 -16.575 5.947 0.00 0.00 C

ATOM 921 N ARG A 120 -3.922 -14.284 0.844 0.00 0.00 N

ATOM 922 CA ARG A 120 -4.326 -14.423 -0.566 0.00 0.00 C

ATOM 923 C ARG A 120 -3.635 -15.579 -1.316 0.00 0.00 C

ATOM 924 O ARG A 120 -4.229 -16.137 -2.235 0.00 0.00 OA

ATOM 925 CB ARG A 120 -4.184 -13.109 -1.344 0.00 0.00 C

ATOM 926 CG ARG A 120 -5.107 -13.028 -2.567 0.00 0.00 C

ATOM 927 CD ARG A 120 -5.313 -11.614 -3.072 0.00 0.00 C

ATOM 928 NE ARG A 120 -4.046 -10.939 -3.366 0.00 0.00 N

ATOM 929 CZ ARG A 120 -3.938 -9.666 -3.727 0.00 0.00 C

ATOM 930 NH1 ARG A 120 -5.020 -8.920 -3.885 0.00 0.00 N1+

ATOM 931 NH2 ARG A 120 -2.745 -9.136 -3.957 0.00 0.00 N

ATOM 932 N ASP A 121 -2.414 -15.948 -0.909 0.00 0.00 N

ATOM 933 CA ASP A 121 -1.658 -17.053 -1.509 0.00 0.00 C

ATOM 934 C ASP A 121 -1.713 -18.330 -0.639 0.00 0.00 C

ATOM 935 O ASP A 121 -1.088 -19.336 -0.998 0.00 0.00 OA

ATOM 936 CB ASP A 121 -0.198 -16.633 -1.796 0.00 0.00 C

ATOM 937 CG ASP A 121 0.011 -15.815 -3.068 0.00 0.00 C

ATOM 938 OD1 ASP A 121 -0.676 -16.102 -4.088 0.00 0.00 OA

ATOM 939 OD2 ASP A 121 0.911 -14.936 -3.070 0.00 0.00 OA1-

ATOM 940 N ASP A 122 -2.480 -18.295 0.481 0.00 0.00 N

ATOM 941 CA ASP A 122 -2.636 -19.418 1.421 0.00 0.00 C

ATOM 942 C ASP A 122 -3.574 -20.506 0.867 0.00 0.00 C

ATOM 943 O ASP A 122 -4.778 -20.273 0.744 0.00 0.00 OA

ATOM 944 CB ASP A 122 -3.106 -18.909 2.806 0.00 0.00 C

ATOM 945 CG ASP A 122 -3.106 -19.930 3.932 0.00 0.00 C

ATOM 946 OD1 ASP A 122 -2.228 -20.825 3.928 0.00 0.00 OA

ATOM 947 OD2 ASP A 122 -3.944 -19.797 4.853 0.00 0.00 OA1-

ATOM 948 N LYS A 123 -3.004 -21.687 0.529 0.00 0.00 N

ATOM 949 CA LYS A 123 -3.693 -22.859 -0.036 0.00 0.00 C

ATOM 950 C LYS A 123 -4.999 -23.231 0.718 0.00 0.00 C

ATOM 951 O LYS A 123 -6.031 -23.437 0.068 0.00 0.00 OA

ATOM 952 CB LYS A 123 -2.723 -24.058 -0.126 0.00 0.00 C

ATOM 953 CG LYS A 123 -3.313 -25.331 -0.749 0.00 0.00 C

ATOM 954 CD LYS A 123 -2.719 -26.619 -0.161 0.00 0.00 C

ATOM 955 CE LYS A 123 -3.224 -26.967 1.227 0.00 0.00 C

ATOM 956 NZ LYS A 123 -4.655 -27.384 1.231 0.00 0.00 N1+

ATOM 957 N ASP A 124 -4.952 -23.281 2.072 0.00 0.00 N

ATOM 958 CA ASP A 124 -6.105 -23.591 2.928 0.00 0.00 C

ATOM 959 C ASP A 124 -7.232 -22.560 2.776 0.00 0.00 C

ATOM 960 O ASP A 124 -8.402 -22.938 2.668 0.00 0.00 OA

ATOM 961 CB ASP A 124 -5.681 -23.716 4.405 0.00 0.00 C

ATOM 962 CG ASP A 124 -4.619 -24.774 4.654 0.00 0.00 C

ATOM 963 OD1 ASP A 124 -3.421 -24.468 4.464 0.00 0.00 OA

ATOM 964 OD2 ASP A 124 -4.988 -25.911 5.040 0.00 0.00 OA1-

ATOM 965 N THR A 125 -6.868 -21.264 2.744 0.00 0.00 N

ATOM 966 CA THR A 125 -7.791 -20.135 2.596 0.00 0.00 C

ATOM 967 C THR A 125 -8.437 -20.130 1.205 0.00 0.00 C

ATOM 968 O THR A 125 -9.620 -19.806 1.081 0.00 0.00 OA

ATOM 969 CB THR A 125 -7.079 -18.815 2.951 0.00 0.00 C

ATOM 970 OG1 THR A 125 -6.457 -18.940 4.235 0.00 0.00 OA

ATOM 971 CG2 THR A 125 -8.023 -17.620 2.960 0.00 0.00 C

ATOM 972 N ILE A 126 -7.659 -20.490 0.165 0.00 0.00 N

ATOM 973 CA ILE A 126 -8.138 -20.561 -1.222 0.00 0.00 C

ATOM 974 C ILE A 126 -9.129 -21.731 -1.364 0.00 0.00 C

ATOM 975 O ILE A 126 -10.181 -21.560 -1.988 0.00 0.00 OA

ATOM 976 CB ILE A 126 -6.965 -20.605 -2.251 0.00 0.00 C

ATOM 977 CG1 ILE A 126 -6.057 -19.357 -2.118 0.00 0.00 C

ATOM 978 CG2 ILE A 126 -7.487 -20.736 -3.693 0.00 0.00 C

ATOM 979 CD1 ILE A 126 -4.586 -19.569 -2.526 0.00 0.00 C

ATOM 980 N GLU A 127 -8.808 -22.892 -0.740 0.00 0.00 N

ATOM 981 CA GLU A 127 -9.647 -24.094 -0.741 0.00 0.00 C

ATOM 982 C GLU A 127 -10.990 -23.830 -0.074 0.00 0.00 C

ATOM 983 O GLU A 127 -12.018 -24.163 -0.667 0.00 0.00 OA

ATOM 984 CB GLU A 127 -8.930 -25.289 -0.084 0.00 0.00 C

ATOM 985 CG GLU A 127 -8.278 -26.247 -1.077 0.00 0.00 C

ATOM 986 CD GLU A 127 -9.185 -27.298 -1.697 0.00 0.00 C

ATOM 987 OE1 GLU A 127 -9.649 -28.199 -0.959 0.00 0.00 OA

ATOM 988 OE2 GLU A 127 -9.384 -27.256 -2.933 0.00 0.00 OA1-

ATOM 989 N LYS A 128 -10.991 -23.193 1.126 0.00 0.00 N

ATOM 990 CA LYS A 128 -12.215 -22.850 1.871 0.00 0.00 C

ATOM 991 C LYS A 128 -13.144 -21.945 1.050 0.00 0.00 C

ATOM 992 O LYS A 128 -14.360 -22.173 1.019 0.00 0.00 OA

ATOM 993 CB LYS A 128 -11.889 -22.208 3.232 0.00 0.00 C

ATOM 994 CG LYS A 128 -11.348 -23.193 4.271 0.00 0.00 C

ATOM 995 CD LYS A 128 -11.076 -22.549 5.642 0.00 0.00 C

ATOM 996 CE LYS A 128 -9.718 -21.887 5.772 0.00 0.00 C

ATOM 997 NZ LYS A 128 -9.804 -20.404 5.657 0.00 0.00 N1+

ATOM 998 N LEU A 129 -12.561 -20.939 0.370 0.00 0.00 N

ATOM 999 CA LEU A 129 -13.275 -19.996 -0.488 0.00 0.00 C

ATOM 1000 C LEU A 129 -13.886 -20.690 -1.714 0.00 0.00 C

ATOM 1001 O LEU A 129 -15.018 -20.369 -2.089 0.00 0.00 OA

ATOM 1002 CB LEU A 129 -12.336 -18.874 -0.962 0.00 0.00 C

ATOM 1003 CG LEU A 129 -12.182 -17.663 -0.068 0.00 0.00 C

ATOM 1004 CD1 LEU A 129 -10.982 -16.840 -0.496 0.00 0.00 C

ATOM 1005 CD2 LEU A 129 -13.420 -16.801 -0.097 0.00 0.00 C

ATOM 1006 N LYS A 130 -13.117 -21.614 -2.345 0.00 0.00 N

ATOM 1007 CA LYS A 130 -13.516 -22.364 -3.533 0.00 0.00 C

ATOM 1008 C LYS A 130 -14.728 -23.251 -3.261 0.00 0.00 C

ATOM 1009 O LYS A 130 -15.591 -23.364 -4.134 0.00 0.00 OA

ATOM 1010 CB LYS A 130 -12.337 -23.173 -4.102 0.00 0.00 C

ATOM 1011 CG LYS A 130 -12.536 -23.630 -5.554 0.00 0.00 C

ATOM 1012 CD LYS A 130 -11.580 -24.768 -5.980 0.00 0.00 C

ATOM 1013 CE LYS A 130 -11.624 -26.034 -5.132 0.00 0.00 C

ATOM 1014 NZ LYS A 130 -12.992 -26.619 -5.021 0.00 0.00 N1+

ATOM 1015 N GLU A 131 -14.816 -23.838 -2.042 0.00 0.00 N

ATOM 1016 CA GLU A 131 -15.953 -24.665 -1.610 0.00 0.00 C

ATOM 1017 C GLU A 131 -17.252 -23.834 -1.594 0.00 0.00 C

ATOM 1018 O GLU A 131 -18.312 -24.337 -1.973 0.00 0.00 OA

ATOM 1019 CB GLU A 131 -15.699 -25.311 -0.231 0.00 0.00 C

ATOM 1020 CG GLU A 131 -14.509 -26.265 -0.150 0.00 0.00 C

ATOM 1021 CD GLU A 131 -14.435 -27.449 -1.104 0.00 0.00 C

ATOM 1022 OE1 GLU A 131 -15.489 -28.054 -1.408 0.00 0.00 OA

ATOM 1023 OE2 GLU A 131 -13.303 -27.800 -1.511 0.00 0.00 OA1-

ATOM 1024 N LYS A 132 -17.149 -22.552 -1.213 0.00 0.00 N

ATOM 1025 CA LYS A 132 -18.264 -21.605 -1.181 0.00 0.00 C

ATOM 1026 C LYS A 132 -18.399 -20.858 -2.539 0.00 0.00 C

ATOM 1027 O LYS A 132 -19.099 -19.837 -2.620 0.00 0.00 OA

ATOM 1028 CB LYS A 132 -18.091 -20.636 0.001 0.00 0.00 C

ATOM 1029 CG LYS A 132 -18.121 -21.332 1.360 0.00 0.00 C

ATOM 1030 CD LYS A 132 -17.492 -20.513 2.469 0.00 0.00 C

ATOM 1031 CE LYS A 132 -17.308 -21.368 3.703 0.00 0.00 C

ATOM 1032 NZ LYS A 132 -16.763 -20.598 4.854 0.00 0.00 N1+

ATOM 1033 N LYS A 133 -17.735 -21.395 -3.607 0.00 0.00 N

ATOM 1034 CA LYS A 133 -17.704 -20.888 -4.998 0.00 0.00 C

ATOM 1035 C LYS A 133 -17.172 -19.440 -5.115 0.00 0.00 C

ATOM 1036 O LYS A 133 -17.499 -18.725 -6.069 0.00 0.00 OA

ATOM 1037 CB LYS A 133 -19.076 -21.052 -5.693 0.00 0.00 C

ATOM 1038 CG LYS A 133 -19.331 -22.464 -6.208 0.00 0.00 C

ATOM 1039 CD LYS A 133 -20.822 -22.792 -6.245 0.00 0.00 C

ATOM 1040 CE LYS A 133 -21.106 -24.145 -6.857 0.00 0.00 C

ATOM 1041 NZ LYS A 133 -20.756 -25.262 -5.945 0.00 0.00 N1+

ATOM 1042 N LEU A 134 -16.317 -19.036 -4.160 0.00 0.00 N

ATOM 1043 CA LEU A 134 -15.730 -17.696 -4.103 0.00 0.00 C

ATOM 1044 C LEU A 134 -14.220 -17.695 -4.306 0.00 0.00 C

ATOM 1045 O LEU A 134 -13.551 -18.690 -4.042 0.00 0.00 OA

ATOM 1046 CB LEU A 134 -16.111 -16.970 -2.794 0.00 0.00 C

ATOM 1047 CG LEU A 134 -17.574 -16.538 -2.654 0.00 0.00 C

ATOM 1048 CD1 LEU A 134 -17.931 -16.281 -1.202 0.00 0.00 C

ATOM 1049 CD2 LEU A 134 -17.892 -15.320 -3.514 0.00 0.00 C

ATOM 1050 N THR A 135 -13.700 -16.572 -4.800 0.00 0.00 N

ATOM 1051 CA THR A 135 -12.284 -16.348 -5.065 0.00 0.00 C

ATOM 1052 C THR A 135 -11.778 -15.143 -4.215 0.00 0.00 C

ATOM 1053 O THR A 135 -12.573 -14.225 -3.954 0.00 0.00 OA

ATOM 1054 CB THR A 135 -12.076 -16.167 -6.596 0.00 0.00 C

ATOM 1055 OG1 THR A 135 -10.684 -16.191 -6.905 0.00 0.00 OA

ATOM 1056 CG2 THR A 135 -12.708 -14.880 -7.151 0.00 0.00 C

ATOM 1057 N PRO A 136 -10.486 -15.090 -3.782 0.00 0.00 N

ATOM 1058 CA PRO A 136 -10.022 -13.892 -3.059 0.00 0.00 C

ATOM 1059 C PRO A 136 -10.080 -12.662 -3.971 0.00 0.00 C

ATOM 1060 O PRO A 136 -10.127 -12.804 -5.202 0.00 0.00 OA

ATOM 1061 CB PRO A 136 -8.575 -14.228 -2.685 0.00 0.00 C

ATOM 1062 CG PRO A 136 -8.410 -15.679 -2.928 0.00 0.00 C

ATOM 1063 CD PRO A 136 -9.383 -16.050 -3.987 0.00 0.00 C

ATOM 1064 N ILE A 137 -10.117 -11.463 -3.380 0.00 0.00 N

ATOM 1065 CA ILE A 137 -10.169 -10.227 -4.160 0.00 0.00 C

ATOM 1066 C ILE A 137 -8.790 -9.925 -4.746 0.00 0.00 C

ATOM 1067 O ILE A 137 -7.793 -9.915 -4.028 0.00 0.00 OA

ATOM 1068 CB ILE A 137 -10.772 -9.056 -3.338 0.00 0.00 C

ATOM 1069 CG1 ILE A 137 -12.229 -9.362 -2.855 0.00 0.00 C

ATOM 1070 CG2 ILE A 137 -10.676 -7.702 -4.062 0.00 0.00 C

ATOM 1071 CD1 ILE A 137 -13.304 -9.758 -3.931 0.00 0.00 C

ATOM 1072 N THR A 138 -8.738 -9.724 -6.052 0.00 0.00 N

ATOM 1073 CA THR A 138 -7.495 -9.429 -6.762 0.00 0.00 C

ATOM 1074 C THR A 138 -7.254 -7.906 -6.763 0.00 0.00 C

ATOM 1075 O THR A 138 -8.208 -7.140 -6.581 0.00 0.00 OA

ATOM 1076 CB THR A 138 -7.569 -10.010 -8.188 0.00 0.00 C

ATOM 1077 OG1 THR A 138 -8.574 -9.313 -8.937 0.00 0.00 OA

ATOM 1078 CG2 THR A 138 -7.861 -11.508 -8.198 0.00 0.00 C

ATOM 1079 N TYR A 139 -5.994 -7.467 -6.973 0.00 0.00 N

ATOM 1080 CA TYR A 139 -5.660 -6.038 -7.049 0.00 0.00 C

ATOM 1081 C TYR A 139 -6.551 -5.311 -8.100 0.00 0.00 C

ATOM 1082 O TYR A 139 -7.140 -4.278 -7.740 0.00 0.00 OA

ATOM 1083 CB TYR A 139 -4.152 -5.801 -7.290 0.00 0.00 C

ATOM 1084 CG TYR A 139 -3.735 -4.351 -7.138 0.00 0.00 A

ATOM 1085 CD1 TYR A 139 -3.801 -3.468 -8.214 0.00 0.00 A

ATOM 1086 CD2 TYR A 139 -3.249 -3.868 -5.927 0.00 0.00 A

ATOM 1087 CE1 TYR A 139 -3.438 -2.127 -8.074 0.00 0.00 A

ATOM 1088 CE2 TYR A 139 -2.878 -2.532 -5.776 0.00 0.00 A

ATOM 1089 CZ TYR A 139 -2.969 -1.666 -6.855 0.00 0.00 A

ATOM 1090 OH TYR A 139 -2.599 -0.350 -6.711 0.00 0.00 OA

ATOM 1091 N PRO A 140 -6.752 -5.856 -9.345 0.00 0.00 N

ATOM 1092 CA PRO A 140 -7.637 -5.169 -10.308 0.00 0.00 C

ATOM 1093 C PRO A 140 -9.079 -4.993 -9.815 0.00 0.00 C

ATOM 1094 O PRO A 140 -9.658 -3.928 -10.059 0.00 0.00 OA

ATOM 1095 CB PRO A 140 -7.545 -6.048 -11.564 0.00 0.00 C

ATOM 1096 CG PRO A 140 -6.277 -6.783 -11.422 0.00 0.00 C

ATOM 1097 CD PRO A 140 -6.160 -7.060 -9.968 0.00 0.00 C

ATOM 1098 N GLN A 141 -9.637 -6.015 -9.090 0.00 0.00 N

ATOM 1099 CA GLN A 141 -10.987 -5.968 -8.483 0.00 0.00 C

ATOM 1100 C GLN A 141 -11.044 -4.813 -7.470 0.00 0.00 C

ATOM 1101 O GLN A 141 -11.902 -3.936 -7.589 0.00 0.00 OA

ATOM 1102 CB GLN A 141 -11.336 -7.281 -7.755 0.00 0.00 C

ATOM 1103 CG GLN A 141 -11.774 -8.429 -8.642 0.00 0.00 C

ATOM 1104 CD GLN A 141 -12.214 -9.609 -7.809 0.00 0.00 C

ATOM 1105 OE1 GLN A 141 -11.417 -10.480 -7.431 0.00 0.00 OA

ATOM 1106 NE2 GLN A 141 -13.499 -9.647 -7.479 0.00 0.00 N

ATOM 1107 N GLY A 142 -10.104 -4.812 -6.522 0.00 0.00 N

ATOM 1108 CA GLY A 142 -9.977 -3.782 -5.497 0.00 0.00 C

ATOM 1109 C GLY A 142 -9.823 -2.365 -6.028 0.00 0.00 C

ATOM 1110 O GLY A 142 -10.465 -1.445 -5.514 0.00 0.00 OA

ATOM 1111 N LEU A 143 -8.982 -2.179 -7.072 0.00 0.00 N

ATOM 1112 CA LEU A 143 -8.752 -0.870 -7.707 0.00 0.00 C

ATOM 1113 C LEU A 143 -10.007 -0.350 -8.436 0.00 0.00 C

ATOM 1114 O LEU A 143 -10.245 0.859 -8.459 0.00 0.00 OA

ATOM 1115 CB LEU A 143 -7.547 -0.923 -8.660 0.00 0.00 C

ATOM 1116 CG LEU A 143 -7.131 0.406 -9.276 0.00 0.00 C

ATOM 1117 CD1 LEU A 143 -6.005 1.049 -8.492 0.00 0.00 C

ATOM 1118 CD2 LEU A 143 -6.760 0.231 -10.714 0.00 0.00 C

ATOM 1119 N ALA A 144 -10.797 -1.260 -9.026 0.00 0.00 N

ATOM 1120 CA ALA A 144 -12.039 -0.917 -9.717 0.00 0.00 C

ATOM 1121 C ALA A 144 -13.078 -0.367 -8.708 0.00 0.00 C

ATOM 1122 O ALA A 144 -13.650 0.706 -8.941 0.00 0.00 OA

ATOM 1123 CB ALA A 144 -12.582 -2.135 -10.441 0.00 0.00 C

ATOM 1124 N MET A 145 -13.256 -1.072 -7.562 0.00 0.00 N

ATOM 1125 CA MET A 145 -14.157 -0.690 -6.468 0.00 0.00 C

ATOM 1126 C MET A 145 -13.757 0.668 -5.917 0.00 0.00 C

ATOM 1127 O MET A 145 -14.628 1.498 -5.656 0.00 0.00 OA

ATOM 1128 CB MET A 145 -14.140 -1.747 -5.355 0.00 0.00 C

ATOM 1129 CG MET A 145 -15.086 -1.447 -4.191 0.00 0.00 C

ATOM 1130 SD MET A 145 -16.859 -1.548 -4.577 0.00 0.00 SA

ATOM 1131 CE MET A 145 -17.143 -3.273 -4.249 0.00 0.00 C

ATOM 1132 N ALA A 146 -12.439 0.899 -5.788 0.00 0.00 N

ATOM 1133 CA ALA A 146 -11.857 2.141 -5.302 0.00 0.00 C

ATOM 1134 C ALA A 146 -12.293 3.295 -6.174 0.00 0.00 C

ATOM 1135 O ALA A 146 -12.930 4.217 -5.669 0.00 0.00 OA

ATOM 1136 CB ALA A 146 -10.344 2.034 -5.265 0.00 0.00 C

ATOM 1137 N LYS A 147 -12.052 3.188 -7.498 0.00 0.00 N

ATOM 1138 CA LYS A 147 -12.417 4.202 -8.498 0.00 0.00 C

ATOM 1139 C LYS A 147 -13.924 4.405 -8.556 0.00 0.00 C

ATOM 1140 O LYS A 147 -14.370 5.548 -8.662 0.00 0.00 OA

ATOM 1141 CB LYS A 147 -11.832 3.853 -9.878 0.00 0.00 C

ATOM 1142 CG LYS A 147 -10.325 4.061 -9.950 0.00 0.00 C

ATOM 1143 CD LYS A 147 -9.738 3.645 -11.289 0.00 0.00 C

ATOM 1144 CE LYS A 147 -8.251 3.915 -11.364 0.00 0.00 C

ATOM 1145 NZ LYS A 147 -7.941 5.367 -11.507 0.00 0.00 N1+

ATOM 1146 N GLU A 148 -14.702 3.302 -8.417 0.00 0.00 N

ATOM 1147 CA GLU A 148 -16.165 3.327 -8.394 0.00 0.00 C

ATOM 1148 C GLU A 148 -16.673 4.166 -7.226 0.00 0.00 C

ATOM 1149 O GLU A 148 -17.470 5.076 -7.445 0.00 0.00 OA

ATOM 1150 CB GLU A 148 -16.736 1.906 -8.310 0.00 0.00 C

ATOM 1151 CG GLU A 148 -18.253 1.846 -8.387 0.00 0.00 C

ATOM 1152 CD GLU A 148 -18.901 0.930 -7.370 0.00 0.00 C

ATOM 1153 OE1 GLU A 148 -18.762 -0.307 -7.508 0.00 0.00 OA

ATOM 1154 OE2 GLU A 148 -19.558 1.450 -6.439 0.00 0.00 OA1-

ATOM 1155 N ILE A 149 -16.178 3.882 -5.992 0.00 0.00 N

ATOM 1156 CA ILE A 149 -16.556 4.587 -4.755 0.00 0.00 C

ATOM 1157 C ILE A 149 -15.781 5.916 -4.595 0.00 0.00 C

ATOM 1158 O ILE A 149 -15.829 6.535 -3.532 0.00 0.00 OA

ATOM 1159 CB ILE A 149 -16.469 3.669 -3.485 0.00 0.00 C

ATOM 1160 CG1 ILE A 149 -15.010 3.382 -3.044 0.00 0.00 C

ATOM 1161 CG2 ILE A 149 -17.272 2.367 -3.637 0.00 0.00 C

ATOM 1162 CD1 ILE A 149 -14.654 4.018 -1.775 0.00 0.00 C

ATOM 1163 N GLY A 150 -15.074 6.327 -5.647 0.00 0.00 N

ATOM 1164 CA GLY A 150 -14.312 7.570 -5.694 0.00 0.00 C

ATOM 1165 C GLY A 150 -13.151 7.687 -4.727 0.00 0.00 C

ATOM 1166 O GLY A 150 -12.786 8.803 -4.343 0.00 0.00 OA

ATOM 1167 N ALA A 151 -12.550 6.548 -4.331 0.00 0.00 N

ATOM 1168 CA ALA A 151 -11.403 6.537 -3.414 0.00 0.00 C

ATOM 1169 C ALA A 151 -10.214 7.248 -4.041 0.00 0.00 C

ATOM 1170 O ALA A 151 -10.000 7.162 -5.257 0.00 0.00 OA

ATOM 1171 CB ALA A 151 -11.021 5.114 -3.023 0.00 0.00 C

ATOM 1172 N VAL A 152 -9.496 8.009 -3.209 0.00 0.00 N

ATOM 1173 CA VAL A 152 -8.304 8.778 -3.555 0.00 0.00 C

ATOM 1174 C VAL A 152 -7.169 7.806 -3.958 0.00 0.00 C

ATOM 1175 O VAL A 152 -6.394 8.097 -4.867 0.00 0.00 OA

ATOM 1176 CB VAL A 152 -7.947 9.687 -2.338 0.00 0.00 C

ATOM 1177 CG1 VAL A 152 -6.461 10.057 -2.282 0.00 0.00 C

ATOM 1178 CG2 VAL A 152 -8.818 10.937 -2.319 0.00 0.00 C

ATOM 1179 N LYS A 153 -7.115 6.644 -3.295 0.00 0.00 N

ATOM 1180 CA LYS A 153 -6.108 5.607 -3.462 0.00 0.00 C

ATOM 1181 C LYS A 153 -6.680 4.243 -3.036 0.00 0.00 C

ATOM 1182 O LYS A 153 -7.660 4.185 -2.287 0.00 0.00 OA

ATOM 1183 CB LYS A 153 -4.885 5.972 -2.588 0.00 0.00 C

ATOM 1184 CG LYS A 153 -3.639 5.118 -2.792 0.00 0.00 C

ATOM 1185 CD LYS A 153 -2.502 5.900 -3.412 0.00 0.00 C

ATOM 1186 CE LYS A 153 -1.207 5.140 -3.268 0.00 0.00 C

ATOM 1187 NZ LYS A 153 -0.037 5.935 -3.726 0.00 0.00 N1+

ATOM 1188 N TYR A 154 -6.069 3.153 -3.546 0.00 0.00 N

ATOM 1189 CA TYR A 154 -6.360 1.755 -3.215 0.00 0.00 C

ATOM 1190 C TYR A 154 -5.048 1.132 -2.774 0.00 0.00 C

ATOM 1191 O TYR A 154 -4.053 1.231 -3.486 0.00 0.00 OA

ATOM 1192 CB TYR A 154 -6.970 0.971 -4.388 0.00 0.00 C

ATOM 1193 CG TYR A 154 -7.062 -0.515 -4.114 0.00 0.00 A

ATOM 1194 CD1 TYR A 154 -7.970 -1.019 -3.187 0.00 0.00 A

ATOM 1195 CD2 TYR A 154 -6.194 -1.414 -4.733 0.00 0.00 A

ATOM 1196 CE1 TYR A 154 -8.028 -2.381 -2.897 0.00 0.00 A

ATOM 1197 CE2 TYR A 154 -6.243 -2.781 -4.449 0.00 0.00 A

ATOM 1198 CZ TYR A 154 -7.162 -3.259 -3.531 0.00 0.00 A

ATOM 1199 OH TYR A 154 -7.234 -4.601 -3.260 0.00 0.00 OA

ATOM 1200 N LEU A 155 -5.049 0.510 -1.602 0.00 0.00 N

ATOM 1201 CA LEU A 155 -3.872 -0.088 -0.996 0.00 0.00 C

ATOM 1202 C LEU A 155 -4.187 -1.430 -0.398 0.00 0.00 C

ATOM 1203 O LEU A 155 -5.281 -1.644 0.121 0.00 0.00 OA

ATOM 1204 CB LEU A 155 -3.328 0.824 0.119 0.00 0.00 C

ATOM 1205 CG LEU A 155 -2.698 2.160 -0.296 0.00 0.00 C

ATOM 1206 CD1 LEU A 155 -2.621 3.094 0.878 0.00 0.00 C

ATOM 1207 CD2 LEU A 155 -1.317 1.980 -0.899 0.00 0.00 C

ATOM 1208 N GLU A 156 -3.211 -2.319 -0.434 0.00 0.00 N

ATOM 1209 CA GLU A 156 -3.286 -3.651 0.144 0.00 0.00 C

ATOM 1210 C GLU A 156 -2.260 -3.713 1.267 0.00 0.00 C

ATOM 1211 O GLU A 156 -1.312 -2.925 1.265 0.00 0.00 OA

ATOM 1212 CB GLU A 156 -3.039 -4.736 -0.919 0.00 0.00 C

ATOM 1213 CG GLU A 156 -4.129 -4.767 -1.990 0.00 0.00 C

ATOM 1214 CD GLU A 156 -4.056 -5.853 -3.051 0.00 0.00 C

ATOM 1215 OE1 GLU A 156 -2.964 -6.434 -3.251 0.00 0.00 OA

ATOM 1216 OE2 GLU A 156 -5.095 -6.113 -3.698 0.00 0.00 OA1-

ATOM 1217 N CYS A 157 -2.480 -4.588 2.257 0.00 0.00 N

ATOM 1218 CA CYS A 157 -1.583 -4.731 3.400 0.00 0.00 C

ATOM 1219 C CYS A 157 -1.826 -6.008 4.185 0.00 0.00 C

ATOM 1220 O CYS A 157 -2.843 -6.676 4.016 0.00 0.00 OA

ATOM 1221 CB CYS A 157 -1.660 -3.507 4.315 0.00 0.00 C

ATOM 1222 SG CYS A 157 -3.258 -3.310 5.151 0.00 0.00 SA

ATOM 1223 N SER A 158 -0.900 -6.305 5.090 0.00 0.00 N

ATOM 1224 CA SER A 158 -1.008 -7.403 6.028 0.00 0.00 C

ATOM 1225 C SER A 158 -0.495 -6.886 7.359 0.00 0.00 C

ATOM 1226 O SER A 158 0.627 -6.367 7.417 0.00 0.00 OA

ATOM 1227 CB SER A 158 -0.190 -8.607 5.574 0.00 0.00 C

ATOM 1228 OG SER A 158 -0.259 -9.646 6.540 0.00 0.00 OA

ATOM 1229 N ALA A 159 -1.314 -6.994 8.419 0.00 0.00 N

ATOM 1230 CA ALA A 159 -0.892 -6.590 9.755 0.00 0.00 C

ATOM 1231 C ALA A 159 0.018 -7.697 10.322 0.00 0.00 C

ATOM 1232 O ALA A 159 0.855 -7.426 11.187 0.00 0.00 OA

ATOM 1233 CB ALA A 159 -2.104 -6.385 10.651 0.00 0.00 C

ATOM 1234 N LEU A 160 -0.159 -8.948 9.825 0.00 0.00 N

ATOM 1235 CA LEU A 160 0.617 -10.121 10.225 0.00 0.00 C

ATOM 1236 C LEU A 160 2.088 -9.978 9.781 0.00 0.00 C

ATOM 1237 O LEU A 160 2.994 -10.172 10.599 0.00 0.00 OA

ATOM 1238 CB LEU A 160 -0.017 -11.418 9.654 0.00 0.00 C

ATOM 1239 CG LEU A 160 0.416 -12.747 10.298 0.00 0.00 C

ATOM 1240 CD1 LEU A 160 -0.646 -13.789 10.138 0.00 0.00 C

ATOM 1241 CD2 LEU A 160 1.710 -13.278 9.690 0.00 0.00 C

ATOM 1242 N THR A 161 2.319 -9.665 8.490 0.00 0.00 N

ATOM 1243 CA THR A 161 3.676 -9.527 7.961 0.00 0.00 C

ATOM 1244 C THR A 161 4.154 -8.067 7.958 0.00 0.00 C

ATOM 1245 O THR A 161 5.329 -7.821 7.672 0.00 0.00 OA

ATOM 1246 CB THR A 161 3.802 -10.201 6.582 0.00 0.00 C

ATOM 1247 OG1 THR A 161 3.247 -9.348 5.579 0.00 0.00 OA

ATOM 1248 CG2 THR A 161 3.158 -11.586 6.538 0.00 0.00 C

ATOM 1249 N GLN A 162 3.240 -7.105 8.267 0.00 0.00 N

ATOM 1250 CA GLN A 162 3.461 -5.642 8.287 0.00 0.00 C

ATOM 1251 C GLN A 162 3.757 -5.051 6.882 0.00 0.00 C

ATOM 1252 O GLN A 162 4.101 -3.875 6.765 0.00 0.00 OA

ATOM 1253 CB GLN A 162 4.519 -5.218 9.327 0.00 0.00 C

ATOM 1254 CG GLN A 162 4.022 -5.357 10.761 0.00 0.00 C

ATOM 1255 CD GLN A 162 4.607 -4.316 11.673 0.00 0.00 C

ATOM 1256 OE1 GLN A 162 4.587 -3.109 11.403 0.00 0.00 OA

ATOM 1257 NE2 GLN A 162 5.119 -4.764 12.795 0.00 0.00 N

ATOM 1258 N ARG A 163 3.567 -5.858 5.827 0.00 0.00 N

ATOM 1259 CA ARG A 163 3.799 -5.485 4.435 0.00 0.00 C

ATOM 1260 C ARG A 163 2.693 -4.544 3.959 0.00 0.00 C

ATOM 1261 O ARG A 163 1.518 -4.909 4.021 0.00 0.00 OA

ATOM 1262 CB ARG A 163 3.843 -6.755 3.556 0.00 0.00 C

ATOM 1263 CG ARG A 163 4.463 -6.559 2.167 0.00 0.00 C

ATOM 1264 CD ARG A 163 4.429 -7.829 1.323 0.00 0.00 C

ATOM 1265 NE ARG A 163 5.050 -8.968 2.007 0.00 0.00 N

ATOM 1266 CZ ARG A 163 6.347 -9.265 1.964 0.00 0.00 C

ATOM 1267 NH1 ARG A 163 7.183 -8.524 1.242 0.00 0.00 N1+

ATOM 1268 NH2 ARG A 163 6.817 -10.309 2.636 0.00 0.00 N

ATOM 1269 N GLY A 164 3.081 -3.358 3.493 0.00 0.00 N

ATOM 1270 CA GLY A 164 2.165 -2.346 2.975 0.00 0.00 C

ATOM 1271 C GLY A 164 1.505 -1.466 4.020 0.00 0.00 C

ATOM 1272 O GLY A 164 0.810 -0.515 3.670 0.00 0.00 OA

ATOM 1273 N LEU A 165 1.745 -1.767 5.305 0.00 0.00 N

ATOM 1274 CA LEU A 165 1.185 -1.104 6.485 0.00 0.00 C

ATOM 1275 C LEU A 165 1.618 0.376 6.661 0.00 0.00 C

ATOM 1276 O LEU A 165 0.762 1.251 6.844 0.00 0.00 OA

ATOM 1277 CB LEU A 165 1.532 -1.953 7.711 0.00 0.00 C

ATOM 1278 CG LEU A 165 0.940 -1.567 9.042 0.00 0.00 C

ATOM 1279 CD1 LEU A 165 -0.577 -1.726 9.047 0.00 0.00 C

ATOM 1280 CD2 LEU A 165 1.523 -2.428 10.127 0.00 0.00 C

ATOM 1281 N LYS A 166 2.936 0.650 6.594 0.00 0.00 N

ATOM 1282 CA LYS A 166 3.487 2.001 6.715 0.00 0.00 C

ATOM 1283 C LYS A 166 2.929 2.890 5.596 0.00 0.00 C

ATOM 1284 O LYS A 166 2.722 4.083 5.820 0.00 0.00 OA

ATOM 1285 CB LYS A 166 5.026 1.962 6.646 0.00 0.00 C

ATOM 1286 CG LYS A 166 5.722 3.240 7.119 0.00 0.00 C

ATOM 1287 CD LYS A 166 6.984 3.549 6.289 0.00 0.00 C

ATOM 1288 CE LYS A 166 7.828 4.687 6.832 0.00 0.00 C

ATOM 1289 NZ LYS A 166 7.109 5.993 6.832 0.00 0.00 N1+

ATOM 1290 N THR A 167 2.675 2.297 4.402 0.00 0.00 N

ATOM 1291 CA THR A 167 2.167 2.997 3.219 0.00 0.00 C

ATOM 1292 C THR A 167 0.763 3.512 3.462 0.00 0.00 C

ATOM 1293 O THR A 167 0.500 4.681 3.207 0.00 0.00 OA

ATOM 1294 CB THR A 167 2.299 2.130 1.942 0.00 0.00 C

ATOM 1295 OG1 THR A 167 3.628 1.608 1.853 0.00 0.00 OA

ATOM 1296 CG2 THR A 167 1.998 2.910 0.674 0.00 0.00 C

ATOM 1297 N VAL A 168 -0.117 2.637 3.971 0.00 0.00 N

ATOM 1298 CA VAL A 168 -1.520 2.893 4.294 0.00 0.00 C

ATOM 1299 C VAL A 168 -1.680 4.226 5.039 0.00 0.00 C

ATOM 1300 O VAL A 168 -2.406 5.104 4.558 0.00 0.00 OA

ATOM 1301 CB VAL A 168 -2.140 1.687 5.069 0.00 0.00 C

ATOM 1302 CG1 VAL A 168 -3.454 2.057 5.766 0.00 0.00 C

ATOM 1303 CG2 VAL A 168 -2.326 0.473 4.165 0.00 0.00 C

ATOM 1304 N PHE A 169 -0.955 4.389 6.176 0.00 0.00 N

ATOM 1305 CA PHE A 169 -1.058 5.565 7.031 0.00 0.00 C

ATOM 1306 C PHE A 169 -0.239 6.742 6.493 0.00 0.00 C

ATOM 1307 O PHE A 169 -0.594 7.889 6.781 0.00 0.00 OA

ATOM 1308 CB PHE A 169 -0.761 5.220 8.504 0.00 0.00 C

ATOM 1309 CG PHE A 169 -1.635 4.077 8.998 0.00 0.00 A

ATOM 1310 CD1 PHE A 169 -2.971 4.289 9.320 0.00 0.00 A

ATOM 1311 CD2 PHE A 169 -1.162 2.770 9.003 0.00 0.00 A

ATOM 1312 CE1 PHE A 169 -3.791 3.225 9.714 0.00 0.00 A

ATOM 1313 CE2 PHE A 169 -1.982 1.708 9.394 0.00 0.00 A

ATOM 1314 CZ PHE A 169 -3.288 1.941 9.752 0.00 0.00 A

ATOM 1315 N ASP A 170 0.773 6.474 5.633 0.00 0.00 N

ATOM 1316 CA ASP A 170 1.550 7.528 4.961 0.00 0.00 C

ATOM 1317 C ASP A 170 0.657 8.224 3.930 0.00 0.00 C

ATOM 1318 O ASP A 170 0.701 9.448 3.808 0.00 0.00 OA

ATOM 1319 CB ASP A 170 2.790 6.947 4.262 0.00 0.00 C

ATOM 1320 CG ASP A 170 4.124 7.203 4.949 0.00 0.00 C

ATOM 1321 OD1 ASP A 170 4.129 7.837 6.034 0.00 0.00 OA

ATOM 1322 OD2 ASP A 170 5.168 6.775 4.397 0.00 0.00 OA1-

ATOM 1323 N GLU A 171 -0.188 7.432 3.233 0.00 0.00 N

ATOM 1324 CA GLU A 171 -1.145 7.868 2.212 0.00 0.00 C

ATOM 1325 C GLU A 171 -2.367 8.512 2.837 0.00 0.00 C

ATOM 1326 O GLU A 171 -3.026 9.352 2.207 0.00 0.00 OA

ATOM 1327 CB GLU A 171 -1.542 6.710 1.277 0.00 0.00 C

ATOM 1328 CG GLU A 171 -0.438 6.263 0.325 0.00 0.00 C

ATOM 1329 CD GLU A 171 0.099 7.286 -0.666 0.00 0.00 C

ATOM 1330 OE1 GLU A 171 -0.681 8.153 -1.127 0.00 0.00 OA

ATOM 1331 OE2 GLU A 171 1.299 7.192 -1.014 0.00 0.00 OA1-

ATOM 1332 N ALA A 172 -2.659 8.126 4.092 0.00 0.00 N

ATOM 1333 CA ALA A 172 -3.740 8.709 4.882 0.00 0.00 C

ATOM 1334 C ALA A 172 -3.353 10.179 5.113 0.00 0.00 C

ATOM 1335 O ALA A 172 -4.158 11.075 4.856 0.00 0.00 OA

ATOM 1336 CB ALA A 172 -3.870 7.974 6.207 0.00 0.00 C

ATOM 1337 N ILE A 173 -2.083 10.415 5.500 0.00 0.00 N

ATOM 1338 CA ILE A 173 -1.534 11.753 5.703 0.00 0.00 C

ATOM 1339 C ILE A 173 -1.498 12.492 4.358 0.00 0.00 C

ATOM 1340 O ILE A 173 -1.952 13.632 4.285 0.00 0.00 OA

ATOM 1341 CB ILE A 173 -0.178 11.693 6.459 0.00 0.00 C

ATOM 1342 CG1 ILE A 173 -0.398 11.148 7.884 0.00 0.00 C

ATOM 1343 CG2 ILE A 173 0.512 13.066 6.511 0.00 0.00 C

ATOM 1344 CD1 ILE A 173 0.728 10.347 8.443 0.00 0.00 C

ATOM 1345 N ARG A 174 -1.065 11.801 3.288 0.00 0.00 N

ATOM 1346 CA ARG A 174 -1.016 12.336 1.917 0.00 0.00 C

ATOM 1347 C ARG A 174 -2.387 12.744 1.347 0.00 0.00 C

ATOM 1348 O ARG A 174 -2.439 13.664 0.531 0.00 0.00 OA

ATOM 1349 CB ARG A 174 -0.310 11.361 0.962 0.00 0.00 C

ATOM 1350 CG ARG A 174 1.212 11.360 1.087 0.00 0.00 C

ATOM 1351 CD ARG A 174 1.842 10.411 0.086 0.00 0.00 C

ATOM 1352 NE ARG A 174 3.302 10.517 0.073 0.00 0.00 N

ATOM 1353 CZ ARG A 174 4.121 9.685 0.706 0.00 0.00 C

ATOM 1354 NH1 ARG A 174 3.636 8.669 1.409 0.00 0.00 N1+

ATOM 1355 NH2 ARG A 174 5.435 9.856 0.631 0.00 0.00 N

ATOM 1356 N ALA A 175 -3.489 12.070 1.772 0.00 0.00 N

ATOM 1357 CA ALA A 175 -4.859 12.376 1.324 0.00 0.00 C

ATOM 1358 C ALA A 175 -5.338 13.702 1.914 0.00 0.00 C

ATOM 1359 O ALA A 175 -6.208 14.366 1.338 0.00 0.00 OA

ATOM 1360 CB ALA A 175 -5.807 11.258 1.706 0.00 0.00 C

ATOM 1361 N VAL A 176 -4.743 14.080 3.064 0.00 0.00 N

ATOM 1362 CA VAL A 176 -4.936 15.348 3.778 0.00 0.00 C

ATOM 1363 C VAL A 176 -3.698 16.176 3.338 0.00 0.00 C

ATOM 1364 O VAL A 176 -2.982 15.726 2.440 0.00 0.00 OA

ATOM 1365 CB VAL A 176 -5.003 15.118 5.329 0.00 0.00 C

ATOM 1366 CG1 VAL A 176 -5.503 16.359 6.060 0.00 0.00 C

ATOM 1367 CG2 VAL A 176 -5.887 13.922 5.678 0.00 0.00 C

ATOM 1368 N LEU A 177 -3.442 17.359 3.939 0.00 0.00 N

ATOM 1369 CA LEU A 177 -2.272 18.210 3.661 0.00 0.00 C

ATOM 1370 C LEU A 177 -2.123 18.578 2.183 0.00 0.00 C

ATOM 1371 O LEU A 177 -1.963 19.748 1.849 0.00 0.00 OA

ATOM 1372 CB LEU A 177 -0.982 17.537 4.186 0.00 0.00 C

ATOM 1373 CG LEU A 177 -0.562 17.807 5.641 0.00 0.00 C

ATOM 1374 CD1 LEU A 177 -1.575 17.280 6.646 0.00 0.00 C

ATOM 1375 CD2 LEU A 177 0.786 17.175 5.932 0.00 0.00 C

ATOM 1376 OXT LEU A 177 -2.135 17.859 1.187 0.00 0.00 OA1-

ATOM 1377 C10 MOL X 1 -15.526 4.593 18.796 1.00 0.00 C

ATOM 1378 N3 MOL X 1 -16.324 5.735 18.895 1.00 0.00 N

ATOM 1379 C11 MOL X 1 -14.325 4.520 19.501 1.00 0.00 C

ATOM 1380 C13 MOL X 1 -13.920 5.587 20.303 1.00 0.00 C

ATOM 1381 C14 MOL X 1 -12.594 5.504 21.082 1.00 0.00 C

ATOM 1382 N4 MOL X 1 -14.717 6.727 20.402 1.00 0.00 N

ATOM 1383 C12 MOL X 1 -15.918 6.801 19.697 1.00 0.00 C

ATOM 1384 N2 MOL X 1 -15.953 3.470 17.951 1.00 0.00 N

ATOM 1385 C MOL X 1 -17.246 2.959 18.432 1.00 0.00 C

ATOM 1386 C5 MOL X 1 -18.386 3.087 17.600 1.00 0.00 C

ATOM 1387 C4 MOL X 1 -19.607 2.620 18.020 1.00 0.00 C

ATOM 1388 C3 MOL X 1 -19.743 2.002 19.294 1.00 0.00 C

ATOM 1389 N MOL X 1 -20.994 1.510 19.755 1.00 0.00 N

ATOM 1390 C2 MOL X 1 -18.602 1.875 20.127 1.00 0.00 C

ATOM 1391 C7 MOL X 1 -21.099 0.919 20.990 1.00 0.00 C

ATOM 1392 C9 MOL X 1 -22.453 0.384 21.493 1.00 0.00 C

ATOM 1393 C8 MOL X 1 -19.959 0.791 21.821 1.00 0.00 C

ATOM 1394 C6 MOL X 1 -18.739 1.258 21.401 1.00 0.00 C

ATOM 1395 C1 MOL X 1 -17.350 2.368 19.666 1.00 0.00 C

ATOM 1396 N1 MOL X 1 -17.548 1.131 22.254 1.00 0.00 N

ATOM 1397 N5 MOL X 1 -16.758 8.004 19.800 1.00 0.00 N

ATOM 1398 C15 MOL X 1 -18.132 7.613 20.148 1.00 0.00 C

ATOM 1399 C16 MOL X 1 -19.078 8.806 19.920 1.00 0.00 C

ATOM 1400 C17 MOL X 1 -18.186 7.190 21.628 1.00 0.00 C

ATOM 1401 C18 MOL X 1 -18.454 5.676 21.719 1.00 0.00 C

ATOM 1402 C19 MOL X 1 -19.732 5.333 20.930 1.00 0.00 C

ATOM 1403 N6 MOL X 1 -20.554 4.398 21.711 1.00 0.00 N

ATOM 1404 C20 MOL X 1 -21.942 4.881 21.751 1.00 0.00 C

ATOM 1405 C22 MOL X 1 -21.978 6.374 21.372 1.00 0.00 C

ATOM 1406 C21 MOL X 1 -20.027 4.307 23.079 1.00 0.00 C

ATOM 1407 C23 MOL X 1 -21.072 3.632 23.988 1.00 0.00 C

Rac1-NSC23766 structure from HDOCK

ATOM 1 N GLN A 2 0.578 19.705 12.487 1.00 44.49 N

ATOM 2 CA GLN A 2 -0.825 19.520 12.140 1.00 44.24 C

ATOM 3 C GLN A 2 -1.331 18.147 12.607 1.00 47.85 C

ATOM 4 O GLN A 2 -0.743 17.125 12.243 1.00 48.30 O

ATOM 5 CB GLN A 2 -1.050 19.726 10.627 1.00 45.62 C

ATOM 6 CG GLN A 2 -2.530 19.905 10.242 1.00 69.79 C

ATOM 7 CD GLN A 2 -2.781 20.211 8.776 1.00 97.83 C

ATOM 8 OE1 GLN A 2 -1.863 20.308 7.953 1.00 97.85 O

ATOM 9 NE2 GLN A 2 -4.046 20.386 8.416 1.00 88.23 N

ATOM 10 N ALA A 3 -2.418 18.135 13.426 1.00 42.73 N

ATOM 11 CA ALA A 3 -3.041 16.921 13.975 1.00 40.85 C

ATOM 12 C ALA A 3 -4.173 16.436 13.064 1.00 42.21 C

ATOM 13 O ALA A 3 -5.178 17.138 12.894 1.00 42.92 O

ATOM 14 CB ALA A 3 -3.559 17.173 15.388 1.00 41.27 C

ATOM 15 N ILE A 4 -3.987 15.250 12.455 1.00 34.48 N

ATOM 16 CA ILE A 4 -4.940 14.625 11.535 1.00 32.17 C

ATOM 17 C ILE A 4 -5.801 13.595 12.277 1.00 32.61 C

ATOM 18 O ILE A 4 -5.348 13.017 13.259 1.00 32.48 O

ATOM 19 CB ILE A 4 -4.181 13.991 10.336 1.00 35.14 C

ATOM 20 CG1 ILE A 4 -3.220 14.997 9.673 1.00 35.33 C

ATOM 21 CG2 ILE A 4 -5.136 13.363 9.311 1.00 35.93 C

ATOM 22 CD1 ILE A 4 -2.371 14.399 8.620 1.00 43.68 C

ATOM 23 N LYS A 5 -7.046 13.387 11.815 1.00 26.95 N

ATOM 24 CA LYS A 5 -7.990 12.407 12.359 1.00 25.22 C

ATOM 25 C LYS A 5 -8.423 11.451 11.244 1.00 28.73 C

ATOM 26 O LYS A 5 -9.010 11.866 10.241 1.00 26.42 O

ATOM 27 CB LYS A 5 -9.193 13.082 13.015 1.00 25.75 C

ATOM 28 CG LYS A 5 -10.119 12.094 13.698 1.00 21.04 C

ATOM 29 CD LYS A 5 -11.454 12.699 14.010 1.00 17.37 C

ATOM 30 CE LYS A 5 -12.456 11.661 14.403 1.00 17.54 C

ATOM 31 NZ LYS A 5 -13.690 12.296 14.948 1.00 21.40 N

ATOM 32 N CYS A 6 -8.108 10.165 11.440 1.00 27.17 N

ATOM 33 CA CYS A 6 -8.360 9.088 10.493 1.00 27.18 C

ATOM 34 C CYS A 6 -9.274 8.037 11.084 1.00 28.15 C

ATOM 35 O CYS A 6 -8.880 7.301 11.990 1.00 27.82 O

ATOM 36 CB CYS A 6 -7.037 8.491 10.004 1.00 28.23 C

ATOM 37 SG CYS A 6 -7.200 6.942 9.070 1.00 32.45 S

ATOM 38 N VAL A 7 -10.493 7.956 10.562 1.00 23.30 N

ATOM 39 CA VAL A 7 -11.449 6.951 11.017 1.00 23.01 C

ATOM 40 C VAL A 7 -11.292 5.670 10.177 1.00 25.99 C

ATOM 41 O VAL A 7 -11.189 5.732 8.949 1.00 25.41 O

ATOM 42 CB VAL A 7 -12.914 7.479 11.056 1.00 26.75 C

ATOM 43 CG1 VAL A 7 -13.853 6.458 11.689 1.00 26.39 C

ATOM 44 CG2 VAL A 7 -12.994 8.791 11.823 1.00 26.54 C

ATOM 45 N VAL A 8 -11.251 4.519 10.855 1.00 21.47 N

ATOM 46 CA VAL A 8 -11.146 3.207 10.223 1.00 20.92 C

ATOM 47 C VAL A 8 -12.533 2.538 10.320 1.00 27.79 C

ATOM 48 O VAL A 8 -13.055 2.366 11.426 1.00 28.30 O

ATOM 49 CB VAL A 8 -10.058 2.337 10.885 1.00 23.79 C

ATOM 50 CG1 VAL A 8 -9.807 1.079 10.072 1.00 23.42 C

ATOM 51 CG2 VAL A 8 -8.764 3.116 11.084 1.00 23.60 C

ATOM 52 N VAL A 9 -13.130 2.198 9.165 1.00 24.58 N

ATOM 53 CA VAL A 9 -14.451 1.560 9.048 1.00 24.55 C

ATOM 54 C VAL A 9 -14.353 0.270 8.227 1.00 28.24 C

ATOM 55 O VAL A 9 -13.391 0.102 7.477 1.00 28.46 O

ATOM 56 CB VAL A 9 -15.558 2.517 8.512 1.00 28.43 C

ATOM 57 CG1 VAL A 9 -15.870 3.618 9.520 1.00 28.71 C

ATOM 58 CG2 VAL A 9 -15.198 3.106 7.147 1.00 27.77 C

ATOM 59 N GLY A 10 -15.337 -0.615 8.379 1.00 23.59 N

ATOM 60 CA GLY A 10 -15.372 -1.906 7.700 1.00 23.34 C

ATOM 61 C GLY A 10 -16.065 -2.970 8.518 1.00 28.54 C

ATOM 62 O GLY A 10 -16.413 -2.724 9.678 1.00 29.15 O

ATOM 63 N ASP A 11 -16.300 -4.159 7.919 1.00 25.69 N

ATOM 64 CA ASP A 11 -17.002 -5.279 8.589 1.00 24.89 C

ATOM 65 C ASP A 11 -16.218 -5.798 9.783 1.00 26.12 C

ATOM 66 O ASP A 11 -15.016 -5.609 9.851 1.00 23.37 O

ATOM 67 CB ASP A 11 -17.244 -6.470 7.621 1.00 26.26 C

ATOM 68 CG ASP A 11 -18.279 -6.317 6.507 1.00 33.03 C

ATOM 69 OD1 ASP A 11 -18.784 -5.179 6.301 1.00 32.56 O

ATOM 70 OD2 ASP A 11 -18.573 -7.333 5.829 1.00 33.02 O

ATOM 71 N GLY A 12 -16.911 -6.470 10.701 1.00 24.44 N

ATOM 72 CA GLY A 12 -16.284 -7.158 11.821 1.00 23.83 C

ATOM 73 C GLY A 12 -15.379 -8.252 11.265 1.00 27.31 C

ATOM 74 O GLY A 12 -15.650 -8.799 10.184 1.00 26.80 O

ATOM 75 N ALA A 13 -14.251 -8.505 11.962 1.00 22.12 N

ATOM 76 CA ALA A 13 -13.217 -9.496 11.673 1.00 21.11 C

ATOM 77 C ALA A 13 -12.358 -9.186 10.422 1.00 26.35 C

ATOM 78 O ALA A 13 -11.515 -10.007 10.064 1.00 25.75 O

ATOM 79 CB ALA A 13 -13.810 -10.901 11.604 1.00 21.36 C

ATOM 80 N VAL A 14 -12.498 -7.988 9.802 1.00 24.19 N

ATOM 81 CA VAL A 14 -11.665 -7.613 8.631 1.00 23.61 C

ATOM 82 C VAL A 14 -10.207 -7.383 9.029 1.00 27.91 C

ATOM 83 O VAL A 14 -9.338 -7.352 8.171 1.00 28.47 O

ATOM 84 CB VAL A 14 -12.198 -6.439 7.759 1.00 26.32 C

ATOM 85 CG1 VAL A 14 -13.527 -6.775 7.109 1.00 25.46 C

ATOM 86 CG2 VAL A 14 -12.272 -5.133 8.540 1.00 26.01 C

ATOM 87 N GLY A 15 -9.963 -7.201 10.319 1.00 24.51 N

ATOM 88 CA GLY A 15 -8.623 -7.003 10.845 1.00 24.18 C

ATOM 89 C GLY A 15 -8.262 -5.567 11.141 1.00 26.98 C

ATOM 90 O GLY A 15 -7.068 -5.249 11.184 1.00 26.37 O

ATOM 91 N LYS A 16 -9.292 -4.700 11.346 1.00 22.10 N

ATOM 92 CA LYS A 16 -9.158 -3.267 11.653 1.00 22.43 C

ATOM 93 C LYS A 16 -8.323 -2.986 12.912 1.00 28.18 C

ATOM 94 O LYS A 16 -7.422 -2.151 12.860 1.00 29.82 O

ATOM 95 CB LYS A 16 -10.539 -2.604 11.828 1.00 24.99 C

ATOM 96 CG LYS A 16 -11.284 -2.296 10.533 1.00 29.17 C

ATOM 97 CD LYS A 16 -12.641 -1.584 10.752 1.00 34.50 C

ATOM 98 CE LYS A 16 -13.580 -2.119 11.830 1.00 31.06 C

ATOM 99 NZ LYS A 16 -13.945 -3.549 11.645 1.00 27.95 N

ATOM 100 N THR A 17 -8.635 -3.649 14.037 1.00 23.93 N

ATOM 101 CA THR A 17 -7.923 -3.434 15.297 1.00 24.58 C

ATOM 102 C THR A 17 -6.478 -3.934 15.239 1.00 31.44 C

ATOM 103 O THR A 17 -5.594 -3.302 15.829 1.00 32.16 O

ATOM 104 CB THR A 17 -8.696 -4.031 16.483 1.00 29.50 C

ATOM 105 OG1 THR A 17 -10.100 -3.812 16.317 1.00 26.79 O

ATOM 106 CG2 THR A 17 -8.204 -3.504 17.838 1.00 21.76 C

ATOM 107 N CYS A 18 -6.240 -5.066 14.539 1.00 27.97 N

ATOM 108 CA CYS A 18 -4.898 -5.631 14.360 1.00 27.67 C

ATOM 109 C CYS A 18 -4.049 -4.709 13.502 1.00 29.21 C

ATOM 110 O CYS A 18 -2.856 -4.588 13.742 1.00 30.44 O

ATOM 111 CB CYS A 18 -4.971 -7.026 13.750 1.00 28.42 C

ATOM 112 SG CYS A 18 -5.387 -8.329 14.931 1.00 32.52 S

ATOM 113 N LEU A 19 -4.666 -4.070 12.507 1.00 22.92 N

ATOM 114 CA LEU A 19 -4.032 -3.135 11.586 1.00 22.50 C

ATOM 115 C LEU A 19 -3.485 -1.925 12.386 1.00 28.29 C

ATOM 116 O LEU A 19 -2.300 -1.602 12.289 1.00 28.03 O

ATOM 117 CB LEU A 19 -5.086 -2.719 10.554 1.00 22.11 C

ATOM 118 CG LEU A 19 -4.725 -1.714 9.497 1.00 27.48 C

ATOM 119 CD1 LEU A 19 -3.998 -2.374 8.376 1.00 28.37 C

ATOM 120 CD2 LEU A 19 -5.979 -1.079 8.932 1.00 30.78 C

ATOM 121 N LEU A 20 -4.344 -1.316 13.210 1.00 26.08 N

ATOM 122 CA LEU A 20 -4.022 -0.184 14.079 1.00 26.11 C

ATOM 123 C LEU A 20 -3.001 -0.528 15.168 1.00 29.46 C

ATOM 124 O LEU A 20 -2.127 0.303 15.435 1.00 29.32 O

ATOM 125 CB LEU A 20 -5.303 0.413 14.705 1.00 25.77 C

ATOM 126 CG LEU A 20 -6.288 0.998 13.711 1.00 30.06 C

ATOM 127 CD1 LEU A 20 -7.650 1.098 14.295 1.00 30.72 C

ATOM 128 CD2 LEU A 20 -5.835 2.325 13.216 1.00 32.47 C

ATOM 129 N ILE A 21 -3.097 -1.728 15.799 1.00 25.42 N

ATOM 130 CA ILE A 21 -2.129 -2.114 16.831 1.00 26.03 C

ATOM 131 C ILE A 21 -0.778 -2.405 16.182 1.00 30.67 C

ATOM 132 O ILE A 21 0.210 -1.846 16.634 1.00 31.01 O

ATOM 133 CB ILE A 21 -2.637 -3.227 17.797 1.00 29.74 C

ATOM 134 CG1 ILE A 21 -3.434 -2.601 18.956 1.00 30.30 C

ATOM 135 CG2 ILE A 21 -1.498 -4.078 18.382 1.00 29.92 C

ATOM 136 CD1 ILE A 21 -4.801 -2.447 18.728 1.00 36.19 C

ATOM 137 N SER A 22 -0.744 -3.183 15.078 1.00 26.96 N

ATOM 138 CA SER A 22 0.490 -3.471 14.336 1.00 25.97 C

ATOM 139 C SER A 22 1.229 -2.205 13.971 1.00 28.39 C

ATOM 140 O SER A 22 2.413 -2.104 14.237 1.00 28.68 O

ATOM 141 CB SER A 22 0.202 -4.268 13.068 1.00 29.18 C

ATOM 142 OG SER A 22 -0.279 -5.559 13.393 1.00 41.63 O

ATOM 143 N TYR A 23 0.531 -1.234 13.394 1.00 24.70 N

ATOM 144 CA TYR A 23 1.121 0.019 12.954 1.00 24.64 C

ATOM 145 C TYR A 23 1.744 0.852 14.068 1.00 29.15 C

ATOM 146 O TYR A 23 2.887 1.279 13.931 1.00 29.71 O

ATOM 147 CB TYR A 23 0.102 0.854 12.167 1.00 25.25 C

ATOM 148 CG TYR A 23 0.660 2.199 11.750 1.00 25.31 C

ATOM 149 CD1 TYR A 23 1.692 2.288 10.823 1.00 25.81 C

ATOM 150 CD2 TYR A 23 0.159 3.381 12.288 1.00 26.06 C

ATOM 151 CE1 TYR A 23 2.213 3.518 10.438 1.00 25.69 C

ATOM 152 CE2 TYR A 23 0.690 4.619 11.926 1.00 26.86 C

ATOM 153 CZ TYR A 23 1.722 4.680 11.003 1.00 31.27 C

ATOM 154 OH TYR A 23 2.242 5.885 10.606 1.00 30.44 O

ATOM 155 N THR A 24 0.985 1.101 15.140 1.00 25.56 N

ATOM 156 CA THR A 24 1.389 1.942 16.268 1.00 25.18 C

ATOM 157 C THR A 24 2.380 1.278 17.203 1.00 31.01 C

ATOM 158 O THR A 24 3.267 1.957 17.724 1.00 30.94 O

ATOM 159 CB THR A 24 0.148 2.421 17.047 1.00 25.73 C

ATOM 160 OG1 THR A 24 -0.599 1.282 17.482 1.00 28.62 O

ATOM 161 CG2 THR A 24 -0.740 3.358 16.238 1.00 14.61 C

ATOM 162 N THR A 25 2.211 -0.038 17.432 1.00 29.14 N

ATOM 163 CA THR A 25 2.968 -0.871 18.373 1.00 29.61 C

ATOM 164 C THR A 25 4.183 -1.560 17.717 1.00 36.21 C

ATOM 165 O THR A 25 5.132 -1.914 18.426 1.00 35.88 O

ATOM 166 CB THR A 25 1.950 -1.838 19.038 1.00 35.76 C

ATOM 167 OG1 THR A 25 1.402 -1.224 20.207 1.00 32.34 O

ATOM 168 CG2 THR A 25 2.489 -3.232 19.346 1.00 35.11 C

ATOM 169 N ASN A 26 4.169 -1.711 16.376 1.00 34.41 N

ATOM 170 CA ASN A 26 5.192 -2.409 15.578 1.00 34.83 C

ATOM 171 C ASN A 26 5.116 -3.931 15.777 1.00 38.43 C

ATOM 172 O ASN A 26 6.059 -4.636 15.417 1.00 38.83 O

ATOM 173 CB ASN A 26 6.617 -1.857 15.791 1.00 37.31 C

ATOM 174 CG ASN A 26 6.916 -0.645 14.956 1.00 67.73 C

ATOM 175 OD1 ASN A 26 7.303 -0.754 13.786 1.00 65.29 O

ATOM 176 ND2 ASN A 26 6.717 0.537 15.530 1.00 58.37 N

ATOM 177 N ALA A 27 3.976 -4.441 16.304 1.00 33.93 N

ATOM 178 CA ALA A 27 3.783 -5.871 16.548 1.00 33.76 C

ATOM 179 C ALA A 27 2.376 -6.345 16.265 1.00 36.71 C

ATOM 180 O ALA A 27 1.417 -5.613 16.512 1.00 36.29 O

ATOM 181 CB ALA A 27 4.167 -6.225 17.981 1.00 34.57 C

ATOM 182 N PHE A 28 2.249 -7.590 15.772 1.00 32.60 N

ATOM 183 CA PHE A 28 0.946 -8.197 15.504 1.00 32.11 C

ATOM 184 C PHE A 28 0.346 -8.754 16.818 1.00 37.57 C

ATOM 185 O PHE A 28 0.974 -9.614 17.440 1.00 37.87 O

ATOM 186 CB PHE A 28 1.046 -9.273 14.411 1.00 33.18 C

ATOM 187 CG PHE A 28 -0.300 -9.804 13.978 1.00 34.44 C

ATOM 188 CD1 PHE A 28 -1.181 -9.008 13.252 1.00 36.14 C

ATOM 189 CD2 PHE A 28 -0.699 -11.095 14.316 1.00 36.17 C

ATOM 190 CE1 PHE A 28 -2.424 -9.499 12.856 1.00 36.83 C

ATOM 191 CE2 PHE A 28 -1.951 -11.580 13.927 1.00 38.37 C

ATOM 192 CZ PHE A 28 -2.804 -10.779 13.201 1.00 36.12 C

ATOM 193 N PRO A 29 -0.854 -8.291 17.256 1.00 34.63 N

ATOM 194 CA PRO A 29 -1.413 -8.757 18.543 1.00 35.50 C

ATOM 195 C PRO A 29 -1.809 -10.237 18.662 1.00 43.19 C

ATOM 196 O PRO A 29 -2.305 -10.634 19.714 1.00 41.85 O

ATOM 197 CB PRO A 29 -2.632 -7.852 18.753 1.00 36.80 C

ATOM 198 CG PRO A 29 -2.985 -7.383 17.430 1.00 40.85 C

ATOM 199 CD PRO A 29 -1.712 -7.256 16.657 1.00 36.30 C

ATOM 200 N GLY A 30 -1.578 -11.027 17.616 1.00 44.10 N

ATOM 201 CA GLY A 30 -1.886 -12.452 17.597 1.00 46.09 C

ATOM 202 C GLY A 30 -3.322 -12.797 17.942 1.00 55.43 C

ATOM 203 O GLY A 30 -4.256 -12.302 17.297 1.00 55.23 O

ATOM 204 N GLU A 31 -3.499 -13.645 18.982 1.00 55.41 N

ATOM 205 CA GLU A 31 -4.817 -14.106 19.439 1.00 56.45 C

ATOM 206 C GLU A 31 -5.517 -13.080 20.338 1.00 60.26 C

ATOM 207 O GLU A 31 -6.657 -12.710 20.026 1.00 60.91 O

ATOM 208 CB GLU A 31 -4.774 -15.514 20.083 1.00 58.37 C

ATOM 209 CG GLU A 31 -3.807 -15.682 21.252 1.00 74.12 C

ATOM 210 CD GLU A 31 -4.047 -16.858 22.184 1.00103.99 C

ATOM 211 OE1 GLU A 31 -5.113 -17.509 22.083 1.00100.46 O

ATOM 212 OE2 GLU A 31 -3.171 -17.106 23.044 1.00103.64 O

ATOM 213 N TYR A 32 -4.857 -12.598 21.426 1.00 54.97 N

ATOM 214 CA TYR A 32 -5.517 -11.594 22.262 1.00 53.78 C

ATOM 215 C TYR A 32 -5.366 -10.188 21.694 1.00 49.03 C

ATOM 216 O TYR A 32 -4.346 -9.526 21.893 1.00 47.38 O

ATOM 217 CB TYR A 32 -5.164 -11.637 23.771 1.00 56.98 C

ATOM 218 CG TYR A 32 -6.023 -10.655 24.549 1.00 61.10 C

ATOM 219 CD1 TYR A 32 -7.368 -10.920 24.795 1.00 62.90 C

ATOM 220 CD2 TYR A 32 -5.537 -9.394 24.894 1.00 63.34 C

ATOM 221 CE1 TYR A 32 -8.191 -9.980 25.417 1.00 64.48 C

ATOM 222 CE2 TYR A 32 -6.355 -8.441 25.502 1.00 64.18 C

ATOM 223 CZ TYR A 32 -7.679 -8.741 25.768 1.00 72.55 C

ATOM 224 OH TYR A 32 -8.480 -7.814 26.389 1.00 75.10 O

ATOM 225 N ILE A 33 -6.432 -9.743 21.021 1.00 40.85 N

ATOM 226 CA ILE A 33 -6.607 -8.421 20.424 1.00 38.10 C

ATOM 227 C ILE A 33 -7.422 -7.615 21.447 1.00 38.70 C

ATOM 228 O ILE A 33 -8.402 -8.159 21.960 1.00 39.15 O

ATOM 229 CB ILE A 33 -7.379 -8.561 19.077 1.00 40.38 C

ATOM 230 CG1 ILE A 33 -6.716 -9.592 18.139 1.00 41.11 C

ATOM 231 CG2 ILE A 33 -7.563 -7.224 18.370 1.00 39.62 C

ATOM 232 CD1 ILE A 33 -7.680 -10.614 17.539 1.00 45.52 C

ATOM 233 N PRO A 34 -7.073 -6.343 21.777 1.00 32.45 N

ATOM 234 CA PRO A 34 -7.923 -5.578 22.715 1.00 30.68 C

ATOM 235 C PRO A 34 -9.373 -5.517 22.231 1.00 29.98 C

ATOM 236 O PRO A 34 -9.641 -5.416 21.031 1.00 26.43 O

ATOM 237 CB PRO A 34 -7.282 -4.182 22.742 1.00 32.59 C

ATOM 238 CG PRO A 34 -6.419 -4.117 21.529 1.00 37.45 C

ATOM 239 CD PRO A 34 -5.949 -5.519 21.278 1.00 33.39 C

ATOM 240 N THR A 35 -10.307 -5.625 23.174 1.00 27.16 N

ATOM 241 CA THR A 35 -11.735 -5.654 22.857 1.00 26.36 C

ATOM 242 C THR A 35 -12.388 -4.271 23.026 1.00 28.68 C

ATOM 243 O THR A 35 -13.414 -4.010 22.410 1.00 28.20 O

ATOM 244 CB THR A 35 -12.411 -6.772 23.637 1.00 28.95 C

ATOM 245 OG1 THR A 35 -12.150 -6.562 25.026 1.00 35.74 O

ATOM 246 CG2 THR A 35 -11.901 -8.151 23.221 1.00 17.78 C

ATOM 247 N VAL A 36 -11.760 -3.375 23.795 1.00 25.06 N

ATOM 248 CA VAL A 36 -12.232 -1.996 23.974 1.00 24.60 C

ATOM 249 C VAL A 36 -11.975 -1.234 22.668 1.00 27.70 C

ATOM 250 O VAL A 36 -10.835 -1.230 22.178 1.00 28.23 O

ATOM 251 CB VAL A 36 -11.548 -1.303 25.194 1.00 28.07 C

ATOM 252 CG1 VAL A 36 -11.923 0.180 25.284 1.00 27.61 C

ATOM 253 CG2 VAL A 36 -11.888 -2.027 26.498 1.00 27.75 C

ATOM 254 N PHE A 37 -13.023 -0.620 22.086 1.00 22.24 N

ATOM 255 CA PHE A 37 -12.844 0.148 20.852 1.00 21.90 C

ATOM 256 C PHE A 37 -12.183 1.460 21.232 1.00 28.40 C

ATOM 257 O PHE A 37 -12.756 2.263 21.964 1.00 29.62 O

ATOM 258 CB PHE A 37 -14.155 0.356 20.085 1.00 23.06 C

ATOM 259 CG PHE A 37 -14.915 -0.909 19.760 1.00 23.76 C

ATOM 260 CD1 PHE A 37 -14.669 -1.606 18.576 1.00 25.40 C

ATOM 261 CD2 PHE A 37 -15.901 -1.392 20.623 1.00 23.37 C

ATOM 262 CE1 PHE A 37 -15.387 -2.770 18.270 1.00 24.84 C

ATOM 263 CE2 PHE A 37 -16.622 -2.544 20.308 1.00 25.14 C

ATOM 264 CZ PHE A 37 -16.363 -3.220 19.131 1.00 23.24 C

ATOM 265 N ASP A 38 -10.941 1.624 20.830 1.00 25.05 N

ATOM 266 CA ASP A 38 -10.191 2.781 21.232 1.00 25.67 C

ATOM 267 C ASP A 38 -9.717 3.603 20.051 1.00 29.81 C

ATOM 268 O ASP A 38 -10.070 3.326 18.907 1.00 29.42 O

ATOM 269 CB ASP A 38 -9.002 2.314 22.104 1.00 28.51 C

ATOM 270 CG ASP A 38 -8.596 3.254 23.238 1.00 45.62 C

ATOM 271 OD1 ASP A 38 -9.012 4.441 23.214 1.00 46.49 O

ATOM 272 OD2 ASP A 38 -7.824 2.818 24.119 1.00 54.24 O

ATOM 273 N ASN A 39 -8.966 4.663 20.357 1.00 26.75 N

ATOM 274 CA ASN A 39 -8.305 5.552 19.418 1.00 26.03 C

ATOM 275 C ASN A 39 -6.831 5.464 19.707 1.00 26.87 C

ATOM 276 O ASN A 39 -6.425 5.254 20.842 1.00 24.48 O

ATOM 277 CB ASN A 39 -8.800 6.999 19.526 1.00 29.94 C

ATOM 278 CG ASN A 39 -8.790 7.600 20.915 1.00 57.35 C

ATOM 279 OD1 ASN A 39 -9.794 7.567 21.638 1.00 50.08 O

ATOM 280 ND2 ASN A 39 -7.668 8.208 21.299 1.00 49.62 N

ATOM 281 N TYR A 40 -6.036 5.612 18.682 1.00 24.82 N

ATOM 282 CA TYR A 40 -4.586 5.507 18.758 1.00 24.20 C

ATOM 283 C TYR A 40 -3.947 6.764 18.173 1.00 27.69 C

ATOM 284 O TYR A 40 -4.649 7.622 17.641 1.00 25.92 O

ATOM 285 CB TYR A 40 -4.126 4.220 18.046 1.00 24.74 C

ATOM 286 CG TYR A 40 -4.869 2.992 18.530 1.00 26.90 C

ATOM 287 CD1 TYR A 40 -4.534 2.380 19.737 1.00 28.84 C

ATOM 288 CD2 TYR A 40 -5.968 2.497 17.832 1.00 27.70 C

ATOM 289 CE1 TYR A 40 -5.228 1.264 20.200 1.00 29.79 C

ATOM 290 CE2 TYR A 40 -6.673 1.382 18.287 1.00 28.65 C

ATOM 291 CZ TYR A 40 -6.295 0.764 19.468 1.00 35.12 C

ATOM 292 OH TYR A 40 -6.985 -0.335 19.918 1.00 32.27 O

ATOM 293 N SER A 41 -2.631 6.888 18.313 1.00 25.66 N

ATOM 294 CA SER A 41 -1.900 8.057 17.855 1.00 25.99 C

ATOM 295 C SER A 41 -0.550 7.663 17.305 1.00 29.48 C

ATOM 296 O SER A 41 0.046 6.699 17.775 1.00 28.66 O

ATOM 297 CB SER A 41 -1.715 9.039 19.014 1.00 29.27 C

ATOM 298 OG SER A 41 -1.047 10.224 18.615 1.00 35.80 O

ATOM 299 N ALA A 42 -0.058 8.432 16.338 1.00 27.46 N

ATOM 300 CA ALA A 42 1.277 8.256 15.782 1.00 29.18 C

ATOM 301 C ALA A 42 1.875 9.613 15.359 1.00 37.66 C

ATOM 302 O ALA A 42 1.262 10.338 14.568 1.00 37.59 O

ATOM 303 CB ALA A 42 1.254 7.280 14.615 1.00 29.84 C

ATOM 304 N ASN A 43 3.040 9.972 15.931 1.00 37.14 N

ATOM 305 CA ASN A 43 3.778 11.188 15.569 1.00 38.38 C

ATOM 306 C ASN A 43 4.694 10.793 14.428 1.00 47.90 C

ATOM 307 O ASN A 43 5.680 10.075 14.634 1.00 47.49 O

ATOM 308 CB ASN A 43 4.594 11.723 16.741 1.00 36.60 C

ATOM 309 CG ASN A 43 3.758 12.302 17.840 1.00 58.04 C

ATOM 310 OD1 ASN A 43 2.948 13.210 17.630 1.00 54.58 O

ATOM 311 ND2 ASN A 43 3.995 11.840 19.053 1.00 48.76 N

ATOM 312 N VAL A 44 4.324 11.185 13.212 1.00 49.18 N

ATOM 313 CA VAL A 44 5.079 10.826 12.018 1.00 51.23 C

ATOM 314 C VAL A 44 5.333 12.015 11.119 1.00 59.56 C

ATOM 315 O VAL A 44 4.470 12.875 10.978 1.00 59.24 O

ATOM 316 CB VAL A 44 4.444 9.638 11.245 1.00 55.69 C

ATOM 317 CG1 VAL A 44 4.464 8.368 12.089 1.00 56.00 C

ATOM 318 CG2 VAL A 44 3.023 9.955 10.784 1.00 55.38 C

ATOM 319 N MET A 45 6.523 12.069 10.515 1.00 59.88 N

ATOM 320 CA MET A 45 6.878 13.139 9.588 1.00 61.29 C

ATOM 321 C MET A 45 7.274 12.568 8.238 1.00 68.13 C

ATOM 322 O MET A 45 8.344 11.978 8.107 1.00 68.02 O

ATOM 323 CB MET A 45 7.928 14.105 10.170 1.00 63.87 C

ATOM 324 CG MET A 45 9.304 13.501 10.365 0.00 67.86 C

ATOM 325 SD MET A 45 10.338 14.574 11.386 0.00 72.65 S

ATOM 326 CE MET A 45 9.908 13.980 13.001 0.00 69.33 C

ATOM 327 N VAL A 46 6.363 12.668 7.253 1.00 67.17 N

ATOM 328 CA VAL A 46 6.588 12.178 5.889 1.00 67.70 C

ATOM 329 C VAL A 46 6.893 13.386 4.975 1.00 73.00 C

ATOM 330 O VAL A 46 5.986 14.134 4.594 1.00 73.06 O

ATOM 331 CB VAL A 46 5.481 11.207 5.365 0.00 71.66 C

ATOM 332 CG1 VAL A 46 4.078 11.817 5.432 0.00 71.48 C

ATOM 333 CG2 VAL A 46 5.800 10.689 3.966 0.00 71.49 C

ATOM 334 N ASP A 47 8.206 13.588 4.693 1.00 69.57 N

ATOM 335 CA ASP A 47 8.820 14.672 3.906 1.00 69.18 C

ATOM 336 C ASP A 47 8.263 16.072 4.284 1.00 71.81 C

ATOM 337 O ASP A 47 7.685 16.785 3.453 1.00 71.42 O

ATOM 338 CB ASP A 47 8.796 14.410 2.380 0.00 70.98 C

ATOM 339 CG ASP A 47 7.461 14.018 1.779 0.00 80.64 C

ATOM 340 OD1 ASP A 47 6.644 14.923 1.510 0.00 81.07 O

ATOM 341 OD2 ASP A 47 7.252 12.811 1.539 0.00 86.46 O

ATOM 342 N GLY A 48 8.459 16.431 5.552 1.00 66.66 N

ATOM 343 CA GLY A 48 8.025 17.705 6.104 1.00 65.73 C

ATOM 344 C GLY A 48 8.401 17.864 7.557 1.00 68.36 C

ATOM 345 O GLY A 48 9.527 17.548 7.955 1.00 67.75 O

ATOM 346 N LYS A 49 7.449 18.371 8.352 1.00 63.92 N

ATOM 347 CA LYS A 49 7.578 18.594 9.795 1.00 62.76 C

ATOM 348 C LYS A 49 6.695 17.570 10.558 1.00 63.67 C

ATOM 349 O LYS A 49 5.936 16.854 9.890 1.00 62.95 O

ATOM 350 CB LYS A 49 7.218 20.054 10.137 0.00 65.36 C

ATOM 351 CG LYS A 49 8.377 21.018 9.908 0.00 80.31 C

ATOM 352 CD LYS A 49 8.099 22.404 10.462 0.00 90.85 C

ATOM 353 CE LYS A 49 9.290 23.315 10.295 0.00102.35 C

ATOM 354 NZ LYS A 49 9.033 24.669 10.853 0.00111.76 N

ATOM 355 N PRO A 50 6.796 17.424 11.914 1.00 58.23 N

ATOM 356 CA PRO A 50 5.971 16.404 12.604 1.00 57.01 C

ATOM 357 C PRO A 50 4.458 16.556 12.439 1.00 56.67 C

ATOM 358 O PRO A 50 3.924 17.673 12.403 1.00 56.49 O

ATOM 359 CB PRO A 50 6.392 16.516 14.077 1.00 58.99 C

ATOM 360 CG PRO A 50 7.702 17.223 14.060 1.00 63.99 C

ATOM 361 CD PRO A 50 7.664 18.141 12.875 1.00 59.63 C

ATOM 362 N VAL A 51 3.777 15.415 12.316 1.00 48.75 N

ATOM 363 CA VAL A 51 2.329 15.343 12.160 1.00 46.75 C

ATOM 364 C VAL A 51 1.804 14.286 13.138 1.00 47.14 C

ATOM 365 O VAL A 51 2.350 13.185 13.189 1.00 46.48 O

ATOM 366 CB VAL A 51 1.945 15.026 10.684 1.00 50.53 C

ATOM 367 CG1 VAL A 51 0.535 14.464 10.565 1.00 50.05 C

ATOM 368 CG2 VAL A 51 2.124 16.244 9.783 1.00 50.47 C

ATOM 369 N ASN A 52 0.765 14.625 13.922 1.00 41.56 N

ATOM 370 CA ASN A 52 0.142 13.676 14.837 1.00 40.45 C

ATOM 371 C ASN A 52 -1.114 13.082 14.193 1.00 42.66 C

ATOM 372 O ASN A 52 -2.078 13.796 13.900 1.00 41.81 O

ATOM 373 CB ASN A 52 -0.165 14.292 16.200 1.00 39.87 C

ATOM 374 CG ASN A 52 -0.652 13.262 17.178 1.00 57.82 C

ATOM 375 OD1 ASN A 52 -1.852 13.112 17.397 1.00 51.52 O

ATOM 376 ND2 ASN A 52 0.260 12.451 17.692 1.00 49.50 N

ATOM 377 N LEU A 53 -1.080 11.774 13.957 1.00 38.02 N

ATOM 378 CA LEU A 53 -2.176 11.056 13.331 1.00 37.13 C

ATOM 379 C LEU A 53 -3.036 10.322 14.364 1.00 37.79 C

ATOM 380 O LEU A 53 -2.641 9.278 14.881 1.00 38.40 O

ATOM 381 CB LEU A 53 -1.621 10.098 12.244 1.00 37.47 C

ATOM 382 CG LEU A 53 -2.608 9.242 11.424 1.00 42.60 C

ATOM 383 CD1 LEU A 53 -3.575 10.104 10.603 1.00 43.04 C

ATOM 384 CD2 LEU A 53 -1.860 8.315 10.491 1.00 45.63 C

ATOM 385 N GLY A 54 -4.199 10.888 14.647 1.00 31.04 N

ATOM 386 CA GLY A 54 -5.206 10.295 15.515 1.00 29.60 C

ATOM 387 C GLY A 54 -5.941 9.234 14.720 1.00 31.25 C

ATOM 388 O GLY A 54 -6.505 9.528 13.662 1.00 30.50 O

ATOM 389 N LEU A 55 -5.866 7.980 15.176 1.00 27.11 N

ATOM 390 CA LEU A 55 -6.452 6.820 14.494 1.00 26.24 C

ATOM 391 C LEU A 55 -7.630 6.328 15.269 1.00 30.07 C

ATOM 392 O LEU A 55 -7.501 6.036 16.448 1.00 29.12 O

ATOM 393 CB LEU A 55 -5.401 5.714 14.348 1.00 26.00 C

ATOM 394 CG LEU A 55 -4.180 6.100 13.521 1.00 30.15 C

ATOM 395 CD1 LEU A 55 -2.931 5.446 14.025 1.00 29.85 C

ATOM 396 CD2 LEU A 55 -4.408 5.851 12.079 1.00 32.07 C

ATOM 397 N TRP A 56 -8.793 6.275 14.631 1.00 28.37 N

ATOM 398 CA TRP A 56 -10.020 5.880 15.310 1.00 28.59 C

ATOM 399 C TRP A 56 -10.575 4.562 14.831 1.00 29.62 C

ATOM 400 O TRP A 56 -10.865 4.403 13.652 1.00 29.63 O

ATOM 401 CB TRP A 56 -11.054 7.007 15.217 1.00 28.43 C

ATOM 402 CG TRP A 56 -10.617 8.238 15.956 1.00 30.47 C

ATOM 403 CD1 TRP A 56 -9.716 9.174 15.538 1.00 33.49 C

ATOM 404 CD2 TRP A 56 -11.007 8.619 17.278 1.00 31.02 C

ATOM 405 NE1 TRP A 56 -9.528 10.119 16.516 1.00 33.29 N

ATOM 406 CE2 TRP A 56 -10.321 9.812 17.591 1.00 35.17 C

ATOM 407 CE3 TRP A 56 -11.880 8.066 18.236 1.00 33.00 C

ATOM 408 CZ2 TRP A 56 -10.464 10.454 18.828 1.00 35.15 C

ATOM 409 CZ3 TRP A 56 -12.026 8.707 19.459 1.00 34.67 C

ATOM 410 CH2 TRP A 56 -11.329 9.889 19.743 1.00 35.32 C

ATOM 411 N ASP A 57 -10.675 3.601 15.741 1.00 25.15 N

ATOM 412 CA ASP A 57 -11.258 2.304 15.444 1.00 25.01 C

ATOM 413 C ASP A 57 -12.745 2.374 15.685 1.00 29.61 C

ATOM 414 O ASP A 57 -13.219 3.148 16.519 1.00 29.79 O

ATOM 415 CB ASP A 57 -10.671 1.209 16.329 1.00 27.25 C

ATOM 416 CG ASP A 57 -10.842 -0.185 15.756 1.00 34.91 C

ATOM 417 OD1 ASP A 57 -11.251 -0.297 14.573 1.00 35.37 O

ATOM 418 OD2 ASP A 57 -10.562 -1.165 16.486 1.00 34.97 O

ATOM 419 N THR A 58 -13.489 1.562 14.952 1.00 26.55 N

ATOM 420 CA THR A 58 -14.932 1.525 15.070 1.00 25.50 C

ATOM 421 C THR A 58 -15.424 0.125 15.317 1.00 30.05 C

ATOM 422 O THR A 58 -14.744 -0.875 15.051 1.00 28.27 O

ATOM 423 CB THR A 58 -15.615 2.084 13.811 1.00 29.70 C

ATOM 424 OG1 THR A 58 -15.179 1.336 12.682 1.00 30.49 O

ATOM 425 CG2 THR A 58 -15.378 3.578 13.604 1.00 27.63 C

ATOM 426 N ALA A 59 -16.648 0.082 15.819 1.00 28.68 N

ATOM 427 CA ALA A 59 -17.432 -1.094 16.072 1.00 28.17 C

ATOM 428 C ALA A 59 -18.210 -1.297 14.753 1.00 30.69 C

ATOM 429 O ALA A 59 -19.104 -0.511 14.418 1.00 29.73 O

ATOM 430 CB ALA A 59 -18.349 -0.825 17.253 1.00 28.79 C

ATOM 431 N GLY A 60 -17.749 -2.277 13.974 1.00 27.40 N

ATOM 432 CA GLY A 60 -18.240 -2.591 12.634 1.00 27.50 C

ATOM 433 C GLY A 60 -19.541 -3.352 12.500 1.00 32.27 C

ATOM 434 O GLY A 60 -20.196 -3.241 11.457 1.00 31.63 O

ATOM 435 N GLN A 61 -19.927 -4.135 13.541 1.00 30.49 N

ATOM 436 CA GLN A 61 -21.155 -4.956 13.574 1.00 31.37 C

ATOM 437 C GLN A 61 -22.465 -4.135 13.327 1.00 37.06 C

ATOM 438 O GLN A 61 -22.484 -2.921 13.541 1.00 36.06 O

ATOM 439 CB GLN A 61 -21.225 -5.773 14.885 1.00 32.61 C

ATOM 440 CG GLN A 61 -22.185 -6.969 14.881 1.00 58.97 C

ATOM 441 CD GLN A 61 -22.572 -7.459 16.267 1.00 81.26 C

ATOM 442 OE1 GLN A 61 -22.734 -6.685 17.219 1.00 79.42 O

ATOM 443 NE2 GLN A 61 -22.797 -8.760 16.396 1.00 70.42 N

ATOM 444 N GLU A 62 -23.537 -4.818 12.854 1.00 34.87 N

ATOM 445 CA GLU A 62 -24.870 -4.269 12.565 1.00 35.31 C

ATOM 446 C GLU A 62 -25.474 -3.532 13.750 1.00 37.73 C

ATOM 447 O GLU A 62 -26.170 -2.547 13.546 1.00 35.98 O

ATOM 448 CB GLU A 62 -25.828 -5.404 12.161 1.00 37.47 C

ATOM 449 CG GLU A 62 -26.116 -5.487 10.671 1.00 53.69 C

ATOM 450 CD GLU A 62 -26.849 -6.749 10.249 1.00 79.35 C

ATOM 451 OE1 GLU A 62 -26.223 -7.597 9.573 1.00 73.01 O

ATOM 452 OE2 GLU A 62 -28.042 -6.898 10.602 1.00 73.70 O

ATOM 453 N ASP A 63 -25.223 -4.020 14.980 1.00 35.85 N

ATOM 454 CA ASP A 63 -25.732 -3.448 16.233 1.00 36.33 C

ATOM 455 C ASP A 63 -25.321 -1.988 16.507 1.00 40.73 C

ATOM 456 O ASP A 63 -26.037 -1.271 17.222 1.00 39.08 O

ATOM 457 CB ASP A 63 -25.340 -4.339 17.422 1.00 38.02 C

ATOM 458 CG ASP A 63 -26.049 -5.679 17.472 1.00 48.11 C

ATOM 459 OD1 ASP A 63 -26.965 -5.901 16.644 1.00 47.44 O

ATOM 460 OD2 ASP A 63 -25.711 -6.498 18.361 1.00 55.19 O

ATOM 461 N TYR A 64 -24.182 -1.558 15.936 1.00 38.48 N

ATOM 462 CA TYR A 64 -23.634 -0.219 16.123 1.00 39.30 C

ATOM 463 C TYR A 64 -23.913 0.731 14.976 1.00 42.25 C

ATOM 464 O TYR A 64 -23.342 1.823 14.966 1.00 42.07 O

ATOM 465 CB TYR A 64 -22.131 -0.279 16.425 1.00 42.26 C

ATOM 466 CG TYR A 64 -21.737 -1.528 17.171 1.00 47.23 C

ATOM 467 CD1 TYR A 64 -22.080 -1.702 18.507 1.00 50.17 C

ATOM 468 CD2 TYR A 64 -21.072 -2.567 16.527 1.00 48.85 C

ATOM 469 CE1 TYR A 64 -21.787 -2.888 19.177 1.00 53.14 C

ATOM 470 CE2 TYR A 64 -20.700 -3.721 17.212 1.00 50.26 C

ATOM 471 CZ TYR A 64 -21.075 -3.887 18.533 1.00 62.03 C

ATOM 472 OH TYR A 64 -20.740 -5.045 19.193 1.00 68.84 O

ATOM 473 N ASP A 65 -24.825 0.360 14.047 1.00 38.19 N

ATOM 474 CA ASP A 65 -25.179 1.180 12.882 1.00 38.32 C

ATOM 475 C ASP A 65 -25.641 2.613 13.230 1.00 41.59 C

ATOM 476 O ASP A 65 -25.360 3.539 12.464 1.00 42.07 O

ATOM 477 CB ASP A 65 -26.214 0.472 11.987 1.00 40.71 C

ATOM 478 CG ASP A 65 -25.664 -0.649 11.103 1.00 57.81 C

ATOM 479 OD1 ASP A 65 -24.524 -1.100 11.350 1.00 60.63 O

ATOM 480 OD2 ASP A 65 -26.402 -1.117 10.203 1.00 63.18 O

ATOM 481 N ARG A 66 -26.313 2.799 14.380 1.00 36.43 N

ATOM 482 CA ARG A 66 -26.810 4.114 14.814 1.00 35.58 C

ATOM 483 C ARG A 66 -25.774 4.926 15.608 1.00 36.57 C

ATOM 484 O ARG A 66 -25.769 6.155 15.529 1.00 35.51 O

ATOM 485 CB ARG A 66 -28.106 3.968 15.641 1.00 36.63 C

ATOM 486 CG ARG A 66 -29.273 3.290 14.927 1.00 52.49 C

ATOM 487 CD ARG A 66 -30.132 4.277 14.164 1.00 75.08 C

ATOM 488 NE ARG A 66 -31.173 3.608 13.379 1.00 97.82 N

ATOM 489 CZ ARG A 66 -31.007 3.141 12.143 1.00120.30 C

ATOM 490 NH1 ARG A 66 -29.828 3.244 11.537 1.00109.65 N

ATOM 491 NH2 ARG A 66 -32.015 2.558 11.507 1.00109.91 N

ATOM 492 N LEU A 67 -24.901 4.246 16.364 1.00 31.29 N

ATOM 493 CA LEU A 67 -23.926 4.904 17.225 1.00 30.32 C

ATOM 494 C LEU A 67 -22.566 5.136 16.603 1.00 35.30 C

ATOM 495 O LEU A 67 -21.854 6.035 17.055 1.00 36.19 O

ATOM 496 CB LEU A 67 -23.776 4.143 18.554 1.00 29.99 C

ATOM 497 CG LEU A 67 -25.040 3.986 19.421 1.00 33.19 C

ATOM 498 CD1 LEU A 67 -24.742 3.180 20.625 1.00 32.69 C

ATOM 499 CD2 LEU A 67 -25.615 5.325 19.828 1.00 33.13 C

ATOM 500 N ARG A 68 -22.174 4.310 15.618 1.00 31.05 N

ATOM 501 CA ARG A 68 -20.888 4.407 14.914 1.00 29.84 C

ATOM 502 C ARG A 68 -20.669 5.804 14.276 1.00 34.12 C

ATOM 503 O ARG A 68 -19.603 6.378 14.518 1.00 35.65 O

ATOM 504 CB ARG A 68 -20.774 3.297 13.859 1.00 26.58 C

ATOM 505 CG ARG A 68 -19.436 3.169 13.159 1.00 26.21 C

ATOM 506 CD ARG A 68 -19.544 2.205 11.990 1.00 32.60 C

ATOM 507 NE ARG A 68 -20.246 0.965 12.344 1.00 30.78 N

ATOM 508 CZ ARG A 68 -21.341 0.519 11.738 1.00 42.91 C

ATOM 509 NH1 ARG A 68 -21.868 1.192 10.717 1.00 17.13 N

ATOM 510 NH2 ARG A 68 -21.913 -0.608 12.137 1.00 32.42 N

ATOM 511 N PRO A 69 -21.634 6.408 13.521 1.00 28.32 N

ATOM 512 CA PRO A 69 -21.361 7.715 12.913 1.00 27.52 C

ATOM 513 C PRO A 69 -21.123 8.869 13.890 1.00 30.97 C

ATOM 514 O PRO A 69 -20.705 9.937 13.440 1.00 31.25 O

ATOM 515 CB PRO A 69 -22.578 7.940 12.016 1.00 28.88 C

ATOM 516 CG PRO A 69 -23.079 6.567 11.729 1.00 33.09 C

ATOM 517 CD PRO A 69 -22.946 5.911 13.059 1.00 29.19 C

ATOM 518 N LEU A 70 -21.311 8.644 15.217 1.00 25.58 N

ATOM 519 CA LEU A 70 -21.053 9.666 16.234 1.00 24.52 C

ATOM 520 C LEU A 70 -19.550 10.010 16.349 1.00 28.73 C

ATOM 521 O LEU A 70 -19.207 11.123 16.781 1.00 27.61 O

ATOM 522 CB LEU A 70 -21.633 9.272 17.601 1.00 24.04 C

ATOM 523 CG LEU A 70 -23.151 9.241 17.711 1.00 26.95 C

ATOM 524 CD1 LEU A 70 -23.573 8.655 19.030 1.00 26.34 C

ATOM 525 CD2 LEU A 70 -23.751 10.616 17.498 1.00 27.42 C

ATOM 526 N SER A 71 -18.664 9.059 15.927 1.00 24.69 N

ATOM 527 CA SER A 71 -17.206 9.231 15.911 1.00 24.38 C

ATOM 528 C SER A 71 -16.716 9.898 14.612 1.00 30.13 C

ATOM 529 O SER A 71 -15.572 10.348 14.551 1.00 29.15 O

ATOM 530 CB SER A 71 -16.490 7.902 16.140 1.00 28.26 C

ATOM 531 OG SER A 71 -17.143 6.791 15.544 1.00 43.46 O

ATOM 532 N TYR A 72 -17.587 9.980 13.585 1.00 27.84 N

ATOM 533 CA TYR A 72 -17.245 10.576 12.294 1.00 27.71 C

ATOM 534 C TYR A 72 -17.009 12.102 12.344 1.00 33.95 C

ATOM 535 O TYR A 72 -16.028 12.528 11.746 1.00 34.53 O

ATOM 536 CB TYR A 72 -18.267 10.222 11.190 1.00 28.03 C

ATOM 537 CG TYR A 72 -18.449 8.747 10.861 1.00 28.61 C

ATOM 538 CD1 TYR A 72 -17.593 7.775 11.382 1.00 30.14 C

ATOM 539 CD2 TYR A 72 -19.461 8.328 10.006 1.00 28.89 C

ATOM 540 CE1 TYR A 72 -17.770 6.423 11.089 1.00 30.83 C

ATOM 541 CE2 TYR A 72 -19.643 6.983 9.700 1.00 29.38 C

ATOM 542 CZ TYR A 72 -18.797 6.033 10.240 1.00 36.04 C

ATOM 543 OH TYR A 72 -19.010 4.713 9.919 1.00 33.72 O

ATOM 544 N PRO A 73 -17.809 12.957 13.034 1.00 30.64 N

ATOM 545 CA PRO A 73 -17.529 14.407 12.992 1.00 30.50 C

ATOM 546 C PRO A 73 -16.085 14.827 13.270 1.00 36.43 C

ATOM 547 O PRO A 73 -15.447 14.276 14.167 1.00 35.73 O

ATOM 548 CB PRO A 73 -18.499 14.969 14.021 1.00 31.86 C

ATOM 549 CG PRO A 73 -19.664 14.053 13.917 1.00 36.20 C

ATOM 550 CD PRO A 73 -19.047 12.695 13.799 1.00 31.71 C

ATOM 551 N GLN A 74 -15.576 15.801 12.461 1.00 35.22 N

ATOM 552 CA GLN A 74 -14.221 16.397 12.486 1.00 35.46 C

ATOM 553 C GLN A 74 -13.150 15.505 11.823 1.00 38.44 C

ATOM 554 O GLN A 74 -11.957 15.822 11.897 1.00 38.24 O

ATOM 555 CB GLN A 74 -13.784 16.826 13.912 1.00 37.51 C

ATOM 556 CG GLN A 74 -14.682 17.875 14.578 1.00 62.26 C

ATOM 557 CD GLN A 74 -14.362 18.043 16.048 1.00 88.25 C

ATOM 558 OE1 GLN A 74 -14.514 17.118 16.857 1.00 85.61 O

ATOM 559 NE2 GLN A 74 -13.940 19.239 16.437 1.00 81.51 N

ATOM 560 N THR A 75 -13.569 14.402 11.170 1.00 34.52 N

ATOM 561 CA THR A 75 -12.645 13.464 10.519 1.00 34.20 C

ATOM 562 C THR A 75 -11.996 14.085 9.278 1.00 37.35 C

ATOM 563 O THR A 75 -12.664 14.735 8.470 1.00 37.84 O

ATOM 564 CB THR A 75 -13.295 12.090 10.240 1.00 38.69 C

ATOM 565 OG1 THR A 75 -12.387 11.269 9.500 1.00 32.08 O

ATOM 566 CG2 THR A 75 -14.568 12.203 9.455 1.00 40.62 C

ATOM 567 N ASP A 76 -10.691 13.860 9.138 1.00 31.90 N

ATOM 568 CA ASP A 76 -9.897 14.376 8.039 1.00 30.59 C

ATOM 569 C ASP A 76 -9.817 13.402 6.864 1.00 33.18 C

ATOM 570 O ASP A 76 -9.770 13.855 5.718 1.00 32.34 O

ATOM 571 CB ASP A 76 -8.504 14.775 8.546 1.00 32.07 C

ATOM 572 CG ASP A 76 -8.552 15.893 9.572 1.00 45.07 C

ATOM 573 OD1 ASP A 76 -8.885 17.044 9.185 1.00 48.40 O

ATOM 574 OD2 ASP A 76 -8.295 15.615 10.773 1.00 45.10 O

ATOM 575 N VAL A 77 -9.794 12.072 7.148 1.00 28.77 N

ATOM 576 CA VAL A 77 -9.682 10.986 6.155 1.00 27.86 C

ATOM 577 C VAL A 77 -10.280 9.666 6.687 1.00 29.19 C

ATOM 578 O VAL A 77 -10.262 9.426 7.886 1.00 28.95 O

ATOM 579 CB VAL A 77 -8.207 10.831 5.648 1.00 32.44 C

ATOM 580 CG1 VAL A 77 -7.255 10.365 6.758 1.00 32.37 C

ATOM 581 CG2 VAL A 77 -8.108 9.924 4.423 1.00 32.42 C

ATOM 582 N PHE A 78 -10.797 8.824 5.795 1.00 25.08 N

ATOM 583 CA PHE A 78 -11.381 7.516 6.118 1.00 25.12 C

ATOM 584 C PHE A 78 -10.591 6.363 5.516 1.00 27.63 C

ATOM 585 O PHE A 78 -10.160 6.458 4.362 1.00 27.43 O

ATOM 586 CB PHE A 78 -12.822 7.411 5.561 1.00 27.07 C

ATOM 587 CG PHE A 78 -13.899 7.974 6.448 1.00 28.33 C

ATOM 588 CD1 PHE A 78 -14.237 9.321 6.388 1.00 30.69 C

ATOM 589 CD2 PHE A 78 -14.569 7.163 7.355 1.00 29.98 C

ATOM 590 CE1 PHE A 78 -15.224 9.846 7.217 1.00 30.99 C

ATOM 591 CE2 PHE A 78 -15.544 7.695 8.200 1.00 32.36 C

ATOM 592 CZ PHE A 78 -15.860 9.035 8.132 1.00 30.08 C

ATOM 593 N LEU A 79 -10.492 5.245 6.256 1.00 23.04 N

ATOM 594 CA LEU A 79 -9.926 3.996 5.754 1.00 22.79 C

ATOM 595 C LEU A 79 -11.052 2.986 5.732 1.00 26.59 C

ATOM 596 O LEU A 79 -11.486 2.507 6.779 1.00 25.32 O

ATOM 597 CB LEU A 79 -8.774 3.435 6.602 1.00 22.98 C

ATOM 598 CG LEU A 79 -7.491 4.235 6.739 1.00 27.71 C

ATOM 599 CD1 LEU A 79 -6.481 3.450 7.525 1.00 27.73 C

ATOM 600 CD2 LEU A 79 -6.907 4.642 5.387 1.00 29.10 C

ATOM 601 N ILE A 80 -11.571 2.710 4.549 1.00 24.63 N

ATOM 602 CA ILE A 80 -12.612 1.712 4.393 1.00 24.87 C

ATOM 603 C ILE A 80 -11.841 0.414 4.227 1.00 29.13 C

ATOM 604 O ILE A 80 -11.003 0.301 3.333 1.00 27.55 O

ATOM 605 CB ILE A 80 -13.595 2.027 3.221 1.00 28.00 C

ATOM 606 CG1 ILE A 80 -14.298 3.411 3.452 1.00 27.95 C

ATOM 607 CG2 ILE A 80 -14.609 0.859 3.033 1.00 27.68 C

ATOM 608 CD1 ILE A 80 -15.478 3.756 2.550 1.00 32.49 C

ATOM 609 N CYS A 81 -12.069 -0.532 5.137 1.00 27.17 N

ATOM 610 CA CYS A 81 -11.339 -1.787 5.129 1.00 27.50 C

ATOM 611 C CYS A 81 -12.183 -2.978 4.767 1.00 30.38 C

ATOM 612 O CYS A 81 -13.375 -3.019 5.092 1.00 30.29 O

ATOM 613 CB CYS A 81 -10.621 -1.996 6.455 1.00 28.22 C

ATOM 614 SG CYS A 81 -9.472 -0.666 6.865 1.00 32.59 S

ATOM 615 N PHE A 82 -11.547 -3.953 4.084 1.00 24.63 N

ATOM 616 CA PHE A 82 -12.159 -5.222 3.697 1.00 23.37 C

ATOM 617 C PHE A 82 -11.106 -6.317 3.663 1.00 27.53 C

ATOM 618 O PHE A 82 -9.943 -6.061 3.320 1.00 26.63 O

ATOM 619 CB PHE A 82 -12.910 -5.119 2.348 1.00 24.45 C

ATOM 620 CG PHE A 82 -12.044 -5.008 1.117 1.00 24.78 C

ATOM 621 CD1 PHE A 82 -11.594 -6.147 0.456 1.00 27.49 C

ATOM 622 CD2 PHE A 82 -11.684 -3.769 0.612 1.00 25.30 C

ATOM 623 CE1 PHE A 82 -10.767 -6.042 -0.669 1.00 27.44 C

ATOM 624 CE2 PHE A 82 -10.863 -3.667 -0.520 1.00 27.52 C

ATOM 625 CZ PHE A 82 -10.421 -4.804 -1.156 1.00 25.24 C

ATOM 626 N SER A 83 -11.512 -7.537 4.010 1.00 24.46 N

ATOM 627 CA SER A 83 -10.603 -8.660 3.962 1.00 24.58 C

ATOM 628 C SER A 83 -10.449 -9.095 2.509 1.00 31.65 C

ATOM 629 O SER A 83 -11.449 -9.331 1.817 1.00 31.37 O

ATOM 630 CB SER A 83 -11.114 -9.804 4.813 1.00 26.84 C

ATOM 631 OG SER A 83 -10.217 -10.897 4.718 1.00 36.17 O

ATOM 632 N LEU A 84 -9.189 -9.177 2.034 1.00 29.86 N

ATOM 633 CA LEU A 84 -8.907 -9.615 0.666 1.00 30.10 C

ATOM 634 C LEU A 84 -9.328 -11.070 0.469 1.00 36.42 C

ATOM 635 O LEU A 84 -9.573 -11.486 -0.664 1.00 37.45 O

ATOM 636 CB LEU A 84 -7.438 -9.386 0.283 1.00 29.79 C

ATOM 637 CG LEU A 84 -7.077 -7.943 -0.121 1.00 33.47 C

ATOM 638 CD1 LEU A 84 -5.590 -7.662 0.075 1.00 33.64 C

ATOM 639 CD2 LEU A 84 -7.511 -7.630 -1.553 1.00 33.92 C

ATOM 640 N VAL A 85 -9.508 -11.808 1.585 1.00 32.51 N

ATOM 641 CA VAL A 85 -9.950 -13.196 1.577 1.00 31.87 C

ATOM 642 C VAL A 85 -11.442 -13.315 1.997 1.00 33.70 C

ATOM 643 O VAL A 85 -11.942 -14.417 2.243 1.00 32.39 O

ATOM 644 CB VAL A 85 -8.992 -14.120 2.368 1.00 36.39 C

ATOM 645 CG1 VAL A 85 -7.571 -13.995 1.835 1.00 36.65 C

ATOM 646 CG2 VAL A 85 -9.030 -13.856 3.871 1.00 36.25 C

ATOM 647 N SER A 86 -12.157 -12.169 2.025 1.00 29.46 N

ATOM 648 CA SER A 86 -13.588 -12.127 2.317 1.00 28.01 C

ATOM 649 C SER A 86 -14.386 -11.328 1.245 1.00 32.96 C

ATOM 650 O SER A 86 -14.446 -10.093 1.282 1.00 32.20 O

ATOM 651 CB SER A 86 -13.867 -11.650 3.737 1.00 27.96 C

ATOM 652 OG SER A 86 -15.233 -11.330 3.946 1.00 34.17 O

ATOM 653 N PRO A 87 -15.042 -12.032 0.293 1.00 30.65 N

ATOM 654 CA PRO A 87 -15.850 -11.320 -0.713 1.00 30.50 C

ATOM 655 C PRO A 87 -17.065 -10.637 -0.090 1.00 32.00 C

ATOM 656 O PRO A 87 -17.445 -9.563 -0.559 1.00 32.28 O

ATOM 657 CB PRO A 87 -16.219 -12.420 -1.725 1.00 32.50 C

ATOM 658 CG PRO A 87 -15.273 -13.572 -1.410 1.00 37.15 C

ATOM 659 CD PRO A 87 -15.091 -13.488 0.070 1.00 32.43 C

ATOM 660 N ALA A 88 -17.630 -11.221 0.999 1.00 26.33 N

ATOM 661 CA ALA A 88 -18.755 -10.635 1.750 1.00 25.16 C

ATOM 662 C ALA A 88 -18.387 -9.227 2.249 1.00 29.29 C

ATOM 663 O ALA A 88 -19.171 -8.297 2.051 1.00 28.52 O

ATOM 664 CB ALA A 88 -19.146 -11.527 2.920 1.00 25.24 C

ATOM 665 N SER A 89 -17.158 -9.056 2.808 1.00 25.96 N

ATOM 666 CA SER A 89 -16.670 -7.755 3.287 1.00 26.28 C

ATOM 667 C SER A 89 -16.376 -6.762 2.161 1.00 31.48 C

ATOM 668 O SER A 89 -16.497 -5.552 2.368 1.00 29.19 O

ATOM 669 CB SER A 89 -15.446 -7.921 4.178 1.00 28.47 C

ATOM 670 OG SER A 89 -14.346 -8.416 3.440 1.00 31.66 O

ATOM 671 N PHE A 90 -15.988 -7.291 0.973 1.00 30.70 N

ATOM 672 CA PHE A 90 -15.686 -6.517 -0.224 1.00 30.64 C

ATOM 673 C PHE A 90 -16.963 -5.937 -0.820 1.00 33.99 C

ATOM 674 O PHE A 90 -16.965 -4.767 -1.191 1.00 33.91 O

ATOM 675 CB PHE A 90 -14.896 -7.354 -1.258 1.00 32.94 C

ATOM 676 CG PHE A 90 -14.565 -6.607 -2.539 1.00 34.94 C

ATOM 677 CD1 PHE A 90 -13.645 -5.562 -2.538 1.00 38.36 C

ATOM 678 CD2 PHE A 90 -15.225 -6.900 -3.725 1.00 37.15 C

ATOM 679 CE1 PHE A 90 -13.367 -4.848 -3.708 1.00 39.35 C

ATOM 680 CE2 PHE A 90 -14.940 -6.193 -4.899 1.00 40.34 C

ATOM 681 CZ PHE A 90 -14.011 -5.173 -4.882 1.00 38.64 C

ATOM 682 N GLU A 91 -18.054 -6.730 -0.893 1.00 30.80 N

ATOM 683 CA GLU A 91 -19.359 -6.259 -1.409 1.00 30.88 C

ATOM 684 C GLU A 91 -19.945 -5.180 -0.497 1.00 32.45 C

ATOM 685 O GLU A 91 -20.619 -4.259 -0.972 1.00 31.67 O

ATOM 686 CB GLU A 91 -20.359 -7.416 -1.554 1.00 32.73 C

ATOM 687 CG GLU A 91 -20.103 -8.278 -2.780 1.00 47.37 C

ATOM 688 CD GLU A 91 -20.817 -9.611 -2.746 1.00 80.31 C

ATOM 689 OE1 GLU A 91 -20.211 -10.593 -2.257 1.00 75.76 O

ATOM 690 OE2 GLU A 91 -21.983 -9.675 -3.204 1.00 82.70 O

ATOM 691 N ASN A 92 -19.637 -5.284 0.814 1.00 27.64 N

ATOM 692 CA ASN A 92 -20.064 -4.353 1.840 1.00 26.82 C

ATOM 693 C ASN A 92 -19.442 -2.970 1.701 1.00 30.50 C

ATOM 694 O ASN A 92 -19.955 -2.016 2.284 1.00 30.25 O

ATOM 695 CB ASN A 92 -19.848 -4.938 3.212 1.00 25.63 C

ATOM 696 CG ASN A 92 -21.083 -5.619 3.727 1.00 35.81 C

ATOM 697 OD1 ASN A 92 -22.209 -5.157 3.529 1.00 23.63 O

ATOM 698 ND2 ASN A 92 -20.909 -6.730 4.401 1.00 32.21 N

ATOM 699 N VAL A 93 -18.403 -2.842 0.862 1.00 27.22 N

ATOM 700 CA VAL A 93 -17.770 -1.563 0.577 1.00 27.09 C

ATOM 701 C VAL A 93 -18.800 -0.676 -0.107 1.00 31.52 C

ATOM 702 O VAL A 93 -19.130 0.375 0.433 1.00 31.42 O

ATOM 703 CB VAL A 93 -16.436 -1.706 -0.209 1.00 30.64 C

ATOM 704 CG1 VAL A 93 -15.888 -0.345 -0.641 1.00 30.14 C

ATOM 705 CG2 VAL A 93 -15.398 -2.462 0.619 1.00 30.36 C

ATOM 706 N ARG A 94 -19.370 -1.139 -1.236 1.00 29.01 N

ATOM 707 CA ARG A 94 -20.398 -0.404 -1.969 1.00 28.93 C

ATOM 708 C ARG A 94 -21.761 -0.509 -1.303 1.00 34.41 C

ATOM 709 O ARG A 94 -22.532 0.447 -1.378 1.00 36.00 O

ATOM 710 CB ARG A 94 -20.473 -0.820 -3.457 1.00 29.71 C

ATOM 711 CG ARG A 94 -20.813 -2.292 -3.725 1.00 41.70 C

ATOM 712 CD ARG A 94 -20.833 -2.596 -5.210 1.00 52.16 C

ATOM 713 NE ARG A 94 -20.374 -3.960 -5.488 1.00 62.24 N

ATOM 714 CZ ARG A 94 -19.636 -4.305 -6.540 1.00 74.03 C

ATOM 715 NH1 ARG A 94 -19.250 -3.386 -7.419 1.00 56.27 N

ATOM 716 NH2 ARG A 94 -19.261 -5.567 -6.711 1.00 60.01 N

ATOM 717 N ALA A 95 -22.083 -1.663 -0.690 1.00 30.06 N

ATOM 718 CA ALA A 95 -23.387 -1.868 -0.053 1.00 30.15 C

ATOM 719 C ALA A 95 -23.601 -1.023 1.187 1.00 34.49 C

ATOM 720 O ALA A 95 -24.622 -0.346 1.292 1.00 34.38 O

ATOM 721 CB ALA A 95 -23.593 -3.336 0.286 1.00 30.83 C

ATOM 722 N LYS A 96 -22.632 -1.050 2.115 1.00 31.02 N

ATOM 723 CA LYS A 96 -22.740 -0.397 3.406 1.00 29.63 C

ATOM 724 C LYS A 96 -21.793 0.793 3.651 1.00 31.02 C

ATOM 725 O LYS A 96 -22.259 1.888 3.980 1.00 31.05 O

ATOM 726 CB LYS A 96 -22.548 -1.474 4.500 1.00 31.43 C

ATOM 727 CG LYS A 96 -22.728 -0.985 5.944 1.00 40.82 C

ATOM 728 CD LYS A 96 -22.527 -2.116 6.934 1.00 47.74 C

ATOM 729 CE LYS A 96 -22.873 -1.708 8.345 1.00 61.76 C

ATOM 730 NZ LYS A 96 -22.295 -2.647 9.348 1.00 71.02 N

ATOM 731 N TRP A 97 -20.484 0.557 3.561 1.00 25.25 N

ATOM 732 CA TRP A 97 -19.456 1.487 3.986 1.00 24.85 C

ATOM 733 C TRP A 97 -19.349 2.773 3.193 1.00 29.64 C

ATOM 734 O TRP A 97 -19.352 3.826 3.831 1.00 29.22 O

ATOM 735 CB TRP A 97 -18.116 0.774 4.103 1.00 23.60 C

ATOM 736 CG TRP A 97 -18.226 -0.366 5.073 1.00 24.44 C

ATOM 737 CD1 TRP A 97 -18.135 -1.695 4.784 1.00 27.34 C

ATOM 738 CD2 TRP A 97 -18.650 -0.279 6.442 1.00 24.21 C

ATOM 739 NE1 TRP A 97 -18.461 -2.444 5.892 1.00 27.07 N

ATOM 740 CE2 TRP A 97 -18.788 -1.600 6.923 1.00 28.38 C

ATOM 741 CE3 TRP A 97 -18.929 0.793 7.315 1.00 25.45 C

ATOM 742 CZ2 TRP A 97 -19.158 -1.882 8.247 1.00 27.57 C

ATOM 743 CZ3 TRP A 97 -19.343 0.511 8.612 1.00 26.79 C

ATOM 744 CH2 TRP A 97 -19.440 -0.810 9.070 1.00 27.39 C

ATOM 745 N TYR A 98 -19.280 2.729 1.853 1.00 26.62 N

ATOM 746 CA TYR A 98 -19.203 3.959 1.070 1.00 27.01 C

ATOM 747 C TYR A 98 -20.514 4.785 1.197 1.00 31.26 C

ATOM 748 O TYR A 98 -20.398 5.960 1.523 1.00 31.19 O

ATOM 749 CB TYR A 98 -18.771 3.685 -0.381 1.00 29.00 C

ATOM 750 CG TYR A 98 -19.069 4.809 -1.353 1.00 30.46 C

ATOM 751 CD1 TYR A 98 -18.313 5.982 -1.349 1.00 32.09 C

ATOM 752 CD2 TYR A 98 -20.094 4.693 -2.290 1.00 30.73 C

ATOM 753 CE1 TYR A 98 -18.558 7.003 -2.269 1.00 31.63 C

ATOM 754 CE2 TYR A 98 -20.363 5.718 -3.197 1.00 31.70 C

ATOM 755 CZ TYR A 98 -19.594 6.872 -3.179 1.00 39.11 C

ATOM 756 OH TYR A 98 -19.852 7.888 -4.060 1.00 42.18 O

ATOM 757 N PRO A 99 -21.747 4.225 1.068 1.00 29.04 N

ATOM 758 CA PRO A 99 -22.944 5.065 1.299 1.00 29.25 C

ATOM 759 C PRO A 99 -22.985 5.724 2.684 1.00 34.85 C

ATOM 760 O PRO A 99 -23.262 6.926 2.753 1.00 34.01 O

ATOM 761 CB PRO A 99 -24.127 4.111 1.080 1.00 30.87 C

ATOM 762 CG PRO A 99 -23.558 2.742 1.072 1.00 35.50 C

ATOM 763 CD PRO A 99 -22.124 2.847 0.683 1.00 30.98 C

ATOM 764 N GLU A 100 -22.652 4.970 3.773 1.00 33.19 N

ATOM 765 CA GLU A 100 -22.653 5.503 5.147 1.00 33.74 C

ATOM 766 C GLU A 100 -21.627 6.610 5.382 1.00 39.76 C

ATOM 767 O GLU A 100 -21.938 7.585 6.071 1.00 39.92 O

ATOM 768 CB GLU A 100 -22.497 4.401 6.201 1.00 35.22 C

ATOM 769 CG GLU A 100 -22.906 4.877 7.589 1.00 45.37 C

ATOM 770 CD GLU A 100 -22.850 3.863 8.712 1.00 56.14 C

ATOM 771 OE1 GLU A 100 -23.878 3.682 9.408 1.00 50.83 O

ATOM 772 OE2 GLU A 100 -21.753 3.313 8.949 1.00 40.06 O

ATOM 773 N VAL A 101 -20.411 6.461 4.819 1.00 36.69 N

ATOM 774 CA VAL A 101 -19.348 7.459 4.953 1.00 36.29 C

ATOM 775 C VAL A 101 -19.784 8.744 4.239 1.00 42.67 C

ATOM 776 O VAL A 101 -19.733 9.819 4.844 1.00 42.96 O

ATOM 777 CB VAL A 101 -17.947 6.926 4.510 1.00 38.90 C

ATOM 778 CG1 VAL A 101 -16.925 8.048 4.396 1.00 38.44 C

ATOM 779 CG2 VAL A 101 -17.431 5.858 5.470 1.00 38.38 C

ATOM 780 N ARG A 102 -20.284 8.620 2.993 1.00 40.12 N

ATOM 781 CA ARG A 102 -20.749 9.760 2.197 1.00 40.19 C

ATOM 782 C ARG A 102 -21.982 10.456 2.779 1.00 44.86 C

ATOM 783 O ARG A 102 -22.105 11.679 2.658 1.00 43.94 O

ATOM 784 CB ARG A 102 -20.964 9.365 0.730 1.00 39.83 C

ATOM 785 CG ARG A 102 -19.649 9.203 -0.015 1.00 46.84 C

ATOM 786 CD ARG A 102 -18.939 10.520 -0.282 1.00 54.27 C

ATOM 787 NE ARG A 102 -17.536 10.312 -0.641 1.00 65.87 N

ATOM 788 CZ ARG A 102 -16.504 10.605 0.145 1.00 79.84 C

ATOM 789 NH1 ARG A 102 -16.703 11.137 1.346 1.00 58.41 N

ATOM 790 NH2 ARG A 102 -15.263 10.381 -0.269 1.00 71.53 N

ATOM 791 N HIS A 103 -22.862 9.687 3.445 1.00 42.72 N

ATOM 792 CA HIS A 103 -24.068 10.198 4.101 1.00 42.83 C

ATOM 793 C HIS A 103 -23.721 11.299 5.103 1.00 42.83 C

ATOM 794 O HIS A 103 -24.233 12.412 5.007 1.00 40.78 O

ATOM 795 CB HIS A 103 -24.822 9.050 4.801 1.00 44.61 C

ATOM 796 CG HIS A 103 -26.193 9.424 5.259 1.00 49.09 C

ATOM 797 ND1 HIS A 103 -26.412 9.976 6.513 1.00 51.47 N

ATOM 798 CD2 HIS A 103 -27.378 9.324 4.612 1.00 51.70 C

ATOM 799 CE1 HIS A 103 -27.718 10.190 6.588 1.00 51.21 C

ATOM 800 NE2 HIS A 103 -28.342 9.813 5.468 1.00 51.56 N

ATOM 801 N HIS A 104 -22.814 10.990 6.027 1.00 39.32 N

ATOM 802 CA HIS A 104 -22.389 11.881 7.103 1.00 39.13 C

ATOM 803 C HIS A 104 -21.257 12.839 6.699 1.00 44.92 C

ATOM 804 O HIS A 104 -21.220 13.974 7.182 1.00 43.96 O

ATOM 805 CB HIS A 104 -22.030 11.048 8.343 1.00 39.27 C

ATOM 806 CG HIS A 104 -23.149 10.149 8.777 1.00 42.42 C

ATOM 807 ND1 HIS A 104 -23.243 8.843 8.325 1.00 44.26 N

ATOM 808 CD2 HIS A 104 -24.222 10.416 9.559 1.00 43.83 C

ATOM 809 CE1 HIS A 104 -24.352 8.353 8.861 1.00 43.39 C

ATOM 810 NE2 HIS A 104 -24.967 9.257 9.622 1.00 43.62 N

ATOM 811 N CYS A 105 -20.351 12.391 5.811 1.00 43.56 N

ATOM 812 CA CYS A 105 -19.208 13.177 5.335 1.00 45.02 C

ATOM 813 C CYS A 105 -19.128 13.034 3.800 1.00 50.42 C

ATOM 814 O CYS A 105 -18.549 12.059 3.304 1.00 51.08 O

ATOM 815 CB CYS A 105 -17.912 12.725 6.013 1.00 45.68 C

ATOM 816 SG CYS A 105 -17.926 12.835 7.822 1.00 49.67 S

ATOM 817 N PRO A 106 -19.718 13.963 3.019 1.00 46.46 N

ATOM 818 CA PRO A 106 -19.724 13.784 1.556 1.00 45.93 C

ATOM 819 C PRO A 106 -18.472 14.257 0.818 1.00 49.02 C

ATOM 820 O PRO A 106 -18.293 13.892 -0.350 1.00 49.12 O

ATOM 821 CB PRO A 106 -20.977 14.556 1.108 1.00 47.52 C

ATOM 822 CG PRO A 106 -21.577 15.171 2.379 1.00 51.85 C

ATOM 823 CD PRO A 106 -20.497 15.156 3.404 1.00 47.71 C

ATOM 824 N ASN A 107 -17.617 15.061 1.477 1.00 43.73 N

ATOM 825 CA ASN A 107 -16.427 15.613 0.837 1.00 43.13 C

ATOM 826 C ASN A 107 -15.093 15.063 1.376 1.00 44.90 C

ATOM 827 O ASN A 107 -14.046 15.293 0.755 1.00 45.00 O

ATOM 828 CB ASN A 107 -16.459 17.149 0.903 1.00 46.88 C

ATOM 829 CG ASN A 107 -17.725 17.750 0.336 1.00 73.05 C

ATOM 830 OD1 ASN A 107 -18.613 18.198 1.074 1.00 62.76 O

ATOM 831 ND2 ASN A 107 -17.857 17.733 -0.988 1.00 67.57 N

ATOM 832 N THR A 108 -15.136 14.341 2.515 1.00 38.97 N

ATOM 833 CA THR A 108 -13.978 13.750 3.193 1.00 37.32 C

ATOM 834 C THR A 108 -13.304 12.666 2.319 1.00 37.39 C

ATOM 835 O THR A 108 -14.008 11.811 1.783 1.00 36.58 O

ATOM 836 CB THR A 108 -14.388 13.267 4.599 1.00 45.02 C

ATOM 837 OG1 THR A 108 -14.921 14.378 5.333 1.00 44.24 O

ATOM 838 CG2 THR A 108 -13.232 12.634 5.381 1.00 41.53 C

ATOM 839 N PRO A 109 -11.956 12.698 2.150 1.00 31.51 N

ATOM 840 CA PRO A 109 -11.297 11.691 1.289 1.00 31.17 C

ATOM 841 C PRO A 109 -11.317 10.258 1.828 1.00 32.91 C

ATOM 842 O PRO A 109 -11.257 10.049 3.031 1.00 30.86 O

ATOM 843 CB PRO A 109 -9.863 12.218 1.159 1.00 32.98 C

ATOM 844 CG PRO A 109 -9.650 13.041 2.393 1.00 37.11 C

ATOM 845 CD PRO A 109 -10.980 13.673 2.684 1.00 32.19 C

ATOM 846 N ILE A 110 -11.391 9.278 0.923 1.00 30.16 N

ATOM 847 CA ILE A 110 -11.395 7.859 1.268 1.00 30.44 C

ATOM 848 C ILE A 110 -10.193 7.143 0.655 1.00 34.60 C

ATOM 849 O ILE A 110 -9.869 7.368 -0.512 1.00 35.28 O

ATOM 850 CB ILE A 110 -12.718 7.170 0.827 1.00 33.70 C

ATOM 851 CG1 ILE A 110 -13.929 7.701 1.616 1.00 35.11 C

ATOM 852 CG2 ILE A 110 -12.619 5.630 0.907 1.00 33.05 C

ATOM 853 CD1 ILE A 110 -15.334 7.246 1.034 1.00 44.79 C

ATOM 854 N ILE A 111 -9.560 6.257 1.439 1.00 29.75 N

ATOM 855 CA ILE A 111 -8.501 5.355 0.992 1.00 28.72 C

ATOM 856 C ILE A 111 -9.118 3.960 1.181 1.00 32.34 C

ATOM 857 O ILE A 111 -9.548 3.626 2.293 1.00 31.88 O

ATOM 858 CB ILE A 111 -7.194 5.501 1.812 1.00 31.68 C

ATOM 859 CG1 ILE A 111 -6.675 6.973 1.876 1.00 32.02 C

ATOM 860 CG2 ILE A 111 -6.113 4.522 1.331 1.00 32.93 C

ATOM 861 CD1 ILE A 111 -6.065 7.607 0.617 1.00 41.84 C

ATOM 862 N LEU A 112 -9.250 3.186 0.090 1.00 27.76 N

ATOM 863 CA LEU A 112 -9.788 1.832 0.173 1.00 25.91 C

ATOM 864 C LEU A 112 -8.635 0.892 0.553 1.00 28.81 C

ATOM 865 O LEU A 112 -7.574 0.921 -0.085 1.00 29.97 O

ATOM 866 CB LEU A 112 -10.471 1.417 -1.131 1.00 25.56 C

ATOM 867 CG LEU A 112 -11.110 0.006 -1.176 1.00 29.71 C

ATOM 868 CD1 LEU A 112 -12.143 -0.198 -0.051 1.00 29.07 C

ATOM 869 CD2 LEU A 112 -11.751 -0.251 -2.533 1.00 29.92 C

ATOM 870 N VAL A 113 -8.816 0.132 1.643 1.00 21.96 N

ATOM 871 CA VAL A 113 -7.791 -0.749 2.177 1.00 20.84 C

ATOM 872 C VAL A 113 -8.208 -2.226 2.198 1.00 25.30 C

ATOM 873 O VAL A 113 -9.131 -2.604 2.914 1.00 25.86 O

ATOM 874 CB VAL A 113 -7.268 -0.288 3.582 1.00 23.73 C

ATOM 875 CG1 VAL A 113 -6.072 -1.133 4.028 1.00 23.29 C

ATOM 876 CG2 VAL A 113 -6.894 1.196 3.602 1.00 22.98 C

ATOM 877 N GLY A 114 -7.461 -3.043 1.468 1.00 22.14 N

ATOM 878 CA GLY A 114 -7.570 -4.496 1.484 1.00 22.48 C

ATOM 879 C GLY A 114 -6.571 -5.074 2.488 1.00 28.46 C

ATOM 880 O GLY A 114 -5.366 -4.823 2.410 1.00 27.33 O

ATOM 881 N THR A 115 -7.075 -5.812 3.470 1.00 27.15 N

ATOM 882 CA THR A 115 -6.301 -6.429 4.552 1.00 27.41 C

ATOM 883 C THR A 115 -6.133 -7.947 4.326 1.00 31.37 C

ATOM 884 O THR A 115 -6.773 -8.510 3.436 1.00 30.43 O

ATOM 885 CB THR A 115 -7.039 -6.193 5.881 1.00 39.67 C

ATOM 886 OG1 THR A 115 -8.368 -6.705 5.743 1.00 37.91 O

ATOM 887 CG2 THR A 115 -7.079 -4.708 6.288 1.00 41.08 C

ATOM 888 N LYS A 116 -5.308 -8.603 5.174 1.00 28.45 N

ATOM 889 CA LYS A 116 -5.015 -10.041 5.147 1.00 28.70 C

ATOM 890 C LYS A 116 -4.424 -10.471 3.795 1.00 34.12 C

ATOM 891 O LYS A 116 -4.742 -11.539 3.272 1.00 33.75 O

ATOM 892 CB LYS A 116 -6.233 -10.902 5.575 1.00 31.10 C

ATOM 893 CG LYS A 116 -6.968 -10.402 6.825 1.00 37.76 C

ATOM 894 CD LYS A 116 -7.890 -11.468 7.387 1.00 40.79 C

ATOM 895 CE LYS A 116 -8.848 -10.904 8.398 1.00 39.17 C

ATOM 896 NZ LYS A 116 -9.552 -11.984 9.148 1.00 40.14 N

ATOM 897 N LEU A 117 -3.532 -9.620 3.256 1.00 32.29 N

ATOM 898 CA LEU A 117 -2.799 -9.800 2.002 1.00 32.37 C

ATOM 899 C LEU A 117 -1.977 -11.080 2.044 1.00 36.19 C

ATOM 900 O LEU A 117 -1.832 -11.734 1.012 1.00 37.38 O

ATOM 901 CB LEU A 117 -1.900 -8.575 1.770 1.00 32.52 C

ATOM 902 CG LEU A 117 -0.919 -8.549 0.597 1.00 37.60 C

ATOM 903 CD1 LEU A 117 -1.642 -8.435 -0.738 1.00 38.04 C

ATOM 904 CD2 LEU A 117 0.057 -7.391 0.748 1.00 39.27 C

ATOM 905 N ASP A 118 -1.485 -11.459 3.239 1.00 31.63 N

ATOM 906 CA ASP A 118 -0.707 -12.685 3.478 1.00 31.51 C

ATOM 907 C ASP A 118 -1.535 -13.971 3.248 1.00 36.90 C

ATOM 908 O ASP A 118 -0.967 -15.027 2.957 1.00 36.89 O

ATOM 909 CB ASP A 118 -0.135 -12.681 4.906 1.00 32.55 C

ATOM 910 CG ASP A 118 -1.199 -12.706 5.980 1.00 36.75 C

ATOM 911 OD1 ASP A 118 -1.963 -11.720 6.085 1.00 36.35 O

ATOM 912 OD2 ASP A 118 -1.302 -13.729 6.690 1.00 41.21 O

ATOM 913 N LEU A 119 -2.860 -13.883 3.394 1.00 33.66 N

ATOM 914 CA LEU A 119 -3.722 -15.037 3.207 1.00 34.03 C

ATOM 915 C LEU A 119 -4.130 -15.269 1.755 1.00 40.48 C

ATOM 916 O LEU A 119 -4.625 -16.356 1.464 1.00 40.64 O

ATOM 917 CB LEU A 119 -4.969 -14.958 4.107 1.00 33.59 C

ATOM 918 CG LEU A 119 -4.760 -15.137 5.596 1.00 37.79 C

ATOM 919 CD1 LEU A 119 -5.987 -14.712 6.355 1.00 37.74 C

ATOM 920 CD2 LEU A 119 -4.398 -16.575 5.947 1.00 40.91 C

ATOM 921 N ARG A 120 -3.922 -14.284 0.844 1.00 38.02 N

ATOM 922 CA ARG A 120 -4.326 -14.423 -0.566 1.00 39.34 C

ATOM 923 C ARG A 120 -3.635 -15.579 -1.316 1.00 47.47 C

ATOM 924 O ARG A 120 -4.229 -16.137 -2.235 1.00 47.00 O

ATOM 925 CB ARG A 120 -4.184 -13.109 -1.344 1.00 37.77 C

ATOM 926 CG ARG A 120 -5.107 -13.028 -2.567 1.00 39.26 C

ATOM 927 CD ARG A 120 -5.313 -11.614 -3.072 1.00 40.16 C

ATOM 928 NE ARG A 120 -4.046 -10.939 -3.366 1.00 39.14 N

ATOM 929 CZ ARG A 120 -3.938 -9.666 -3.727 1.00 44.01 C

ATOM 930 NH1 ARG A 120 -5.020 -8.920 -3.885 1.00 19.21 N

ATOM 931 NH2 ARG A 120 -2.745 -9.136 -3.957 1.00 38.30 N

ATOM 932 N ASP A 121 -2.414 -15.948 -0.909 1.00 48.01 N

ATOM 933 CA ASP A 121 -1.658 -17.053 -1.509 1.00 49.42 C

ATOM 934 C ASP A 121 -1.713 -18.330 -0.639 1.00 54.80 C

ATOM 935 O ASP A 121 -1.088 -19.336 -0.998 1.00 54.59 O

ATOM 936 CB ASP A 121 -0.198 -16.633 -1.796 1.00 51.90 C

ATOM 937 CG ASP A 121 0.011 -15.815 -3.068 1.00 67.47 C

ATOM 938 OD1 ASP A 121 -0.676 -16.102 -4.088 1.00 69.25 O

ATOM 939 OD2 ASP A 121 0.911 -14.936 -3.070 1.00 72.05 O

ATOM 940 N ASP A 122 -2.480 -18.295 0.481 1.00 51.56 N

ATOM 941 CA ASP A 122 -2.636 -19.418 1.421 1.00 51.62 C

ATOM 942 C ASP A 122 -3.574 -20.506 0.867 1.00 54.63 C

ATOM 943 O ASP A 122 -4.778 -20.273 0.744 1.00 54.16 O

ATOM 944 CB ASP A 122 -3.106 -18.909 2.806 1.00 53.66 C

ATOM 945 CG ASP A 122 -3.106 -19.930 3.932 1.00 66.79 C

ATOM 946 OD1 ASP A 122 -2.228 -20.825 3.928 1.00 68.10 O

ATOM 947 OD2 ASP A 122 -3.944 -19.797 4.853 1.00 73.29 O

ATOM 948 N LYS A 123 -3.004 -21.687 0.529 1.00 50.77 N

ATOM 949 CA LYS A 123 -3.693 -22.859 -0.036 1.00 50.80 C

ATOM 950 C LYS A 123 -4.999 -23.231 0.718 1.00 55.93 C

ATOM 951 O LYS A 123 -6.031 -23.437 0.068 1.00 56.08 O

ATOM 952 CB LYS A 123 -2.723 -24.058 -0.126 1.00 52.85 C

ATOM 953 CG LYS A 123 -3.313 -25.331 -0.749 1.00 61.82 C

ATOM 954 CD LYS A 123 -2.719 -26.619 -0.161 1.00 66.26 C

ATOM 955 CE LYS A 123 -3.224 -26.967 1.227 1.00 77.07 C

ATOM 956 NZ LYS A 123 -4.655 -27.384 1.231 1.00 87.22 N

ATOM 957 N ASP A 124 -4.952 -23.281 2.072 1.00 51.83 N

ATOM 958 CA ASP A 124 -6.105 -23.591 2.928 1.00 51.82 C

ATOM 959 C ASP A 124 -7.232 -22.560 2.776 1.00 54.00 C

ATOM 960 O ASP A 124 -8.402 -22.938 2.668 1.00 53.00 O

ATOM 961 CB ASP A 124 -5.681 -23.716 4.405 1.00 54.37 C

ATOM 962 CG ASP A 124 -4.619 -24.774 4.654 1.00 70.73 C

ATOM 963 OD1 ASP A 124 -3.421 -24.468 4.464 1.00 72.85 O

ATOM 964 OD2 ASP A 124 -4.988 -25.911 5.040 1.00 76.55 O

ATOM 965 N THR A 125 -6.868 -21.264 2.744 1.00 49.91 N

ATOM 966 CA THR A 125 -7.791 -20.135 2.596 1.00 49.41 C

ATOM 967 C THR A 125 -8.437 -20.130 1.205 1.00 52.97 C

ATOM 968 O THR A 125 -9.620 -19.806 1.081 1.00 51.58 O

ATOM 969 CB THR A 125 -7.079 -18.815 2.951 1.00 56.27 C

ATOM 970 OG1 THR A 125 -6.457 -18.940 4.235 1.00 55.06 O

ATOM 971 CG2 THR A 125 -8.023 -17.620 2.960 1.00 52.99 C

ATOM 972 N ILE A 126 -7.659 -20.490 0.165 1.00 50.81 N

ATOM 973 CA ILE A 126 -8.138 -20.561 -1.222 1.00 50.92 C

ATOM 974 C ILE A 126 -9.129 -21.731 -1.364 1.00 54.16 C

ATOM 975 O ILE A 126 -10.181 -21.560 -1.988 1.00 53.24 O

ATOM 976 CB ILE A 126 -6.965 -20.605 -2.251 1.00 54.18 C

ATOM 977 CG1 ILE A 126 -6.057 -19.357 -2.118 1.00 54.52 C

ATOM 978 CG2 ILE A 126 -7.487 -20.736 -3.693 1.00 55.06 C

ATOM 979 CD1 ILE A 126 -4.586 -19.569 -2.526 1.00 61.39 C

ATOM 980 N GLU A 127 -8.808 -22.892 -0.740 1.00 50.62 N

ATOM 981 CA GLU A 127 -9.647 -24.094 -0.741 1.00 50.29 C

ATOM 982 C GLU A 127 -10.990 -23.830 -0.074 1.00 52.16 C

ATOM 983 O GLU A 127 -12.018 -24.163 -0.667 1.00 53.16 O

ATOM 984 CB GLU A 127 -8.930 -25.289 -0.084 1.00 52.12 C

ATOM 985 CG GLU A 127 -8.278 -26.247 -1.077 1.00 66.20 C

ATOM 986 CD GLU A 127 -9.185 -27.298 -1.697 1.00 91.39 C

ATOM 987 OE1 GLU A 127 -9.649 -28.199 -0.959 1.00 84.72 O

ATOM 988 OE2 GLU A 127 -9.384 -27.256 -2.933 1.00 83.42 O

ATOM 989 N LYS A 128 -10.991 -23.193 1.126 1.00 45.24 N

ATOM 990 CA LYS A 128 -12.215 -22.850 1.871 1.00 43.67 C

ATOM 991 C LYS A 128 -13.144 -21.945 1.050 1.00 46.05 C

ATOM 992 O LYS A 128 -14.360 -22.173 1.019 1.00 45.87 O

ATOM 993 CB LYS A 128 -11.889 -22.208 3.232 1.00 45.58 C

ATOM 994 CG LYS A 128 -11.348 -23.193 4.271 1.00 62.31 C

ATOM 995 CD LYS A 128 -11.076 -22.549 5.642 1.00 73.77 C

ATOM 996 CE LYS A 128 -9.718 -21.887 5.772 1.00 82.13 C

ATOM 997 NZ LYS A 128 -9.804 -20.404 5.657 1.00 87.15 N

ATOM 998 N LEU A 129 -12.561 -20.939 0.370 1.00 40.98 N

ATOM 999 CA LEU A 129 -13.275 -19.996 -0.488 1.00 40.25 C

ATOM 1000 C LEU A 129 -13.886 -20.690 -1.714 1.00 44.53 C

ATOM 1001 O LEU A 129 -15.018 -20.369 -2.089 1.00 43.46 O

ATOM 1002 CB LEU A 129 -12.336 -18.874 -0.962 1.00 39.66 C

ATOM 1003 CG LEU A 129 -12.182 -17.663 -0.068 1.00 43.33 C

ATOM 1004 CD1 LEU A 129 -10.982 -16.840 -0.496 1.00 43.22 C

ATOM 1005 CD2 LEU A 129 -13.420 -16.801 -0.097 1.00 44.33 C

ATOM 1006 N LYS A 130 -13.117 -21.614 -2.345 1.00 41.24 N

ATOM 1007 CA LYS A 130 -13.516 -22.364 -3.533 1.00 41.73 C

ATOM 1008 C LYS A 130 -14.728 -23.251 -3.261 1.00 46.77 C

ATOM 1009 O LYS A 130 -15.591 -23.364 -4.134 1.00 47.25 O

ATOM 1010 CB LYS A 130 -12.337 -23.173 -4.102 1.00 44.84 C

ATOM 1011 CG LYS A 130 -12.536 -23.630 -5.554 1.00 61.27 C

ATOM 1012 CD LYS A 130 -11.580 -24.768 -5.980 1.00 75.02 C

ATOM 1013 CE LYS A 130 -11.624 -26.034 -5.132 1.00 88.52 C

ATOM 1014 NZ LYS A 130 -12.992 -26.619 -5.021 1.00 99.39 N

ATOM 1015 N GLU A 131 -14.816 -23.838 -2.042 1.00 43.09 N

ATOM 1016 CA GLU A 131 -15.953 -24.665 -1.610 1.00 42.84 C

ATOM 1017 C GLU A 131 -17.252 -23.834 -1.594 1.00 47.66 C

ATOM 1018 O GLU A 131 -18.312 -24.337 -1.973 1.00 48.78 O

ATOM 1019 CB GLU A 131 -15.699 -25.311 -0.231 1.00 44.13 C

ATOM 1020 CG GLU A 131 -14.509 -26.265 -0.150 1.00 56.89 C

ATOM 1021 CD GLU A 131 -14.435 -27.449 -1.104 1.00 81.36 C

ATOM 1022 OE1 GLU A 131 -15.489 -28.054 -1.408 1.00 79.37 O

ATOM 1023 OE2 GLU A 131 -13.303 -27.800 -1.511 1.00 74.52 O

ATOM 1024 N LYS A 132 -17.149 -22.552 -1.213 1.00 43.59 N

ATOM 1025 CA LYS A 132 -18.264 -21.605 -1.181 1.00 43.34 C

ATOM 1026 C LYS A 132 -18.399 -20.858 -2.539 1.00 49.20 C

ATOM 1027 O LYS A 132 -19.099 -19.837 -2.620 1.00 49.13 O

ATOM 1028 CB LYS A 132 -18.091 -20.636 0.001 1.00 45.18 C

ATOM 1029 CG LYS A 132 -18.121 -21.332 1.360 1.00 53.57 C

ATOM 1030 CD LYS A 132 -17.492 -20.513 2.469 1.00 59.91 C

ATOM 1031 CE LYS A 132 -17.308 -21.368 3.703 1.00 72.48 C

ATOM 1032 NZ LYS A 132 -16.763 -20.598 4.854 1.00 80.41 N

ATOM 1033 N LYS A 133 -17.735 -21.395 -3.607 1.00 46.45 N

ATOM 1034 CA LYS A 133 -17.704 -20.888 -4.998 1.00 46.72 C

ATOM 1035 C LYS A 133 -17.172 -19.440 -5.115 1.00 50.87 C

ATOM 1036 O LYS A 133 -17.499 -18.725 -6.069 1.00 50.54 O

ATOM 1037 CB LYS A 133 -19.076 -21.052 -5.693 1.00 49.37 C

ATOM 1038 CG LYS A 133 -19.331 -22.464 -6.208 1.00 62.71 C

ATOM 1039 CD LYS A 133 -20.822 -22.792 -6.245 1.00 69.61 C

ATOM 1040 CE LYS A 133 -21.106 -24.145 -6.857 1.00 73.48 C

ATOM 1041 NZ LYS A 133 -20.756 -25.262 -5.945 1.00 77.62 N

ATOM 1042 N LEU A 134 -16.317 -19.036 -4.160 1.00 47.50 N

ATOM 1043 CA LEU A 134 -15.730 -17.696 -4.103 1.00 47.07 C

ATOM 1044 C LEU A 134 -14.220 -17.695 -4.306 1.00 49.74 C

ATOM 1045 O LEU A 134 -13.551 -18.690 -4.042 1.00 49.83 O

ATOM 1046 CB LEU A 134 -16.111 -16.970 -2.794 1.00 46.88 C

ATOM 1047 CG LEU A 134 -17.574 -16.538 -2.654 1.00 51.43 C

ATOM 1048 CD1 LEU A 134 -17.931 -16.281 -1.202 1.00 51.81 C

ATOM 1049 CD2 LEU A 134 -17.892 -15.320 -3.514 1.00 53.41 C

ATOM 1050 N THR A 135 -13.700 -16.572 -4.800 1.00 44.34 N

ATOM 1051 CA THR A 135 -12.284 -16.348 -5.065 1.00 43.31 C

ATOM 1052 C THR A 135 -11.778 -15.143 -4.215 1.00 44.19 C

ATOM 1053 O THR A 135 -12.573 -14.225 -3.954 1.00 43.94 O

ATOM 1054 CB THR A 135 -12.076 -16.167 -6.596 1.00 52.05 C

ATOM 1055 OG1 THR A 135 -10.684 -16.191 -6.905 1.00 52.60 O

ATOM 1056 CG2 THR A 135 -12.708 -14.880 -7.151 1.00 50.31 C

ATOM 1057 N PRO A 136 -10.486 -15.090 -3.782 1.00 37.04 N

ATOM 1058 CA PRO A 136 -10.022 -13.892 -3.059 1.00 35.27 C

ATOM 1059 C PRO A 136 -10.080 -12.662 -3.971 1.00 36.31 C

ATOM 1060 O PRO A 136 -10.127 -12.804 -5.202 1.00 36.88 O

ATOM 1061 CB PRO A 136 -8.575 -14.228 -2.685 1.00 37.05 C

ATOM 1062 CG PRO A 136 -8.410 -15.679 -2.928 1.00 41.96 C

ATOM 1063 CD PRO A 136 -9.383 -16.050 -3.987 1.00 37.96 C

ATOM 1064 N ILE A 137 -10.117 -11.463 -3.380 1.00 29.22 N

ATOM 1065 CA ILE A 137 -10.169 -10.227 -4.160 1.00 27.75 C

ATOM 1066 C ILE A 137 -8.790 -9.925 -4.746 1.00 31.88 C

ATOM 1067 O ILE A 137 -7.793 -9.915 -4.028 1.00 31.40 O

ATOM 1068 CB ILE A 137 -10.772 -9.056 -3.338 1.00 30.32 C

ATOM 1069 CG1 ILE A 137 -12.229 -9.362 -2.855 1.00 30.96 C

ATOM 1070 CG2 ILE A 137 -10.676 -7.702 -4.062 1.00 30.47 C

ATOM 1071 CD1 ILE A 137 -13.304 -9.758 -3.931 1.00 39.71 C

ATOM 1072 N THR A 138 -8.738 -9.724 -6.052 1.00 29.44 N

ATOM 1073 CA THR A 138 -7.495 -9.429 -6.762 1.00 30.77 C

ATOM 1074 C THR A 138 -7.254 -7.906 -6.763 1.00 37.41 C

ATOM 1075 O THR A 138 -8.208 -7.140 -6.581 1.00 37.82 O

ATOM 1076 CB THR A 138 -7.569 -10.010 -8.188 1.00 40.04 C

ATOM 1077 OG1 THR A 138 -8.574 -9.313 -8.937 1.00 41.07 O

ATOM 1078 CG2 THR A 138 -7.861 -11.508 -8.198 1.00 36.96 C

ATOM 1079 N TYR A 139 -5.994 -7.467 -6.973 1.00 34.85 N

ATOM 1080 CA TYR A 139 -5.660 -6.038 -7.049 1.00 34.95 C

ATOM 1081 C TYR A 139 -6.551 -5.311 -8.100 1.00 39.83 C

ATOM 1082 O TYR A 139 -7.140 -4.278 -7.740 1.00 38.23 O

ATOM 1083 CB TYR A 139 -4.152 -5.801 -7.290 1.00 35.93 C

ATOM 1084 CG TYR A 139 -3.735 -4.351 -7.138 1.00 38.39 C

ATOM 1085 CD1 TYR A 139 -3.801 -3.468 -8.214 1.00 40.54 C

ATOM 1086 CD2 TYR A 139 -3.249 -3.868 -5.927 1.00 39.31 C

ATOM 1087 CE1 TYR A 139 -3.438 -2.127 -8.074 1.00 41.50 C

ATOM 1088 CE2 TYR A 139 -2.878 -2.532 -5.776 1.00 40.58 C

ATOM 1089 CZ TYR A 139 -2.969 -1.666 -6.855 1.00 48.72 C

ATOM 1090 OH TYR A 139 -2.599 -0.350 -6.711 1.00 49.41 O

ATOM 1091 N PRO A 140 -6.752 -5.856 -9.345 1.00 38.09 N

ATOM 1092 CA PRO A 140 -7.637 -5.169 -10.308 1.00 38.37 C

ATOM 1093 C PRO A 140 -9.079 -4.993 -9.815 1.00 41.53 C

ATOM 1094 O PRO A 140 -9.658 -3.928 -10.059 1.00 39.75 O

ATOM 1095 CB PRO A 140 -7.545 -6.048 -11.564 1.00 39.96 C

ATOM 1096 CG PRO A 140 -6.277 -6.783 -11.422 1.00 43.76 C

ATOM 1097 CD PRO A 140 -6.160 -7.060 -9.968 1.00 39.41 C

ATOM 1098 N GLN A 141 -9.637 -6.015 -9.090 1.00 38.78 N

ATOM 1099 CA GLN A 141 -10.987 -5.968 -8.483 1.00 38.69 C

ATOM 1100 C GLN A 141 -11.044 -4.813 -7.470 1.00 41.69 C

ATOM 1101 O GLN A 141 -11.902 -3.936 -7.589 1.00 41.14 O

ATOM 1102 CB GLN A 141 -11.336 -7.281 -7.755 1.00 39.93 C

ATOM 1103 CG GLN A 141 -11.774 -8.429 -8.642 1.00 52.11 C

ATOM 1104 CD GLN A 141 -12.214 -9.609 -7.809 1.00 69.41 C

ATOM 1105 OE1 GLN A 141 -11.417 -10.480 -7.431 1.00 57.80 O

ATOM 1106 NE2 GLN A 141 -13.499 -9.647 -7.479 1.00 69.65 N

ATOM 1107 N GLY A 142 -10.104 -4.812 -6.522 1.00 37.64 N

ATOM 1108 CA GLY A 142 -9.977 -3.782 -5.497 1.00 37.33 C

ATOM 1109 C GLY A 142 -9.823 -2.365 -6.028 1.00 40.29 C

ATOM 1110 O GLY A 142 -10.465 -1.445 -5.514 1.00 38.82 O

ATOM 1111 N LEU A 143 -8.982 -2.179 -7.072 1.00 37.53 N

ATOM 1112 CA LEU A 143 -8.752 -0.870 -7.707 1.00 37.37 C

ATOM 1113 C LEU A 143 -10.007 -0.350 -8.436 1.00 40.12 C

ATOM 1114 O LEU A 143 -10.245 0.859 -8.459 1.00 40.15 O

ATOM 1115 CB LEU A 143 -7.547 -0.923 -8.660 1.00 37.59 C

ATOM 1116 CG LEU A 143 -7.131 0.406 -9.276 1.00 42.60 C

ATOM 1117 CD1 LEU A 143 -6.005 1.049 -8.492 1.00 43.32 C

ATOM 1118 CD2 LEU A 143 -6.760 0.231 -10.714 1.00 45.45 C

ATOM 1119 N ALA A 144 -10.797 -1.260 -9.026 1.00 35.63 N

ATOM 1120 CA ALA A 144 -12.039 -0.917 -9.717 1.00 35.18 C

ATOM 1121 C ALA A 144 -13.078 -0.367 -8.708 1.00 39.58 C

ATOM 1122 O ALA A 144 -13.650 0.706 -8.941 1.00 39.03 O

ATOM 1123 CB ALA A 144 -12.582 -2.135 -10.441 1.00 35.61 C

ATOM 1124 N MET A 145 -13.256 -1.072 -7.562 1.00 36.36 N

ATOM 1125 CA MET A 145 -14.157 -0.690 -6.468 1.00 36.16 C

ATOM 1126 C MET A 145 -13.757 0.668 -5.917 1.00 39.77 C

ATOM 1127 O MET A 145 -14.628 1.498 -5.656 1.00 39.32 O

ATOM 1128 CB MET A 145 -14.140 -1.747 -5.355 1.00 38.56 C

ATOM 1129 CG MET A 145 -15.086 -1.447 -4.191 1.00 42.76 C

ATOM 1130 SD MET A 145 -16.859 -1.548 -4.577 1.00 47.10 S

ATOM 1131 CE MET A 145 -17.143 -3.273 -4.249 1.00 43.45 C

ATOM 1132 N ALA A 146 -12.439 0.899 -5.788 1.00 36.54 N

ATOM 1133 CA ALA A 146 -11.857 2.141 -5.302 1.00 36.10 C

ATOM 1134 C ALA A 146 -12.293 3.295 -6.174 1.00 40.86 C

ATOM 1135 O ALA A 146 -12.930 4.217 -5.669 1.00 39.91 O

ATOM 1136 CB ALA A 146 -10.344 2.034 -5.265 1.00 36.39 C

ATOM 1137 N LYS A 147 -12.052 3.188 -7.498 1.00 39.01 N

ATOM 1138 CA LYS A 147 -12.417 4.202 -8.498 1.00 38.58 C

ATOM 1139 C LYS A 147 -13.924 4.405 -8.556 1.00 40.61 C

ATOM 1140 O LYS A 147 -14.370 5.548 -8.662 1.00 40.17 O

ATOM 1141 CB LYS A 147 -11.832 3.853 -9.878 1.00 41.27 C

ATOM 1142 CG LYS A 147 -10.325 4.061 -9.950 1.00 58.69 C

ATOM 1143 CD LYS A 147 -9.738 3.645 -11.289 1.00 72.49 C

ATOM 1144 CE LYS A 147 -8.251 3.915 -11.364 1.00 86.66 C

ATOM 1145 NZ LYS A 147 -7.941 5.367 -11.507 1.00 95.56 N

ATOM 1146 N GLU A 148 -14.702 3.302 -8.417 1.00 35.86 N

ATOM 1147 CA GLU A 148 -16.165 3.327 -8.394 1.00 35.61 C

ATOM 1148 C GLU A 148 -16.673 4.166 -7.226 1.00 39.59 C

ATOM 1149 O GLU A 148 -17.470 5.076 -7.445 1.00 39.44 O

ATOM 1150 CB GLU A 148 -16.736 1.906 -8.310 1.00 37.17 C

ATOM 1151 CG GLU A 148 -18.253 1.846 -8.387 1.00 47.74 C

ATOM 1152 CD GLU A 148 -18.901 0.930 -7.370 1.00 66.59 C

ATOM 1153 OE1 GLU A 148 -18.762 -0.307 -7.508 1.00 60.65 O

ATOM 1154 OE2 GLU A 148 -19.558 1.450 -6.439 1.00 60.39 O

ATOM 1155 N ILE A 149 -16.178 3.882 -5.992 1.00 35.82 N

ATOM 1156 CA ILE A 149 -16.556 4.587 -4.755 1.00 34.93 C

ATOM 1157 C ILE A 149 -15.781 5.916 -4.595 1.00 39.25 C

ATOM 1158 O ILE A 149 -15.829 6.535 -3.532 1.00 39.01 O

ATOM 1159 CB ILE A 149 -16.469 3.669 -3.485 1.00 37.81 C

ATOM 1160 CG1 ILE A 149 -15.010 3.382 -3.044 1.00 38.39 C

ATOM 1161 CG2 ILE A 149 -17.272 2.367 -3.637 1.00 37.57 C

ATOM 1162 CD1 ILE A 149 -14.654 4.018 -1.775 1.00 43.71 C

ATOM 1163 N GLY A 150 -15.074 6.327 -5.647 1.00 36.25 N

ATOM 1164 CA GLY A 150 -14.312 7.570 -5.694 1.00 36.20 C

ATOM 1165 C GLY A 150 -13.151 7.687 -4.727 1.00 41.19 C

ATOM 1166 O GLY A 150 -12.786 8.803 -4.343 1.00 41.69 O

ATOM 1167 N ALA A 151 -12.550 6.548 -4.331 1.00 37.79 N

ATOM 1168 CA ALA A 151 -11.403 6.537 -3.414 1.00 37.66 C

ATOM 1169 C ALA A 151 -10.214 7.248 -4.041 1.00 41.72 C

ATOM 1170 O ALA A 151 -10.000 7.162 -5.257 1.00 42.14 O

ATOM 1171 CB ALA A 151 -11.021 5.114 -3.023 1.00 38.05 C

ATOM 1172 N VAL A 152 -9.496 8.009 -3.209 1.00 36.70 N

ATOM 1173 CA VAL A 152 -8.304 8.778 -3.555 1.00 35.88 C

ATOM 1174 C VAL A 152 -7.169 7.806 -3.958 1.00 37.34 C

ATOM 1175 O VAL A 152 -6.394 8.097 -4.867 1.00 37.91 O

ATOM 1176 CB VAL A 152 -7.947 9.687 -2.338 1.00 40.12 C

ATOM 1177 CG1 VAL A 152 -6.461 10.057 -2.282 1.00 40.13 C

ATOM 1178 CG2 VAL A 152 -8.818 10.937 -2.319 1.00 39.74 C

ATOM 1179 N LYS A 153 -7.115 6.644 -3.295 1.00 30.95 N

ATOM 1180 CA LYS A 153 -6.108 5.607 -3.462 1.00 29.10 C

ATOM 1181 C LYS A 153 -6.680 4.243 -3.036 1.00 30.84 C

ATOM 1182 O LYS A 153 -7.660 4.185 -2.287 1.00 29.61 O

ATOM 1183 CB LYS A 153 -4.885 5.972 -2.588 1.00 30.67 C

ATOM 1184 CG LYS A 153 -3.639 5.118 -2.792 1.00 36.80 C

ATOM 1185 CD LYS A 153 -2.502 5.900 -3.412 1.00 45.00 C

ATOM 1186 CE LYS A 153 -1.207 5.140 -3.268 1.00 59.40 C

ATOM 1187 NZ LYS A 153 -0.037 5.935 -3.726 1.00 69.39 N

ATOM 1188 N TYR A 154 -6.069 3.153 -3.546 1.00 27.11 N

ATOM 1189 CA TYR A 154 -6.360 1.755 -3.215 1.00 26.13 C

ATOM 1190 C TYR A 154 -5.048 1.132 -2.774 1.00 30.13 C

ATOM 1191 O TYR A 154 -4.053 1.231 -3.486 1.00 31.30 O

ATOM 1192 CB TYR A 154 -6.970 0.971 -4.388 1.00 25.80 C

ATOM 1193 CG TYR A 154 -7.062 -0.515 -4.114 1.00 26.75 C

ATOM 1194 CD1 TYR A 154 -7.970 -1.019 -3.187 1.00 28.44 C

ATOM 1195 CD2 TYR A 154 -6.194 -1.414 -4.733 1.00 27.39 C

ATOM 1196 CE1 TYR A 154 -8.028 -2.381 -2.897 1.00 28.80 C

ATOM 1197 CE2 TYR A 154 -6.243 -2.781 -4.449 1.00 27.61 C

ATOM 1198 CZ TYR A 154 -7.162 -3.259 -3.531 1.00 33.24 C

ATOM 1199 OH TYR A 154 -7.234 -4.601 -3.260 1.00 32.73 O

ATOM 1200 N LEU A 155 -5.049 0.510 -1.602 1.00 25.76 N

ATOM 1201 CA LEU A 155 -3.872 -0.088 -0.996 1.00 25.31 C

ATOM 1202 C LEU A 155 -4.187 -1.430 -0.398 1.00 29.75 C

ATOM 1203 O LEU A 155 -5.281 -1.644 0.121 1.00 31.60 O

ATOM 1204 CB LEU A 155 -3.328 0.824 0.119 1.00 25.50 C

ATOM 1205 CG LEU A 155 -2.698 2.160 -0.296 1.00 30.65 C

ATOM 1206 CD1 LEU A 155 -2.621 3.094 0.878 1.00 31.51 C

ATOM 1207 CD2 LEU A 155 -1.317 1.980 -0.899 1.00 30.12 C

ATOM 1208 N GLU A 156 -3.211 -2.319 -0.434 1.00 25.26 N

ATOM 1209 CA GLU A 156 -3.286 -3.651 0.144 1.00 25.10 C

ATOM 1210 C GLU A 156 -2.260 -3.713 1.267 1.00 27.77 C

ATOM 1211 O GLU A 156 -1.312 -2.925 1.265 1.00 26.40 O

ATOM 1212 CB GLU A 156 -3.039 -4.736 -0.919 1.00 26.68 C

ATOM 1213 CG GLU A 156 -4.129 -4.767 -1.990 1.00 40.34 C

ATOM 1214 CD GLU A 156 -4.056 -5.853 -3.051 1.00 57.56 C

ATOM 1215 OE1 GLU A 156 -2.964 -6.434 -3.251 1.00 62.41 O

ATOM 1216 OE2 GLU A 156 -5.095 -6.113 -3.698 1.00 36.56 O

ATOM 1217 N CYS A 157 -2.480 -4.588 2.257 1.00 25.16 N

ATOM 1218 CA CYS A 157 -1.583 -4.731 3.400 1.00 26.57 C

ATOM 1219 C CYS A 157 -1.826 -6.008 4.185 1.00 31.19 C

ATOM 1220 O CYS A 157 -2.843 -6.676 4.016 1.00 29.53 O

ATOM 1221 CB CYS A 157 -1.660 -3.507 4.315 1.00 27.67 C

ATOM 1222 SG CYS A 157 -3.258 -3.310 5.151 1.00 32.25 S

ATOM 1223 N SER A 158 -0.900 -6.305 5.090 1.00 29.54 N

ATOM 1224 CA SER A 158 -1.008 -7.403 6.028 1.00 30.08 C

ATOM 1225 C SER A 158 -0.495 -6.886 7.359 1.00 35.61 C

ATOM 1226 O SER A 158 0.627 -6.367 7.417 1.00 37.02 O

ATOM 1227 CB SER A 158 -0.190 -8.607 5.574 1.00 33.55 C

ATOM 1228 OG SER A 158 -0.259 -9.646 6.540 1.00 40.25 O

ATOM 1229 N ALA A 159 -1.314 -6.994 8.419 1.00 30.00 N

ATOM 1230 CA ALA A 159 -0.892 -6.590 9.755 1.00 29.24 C

ATOM 1231 C ALA A 159 0.018 -7.697 10.322 1.00 32.45 C

ATOM 1232 O ALA A 159 0.855 -7.426 11.187 1.00 31.53 O

ATOM 1233 CB ALA A 159 -2.104 -6.385 10.651 1.00 29.92 C

ATOM 1234 N LEU A 160 -0.159 -8.948 9.825 1.00 28.73 N

ATOM 1235 CA LEU A 160 0.617 -10.121 10.225 1.00 28.50 C

ATOM 1236 C LEU A 160 2.088 -9.978 9.781 1.00 35.19 C

ATOM 1237 O LEU A 160 2.994 -10.172 10.599 1.00 34.57 O

ATOM 1238 CB LEU A 160 -0.017 -11.418 9.654 1.00 27.87 C

ATOM 1239 CG LEU A 160 0.416 -12.747 10.298 1.00 31.46 C

ATOM 1240 CD1 LEU A 160 -0.646 -13.789 10.138 1.00 31.64 C

ATOM 1241 CD2 LEU A 160 1.710 -13.278 9.690 1.00 33.97 C

ATOM 1242 N THR A 161 2.319 -9.665 8.490 1.00 33.29 N

ATOM 1243 CA THR A 161 3.676 -9.527 7.961 1.00 33.71 C

ATOM 1244 C THR A 161 4.154 -8.067 7.958 1.00 38.39 C

ATOM 1245 O THR A 161 5.329 -7.821 7.672 1.00 37.88 O

ATOM 1246 CB THR A 161 3.802 -10.201 6.582 1.00 41.30 C

ATOM 1247 OG1 THR A 161 3.247 -9.348 5.579 1.00 43.26 O

ATOM 1248 CG2 THR A 161 3.158 -11.586 6.538 1.00 37.54 C

ATOM 1249 N GLN A 162 3.240 -7.105 8.267 1.00 35.69 N

ATOM 1250 CA GLN A 162 3.461 -5.642 8.287 1.00 35.86 C

ATOM 1251 C GLN A 162 3.757 -5.051 6.882 1.00 40.10 C

ATOM 1252 O GLN A 162 4.101 -3.875 6.765 1.00 40.02 O

ATOM 1253 CB GLN A 162 4.519 -5.218 9.327 1.00 37.03 C

ATOM 1254 CG GLN A 162 4.022 -5.357 10.761 1.00 49.93 C

ATOM 1255 CD GLN A 162 4.607 -4.316 11.673 1.00 71.51 C

ATOM 1256 OE1 GLN A 162 4.587 -3.109 11.403 1.00 65.45 O

ATOM 1257 NE2 GLN A 162 5.119 -4.764 12.795 1.00 70.50 N

ATOM 1258 N ARG A 163 3.567 -5.858 5.827 1.00 36.85 N

ATOM 1259 CA ARG A 163 3.799 -5.485 4.435 1.00 37.10 C

ATOM 1260 C ARG A 163 2.693 -4.544 3.959 1.00 40.26 C

ATOM 1261 O ARG A 163 1.518 -4.909 4.021 1.00 41.33 O

ATOM 1262 CB ARG A 163 3.843 -6.755 3.556 1.00 39.00 C

ATOM 1263 CG ARG A 163 4.463 -6.559 2.167 1.00 50.80 C

ATOM 1264 CD ARG A 163 4.429 -7.829 1.323 1.00 61.00 C

ATOM 1265 NE ARG A 163 5.050 -8.968 2.007 1.00 73.38 N

ATOM 1266 CZ ARG A 163 6.347 -9.265 1.964 1.00 91.34 C

ATOM 1267 NH1 ARG A 163 7.183 -8.524 1.242 1.00 74.75 N

ATOM 1268 NH2 ARG A 163 6.817 -10.309 2.636 1.00 81.23 N

ATOM 1269 N GLY A 164 3.081 -3.358 3.493 1.00 34.06 N

ATOM 1270 CA GLY A 164 2.165 -2.346 2.975 1.00 32.84 C

ATOM 1271 C GLY A 164 1.505 -1.466 4.020 1.00 34.45 C

ATOM 1272 O GLY A 164 0.810 -0.515 3.670 1.00 32.96 O

ATOM 1273 N LEU A 165 1.745 -1.767 5.305 1.00 31.36 N

ATOM 1274 CA LEU A 165 1.185 -1.104 6.485 1.00 31.43 C

ATOM 1275 C LEU A 165 1.618 0.376 6.661 1.00 36.11 C

ATOM 1276 O LEU A 165 0.762 1.251 6.844 1.00 35.14 O

ATOM 1277 CB LEU A 165 1.532 -1.953 7.711 1.00 31.55 C

ATOM 1278 CG LEU A 165 0.940 -1.567 9.042 1.00 37.85 C

ATOM 1279 CD1 LEU A 165 -0.577 -1.726 9.047 1.00 38.33 C

ATOM 1280 CD2 LEU A 165 1.523 -2.428 10.127 1.00 42.16 C

ATOM 1281 N LYS A 166 2.936 0.650 6.594 1.00 33.26 N

ATOM 1282 CA LYS A 166 3.487 2.001 6.715 1.00 33.04 C

ATOM 1283 C LYS A 166 2.929 2.890 5.596 1.00 37.61 C

ATOM 1284 O LYS A 166 2.722 4.083 5.820 1.00 38.65 O

ATOM 1285 CB LYS A 166 5.026 1.962 6.646 1.00 35.74 C

ATOM 1286 CG LYS A 166 5.722 3.240 7.119 1.00 54.81 C

ATOM 1287 CD LYS A 166 6.984 3.549 6.289 1.00 64.60 C

ATOM 1288 CE LYS A 166 7.828 4.687 6.832 1.00 66.01 C

ATOM 1289 NZ LYS A 166 7.109 5.993 6.832 1.00 69.94 N

ATOM 1290 N THR A 167 2.675 2.297 4.402 1.00 32.91 N

ATOM 1291 CA THR A 167 2.167 2.997 3.219 1.00 31.70 C

ATOM 1292 C THR A 167 0.763 3.512 3.462 1.00 32.39 C

ATOM 1293 O THR A 167 0.500 4.681 3.207 1.00 31.20 O

ATOM 1294 CB THR A 167 2.299 2.130 1.942 1.00 37.86 C

ATOM 1295 OG1 THR A 167 3.628 1.608 1.853 1.00 41.36 O

ATOM 1296 CG2 THR A 167 1.998 2.910 0.674 1.00 31.39 C

ATOM 1297 N VAL A 168 -0.117 2.637 3.971 1.00 27.98 N

ATOM 1298 CA VAL A 168 -1.520 2.893 4.294 1.00 27.11 C

ATOM 1299 C VAL A 168 -1.680 4.226 5.039 1.00 34.08 C

ATOM 1300 O VAL A 168 -2.406 5.104 4.558 1.00 34.40 O

ATOM 1301 CB VAL A 168 -2.140 1.687 5.069 1.00 29.49 C

ATOM 1302 CG1 VAL A 168 -3.454 2.057 5.766 1.00 28.97 C

ATOM 1303 CG2 VAL A 168 -2.326 0.473 4.165 1.00 28.74 C

ATOM 1304 N PHE A 169 -0.955 4.389 6.176 1.00 30.99 N

ATOM 1305 CA PHE A 169 -1.058 5.565 7.031 1.00 30.74 C

ATOM 1306 C PHE A 169 -0.239 6.742 6.493 1.00 36.12 C

ATOM 1307 O PHE A 169 -0.594 7.889 6.781 1.00 35.52 O

ATOM 1308 CB PHE A 169 -0.761 5.220 8.504 1.00 32.17 C

ATOM 1309 CG PHE A 169 -1.635 4.077 8.998 1.00 33.38 C

ATOM 1310 CD1 PHE A 169 -2.971 4.289 9.320 1.00 36.08 C

ATOM 1311 CD2 PHE A 169 -1.162 2.770 9.003 1.00 34.64 C

ATOM 1312 CE1 PHE A 169 -3.791 3.225 9.714 1.00 36.69 C

ATOM 1313 CE2 PHE A 169 -1.982 1.708 9.394 1.00 36.80 C

ATOM 1314 CZ PHE A 169 -3.288 1.941 9.752 1.00 35.30 C

ATOM 1315 N ASP A 170 0.773 6.474 5.633 1.00 33.71 N

ATOM 1316 CA ASP A 170 1.550 7.528 4.961 1.00 33.89 C

ATOM 1317 C ASP A 170 0.657 8.224 3.930 1.00 37.26 C

ATOM 1318 O ASP A 170 0.701 9.448 3.808 1.00 36.65 O

ATOM 1319 CB ASP A 170 2.790 6.947 4.262 1.00 36.24 C

ATOM 1320 CG ASP A 170 4.124 7.203 4.949 1.00 52.04 C

ATOM 1321 OD1 ASP A 170 4.129 7.837 6.034 1.00 52.95 O

ATOM 1322 OD2 ASP A 170 5.168 6.775 4.397 1.00 60.56 O

ATOM 1323 N GLU A 171 -0.188 7.432 3.233 1.00 34.21 N

ATOM 1324 CA GLU A 171 -1.145 7.868 2.212 1.00 34.15 C

ATOM 1325 C GLU A 171 -2.367 8.512 2.837 1.00 39.18 C

ATOM 1326 O GLU A 171 -3.026 9.352 2.207 1.00 37.04 O

ATOM 1327 CB GLU A 171 -1.542 6.710 1.277 1.00 35.49 C

ATOM 1328 CG GLU A 171 -0.438 6.263 0.325 1.00 46.34 C

ATOM 1329 CD GLU A 171 0.099 7.286 -0.666 1.00 75.53 C

ATOM 1330 OE1 GLU A 171 -0.681 8.153 -1.127 1.00 73.83 O

ATOM 1331 OE2 GLU A 171 1.299 7.192 -1.014 1.00 73.81 O

ATOM 1332 N ALA A 172 -2.659 8.126 4.092 1.00 38.32 N

ATOM 1333 CA ALA A 172 -3.740 8.709 4.882 1.00 38.61 C

ATOM 1334 C ALA A 172 -3.353 10.179 5.113 1.00 43.28 C

ATOM 1335 O ALA A 172 -4.158 11.075 4.856 1.00 43.20 O

ATOM 1336 CB ALA A 172 -3.870 7.974 6.207 1.00 39.21 C

ATOM 1337 N ILE A 173 -2.083 10.415 5.500 1.00 40.03 N

ATOM 1338 CA ILE A 173 -1.534 11.753 5.703 1.00 39.90 C

ATOM 1339 C ILE A 173 -1.498 12.492 4.358 1.00 45.54 C

ATOM 1340 O ILE A 173 -1.952 13.632 4.285 1.00 44.73 O

ATOM 1341 CB ILE A 173 -0.178 11.693 6.459 1.00 42.31 C

ATOM 1342 CG1 ILE A 173 -0.398 11.148 7.884 1.00 42.12 C

ATOM 1343 CG2 ILE A 173 0.512 13.066 6.511 1.00 42.89 C

ATOM 1344 CD1 ILE A 173 0.728 10.347 8.443 1.00 49.05 C

ATOM 1345 N ARG A 174 -1.065 11.801 3.288 1.00 44.37 N

ATOM 1346 CA ARG A 174 -1.016 12.336 1.917 1.00 45.45 C

ATOM 1347 C ARG A 174 -2.387 12.744 1.347 1.00 51.56 C

ATOM 1348 O ARG A 174 -2.439 13.664 0.531 1.00 51.07 O

ATOM 1349 CB ARG A 174 -0.310 11.361 0.962 1.00 46.12 C

ATOM 1350 CG ARG A 174 1.212 11.360 1.087 1.00 58.44 C

ATOM 1351 CD ARG A 174 1.842 10.411 0.086 1.00 72.90 C

ATOM 1352 NE ARG A 174 3.302 10.517 0.073 1.00 84.18 N

ATOM 1353 CZ ARG A 174 4.121 9.685 0.706 1.00 97.89 C

ATOM 1354 NH1 ARG A 174 3.636 8.669 1.409 1.00 80.24 N

ATOM 1355 NH2 ARG A 174 5.435 9.856 0.631 1.00 87.07 N

ATOM 1356 N ALA A 175 -3.489 12.070 1.772 1.00 49.83 N

ATOM 1357 CA ALA A 175 -4.859 12.376 1.324 1.00 49.96 C

ATOM 1358 C ALA A 175 -5.338 13.702 1.914 1.00 55.92 C

ATOM 1359 O ALA A 175 -6.208 14.366 1.338 1.00 55.40 O

ATOM 1360 CB ALA A 175 -5.807 11.258 1.706 1.00 50.50 C

ATOM 1361 N VAL A 176 -4.743 14.080 3.064 1.00 53.69 N

ATOM 1362 CA VAL A 176 -4.936 15.348 3.778 1.00 54.11 C

ATOM 1363 C VAL A 176 -3.698 16.176 3.338 1.00 59.92 C

ATOM 1364 O VAL A 176 -2.982 15.726 2.440 1.00 60.21 O

ATOM 1365 CB VAL A 176 -5.003 15.118 5.329 1.00 57.65 C

ATOM 1366 CG1 VAL A 176 -5.503 16.359 6.060 1.00 57.30 C

ATOM 1367 CG2 VAL A 176 -5.887 13.922 5.678 1.00 57.36 C

ATOM 1368 N LEU A 177 -3.442 17.359 3.939 1.00 57.04 N

ATOM 1369 CA LEU A 177 -2.272 18.210 3.661 1.00 79.27 C

ATOM 1370 C LEU A 177 -2.123 18.578 2.183 1.00 96.21 C

ATOM 1371 O LEU A 177 -1.963 19.748 1.849 1.00 55.06 O

ATOM 1372 CB LEU A 177 -0.982 17.537 4.186 1.00 79.25 C

ATOM 1373 CG LEU A 177 -0.562 17.807 5.641 1.00 84.07 C

ATOM 1374 CD1 LEU A 177 -1.575 17.280 6.646 1.00 84.05 C

ATOM 1375 CD2 LEU A 177 0.786 17.175 5.932 1.00 86.77 C

ATOM 1376 PB GDP A 201 -11.854 -6.295 13.331 1.00 24.55 P

ATOM 1377 O1B GDP A 201 -11.990 -5.754 11.921 1.00 24.29 O

ATOM 1378 O2B GDP A 201 -11.219 -5.214 14.201 1.00 27.74 O

ATOM 1379 O3B GDP A 201 -13.105 -6.829 13.858 1.00 23.58 O

ATOM 1380 O3A GDP A 201 -10.786 -7.475 13.286 1.00 24.94 O

ATOM 1381 PA GDP A 201 -9.542 -7.862 14.201 1.00 24.48 P

ATOM 1382 O1A GDP A 201 -8.541 -6.761 14.218 1.00 23.11 O

ATOM 1383 O2A GDP A 201 -10.106 -8.096 15.595 1.00 25.22 O

ATOM 1384 O5* GDP A 201 -8.972 -9.255 13.662 1.00 24.52 O

ATOM 1385 C5* GDP A 201 -9.837 -10.152 12.944 1.00 28.42 C

ATOM 1386 C4* GDP A 201 -9.212 -11.512 12.753 1.00 31.11 C

ATOM 1387 O4* GDP A 201 -8.282 -11.467 11.650 1.00 32.61 O

ATOM 1388 C3* GDP A 201 -8.422 -12.067 13.933 1.00 32.41 C

ATOM 1389 O3* GDP A 201 -8.513 -13.488 13.972 1.00 34.07 O

ATOM 1390 C2* GDP A 201 -6.996 -11.610 13.619 1.00 31.23 C

ATOM 1391 O2* GDP A 201 -6.022 -12.432 14.250 1.00 31.53 O

ATOM 1392 C1* GDP A 201 -6.971 -11.756 12.099 1.00 31.44 C

ATOM 1393 N9 GDP A 201 -6.066 -10.835 11.411 1.00 31.87 N

ATOM 1394 C8 GDP A 201 -6.136 -9.467 11.435 1.00 32.21 C

ATOM 1395 N7 GDP A 201 -5.381 -8.879 10.535 1.00 31.01 N

ATOM 1396 C5 GDP A 201 -4.765 -9.929 9.880 1.00 31.18 C

ATOM 1397 C6 GDP A 201 -3.905 -9.950 8.740 1.00 30.56 C

ATOM 1398 O6 GDP A 201 -3.456 -8.983 8.132 1.00 31.02 O

ATOM 1399 N1 GDP A 201 -3.599 -11.238 8.338 1.00 31.40 N

ATOM 1400 C2 GDP A 201 -4.027 -12.387 8.959 1.00 30.84 C

ATOM 1401 N2 GDP A 201 -3.641 -13.543 8.422 1.00 29.64 N

ATOM 1402 N3 GDP A 201 -4.813 -12.392 10.037 1.00 31.79 N

ATOM 1403 C4 GDP A 201 -5.161 -11.147 10.430 1.00 32.67 C

ATOM 1404 H50 GDP A 201 -10.808 -10.207 13.434 1.00 28.90 H

ATOM 1405 H51 GDP A 201 -9.979 -9.632 11.998 1.00 28.23 H

ATOM 1406 H40 GDP A 201 -9.968 -12.217 12.409 1.00 30.96 H

ATOM 1407 H30 GDP A 201 -8.787 -11.669 14.878 1.00 32.69 H

ATOM 1408 H3* GDP A 201 -8.726 -13.744 14.908 1.00 34.06 H

ATOM 1409 H20 GDP A 201 -6.853 -10.571 13.908 1.00 31.37 H

ATOM 1410 H2* GDP A 201 -6.287 -13.368 14.046 1.00 31.87 H

ATOM 1411 H10 GDP A 201 -6.726 -12.772 11.792 1.00 31.23 H

ATOM 1412 H80 GDP A 201 -6.757 -8.925 12.145 1.00 32.46 H

ATOM 1413 H1N GDP A 201 -3.029 -11.295 7.502 1.00 31.69 H

ATOM 1414 H21 GDP A 201 -3.931 -14.421 8.841 1.00 30.03 H

ATOM 1415 H22 GDP A 201 -3.057 -13.617 7.597 1.00 29.35 H

ATOM 1416 MC MG2 A 202 -11.915 -4.482 16.211 1.00 20.12 MG

ATOM 1416 DU1 MG2 A 202 -12.005 -4.482 16.211 1.00 20.12 MG

ATOM 1416 DU2 MG2 A 202 -11.825 -4.482 16.211 1.00 20.12 MG

ATOM 1416 DU3 MG2 A 202 -11.915 -4.572 16.211 1.00 20.12 MG

ATOM 1416 DU4 MG2 A 202 -11.915 -4.392 16.211 1.00 20.12 MG

ATOM 1416 DU5 MG2 A 202 -11.915 -4.482 16.301 1.00 20.12 MG

ATOM 1416 DU6 MG2 A 202 -11.915 -4.482 16.121 1.00 20.12 MG

ATOM 1417 O HOH A 301 -11.769 -12.426 -7.256 1.00 30.00 O

ATOM 1418 O HOH A 302 -18.097 4.920 15.836 1.00 30.00 O

ATOM 1419 O HOH A 303 -8.328 -13.776 -5.957 1.00 30.00 O

ATOM 1420 O HOH A 304 -14.813 1.992 23.553 1.00 26.73 O

ATOM 1421 O HOH A 305 -13.671 -5.260 15.955 1.00 24.83 O

ATOM 1422 O HOH A 306 -0.033 -0.555 0.933 1.00 24.76 O

ATOM 1423 O HOH A 307 -10.846 -3.084 20.189 1.00 10.56 O

ATOM 1424 O HOH A 308 -11.242 -6.144 17.151 1.00 24.43 O

ATOM 1425 O HOH A 309 -12.249 -3.664 18.000 1.00 8.25 O

ATOM 1426 O HOH A 310 -15.832 -4.224 5.121 1.00 8.64 O

ATOM 1427 O HOH A 311 -18.612 -10.063 6.337 1.00 21.87 O

ATOM 1428 O HOH A 312 -24.291 -11.021 -3.971 1.00 30.00 O

ATOM 1429 O HOH A 313 -12.563 -2.824 15.324 1.00 19.70 O

ATOM 1430 O HOH A 314 -9.717 -0.688 19.194 1.00 30.00 O

ATOM 1431 O HOH A 315 2.200 -10.464 2.975 1.00 37.72 O

ATOM 1432 O HOH A 316 -16.657 -13.776 2.647 1.00 30.00 O

ATOM 1433 O HOH A 317 -17.730 2.956 16.471 1.00 16.35 O

ATOM 1434 O HOH A 318 4.599 7.803 17.670 1.00 31.75 O

ATOM 1435 O HOH A 319 3.474 5.146 17.386 1.00 21.75 O

ATOM 1436 O HOH A 320 -4.146 -10.107 -7.392 1.00 22.14 O

ATOM 1437 O HOH A 321 1.166 -12.955 -0.480 1.00 38.92 O

ATOM 1438 O HOH A 322 2.776 16.531 17.209 1.00 30.00 O

ATOM 1439 O HOH A 323 -9.901 18.677 12.505 1.00 33.86 O

ATOM 1 C9 MOL X 1 -20.901 -2.202 24.089

ATOM 2 C7 MOL X 1 -20.864 -1.286 22.851

ATOM 3 N MOL X 1 -19.675 -1.005 22.225

ATOM 4 C3 MOL X 1 -19.643 -0.159 21.082

ATOM 5 C4 MOL X 1 -18.426 0.148 20.413

ATOM 6 C5 MOL X 1 -18.425 0.967 19.312

ATOM 7 C8 MOL X 1 -22.076 -0.735 22.368

ATOM 8 C6 MOL X 1 -22.074 0.084 21.267

ATOM 9 N1 MOL X 1 -23.325 0.662 20.755

ATOM 10 C2 MOL X 1 -20.858 0.392 20.598

ATOM 11 C1 MOL X 1 -20.826 1.238 19.455

ATOM 12 C MOL X 1 -19.637 1.518 18.828

ATOM 13 N2 MOL X 1 -19.601 2.392 17.646

ATOM 14 C10 MOL X 1 -18.752 3.559 17.926

ATOM 15 N3 MOL X 1 -17.402 3.539 17.574

ATOM 16 C11 MOL X 1 -19.296 4.685 18.542

ATOM 17 C13 MOL X 1 -18.490 5.793 18.808

ATOM 18 C14 MOL X 1 -19.091 7.037 19.487

ATOM 19 N4 MOL X 1 -17.140 5.773 18.456

ATOM 20 C12 MOL X 1 -16.596 4.646 17.841

ATOM 21 N5 MOL X 1 -15.173 4.625 17.471

ATOM 22 C15 MOL X 1 -14.418 5.502 18.378

ATOM 23 C16 MOL X 1 -12.909 5.303 18.146

ATOM 24 C17 MOL X 1 -14.764 5.149 19.837

ATOM 25 C18 MOL X 1 -13.506 5.303 20.714

ATOM 26 C19 MOL X 1 -13.904 5.223 22.199

ATOM 27 N6 MOL X 1 -12.721 5.466 23.037

ATOM 28 C21 MOL X 1 -12.856 6.765 23.711

ATOM 29 C23 MOL X 1 -12.826 7.893 22.663

ATOM 30 C20 MOL X 1 -12.605 4.401 24.043

ATOM 31 C22 MOL X 1 -11.453 4.731 25.011
